# ATRX deficiency drives aberrant type I interferon signalling through cGAS-dependent transcriptional dysregulation

**DOI:** 10.1038/s41467-026-78094-8

**Published:** 2026-10-08

**Authors:** Marie-Thérèse A. El-Daher, Eve L. White, Andreas Fellas, Graeme Grimes, Simone Pelliciari, Gaofeng Zhu, Tom A. Tait, Carolina Uggenti, Natalie Blair, Erisa Nita, Philippe Gautier, Hywel Dunn-Davies, Sophie Glen, Somdutta Dhir, Mathieu P. Rodero, Gillian I. Rice, Luis Seabra, Richard Clark, Angie Fawkes, Joseph A. Marsh, Minke HW Huibers, Joost B. Koedijk, Erika Van Nieuwenhove, John H. Livingston, Evangeline Wassmer, Swati Naik, Julie Vogt, Bertrand Isidor, Tom D. Deegan, Alice Lepelley, Rebekah Tillotson, Patrick Revy, Richard J. Gibbons, Yanick J. Crow

**Affiliations:** 1 MRC Human Genetics Unit, Institute of Genetics and Cancer, University of Edinburgh, Edinburgh, UK; 2 Institute of Medical Biology, Polish Academy of Sciences, Łódz´, Poland; 3 Institute of Genetics and Cancer, University of Edinburgh, Edinburgh, UK; 4 Division of Psychiatry, Royal Edinburgh Hospital, University of Edinburgh, Edinburgh, UK; 5 Centre for Genomic and Experimental Medicine, Institute of Genetics and Cancer, University of Edinburgh, Edinburgh, UK; 6 Laboratory of Neurogenetics and Neuroinflammation, INSERM UMR 1163, Imagine Institute, Université Paris Cité, Paris, France; 7 Division of Evolution, Infection and Genomic Sciences, School of Biological Sciences, Faculty of Biology, Medicine and Health, University of Manchester, Manchester Academic Health Science Centre, Manchester, UK; 8 Edinburgh Clinical Research Facility, Western General Hospital, University of Edinburgh, Edinburgh, UK; 9 Prinses Máxima Centrum, Centrum for Pediatric Oncology, Utrecht, The Netherlands; 10 Department of Genetics, University Medical Center Utrecht, Utrecht, The Netherlands; 11 Department of Paediatric Immunology and Rheumatology, Wilhelmina Children’s Hospital/ University Medical Center Utrecht, Utrecht, The Netherlands; 12 Faculty of Medicine, Utrecht University, Utrecht, The Netherlands; 13 Department of Paediatric Neurology, Leeds Teaching Hospitals NHS Trust, Leeds, UK; 14 Department of Paediatric Neurology, Birmingham Children’s Hospital, Birmingham Women’s and Children’s NHS Foundation Trust, Birmingham, UK; 15 Institute of Health and Neurodevelopment, Aston University, Birmingham, UK; 16 West Midlands Regional Genetics Service, Birmingham Women’s and Children’s NHS Foundation Trust, Birmingham, UK; 17 Service de Génétique Médicale, CHU Nantes, Nantes, France; 18 L’institut du Thorax, Université de Nantes, CNRS, INSERM, Nantes, France; 19 Laboratory of Genome Dynamics in Human Diseases, INSERM UMR 1163, Imagine Institute, Université Paris Cité, Paris, France; 20 Laboratoire labellisé Ligue 2023, Imagine Institute, Paris Cité University, Paris, France; 21 MRC Molecular Haematology Unit, MRC Weatherall Institute of Molecular Medicine, Radcliffe Department of Medicine, University of Oxford, Oxford, UK

## Abstract

The X-linked α-thalassaemia intellectual disability syndrome (ATRX) protein is a chromatin remodeller involved in transcriptional regulation and genome stability. While the importance of ATRX in development and malignancy is well recognised, its role in innate immunity is less well defined. Here we describe three unrelated patients with ATR-X syndrome carrying missense mutations in the ATPase domain of ATRX, each manifesting features of severe inflammation accompanied by persistent upregulation of interferon-stimulated gene expression in whole blood. Studies in patient-derived cells, CRISPR-engineered fibroblasts and neuronal models demonstrate that ATRX loss-of-function mutations drive enhanced type I interferon signalling through a cGAS-dependent mechanism uncoupled from the DNA sensing activity of cGAS. Mechanistically, ATRX deficiency disrupts the chromatin distribution of DAXX and H3.3, with cGAS essential for the changes in nucleosome composition and gene expression mediated by loss of ATRX. Thus, our study highlights a link between ATRX dysfunction and inflammation involving a non-canonical role of cGAS.

The X-linked α-thalassaemia intellectual disability syndrome (ATRX) protein acts as an ATP-dependent molecular motor involved in chromatin remodelling and transcriptional regulation^1^. Positioned predominantly in the nucleus, ATRX exhibits a broad genomic distribution, localising to heterochromatic features including silenced repetitive elements, telomeres and pericentromeres, and to euchromatin regions^2^. In complex with death domain-associated protein (DAXX), ATRX facilitates the deposition of the histone variant H3.3 at sites of nucleosome turnover in a replication-independent manner^3^, and as a key epigenetic regulator, disturbance of ATRX function can impact DNA replication, chromatin state and gene expression^4^. Germline mutations in *ATRX* cause a sex-linked recessive disorder associated with learning difficulties, referred to as ATR-X syndrome^5,6^. Furthermore, acquired somatic loss-of-function mutations in ATRX are characteristic of tumours that utilise the alternative lengthening of telomeres (ALT) pathway, an adaptive mechanism active in 10–15% of all cancers^7,8^. Notably, mutations in the ATRX/DAXX complex are present in 90% of ALT-positive gliomas, frequently co-occurring with mutations in isocitrate dehydrogenase (IDH)^9^, the latter facilitating tumour immune evasion by limiting the inflammatory response triggered by ATRX deficiency^10^. ATRX also contributes to innate immune defence by restricting viral gene expression through the promotion of heterochromatin formation^11^. These multiple functions of ATRX underscore its importance not only in development and cancer biology, but also in innate immunity.

While type I interferons are integral to the response to viral infection, establishing an anti-viral state through the upregulation of hundreds of interferon-stimulated genes (ISGs)^12^, inappropriate activation of an interferon response can be pathogenic, leading to tissue damage. This dichotomy is particularly well illustrated by the type I interferonopathies, Mendelian diseases characterised by chronically enhanced type I interferon signalling^13^. Type I interferons can be induced through the sensing of both foreign and self-nucleic acids by innate immune receptors, among them cyclic GMP-AMP synthase (cGAS)^14^. Upon binding to double-stranded DNA in a sequence-independent manner, cGAS catalyses the production of the second messenger 2′−3′ cyclic guanosine monophosphate-adenosine monophosphate (cGAMP) from GTP and ATP. cGAMP binds to the signalling adaptor stimulator of interferon genes (STING). Activated STING is transported from the endoplasmic reticulum (ER) to the ER-Golgi intermediate compartment and the Golgi, where it recruits Tank binding kinase 1 (TBK1), which in turn activates interferon regulatory factor 3 (IRF3) and nuclear factor kappa B subunit (NF-κB). Both of these transcription factors then translocate to the nucleus, leading to the induction of type I interferon and other pro-inflammatory cytokine genes^15^. Sensing of self-DNA by cGAS has been shown to occur in a number of type I interferonopathies, for example due to loss-of-function mutations in the three prime repair exonuclease TREX1^16^. While originally considered as an exclusively cytoplasmic protein, it is now recognised that cGAS also localises to the nucleus, where it is associated with nucleosomes, DNA replication forks, double-strand breaks and centromeres^17,18^. These observations suggest that cGAS may have functions beyond its canonical role in cytosolic DNA sensing.

Here, we report three unrelated patients with ATR-X syndrome carrying missense mutations in the ATPase domain of ATRX, each manifesting severe inflammation and increased ISG expression in whole blood. These mutations drive enhanced type I interferon signalling through a cGAS-dependent mechanism uncoupled from the DNA sensing activity of cGAS, where cGAS is essential for changes in nucleosome composition and gene expression mediated by loss of ATRX. Thus, this study highlights a link between ATRX dysfunction and inflammation involving a non-canonical role of cGAS, a process that depends on the interplay between the ATRX/DAXX complex, H3.3 and cGAS in regulating transcription.

## Results

### Substitutions in ATRX can cause type I interferon-mediated inflammation

**Fig. 1:**
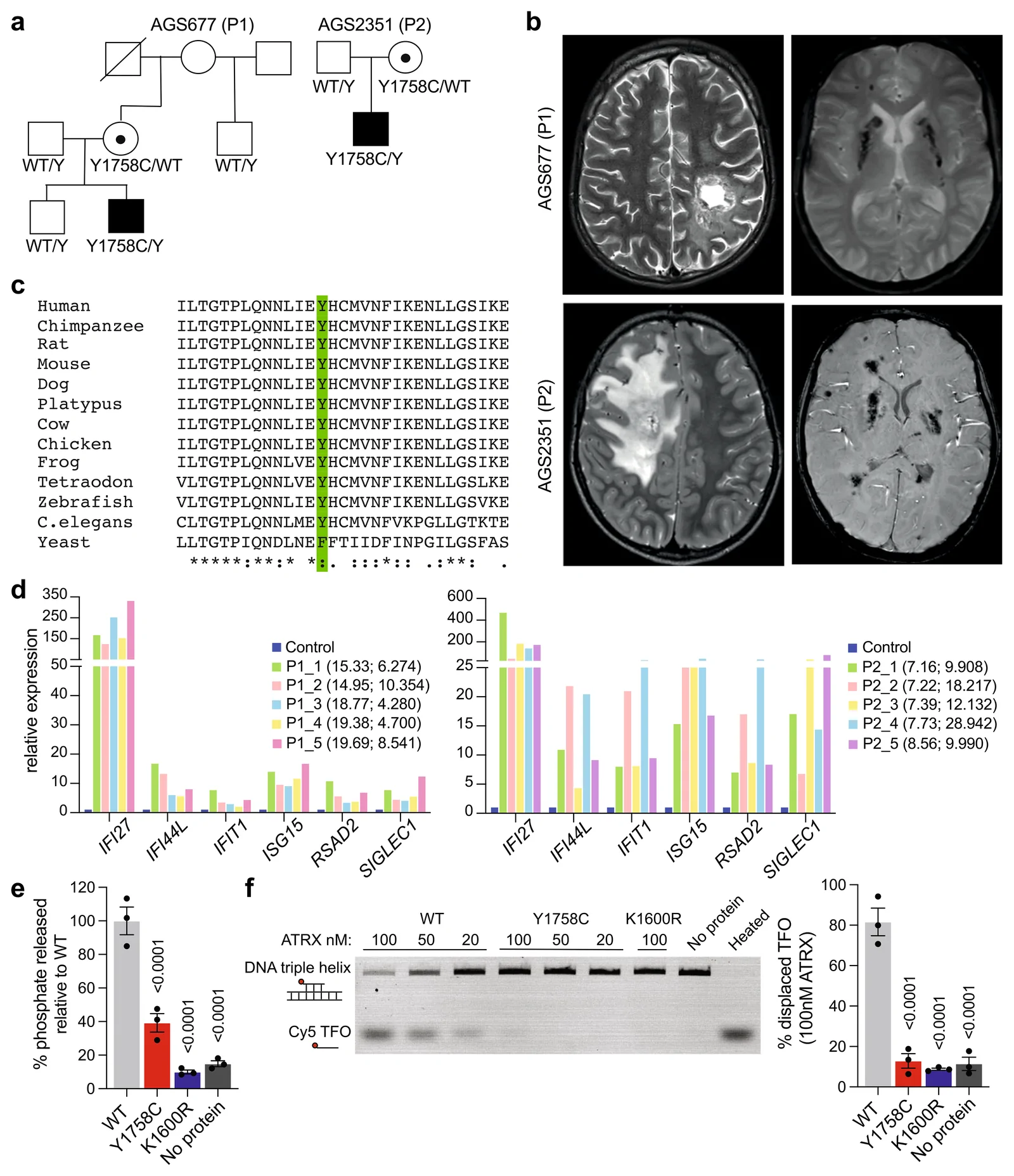
Loss of function of ATRX results in enhanced type I interferon signaling. **a** Pedigree structure of family AGS677 (P1) and family AGS2351 (P2). Circles and squares indicate female and male family members, respectively, with associated hemizygous (male) and heterozygous (female) genotypes given where tested; blackened squares denote affected status, dotted circles denote carrier status, and the diagonal line deceased status. WT = wild-type. **b** Representative cerebral MRI images demonstrating tumefactive lesions (top and bottom left: axial T2-weighted) and dense calcification (right: axial SWI in P1 and GRE in P2). **c** CLUSTAL Omega alignment of ATRX homologs. The position of the substituted Y1758 is indicated in green. **d** Relative expression values of six interferon- stimulated genes (ISGs) measured on five different occasions in whole blood of P1 (left) and P2 (right) by qPCR. The first number in brackets is the decimalised age in years at sampling, and the second number is the interferon score calculated from the median fold change in relative expression value for the panel of six ISGs indicated on the x-axis (normal <2.4). Colours relate to different dates at sampling. Dark blue bars represent the composite data of 29 controls. **e** ATPase activity of WT, Y1758C and catalytically dead K1600R ATRX recombinant proteins. The quantity of liberated phosphate was measured using 20 nM of ATRX protein in the presence of excess plasmid DNA and a no-protein control; values were normalised to the mean of all WT samples pooled together, set to 100%. **f** Assessment of DNA translocation by triple helix displacement. Left: representative agarose gel electrophoresis tracking the Cy5 TFO (triple helix forming oligo), top and bottom bands correspond to triple helix and free TFO, respectively. The concentration (nM) of ATRX protein used in each lane is indicated; right: triple helix displacement assay quantification. Percentage (%) of displaced TFO at 100 nM or no recombinant protein; values were normalised to the mean of all heat-denatured control samples pooled together, set to 100%, **e, f** three independent experiments; ordinary one-way ANOVA, two-stage linear step-up procedure of Benjamini, Krieger and Yekutieli; *q* values indicated on graph; mean ± SEM]. Source data are provided as a Source Data file.

As part of an ongoing protocol involving the agnostic screening of patients with uncharacterised disease phenotypes for an upregulation of type I interferon signalling, we identified two unrelated male patients (P1 and P2 from families AGS677 and AGS2351, respectively) (Fig. 1a) demonstrating similar cerebral white matter lesions and intracranial calcification, moderate to severe intellectual disability, and facial features consistent with ATR-X syndrome in P2 (Figs. 1b and S1a–c). Exome sequencing in P1 and P2 revealed the same p.(Tyr1758Cys) (Y1758C, c.5273A>G) substitution in the helicase domain of ATRX, which was maternally inherited in the absence of a family history of ATR-X or inflammatory disease in both cases. An unaffected brother and maternal uncle in family AGS677 were found not to carry the variant (Fig. 1a). This substitution is not present on gnomAD v4 (0/1,018,707 alleles) and is predicted as pathogenic by in silico analyses, with the tyrosine residue at position 1758 conserved to *C. elegans* (Fig. 1c). Clinically accredited genome-wide DNA methylation testing in P2 revealed a methylation episignature diagnostic of ATR-X syndrome^19,20^. As evidence of enhanced interferon signalling, ISG expression in whole blood was persistently elevated in both individuals on all occasions tested (six times between the ages of 14.95 and 23.1 years in P1, and eight times between the ages of 7.1 and 12.8 years in P2) (Figs. 1d and S1d–f). Cerebral white matter disease reminiscent of type I interferon-driven neuroinflammation^21^ has been reported in patients with ATR-X syndrome^22,23^, and gene set enrichment pathway analysis of publicly available RNA sequencing (RNA-seq) datasets^24–26^ identified an upregulation of interferon responses in *ATRX*-null induced pluripotent stem cells, *Atrx*-null mouse forebrain and *Atrx*-null mouse neuronal progenitor cells (Fig. S2a). Subsequently, we ascertained a third patient (P3, Fig. S2b) with ATR-X syndrome, manifesting severe developmental delay with characteristic facial features and a diagnostic methylation episignature, who presented in the first year of life with persistent fever, hepatitis and lymphocytic panniculitis associated with enhanced type I interferon signalling in blood, and highly elevated neopterin in the cerebrospinal fluid indicating neuroinflammation. Notably, the systemic inflammatory features experienced have responded to ‘anti-interferon’ therapy using the JAK1/2 inhibitor ruxolitinib. The maternally inherited p.(Ala2137Thr) (A2137T, c.6409G>A) substitution seen in this patient involves an evolutionarily conserved amino acid (Fig. S2c) in the SNF2 domain of the protein, is not present on gnomAD, and has been recorded twice on the DECI-PHER database as a cause of classical ATR-X syndrome in the absence of reported features of inflammation^27^.

### The Y1758C substitution is associated with reduced ATPase and translocase activity

Germline loss-of-function mutations in *ATRX* are non-viable^28^, so that mutations causing ATR-X syndrome act as hypomorphs. All previously analysed mutations causing ATR-X syndrome result in reduced ATRX protein levels to 7–50% of controls^29–31^, with some also demonstrated to impact binding to heterochromatin or chromatin remodelling activity^30,31^. Consistent with these observations, levels of ATRX protein were reduced in primary fibroblasts obtained from four patients with classical ATR-X syndrome and no reported inflammatory features (P4–P7). In contrast, ATRX levels were normal in primary fibroblasts from P1 and P2 (Fig. S3a). Given its localisation in the SNF2 domain, we hypothesised that the Y1758C substitution might impair the ATPase/helicase activity of ATRX. To explore this possibility, we expressed full-length wild-type (WT) and Y1758C human ATRX protein in yeast cells. As a negative control, we also generated the K1600R mutation, which abolishes ATRX ATPase activity (Fig. S3b and Table 1). We first observed that the Y1758C substitution reduced the ATPase activity of ATRX to ~40% of WT levels (Fig. 1e). Next, we used a triple helix displacement assay to test the ability of Y1758C ATRX to translocate along a duplex DNA and displace a third DNA strand. In this way, we found that Y1758C, like the K1600R catalytically dead mutant, lost the ability to remove the third strand (Fig. 1f). These results, as previously reported for two other disease-associated mutations in the SNF2 domain, V1552F and K1650N^31^, suggest that Y1758C ATRX is defective in ATPase and translocase activity, and hence primarily affects the function of ATRX in chromatin remodelling.

### Loss of function of ATRX results in enhanced type I interferon signalling

**Fig. 2:**
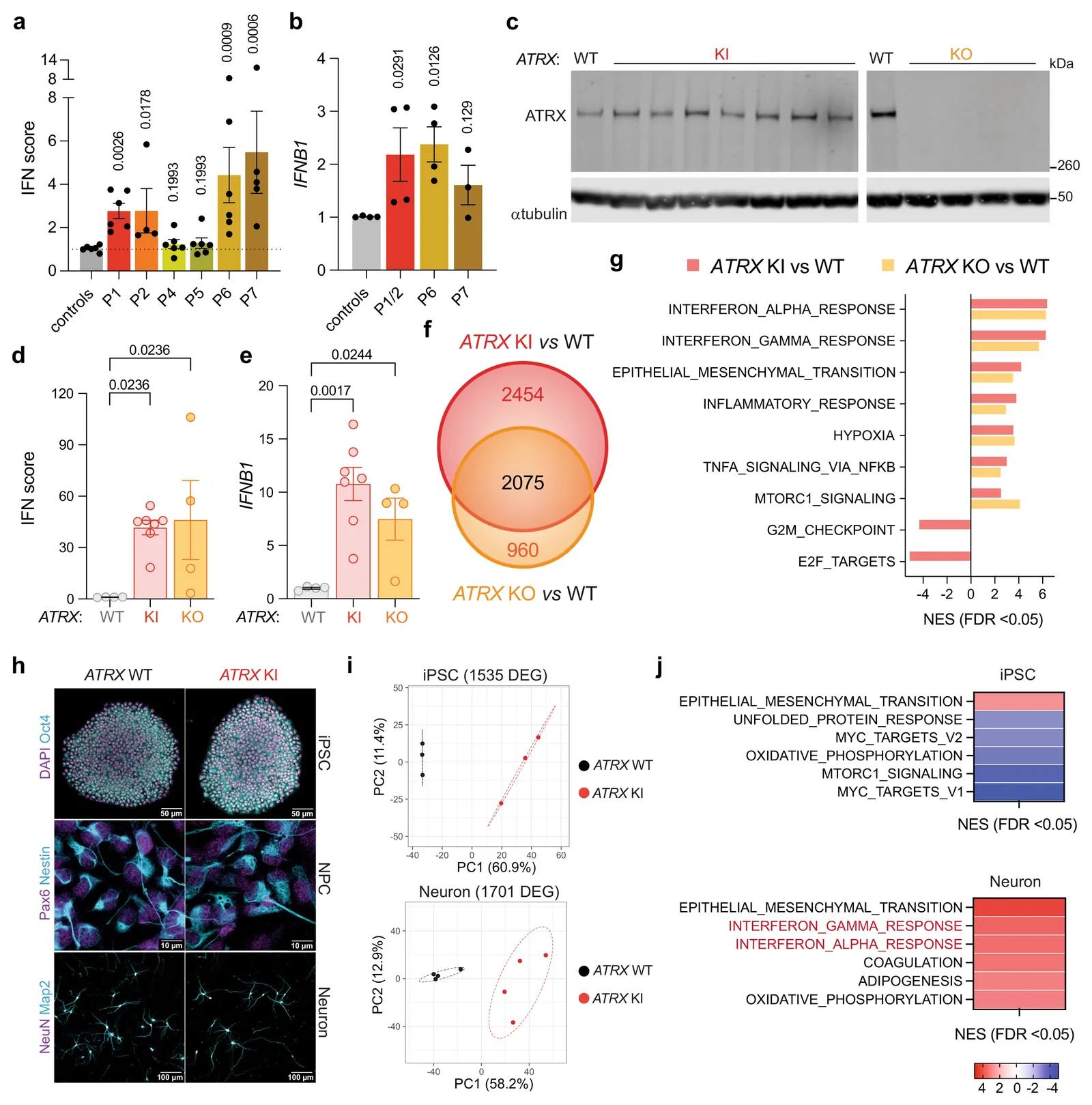
Enhanced interferon signalling in ATRX-mutated cells. **a** Interferon (IFN) score calculated by qPCR representing the median of the relative expression levels of *IFI27, RSAD2, MX1, ISG15, IFI44L, IFIT1, TNFAIP3* in primary fibroblasts from ATR-X syndrome patients and controls (normalised to one). **b** Relative expression level of *IFNB1* as in **(a). a, b** Each circle represents an independent experiment; Kruskal–Wallis test, two-stage linear step-up procedure of Benjamini, Krieger and Yekutieli. **c** ATRX protein expression assessed by Western blot in BJ-hTERT knock-in (KI) for the Y1758C mutation or knock-out (KO) for *ATRX*. IFN score **(d**) and expression of *IFNB1* **(e)** in BJ-hTERT WT, KI or KO for *ATRX* (calculated as in **a, b**); [**d, e** each circle represents the mean of an individual clone or the parental line across independent replicates. WT: clones 1, 2, *n* = 2; clone 3, *n* = 1; parental line, *n* = 5. KI: clones 1, 3, 4, 6, 7; *n* = 2; clone 2, *n* = 3; clone 5, *n* = 4. KO: clones 1, 4, *n* = 3; clone 2, *n* = 2; clone 3, *n* = 4; ordinary one-way ANOVA, two-stage linear step-up procedure of Benjamini, Krieger and Yekutieli]. **f** Differential gene expression analysis comparing three independent BJ-hTERT clones WT, KI or KO for *ATRX*. Top and bottom values represent the number of genes exclusively dysregulated in cells KI or KO for *ATRX*, respectively. The middle value represents the number of genes dys-regulated in both KI and KO *ATRX* cells compared to WT. Gene expression was determined by RNA-seq, and pairwise differential expression analysis was performed by *DESeq2*. **g** Differentially expressed genes identified in **(f)** were analysed with GSEA, and normalised enrichment scores (NES) of hallmark pathways with a false discovery rate (FDR) < 0.05 are displayed as a bar plot. **h** Immunofluorescence of induced pluripotent stem cells (iPSCs), neural progenitor cells (NPCs) and forebrain neurons in WT or KI *ATRX* mutant cells. Images are representative of four biological clones. **i** Principal component (PC) analysis of RNA-seq data. DEG: differentially expressed genes between WT and KI *ATRX*. **j** Heatmaps NES of hallmark pathways with FDR < 0.05. **a, b, d, e** *q* values indicated on graph, mean ± SEM]. Source data are provided as a Source Data file.

In primary fibroblasts from both P1 and P2, we recorded an elevated interferon score, based on the expression of a panel of 7 representative ISGs, and found the expression of the interferon beta gene (*IFNB1*) to be increased (Fig. 2a, b). The interferon score and *IFNB1* expression were also increased in two of the four (P4 to P7) primary fibroblast cell lines from patients with classical ATR-X syndrome that were available to us (Fig. 2a, b). Given the potential variability of genetic background in available patient material, we introduced the Y1758C mutation (referred to as knock-in (KI)) into BJ-5ta immortalised fibroblasts (referred to as BJ-hTERT), and also generated *ATRX* knock-out (KO) clones using CRISPR/Cas9 technology. As in patient primary fibroblasts, ATRX protein levels were unaffected in BJ-hTERT KI clones, while we could not detect ATRX protein in the engineered KO clones (Fig. 2c). ISG and *IFNB1* mRNA expression was elevated in both KI and KO *ATRX* clones (Fig. 2d, e), and increased type I interferon protein activity was observed using a reporter assay (Fig. S4a). Transcriptomic analysis by RNA-seq of three independent KI, KO and WT clones revealed 4529 and 3035 differentially expressed genes (adjusted *p*-value < 0.1) in *ATRX* KI and KO cells, respectively, compared to WT clones (Fig. 2f). Two thousand seventy-five genes were similarly dysregulated in both KI and KO *ATRX* cells, suggesting that, at least in part, the Y1758C mutation recapitulates ATRX loss of function. Gene set enrichment analysis of these expression data identified significant upregulation of the interferon response and of genes involved in epithelial-mesenchymal transition (Fig. 2g). Concordant with these data, short-hairpin (sh) RNA mediated knock-down of *ATRX* in the human monocytic cell line THP-1 resulted in increased ISG expression and type I interferon production (Fig. S4b–d).

Since ATR-X syndrome is primarily a disorder of the brain, we next introduced the Y1758C mutation into induced pluripotent stem cells (iPSCs). iPSCs KO for *ATRX* did not survive clonal expansion and hence could not be studied. Using three WT and three KI clones, we then differentiated iPSCs to neural progenitor cells (NPCs) and forebrain neurons (Fig. S4e). Immunostaining and global mRNA expression analysis using 3′ end RNA-seq confirmed the expression of expected iPSC, NPC and neuronal lineage markers (Figs. 2h and S4f). Mutation of *ATRX* led to marked differences in gene expression in both iPSCs and neurons (Fig. 2i), with functional pathway enrichment analysis identifying significant dysregulation of several transcriptional programmes, including the emergence of an interferon response in Y1758C KI neurons (Fig. 2j).

These observations indicate that, as in patient blood and patient-derived primary fibroblasts, multiple cell types mutated or null for *ATRX* demonstrate activation of type I interferon signalling.

### cGAS is required for the activation of interferon signalling in ATRX mutant cells

**Fig. 3:**
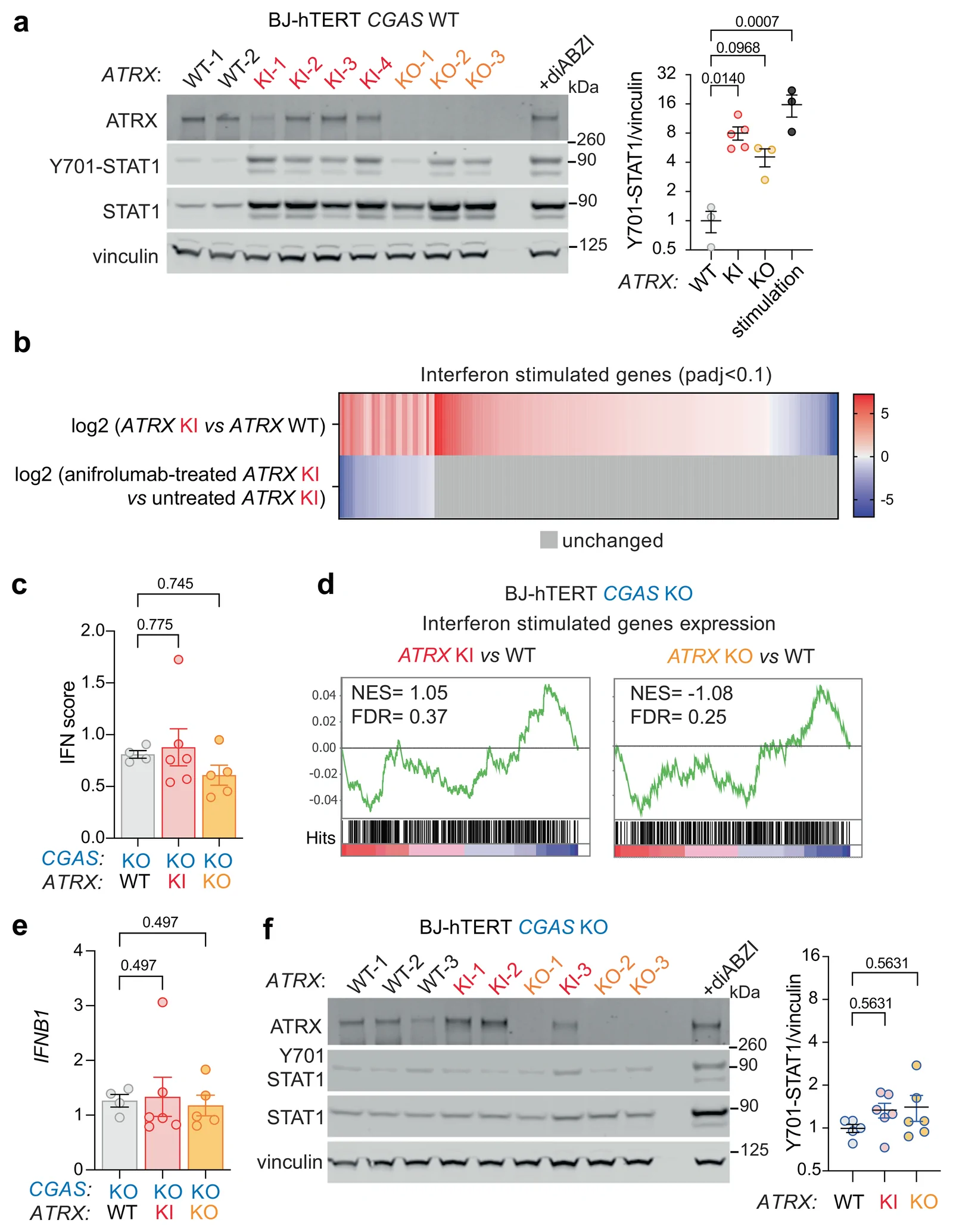
cGAS is required for the activation of interferon signalling in ATRX-deficient cells. **a** Left: immunoblot of ATRX, phosphorylated (Y701) and total STAT1, and vinculin in individual BJ-hTERT clones WT, KI (Y1758C) or KO for *ATRX*. Last lane: WT cells stimulated with diABZI for 24 h. Right: quantification of Y701-STAT1, [each circle represents the mean of an individual clone or the parental line across independent replicates. WT: parental line and clones 1, 2, *n* = 2; KI: clones 1, 5, *n* = 3; clones 2, 4, *n* = 1; clone 3, *n* = 2; KO: clone 1, *n* = 2; clone 2, *n* = 3; clone 3, *n* = 1; stimulation: each dot represents an independent experiment, *n* = 3]. **b** Effect of anifrolumab treatment on ISG expression in BJ-hTERT WT or KI for *ATRX*. Gene expression was determined by RNA-seq, and pairwise differential expression analysis was performed by *DESeq2*. In the untreated condition (top), ISGs differentially expressed between WT and KI cells are displayed. Upon treatment (bottom), genes whose expression decreased are indicated in blue, and genes showing no change in grey. **c** Interferon (IFN) score (median relative expression of *IFI27, RSAD2, MX1*,. *ISG15, IFI44L, IFIT1, TNFAIP3*) in BJ-hTERT KO for *CGAS* and either WT, KI, or KO for *ATRX*, [each circle represents the mean of an individual clone or the parental line across independent replicates. WT: parental line, *n* = 4; clones 1, 2, *n* = 3; clone 3, *n* = 2; KI: clones 1, 5, *n* = 4; clones 2, 3, 4, 6, *n* = 2; KO: clones 1–4, *n* = 2; clone 5, *n* = 3]. **d** The expression level of 444 canonical ISGs was determined in BJ-hTERT lacking *CGAS* by RNA-seq and analysed with GSEA; corresponding normalised enrichment scores (NES) and false discovery rate (FDR) are indicated. **e** *IFNB1* expression analysed as in (**c**). **f** Protein levels analysed as in (**a**) in cells KO for *CGAS*, [each circle represents the mean of an individual clone or the parental line across independent replicates. WT: parental line, clones 2, 3, *n* = 2; clones 1, 4, *n* = 1; KI: clone 1; *n* = 3; clones 2, 3, 4, *n* = 1; clones 5, 6, *n* = 2; KO: clones 1, 3, 4, 5, *n* = 1; clones 2, 6, *n* = 2. **a, c, d, e** Ordinary one-way ANOVA, two-stage linear step-up procedure of Benjamini, Krieger and Yekutieli, *q* values indicated on graph, mean ± SEM]. Source data are provided as a Source Data file.

The induction of type I interferon signalling involves host-encoded nucleic acid-binding pattern-recognition receptors. These include the cytosolic sensors of dsRNA (melanoma differentiation associated protein 5, MDA5, and retinoic acid inducible gene I, RIG-I), which signal through mitochondrial antiviral signalling protein (MAVS)^32^, and dsDNA sensors, most particularly cGAS, which signals to STING through the second messenger cGAMP^33^. Both RNA and DNA sensing pathways activate IRF3, leading to the subsequent induction of type I interferons expression. In turn, type I interferons are secreted and bind to the interferon-α/β receptor (IFNAR), initiating a JAK-STAT signalling cascade and phosphorylation process that induces the expression of ISGs. We detected increased levels of phosphorylated STAT1, a marker of interferon signalling through IFNAR, in both *ATRX* KI and KO cells, in the absence of any additional stimulation, and in control cells stimulated with the STING agonist diABZI (Fig. 3a). Exposing *ATRX* mutant cells to anifrolumab, a monoclonal antibody that blocks IFNAR signalling, led to the reduced expression of 35 of the 183 ISGs dysregulated in these cells prior to treatment (Fig. 3b), indicating a partial contribution of IFNAR downstream signalling in ATRX-mediated inflammation.

Next, we investigated the role of discrete nucleic acid sensors in the observed upregulation of interferon signalling. ISG expression remained elevated following *MAVS* depletion by siRNA in primary fibroblasts from patients with *ATRX* mutations (Fig. S5a, b) and in THP-1 cells KO for *MAVS* and knocked down for *ATRX* (Fig. S5c, d), suggesting that cytosolic RNA sensing is not involved in the interferon response induced by ATRX deficiency. To interrogate the role of DNA sensing, we examined the impact of ATRX loss of function in BJ-hTERT fibroblasts null for *CGAS*^34,35^ (Fig. S5e), and in CRISPR-engineered clones either KI or KO for *ATRX*. Notably, the increased expression of a curated list of 444 canonical ISGs (Supplementary Data 1) observed in ATRX-deficient cells was absent in cells also KO for *CGAS* (Fig. 3c, d). Of further note, while ATRX loss of function induced *IFNB1* upregulation and STAT1 phosphorylation in WT cells (Figs. 2e and 3a), *IFNB1* expression and STAT1 phosphorylation in cells KI or KO for *ATRX* were comparable to cells WT for *ATRX* on a *CGAS* KO background (Fig. 3e, f). Consistent with these observations, genome-wide transcriptomic analysis revealed little variation between WT cells and ATRX-deficient cells on a *CGAS* KO background (Fig. S5f), in contrast to cells WT for *CGAS* (Fig. S5g).

Collectively, these results demonstrate a requirement for cGAS in mediating the enhanced interferon response observed in the context of ATRX deficiency.

### While interferon induction in ATRX-deficient cells is cGAS-dependent, this does not involve the canonical DNA sensing pathway

**Fig. 4:**
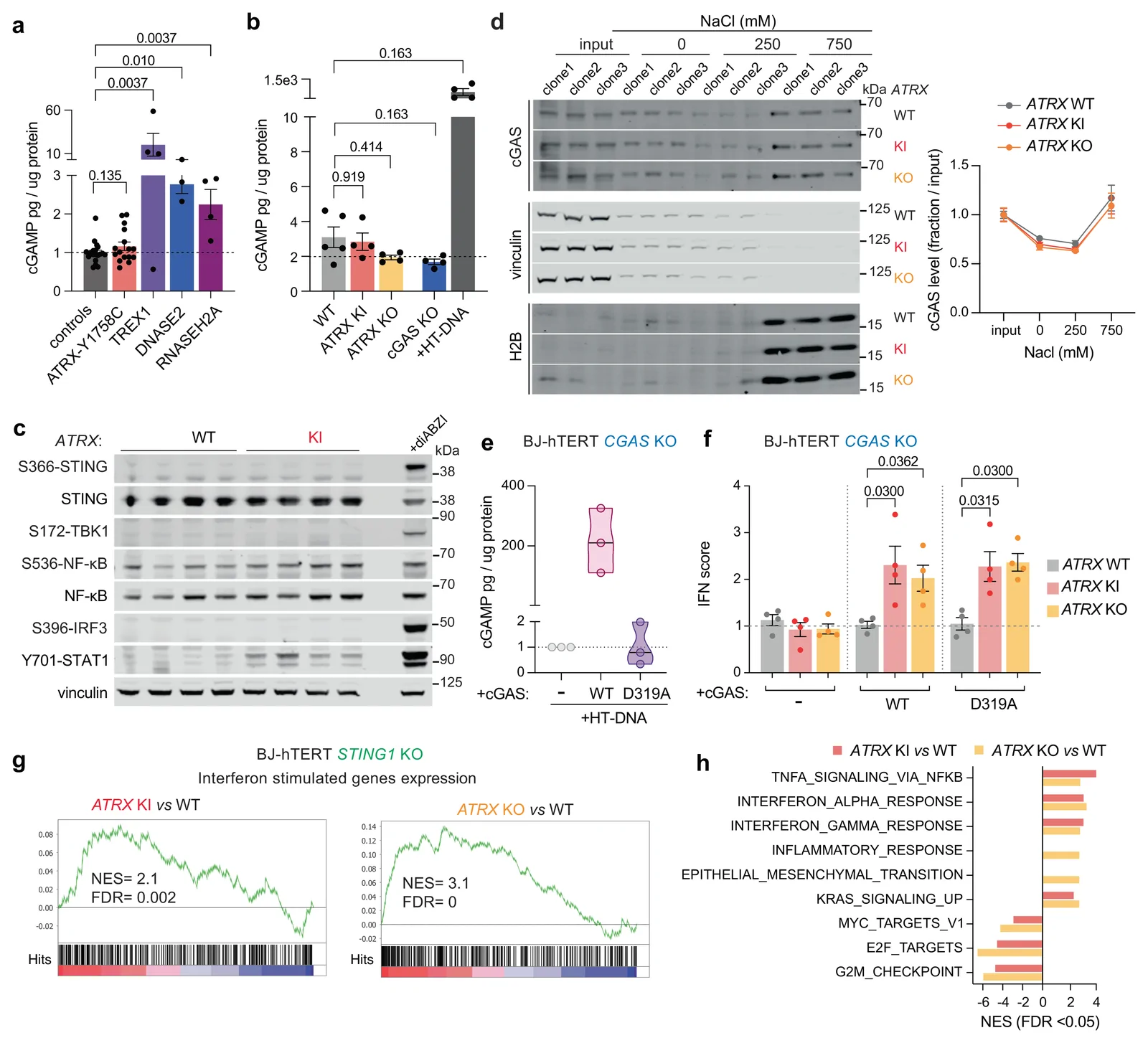
cGAS, but not its second messenger 2′3′cGAMP or STING, is required for the activation of interferon-stimulated genes in ATRX-deficient cells. **a** cGAMP levels measured in primary fibroblasts from controls, ATRX-Y1758C patient, and patients with biallelic mutations in *TREX1, DNASE2* or *RNASEH2A*, [each circle represents an independent replicate: controls, *n* = 19; ATRX-Y1758C, *n* = 16; TREX1, *n* = 4; DNASE2, *n* = 3; RNASEH2A, *n* = 4]. **b** cGAMP levels measured in unstimulated BJ-hTERT cells WT, KI (Y1758C) or KO for *ATRX* [each circle represents the mean of an individual clone or the parental line across independent replicates. WT: clones 1, 4, *n* = 2; clones 2, 3, *n* = 3; parental line, *n* = 1; KI: clone 1, *n* = 4; clone 2, *n* = 1; clones 3, 4, *n* = 3; KO: clones 1–4, *n* = 2]; KO for *CGAS*, and control cells stimulated with HT-DNA [each circle represents an independent replicate, parental line, *n* = 4]. **c** Immunoblots displaying the levels of phosphorylated STING (S366), phosphorylated and total forms of NF-κB, phosphorylated IRF3 (S396), TBK1 (S172) and STAT1 (Y701) in four independent clones of BJ-hTERT WT or KI for *ATRX*. Last lane: WT cells treated with diABZI for 5.5 h. **d** BJ-hTERT WT, KI or KO for *ATRX* were separated into cytosolic and nuclear fractions, followed by sequential elution of nuclear pellets with the indicated concentrations of NaCl. Quantification (right) of cGAS levels in the different fractions. Vinculin and H2B are cytosolic and chromatin markers, respectively [three independent clones, mean ± SEM]. **e** cGAMP levels measured in BJ-hTERT KO for *CGAS* transfected with lipofectamine only (−), WT or catalytically inactive (D319A mutant) cGAS and stimulated with HT-DNA for 4 h. Black bars represent the median of three independent experiments (circles). **f** Interferon (IFN) score (median of relative expression of *IFI27, IFI44L, OASL, IFIT2, IFI6, ALDHA1*) in BJ-hTERT KO for *CGAS* transfected with lipofectamine only (−), WT or D319A inactive cGAS, [each circle represents the mean of a clone, *n* = 4]. **g** The expression level of 444 canonical ISGs was determined in BJ-hTERT lacking *STING1* by RNA-seq and analysed with GSEA; corresponding normalised enrichment scores (NES) and false discovery rate (FDR) are indicated. **h** Bar plot depicting NES of hallmark pathway analysis performed with GSEA in BJ-hTERT *STING1* KO, comparing gene expression in cells KI or KO for *ATRX* to cells WT for *ATRX*. [**a, b, f** Kruskal–Wallis test, two-stage linear step-up procedure of Benjamin, Krieger and Yekutieli, *q* values are indicated, mean ± SEM]. Source data are provided as a Source Data file.

Having demonstrated that ATRX deficiency leads to enhanced interferon signalling dependent on cGAS, we next investigated the activation of the canonical sensing pathway downstream of cGAS, first analysing levels of its product, the second messenger cGAMP^36,37^. Despite sample concentrations falling within the reliable detection range of the ELISA measurements (Fig. S6a), cGAMP levels in primary fibroblasts from patients with ATRX mutations were in the control range (Fig. 4a). In contrast, levels of cGAMP were elevated in fibroblasts from patients with the exemplar cGAS-mediated type I interferonopathies, Aicardi-Goutières syndrome (here, due to mutations in TREX1 and RNASEH2A), and DNase II deficiency (Fig. 4a). While demonstrating the expected reactivity to DNA stimulation, cGAMP levels were also found to be in the WT range in ATRX mutant BJ-hTERT fibroblasts (Figs. 4b and S6b). Correspondingly, we observed no difference in the phosphorylation status of effectors involved in type I interferon induction downstream of cGAS activation by DNA and cGAMP production (and upstream of IFNAR and STAT1 phosphorylation), i.e. STING, TBK1, IRF3 and NF-κB p65, in *CGAS* WT BJ-hTERT clones mutant for ATRX compared to control cells (Fig. 4c). Additionally, because cGAS resides in the nucleus and its detachment from chromatin could trigger DNA sensing, we verified that cGAS remained tightly tethered to chromatin in ATRX deficient cells, similar to control cells (Fig. 4d).

The finding that cGAS is present in the nucleus has raised the possibility of additional roles for cGAS distinct from its DNA sensing function^17,18^. To further establish the non-requirement for cGAS-dependent cGAMP production in mediating the ATRX inflammatory phenotype, we overexpressed WT or catalytically dead (D319A mutant) cGAS in BJ-hTERT fibroblasts null for *CGAS* (Fig. S6c, d). We validated that cGAS D319A was enzymatically inactive, as it failed to produce cGAMP after stimulation with herring testis DNA (Fig. 4e). Nevertheless, we recorded a similar elevation of ISG expression in ATRX mutant cells expressing D319A mutant or WT cGAS (Fig. 4f), demonstrating that the enzymatic activity of cGAS is not required to mediate the interferon response observed in the context of loss of ATRX.

To also explore the role of STING in cGAS-mediated interferon activation induced by ATRX loss of function, we generated BJ-hTERT fibroblasts KO for *STING1* (Fig. S6e) and confirmed that cGAS and MAVS remained functional (Fig. S6f–h). We then engineered clones KI or KO for *ATRX* on a *STING1* KO background and assessed their transcriptional status. Despite the absence of STING, we observed a significant elevation of interferon signalling in ATRX-deficient cells (Figs. 4g and S6i), with the changes in hallmark pathways recapitulating those observed in ATRX mutant cells on a *STING1* WT background (Figs. 2g and 4h). Finally, when we knocked down ATRX in THP-1 cells KO for *IRF3*, a key transcription factor triggered downstream of cGAS-dependent interferon induction after DNA sensing, we observed an interferon response similar to that seen in WT THP-1 cells (Fig. S6j, k).

Overall, these data suggest that, although the induction of interferon in ATRX deficiency involves cGAS, this induction is not mediated through the cGAS-dependent canonical DNA sensing pathway.

### Y1758C ATRX retains its capacity to bind chromatin

**Fig. 5:**
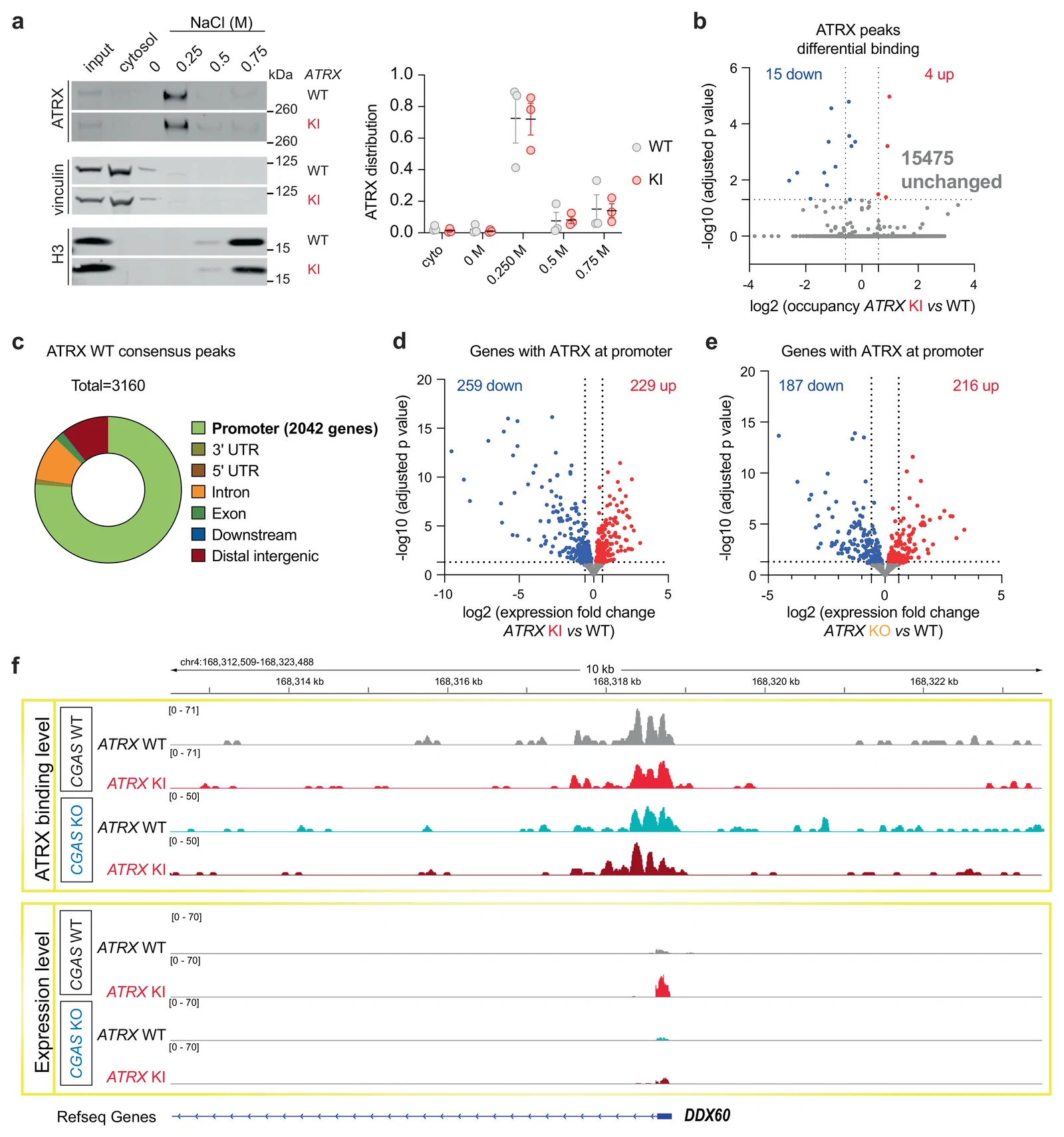
ATRX Y1758C retains its chromatin binding capacity. **a** Subcellular fractionation of BJ-hTERT WT or KI for *ATRX*. Cells were separated into cytosolic and nuclear fractions, and nuclear pellets were subjected to sequential elution with the indicated concentrations of NaCl. Amounts of ATRX and indicated control proteins (vinculin and H3) were assessed by Western blot (left); quantification of the distribution of WT and KI ATRX in the different fractions (right). The band intensities of ATRX in the cytosol and in each of the 0, 0.25, 0.5, 0.75 M salt fractions were summed and set to 1, and the intensity of each fraction was then expressed as a proportion of that sum [three independent experiments, mean ± SEM]. **b** Genome-wide differential binding analysis of ATRX between KI and WT *ATRX* cells; grey, red and blue dots represent peaks that are, respectively, unchanged, enriched or depleted in ATRX KI cells (adjusted *p* value < 0.1). The *Diffbind* tool was employed to compare binding levels between indicated genotypes. **c** Genomic distribution of WT ATRX peaks based on their position in relation to genes. Peaks were defined as shared between two biological replicates. **d, e** Volcano plots depicting differential expression of genes whose promoters are occupied by WT ATRX. Gene expression was determined by RNA-seq, and pairwise differential expression analysis between indicated genotypes (*ATRX* KI (**d**) or KO (**e**) vs WT cells) was performed by *DESeq2*; red and blue dots represent up- and down-regulated genes, respectively (adjusted *p*-value < 0.1). **f** Representative genome browser tracks showing levels of ATRX and RNA at the upregulated gene *DDX60* in BJ-hTERT fibroblasts WT or KI for *ATRX*, and WT or KO for *CGAS*. Source data are provided as a Source Data file.

Considering the role of ATRX as a master genomic regulator^38^, and the finding that transcriptional activation of interferon signalling genes is independent of classical sensing pathways in the context of ATRX deficiency, we next analysed chromatin alterations resulting from a loss of function of ATRX. First, we interrogated the chromatin distribution of KI *versus* WT ATRX. In BJ-hTERT fibroblasts WT or KO for *CGAS*, both WT and KI mutant ATRX were extracted from nuclei at low salt concentration, indicating a similar degree of association to chromatin (Figs. 5a and S7a). We then used Cleavage Under Targets & Tagmentation (CUT&Tag)^39^ to characterize the genomic distribution of ATRX in BJ-hTERT fibroblasts WT and KI for *ATRX*, WT or KO for *CGAS*. This analysis revealed that the Y1758C mutation did not alter ATRX binding to chromatin (with 15,475 of 15,494 individual peaks unchanged) (Fig. 5b), and that cGAS was not involved in determining the localisation of ATRX on chromatin (Fig. S7b). Focusing next on ATRX-bound regions shared between biological replicates, we found that a large fraction of WT ATRX occupied promoters (TSS < 3 kb), localising near 2042 genes (Fig. 5c). Notably, analysis of our RNA-seq data showed that the expression of a subset of between 20 and 24% of these ATRX-associated genes exhibited a statistically significant down or upregulation in *ATRX* KI or KO cells, as compared to *ATRX* WT cells (Fig. 5d, e).

Overall, these data show that, while the Y1758C mutation in ATRX does not affect its binding to chromatin, this mutation does result in changes in the transcription of genes in the vicinity of ATRX-bound sites (Fig. 5f).

### Loss of function of ATRX alters the nucleosomal landscape at genes in a cGAS-dependent manner

**Fig. 6:**
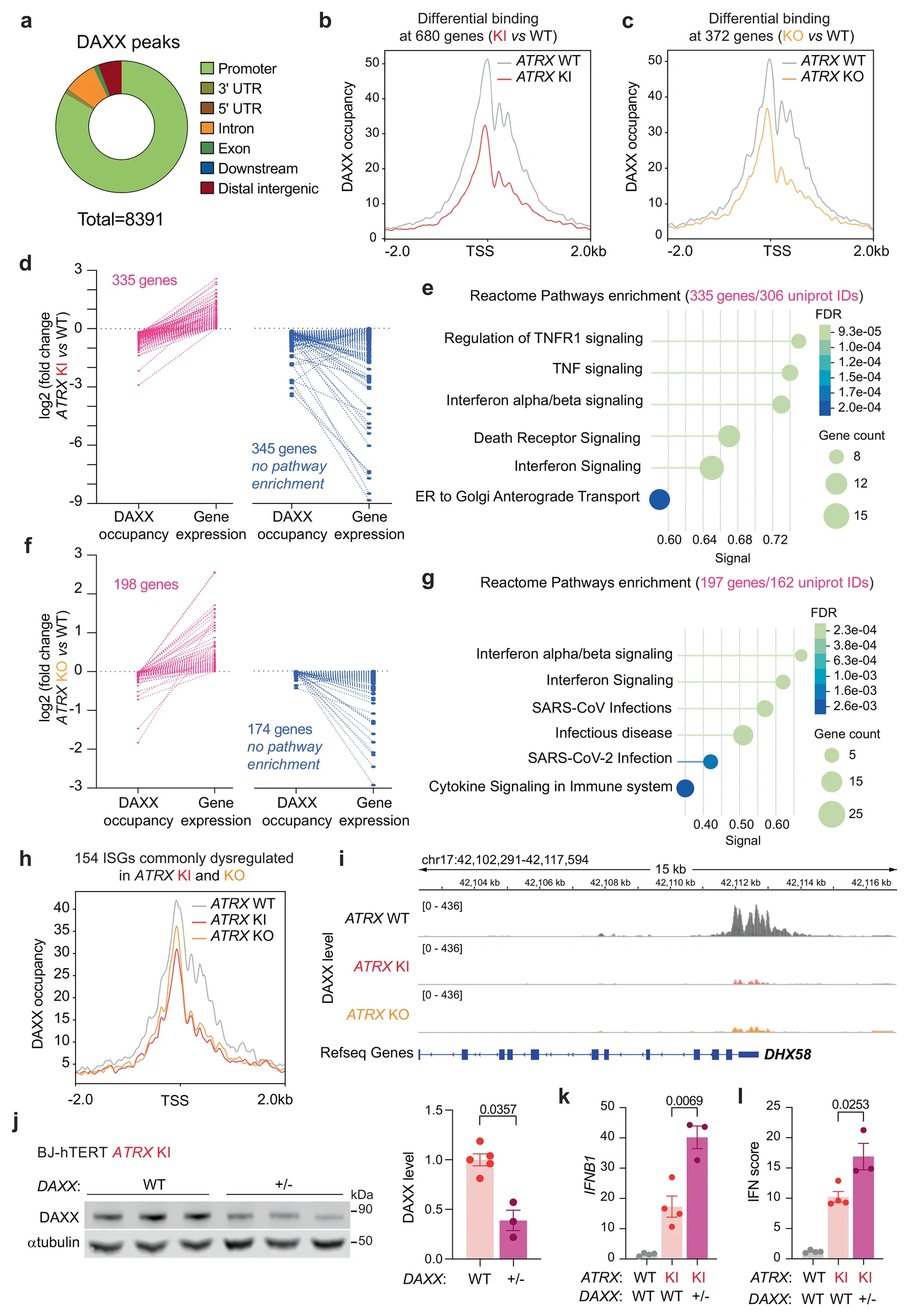
Loss of ATRX function provokes DAXX depletion at interferon stimulated genes. **a** Genomic distribution of DAXX consensus peaks in BJ-hTERT WT for *ATRX*, based on their position in relation to genes. Peaks were defined as shared between two biological replicates. DAXX occupancy was assessed by CUT&Tag at ±2 kb of transcription starting sites (TSS) of genes showing differential binding of DAXX at their promoters in BJ-hTERT KI **(b)** or KO (**c**) for *ATRX*, as compared to WT. Plots showing changes in expression levels of genes exhibiting a significant (adjusted *p*-value < 0.1) decrease in DAXX levels at promoters in *ATRX* KI **(d)** or KO **(f)** cells, as compared to WT. Gene expression was determined by RNA-seq, and pairwise differential expression analysis between indicated genotypes was performed by *DESeq2*. Dot plots depicting Reactome pathway enrichment results obtained by using the STRING database on protein-coding genes exhibiting decreased DAXX at promoters and higher gene expression in BJ-hTERT KI (**e**) or KO (**g**) for *ATRX*, as compared to WT. Gene lists are provided in the Source Data File. **h** DAXX occupancy assessed by CUT&Tag at ±2 kb of the TSS of the 154 ISGs dysregulated in BJ-hTERT KI and KO for *ATRX*, as compared to WT. **i** Representative genome browser tracks showing levels of DAXX at the ISG *STAT1*. **j** Western blot (left) and total protein quantification (right) of DAXX in BJ-hTERT KI for *ATRX* and WT or heterozygous KO (±) for *DAXX*, [circles represent individual clones, *DAXX* WT: clones 1–5, *n* = 1; DAXX+/-: clones 1–3, *n* = 5; Mann–Whitney test, *p* values indicated on graph, mean ± SEM]. Relative expression of *IFNB1* **(k)** and interferon (IFN) score **(l)** (median of relative expression of *IFI27, IFI6, STAT1, IFIT2, IRF7, IRF9, IFIH1, MX1*) measured by qPCR in BJ-hTERT WT or KI for *ATRX*, and WT or hetero-zygous KO for *DAXX*, [each circle represents the mean of an individual clone across independent replicates, *n* = 3; Kruskal–Wallis test, two-stage linear step-up procedure of Benjamin, Krieger and Yekutieli, *q* values are indicated, mean ± SEM]. Source data are provided as a Source Data file.

**Fig. 7:**
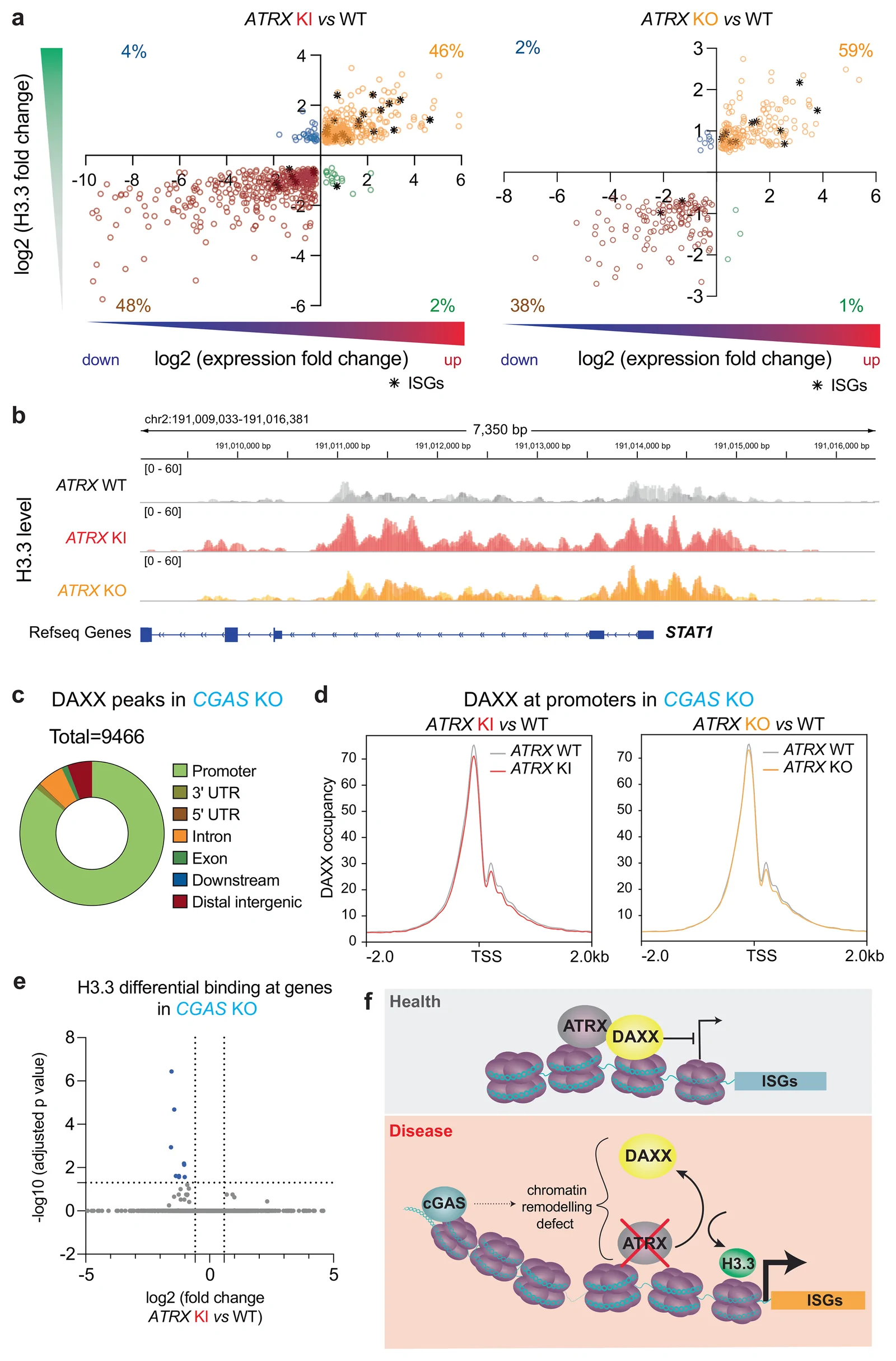
cGAS is required for chromatin landscape changes resulting from ATRX deficiency. **a** Dot plots of genes exhibiting significant (adjusted *p*-value < 0.1) changes in both transcription and H3.3 levels in *ATRX* KI (right) or KO (left), as compared to WT cells. Quadrant colour coding is as follows: blue = genes enriched in H3.3 and downregulated in expression; orange = genes enriched in H3.3 and upregulated in expression; brown = genes depleted of H3.3 and downregulated in expression; green = genes depleted of H3.3 and upregulated in expression; ISGs are denoted separately by a black asterisk, while other genes are represented by empty circles. **b** Representative genome browser tracks showing levels of H3.3 at the ISG *STAT1*. **c** Genomic distribution of DAXX consensus peaks in BJ-hTERT WT for *ATRX* and KO for *CGAS*, based on their position in relation to genes. Peaks were defined as shared between two biological replicates. **d** DAXX occupancy was assessed by CUT&Tag at ±2 kb of transcription start sites (TSS) of genes associated to DAXX in BJ-hTERT KO for *CGAS* and WT, KI or KO for *ATRX*. **e** Comparison of H3.3 occupancy at 17,871 protein-coding genes between BJ-hTERT WT and KI for *ATRX* on a *CGAS* KO background; grey dots indicate genes exhibiting no difference in DAXX or H3.3 occupancy; blue dots indicate genes with significantly altered (adjusted *p*-value < 0.1) H3.3 occupancy in *ATRX* KI cells as compared to WT cells. *Diffbind* tool was employed to compare binding levels between indicated genotypes. **f** In homeostasis (top), ATRX and DAXX bind near the promoters of ISGs to regulate their expression. In ATRX loss of function (bottom), DAXX is depleted, and H3.3 is enriched at the promoters of ISGs. cGAS, which remains normally bound to nucleosomes, mediates these alterations in nucleosomal patterning and sub-sequent changes in gene expression. Source data are provided as a Source Data file.

We hypothesised that altered transcriptional regulation of genes might be associated with changes in the chromatin landscape in cells lacking functional ATRX, particularly focusing on the role of the histone chaperone DAXX and histone variant H3.3 in transcription^40–43^. We employed CUT&Tag to assess the levels of DAXX and H3.3 genome-wide. In control cells, we identified 8391 DAXX binding sites consistent across two biological replicates (Fig. 6a), with 84.5% of these binding sites located at promoters (Fig. 6a). We observed a statistically different pattern of occupancy in cells deficient for ATRX, noting a decrease in DAXX binding at 751 and 394 sites (Fig. S8a, b), associated with promoters of 680 and 372 protein-coding genes, in *ATRX* KI and KO cells respectively (Fig. 6b, c). While the reduced binding of DAXX at promoters showed a variable correlation with gene expression (Fig. 6d, f), genes with both diminished DAXX binding and increased expression were enriched (adjusted *p*-value < 0.1) in interferon and immune signalling pathways according to a STRING Reactome pathways analysis (Fig. 6e, g). Concordantly, levels of DAXX were decreased at the promoters of the dysregulated ISGs recorded in ATRX-deficient cells (Fig. 6h, i). In contrast, genes showing both reduced DAXX levels and reduced gene expression were not characterised by any functional clustering. While EMT-related genes, like ISGs, show increased expression in ATRX-deficient cells, there was no statistically significant change in DAXX occupancy at their promoters (Fig. S8c). As such, these data suggest a specificity relating to the homeostatic regulation of ISG expression by DAXX. To further investigate the link between DAXX and ISG expression, we depleted DAXX in ATRX- deficient cells (Fig. 6j), finding that cells mutated for *ATRX* and heterozygous KO for *DAXX* demonstrated an exaggerated interferon response, as reflected by the enhanced expression of *IFNB1* and ISGs (Fig. 6k, l) (with cells completely deficient for DAXX being non-viable). In contrast to DAXX, we observed a positive correlation between H3.3 levels and gene expression in cells KI or KO for *ATRX* (Figs. 7a, b and S8d, e). Given the cGAS-dependency of transcriptional changes mediated by the loss of function of ATRX, we next explored the behaviour of DAXX and H3.3 in cells KO for *CGAS*. We found that, as in WT cells, DAXX primarily bound to promoters (Fig. 7c). However, unlike in ATRX-deficient cells, where DAXX occupancy at promoters decreased in the presence of cGAS, there was no reduction in ATRX-deficient cells in the absence of cGAS (Figs. 7d and S8e). Genome-wide, H3.3 occupancy was also similar between WT cells and cells mutant for *ATRX* on a *CGAS* KO background (Figs. 7e and S8f).

Collectively, these findings indicate that ATRX deficiency leads to a cGAS-mediated functionally relevant depletion of DAXX at ISG promoters, with an associated enhancement in ISG expression.

## Discussion

In this study, we demonstrate that loss-of-function mutations in the chromatin remodeller ATRX can result in enhanced type I interferon signalling. We show that while this enhanced interferon signalling is cGAS-dependent, it does not involve the canonical pathway downstream of cGAS where the production of cGAMP leads to STING activation. Rather, in the context of a loss of ATRX activity, our study reveals a non-canonical role of cGAS in regulating the genomic occupancy of DAXX and H3.3, and thus gene transcription. Of note, while we observed an expected increase in gammaH2AX foci in ATRX mutant cells (Fig. S9a)^44–46^, the absence of elevated cGAMP in patient cells, and our demonstration of a dependency of interferon signalling on cGAS independent of its enzymatic function, indicates that genome instability does not make a major contribution to the enhanced interferon signalling observed in the ATRX-deficient cells that we studied. Further, we did not detect cytoplasmic telomere foci by telomere-FISH (fluorescence in situ hybridisation (Fig. S9b), arguing against the leakage of telomere fragments into the cytosol activating canonical DNA sensing.

The intracranial calcification seen in P1 and P2 has not been reported previously in ATR-X syndrome. However, there have been previous descriptions of cerebral white matter disease in ATR-X syndrome^22,23^ and recent data implicating ATRX in myelination^47^, raising the possibility that neuroinflammation may be more common in this disorder than is currently appreciated. In support of this, we identified a third patient (P3) with ATR-X syndrome demonstrating systemic and central nervous system inflammation, also associated with enhanced type I interferon signalling, and observed increased ISG expression in fibroblasts derived from two of four other patients with classical ATR-X syndrome tested. Such observed variability between patients may be due to the causative mutation, or to genetic background. Certainly, the latter is the case in regard to the severity of alpha-thalassemia seen in some patients with ATR-X syndrome (quantified by the percentage of red blood cells with haemoglobin H inclusions), where manifestation depends on the length of the G-rich tandem repeat at the alpha-globin locus^29,48^. We hypothesise that variability in the inflammatory phenomenon that we report reflects the recognised stochastic nature of perturbed chromatin accessibility and gene expression^2^. Indeed, the A2137T mutation observed in a patient with systemic and neuroinflammation described here has been recorded twice on the DECIPHER database as a cause of classical ATR-X syndrome in the absence of features of inflammation. More generally, mutations in ATRX are characterised by variable clinical expression, with intellectual disability the only feature shared by all patients in a recent review of more than 200 individuals with ATR-X syndrome^6^. We note also that the breadth of phenotypic spectrum consequent upon germline mutations in ATRX is likely broader than currently appreciated, with the description of osteosarcoma as a feature of ATR-X syndrome only recently recognised^49–52^. Furthermore, the immunogenicity of ATRX deficiency is apparently cell type/state dependent; thus, while *ATRX* null sarcomas demonstrate impaired adaptive and innate immune signalling after ionising radiation^53^, *ATRX* mutations mediate an immunogenic phenotype with macrophage infiltration in neuroblastoma^54^, and upregulation of type I interferon signalling in *Atrx*-null microglia^55^. These observations support the contention that the link between mutations in *ATRX* and engagement of innate immune signalling is not mutation-specific, but reflective of both stochastic and context-dependent phenomena related to ATRX dysfunction.

While demonstrating that the Y1758C ATRX protein is stable and binds normally to chromatin, we find that the cellular consequences of the Y1758C mutation are largely equivalent to a loss of ATRX. Given its location, with the tyrosine at 1758 falling outside the DAXX-binding domain of ATRX (amino acids 1260–1289)^56^, we considered it unlikely that this mutation directly disrupts the interaction between ATRX and DAXX. Rather, we show that the Y1758C substitution impairs the ATPase activity of ATRX and its ability to translocate. These results are in line with data previously generated relating to other ATR-X syndrome mutations associated with retained protein expression^31^. As such, we believe that the Y1758C substitution behaves as a hypomorphic variant consistent with other mutations causative of ATR-X syndrome.

It is known that chromatin remodelling complexes, such as ATRX, can influence the association of transcription regulators with chromatin, thereby redefining the chromatin landscape and gene transcription^57,58^. Here, we show that ATRX deficiency altered nucleosome composition at transcriptionally dysregulated genes. Specifically, we observed reduced levels of DAXX and enrichment of H3.3 at upregulated ISGs. Our findings support the notion that the loss of ATRX and DAXX has a cooperative and/or synergistic effect in activating an interferon signalling transcriptional program, as DAXX depletion enhances the interferon response in ATRX-deficient cells.

In the context of ATRX deficiency, it seems unlikely that DAXX is the chaperone responsible for H3.3 deposition. It is known that H3.3 is present at both actively transcribed genes and constitutive heterochromatin, with its deposition regulated by distinct mechanisms in these different genomic contexts^40^. ATRX/DAXX mediates H3.3 deposition at regions enriched with H3K9me3, whereas the HIRA complex can deposit H3.3 in transcriptionally active genomic regions such as gene bodies and promoters^59,60^. Elucidating the pathways involved in the altered dynamics of DAXX and H3.3 at chromatin, and determining if these changes work alongside the recruitment of STATs/IRFs to boost the expression of inflammatory genes, will be necessary to understand the regulatory mechanisms underpinning the autoinflammation that we report in the context of ATRX deficiency.

In the absence of cGAS, ATRX-deficient cells demonstrate behaviour equivalent to ATRX WT cells, indicating that cGAS plays a central role in the chromatin alterations associated with ATRX deficiency. While an important function of the interaction of cGAS with nucleosomes is to prevent its autoactivation through sequestration away from cellular DNA^61,62^, sequential salt elution showed that cGAS distribution on chromatin remained consistent between WT and ATRX mutant cells (Fig. 4D), suggesting that ATRX does not affect the ability of cGAS to bind to nucleosomes. Rather, our study indicates a non-canonical role for nuclear cGAS in regulating transcription in the context of ATRX deficiency, independent of the activation state of cGAS. This role thus contrasts with our previous observation of disrupted histone stoichiometry leading to cGAS-dependent, cGAMP-mediated enhancement of interferon signalling^63^, or the finding that BAF can outcompete cGAS on DNA^64^, both of which serve to limit the activation of cGAS by cellular DNA. Thus, future research should explore how cGAS contributes to changes in chromatin remodelling consequent upon ATRX deficiency.

In summary, our findings establish a model where the loss of ATRX function affects the chromatin landscape, leading to altered distributions of DAXX and H3.3, and associated changes in gene transcription. In this context, cGAS exerts a non-canonical role in regulating chromatin remodelling mediated by ATRX, a process that occurs independently of the activation state of cGAS (Fig. 7f).

## Methods

### Clinical reports

Clinical information and samples were obtained with informed consent. The study was approved by the Comité de Protection des Personnes (ID-RCB/EUDRACT: 2014-A01017-40) in France, and the Leeds (East) Research Ethics Committee (REC reference: 10/H1307/2; IRAS project ID: 62971) in the UK.

### P1 (family AGS677)

This male was born to non-consanguineous parents of white European ancestry. There was no family history of note. He was delivered at 39 weeks gestation, demonstrating intrauterine growth retardation with a weight of 2.25 kg and head circumference of 34.5 cm. There were significant early problems with feeding, poor weight gain and recurrent upper respiratory tract infections. He walked at 20 months of age, demonstrated a delay in articulation and comprehension, and subsequently exhibited significant learning difficulties leading to placement in a special school.

He required surgical correction of an umbilical hernia at the age of 6 years. He underwent tibial tendon transfer on the left and then on the right at the ages of 8 and 15 years, respectively. Chondromatous lesions of the distal femoral metaphyses, more marked on the left than the right, were identified during this period.

At the age of 14 years, he presented with a single seizure. Cerebral CT showed multiple small round high signal spots of calcification in the cerebellar hemispheres (left > right), middle cerebral peduncle on the left and pons. Supratentorially, larger, dense calcifications were present in the caudate, globus pallidus and putamen, and small dots of calcification were seen in the right frontal and left parietal white matter/grey-white matter junction. There was a left parietal cystic area surrounded by calcium deposits. On T2-weighted cerebral MRI, there was left hemispheric swelling and a high signal white matter lesion which appeared solid, with heterogeneous signal intensity and surrounding contrast enhancement. Biopsy of the left-sided cerebral lesion demonstrated features of ischaemic necrosis, with numerous foci of calcification and a minor lymphocytic infiltrate of the mediumsized vascular walls in the absence of granuloma, tumour or infection.

ISG expression in blood was persistently elevated on each of six occasions assayed between the ages of 14.95 and 23.1 years. Exome sequencing revealed a p.(Tyr1758Cys) (Y1758C, c.5273A>G) substitution in ATRX, which was maternally inherited. An unaffected brother and maternal uncle were found not to carry the variant.

He has experienced persistently poor weight gain, with a weight of 36 kg and height of 164 cm at age of 16 years and 11 months. Investigation for the presence of haemoglobin H (HbH) inclusions was normal.

### P2 (family AGS2351)

This 14-year male is the only child of unrelated white British parents. There is no family history of note beyond a unilateral ptosis and strabismus in his father. He was delivered vaginally at 33 weeks gestation, weighing 1.76 kg following a pregnancy complicated by premature rupture of membranes and poor fetal growth. He required CPAP for 3 days, was fed by nasogastric tube for 4 weeks, received phototherapy for jaundice and was treated for presumed sepsis. Atrial and ventricular septal defects were identified on echocardiography, which closed spontaneously. He was discharged home at 5 weeks of age. At 1 month of age, his weight was 2.28 kg, length 45.5 cm and head circumference 33 cm. Mild developmental delay became evident during the first year of life, and he was noted to have mild brachycephaly with a tall forehead, a short nose, a tented upper lip and high arched palate. Between 18 months and 3 years of age, he experienced three generalised febrile seizures.

At the age of 3 years, his parents observed a persistent absent red reflex on the left side. He was subsequently diagnosed with bilateral asymmetric retinal vascular disease, more marked on the left, leading to recurrent vitreous haemorrhages, a vitrectomy, retinal detachment and loss of vision in the left eye.

MRI at the age of 5 years revealed numerous peripheral enhancing lesions with haemorrhagic foci in the cerebrum, putamen and left thalamus, considered most likely to represent small vessel vasculitis. A CT scan showed extensive calcification. At the age of 7 years, he presented with headaches, and further imaging revealed progressive suspected inflammatory changes. He developed a left hemiparesis, prompting treatment with prednisolone and mycophenolate mofetil. At the age of 8 years, he received intravenous and intramuscular fetal stem cell therapy outside of the UK.

At 8 years of age, he presented with symptomatic hypoalbuminemia, considered to have been precipitated by a bout of gastroenteritis. This was initially managed with a high protein diet, alternate day prednisolone, omeprazole, penicillin prophylaxis and albumin infusions. Investigations confirmed a protein-losing enteropathy, with diffuse thickening and signal enhancement of the small bowel wall, several small gastric varices, and diffuse low attenuation of the liver on ultrasound suggestive of fatty infiltration in the absence of portal hypertension. He has continued to experience intermittent swelling of legs and genitals.

He has experienced two fractures requiring surgery: a left transverse distal shaft femur fracture following a fall on a trampoline at the age of 6 years, and a fractured left neck of femur at 7 years of age. Following surgical treatment with metal plates, he developed chronic osteomyelitis necessitating prolonged antibiotic treatment.

Investigations have included a normal male karyotype and normal testing of the following genes: *DADA2, CTC1, STN1, SNORD118*, and the following two panels: ‘Inflammatory disorders’ and ‘interferonopathy-related genes’. Exome sequencing revealed no other putative mutations beyond the Y1758C substitution in ATRX. Autoantibody screen, cardiolipin, glycoprotein 1, Beta-2-glycoprotein antibodies, rheumatoid factor, double-stranded DNA, C4, C4 normal IgG and IgM, transferrin, electrolytes, liver function tests, CK, thyroid function, and full blood count were all essentially normal.

ISG expression in blood was persistently elevated on each of seven occasions assayed between the ages of 7.1 and 10.9 years. Exome and confirmatory Sanger sequencing identified a p.(Tyr1758Cys) (Y1758C, c.5273A>G) substitution in ATRX, which was maternally inherited.

Now aged 14 years, he demonstrates autistic traits and moderate learning difficulties. His facial features are also consistent with ATR-X syndrome. HbH inclusions were not detected on two occasions.

### P3 (family AGS4114)

P3 is a boy aged 2 years 3 months, the second child born to non-consanguineous parents of white European ancestry. He was delivered at 38 + 6 weeks gestation after an uneventful pregnancy with a birth weight of 2.46 kg. Early postnatal assessment revealed microcephaly, mild axial hypotonia, and hip dysplasia, which was managed with a Pavlik harness during the first year of life. Feeding difficulties, including frequent vomiting, were evident from birth, ultimately necessitating nasogastric tube feeding. He was diagnosed with a hiatus hernia and gastro-oesophageal reflux and is currently being evaluated for a gastrostomy. At the age of 18 months, his head circumference was −3.2 SD. He demonstrated age-appropriate social responsiveness; however, language development was delayed, with no spoken words. His gross motor development was also delayed: he could roll and sit only with support but had intact fine motor abilities. He was unable to pull to stand and had not achieved independent walking. Recurrent viral lower respiratory infections were noted from approximately 10 months of age, with one admission requiring high-flow oxygen support. No invasive or atypical infections were reported.

From early infancy, progressive erythematous to violaceous subcutaneous nodules and indurated plaques were observed on the extremities, gradually progressing to the face and trunk, consistent with inflammation of the subcutaneous fat. Lesions were non-ulcerative with soft tissue oedema, resulting in localised swelling of the affected regions. By the age of 18 months, he developed prolonged spiking fevers (>4 weeks) associated with exacerbation of skin lesions and mild splenomegaly, for which he was transferred to tertiary care. He was noted to have a mildly coarse facial appearance with bilateral epicanthal folds, upturned nasal tip and a broad mouth with a tented upper lip. Laboratory evaluation demonstrated normocytic anaemia (5.5 mmol/L), only modestly elevated systemic inflammatory markers (ESR 33 mm/h, CRP 14 mg/L), hepatitis with elevated transaminases (ALAT 473 U/L, ASAT 686 U/L, γGT 74 U/L), and proteinuria with preserved renal function (protein-to-creatinine ratio 115 mg/mmol). No auto-immune hepatitis associated antibodies were present. Immunologic studies revealed a hyperinflammatory cytokine profile with markedly elevated ferritin (904 µg/L), galectin-9 (42085 pg/mL; <5073 pg/mL), CXCL10 (4584 pg/mL; <805 pg/mL), IL-18 (6125 pg/mL; <175 pg/mL), and soluble IL-2 receptor levels above the detection range of 300 U/mL. A type I interferon RNA signature was strongly elevated at presentation (18.52 A.U.; <9). Flow cytometry showed low CD8 T-cell counts and a predominance of naïve B cells with marked reduction of memory B cells. Imaging revealed mild splenomegaly and coarse hepatic echotexture on abdominal ultrasound, while high-resolution CT of the chest was normal. Brain MRI demonstrated mildly enlarged ventricles and a thin corpus callosum within normal variation and no calcifications seen on SWI. Echocardiography was unremarkable. Lumbar puncture showed normal opening pressure, with CSF neopterin elevated during active disease (298 nmol/L; <40 nmol/L). Histopathology of the skin demonstrated lobular panniculitis with lymphocytic rimming and abundant histiocytes, without loss of T-cell markers with negative microbial stains. Molecular studies detected clonal TRD and TRG-VJ rearrangements, and immunohistochemistry confirmed an α/β-positive, γ/δ-negative T-cell phenotype, supporting the final diagnosis of a subcutaneous panniculitis-like T-cell lymphoma. Notably, distinction from severe inflammatory panniculitides remains challenging, as overlapping histopathologic features and limited clonality can obscure the boundary between reactive and neoplastic processes.

Genetic investigations, including trio whole-genome sequencing with targeted analysis for developmental delay and immune disorders, an exome-wide assessment and genome-wide copy-number variant analysis, revealed a maternally inherited hemizygous VUS in *ATRX* (c.6409G>A p.(Ala2137Thr)). This variant has been reported twice in the DECIPHER database (Patient 281787: https://www.deciphergenomics.org/patient/281787/genotype/205426/browser; Patient 531448: https://www.deciphergenomics.org/patient/531448/genotype/309651/browser), and diagnostic methylation studies indicated pathogenicity of the *ATRX* substitution, establishing the diagnosis of ATR-X syndrome. Apart from the variant in *ATRX*, genetic investigations were negative.

While the patient did not meet HLH-2004 criteria, the child’s hyperinflammatory state led to treatment with intravenous methyl-prednisolone 15 mg/kg/day for 3 days, resulting in rapid resolution of fever, improvement of cutaneous lesions, and normalisation of liver enzymes. Ruxolitinib 2.5 mg twice daily was initiated after 1 month as corticosteroid-sparing therapy. Oral maintenance prednisolone 1 mg/kg/d was continued followed by a gradual taper and stop after 3 months. MRI whole body under low-dose prednisone performed after 3 months of therapy showed near-complete remission with only minor residual cutaneous abnormalities. Proteinuria, anaemia and elevated neopterin in CSF (16 nmol/L) resolved with corticosteroid therapy. All pro-inflammatory cytokines and markers improved under prednisone, including the type I interferon signature (albeit remaining elevated at 4.34; <2.758), and pro-inflammatory cytokines all normalised within 1 month of starting ruxolitinib. The patient showed improvement in his motor development and learned to crawl and pull himself to standing. Within weeks of stopping prednisone a moderate elevation in liver enzymes was noted in the absence of a clinical recurrence of skin lesions, type I interferon signature had normalised (6.04 A.U. (<9.4)), and ultrasound imaging of the skin and abdomen were normal. However, MRI whole body performed routinely 8 months after initiation of treatment showed recurrence of skin lesions at multiple sites. The patient is otherwise well, has no fever and no upregulation of pro-inflammatory cytokines except a mild increase in soluble IL-2 receptor (80 U/mL; <44 U/mL). Skin biopsy results are currently awaited, with consideration being given to increasing the dose of ruxolitinib. The patient continues multidisciplinary outpatient follow-up with rheumatology/immunology, oncology, gastroenterology, dermatology, and rehabilitation services.

### Genetic studies

DNA was extracted from whole blood using standard methods. Exome sequencing was performed on genomic DNA from P1 and P2, and the parents of patient 1, using a SureSelect Human All Exon kit (Agilent Technologies) for targeted enrichment, and Illumina HiSeq2000 for sequencing. The variant c.5273A>G/p.Tyr1758Cys in exon 21 of ATRX was assessed using the in silico programs SIFT and Polyphen2 (http://genetics.bwh.harvard.edu/pph2/). Confirmatory Sanger sequencing was undertaken in both patients and used to assess the genetic status of other family members. The reference sequence used for primer design and nucleotide numbering was NM_000489.3. Primers for Sanger sequencing of ATRX exon 21: ATRX Ex21F tgtgtttttgacatgagcatttca, ATRX Ex21R tgaggctttacacctggcaa, generating a PCR product of 506 bp. The p.(Ala2137Thr) (A2137T, c.6409G>A) recorded in P3 was identified on diagnostic trio genome sequencing performed at University Medical Center Groningen, The Netherlands. Methylation studies (episignature) in P1 and P3 were performed, respectively, at the West Midlands Regional Genetics Laboratory, UK, and the Amsterdam University Medical Center, The Netherlands. Population allele frequencies were obtained from the gnomAD database (http://gnomad.broadinstitute.org).

### Yeast strain generation

A *Saccharomyces cerevisiae* optimised codon sequence was designed to express human cDNA of ATRX (2492 amino acids) and synthesised by GeneArt (Thermo Scientific); ATRX was C-terminally fused to 2xFLAG (DYKDDDGDYKDDD) and inserted into a pTDK8 plasmid using Xma1 and Spe1 restriction sites. The full sequence of the plasmid (13,889 bp) is provided in the Supplementary Information File. Mutant variants of the plasmid (K1600R and Y1758C) were generated using site directed mutagenesis (NEB Q5® Site-Directed Mutagenesis Kit E0554S) and the following primers: Y1758C_fwd: CTTGATCGAAtgtCACTGTATGG, Y1758C_rev: TTGTTTTGCAATGGAGTAC, K1600R_fwd: GGGTTTGGGTagaACATTGCAAG, K1600R_rev: ATGCAATGAGCCAAAATAC. Manufacturer’s instructions were followed aside from the use of Stable Competent *E. coli* (High Efficiency) (NEB #C3040H). Following DNA sequence verification by whole plasmid sequencing, plasmids were linearised and integrated into yJF1 budding yeast strain, resulting in yJF1-ATRX-WT (ATRX-2XFLAG-WT), yJF1-ATRX-K1600R (ATRX-2XFLAG-K1600R) and yJF1-ATRX-Y1758C strains (ATRX-2XFLAG-Y1758C). Generated strains are summarised in the following Table 1.

### Purification of recombinant ATRX from budding yeast

Nine litres of appropriate yeast strains (yJF1-ATRX-WT, yJF1-ATRX-K1600R, yJF1-ATRX-Y1758C) were grown in YP-raffinose (2%) at 30 °C and allowed to reach 2 × 10^7^ cell/mL. Induction of ATRX expression was achieved by addition of 2% galactose followed by 3 h incubation at 30 °C. Cells were harvested (Beckman Coulter J-Lite JLA-9.1000 rotor 5000 rpm/5185 × *g*, 10 min, 4 °C) and pellets were washed twice in ice-cold buffer 1 (25 mM HEPES KOH, pH 7.6, 10% glycerol, 0.5 M NaCl, 0.1% NP-40 alternative). The resulting pellet was weighed and resuspended in 0.4 volumes of buffer 1 (supplemented with 1 mM DTT and protease inhibitors (Roche, 5056489001 and Sigma-Aldrich, P8215), and the cell suspension frozen in liquid nitrogen drop by drop using a stripette. Frozen droplets were pulverised in a freezer mill (SPEX™ Freezer/Mill™ High Capacity Cryogenic Grinder) for three cycles (15 c.p.s., 2 min/cycle with 2 min rest between cycles). The resulting powder was thawed at room temperature and resuspended in an appropriate volume of buffer 1 supplemented with 1 mM DTT and protease inhibitors to fill Beckman Coulter Type 45 Ti Fixed-Angle Titanium Rotor tubes and centrifuged at 45,000 rpm/235,000 × *g*, at 4 °C for 1 h. Supernatant was added to 1 mL of pre-washed ANTI-FLAG® M2 Affinity Gel (Millipore A2220) and incubated at 4 °C for 1 h with rotation. This step was repeated for a total of 2 mL of ANTI-FLAG® M2 Affinity Gel. The resin recovered was washed with 100 mL buffer 1 supplemented with 1 mM DTT and protease inhibitors, followed by a 10-min incubation with buffer 1 supplemented with 1 mM DTT and protease inhibitors + 1 mM ATP + 5 mM MgOAc on ice, followed by another 40 mL buffer 1 with 1 mM DTT and protease inhibitor wash. ATRX was eluted sequentially by incubating resin with 2× resin volumes (RV) of buffer 1/0.5 mg/mL 3XFLAG peptide (Sigma-Aldrich F4799) for 15 min and 2× resin volumes (RV) buffer 1 supplemented with 0.25 mg/mL 3XFLAG peptide for another 15 min. ATRX eluate was concentrated using Amicon® Ultra Centrifugal Filter 50 kDa MWCO (Millipore UFC9050) at 4000 × *g*, 16 min at 4 °C and further purified by size exclusion chromatography (Sigma-Aldrich Superdex® 200). Peak fractions were pooled and concentrated using Amicon® Ultra Centrifugal Filter 50 kDa MWCO (Millipore UFC9050) at 5000 rpm 4 °C. Protein concentration was determined using Bradford assay, and the protein aliquoted, flash frozen in liquid nitrogen and stored at −70 °C freezer. SDS-page (NuPAGE Tris-Acetate mini protein gels, Invitrogen) was stained with InstantBlue Coomassie protein stain (ISB1L) (Abcam, AB119211).

### ATPase assay

The protocol was adapted from Dürr and Hopfner 2006^65^ using BIOMOL® Green for detection of liberated phosphates (Enzo Life Sciences BML-AK111). Reactions were set up on ice with 20 nM recombinant ATRX (WT, K1600R, Y1758C) or no protein and excess plasmid DNA in a buffer containing 50 mM HEPES KOH pH 7.6, 5% glycerol, 50 mM NaCl, 10 mM MgCl_2_, 10 mM KCl, 1 mM DTT, 2 mM ATP. Reactions were incubated in a thermocycler for 1 h at 30 °C and stopped by the addition of BIOMOL® Green (90 μL of reagent to 10 μL of each reaction, transferred to a 96-well plate, mixed briefly by gentle vortexing and incubated for 30 min at room temperature, protected from light, before determining the absorbance at 620 nm using a Multiskan SkyHigh Microplate Spectrophotometer (Thermo Scientific). A fresh standard curve was generated using phosphate standard (Enzo Life Sciences BML-KI102) each time and used to determine the amount of phosphate released in the samples. Each standard and sample were run in technical duplicates.

### Triple helix displacement assay

Protocol for the Triple helix displacement assay was adapted from previously published protocols^31,65,66^. Plasmid with a binding site for triple helix forming oligo (TFO) was synthesised by GeneArt in a pMA-T backbone. The full sequence of the plasmid is provided in the Supplementary Information File. For generation of the triple helix, 60 nM of this plasmid was linearised with PvuI and mixed with 2-fold excess 5′ Cy5-TFO (5′Cy5/TTCTTTTCTTTCTTCTTTCTTT) in annealing buffer (25 mM MES solution Sigma-Aldrich 76039, 10 mM MgCl2, 50 mM NaCl). The annealing reaction was incubated in the thermocycler at 60 °C for 15 min, followed by gradual cooling (99 cycles, −0.5 °C/30 s), and the excess TFO removed using Monarch® Spin PCR & DNA Cleanup Kit (NEB 1130S) and eluted in annealing buffer. For the displacement assay, reactions were started by the addition of recombinant ATRX protein to reaction buffer containing 50 mM HEPES KOH pH 7.6, 5% glycerol, 50 mM NaCl, 10 mM MgCl_2_, 10 mM KCl, 1 mM DTT, 20 mg/ml BSA, 2 mM ATP and 6 nM triple helix. Reactions were incubated for 30 min at 30 °C, except for the heat denatured control at 80 °C, and stopped by the addition of gel loading buffer (6 × 15% Ficoll-400, 19.8 mM Tris-HCl pH 8, 0.48% SDS). Reactions were resolved in a 1.25% agarose gel (40 mM Tris-acetate pH 5.5, 5 mM Na-acetate, 1 mM MgCl_2_), at 5 V/cm for 35 min at 4 °C and imaged using 635 nm Laser-Cy5 FLA-5100 Fluorescent Image Analyser (FujiFilm). Resulting images were quantified using FIJI. Area adjusted background intensity was subtracted from bands, and the percentage of displaced TFO calculated according to the formula: % displaced TFO = 100 × TFO/(TFO + Triple helix). Results were then normalised to a heat denatured control samples that were pooled and set to 100%.

### RT-qPCR quantification and interferon stimulated gene (ISG) gene expression

Whole blood was collected into PAXgene tubes (Qiagen) and total RNA extracted using a PreAnalytix RNA isolation kit. Interferon (IFN) scores were generated as previously described^67^.

For NanoString ISG analysis, total RNA was similarly extracted from whole blood with a PAXgene (PreAnalytix) RNA isolation kit. Analysis of 24 ISGs and 3 housekeeping genes (probes of interest [*n* = 24]: *IFI27, IFI44L, IFIT1, ISG15, RSAD2, SIGLEC1, CMPK2, DDX60, EPSTI1, FBXO39, HERC5, HES4, IFI44, IFI6, IFIH1, IRF7, LAMP3, LY6E, MX1, NRIR, OAS1, OASL, OTOF*, and *SPATS2L*; reference probes [*n* = 3]: *NRDC, OTUD5*, and *TUBB*) was conducted using the NanoString customer designed CodeSets according to the manufacturer’s recommendations (NanoString Technologies). Data were processed with nSolver software (NanoString Technologies). The data were normalised relative to the internal positive and negative calibrators, the three reference probes, and the control samples. Scores above 2.75 are designated as positive.

In cell lines, total RNA was extracted using the RNeasy Micro Kit (Qiagen, 74004), and reverse transcription was performed with the High-Capacity cDNA Reverse Transcription Kit or SuperScript™ IV VILO™ Master Mix (ThermoFisher Scientific). Levels of cDNA were quantified by RT-qPCR using TaqMan Gene Expression Assays (ThermoFisher Scientific), normalised to *HPRT1* (Hs03929096), *ACTB* (Hs01060665_g1), *VCL* (Hs00419715_m1) and *TBP* (Hs00427620_m1). Relative quantitation of target cDNA was determined using the formula 2^−ΔΔCT^, with ΔΔCT representing the fold increase above control. Interferon (IFN) scores were calculated as the median of the fold change expression of selected ISGs: *IFI27* (Hs01086370_m1), *RSAD2* (Hs01057264_m1), *MX1* (Hs00895608_m1), *ISG15* (Hs00192713_m1), *IFI44L* (Hs01060665_g1), *IFIT1* (Hs00356631_g1), (Hs00234713_m1), *STAT1* (Hs01013996_m1), (Hs00946916_m1), *IFI6* (Hs00242571_m1), *IFIH1* (Hs00223420_m1), *OAS1* (Hs00973637_m1). Other probes used in this study were: *IFNB1* (Hs01077958_s1) and *MAVS* (Hs00920075).

### Cell culture

Fibroblasts and 293FT (Invitrogen, R70007) cells were grown in DMEM-Glutamax supplemented with 10% FBS and 1% P/S (10,000 U/mL). Primary fibroblasts from P1 and P2 were generated in this paper; P3 to P6 are part of the Gibbons laboratory cell bank, P3: c.666G>T/p.(W222C); P4: c.797A>G/p.(Y266C); P5: c.5039T>C/p(.I1680T); P6: c.413_414delAA/p.(K138fs); controls were purchased from Gibco and Innoprot; patient TREX1 (AGS261.1): p.(Glu20Glyfs*82)/p.(Ala81-Serfs*21); patient DNASEII (AGS85.1): homozygous p.(Gly116Ala) and patient RNASEH2A (AGS070.1): p.(Arg235Gln)/p.(Arg25Arg) are part of the Crow laboratory cell bank. BJ-hTERT WT and null for cGAS were obtained from the Chen laboratory (University of Texas, Dallas)^35^; THP-1 and *IRF3* KO THP-1 cells were from ATCC (TIB-202) and InvivoGen, respectively.

Fibroblast stimulations were conducted by treating cells for 4 h with 1 µg/mL herring testes (HT)-DNA (D6898; Sigma-Aldrich) complexed with Lipofectamine 2000 (Invitrogen) according to the manufacturer’s instructions, with 1 µM diABZI (Cayman, 280541), or with 100 ng/mL Poly (I:C) (Invitrogen, cat nb tlrl-piclv, (HMW) / LyoVec™) for 6 h.

### ISG reporter HEK 293 cells

Interferon regulatory factor (IRF)-Inducible SEAP Reporter HEK293 (HEK-Blue™ ISG) cells (InvivoGen, hkb-isg-1) were used as an interferon activity reporter. Fifty thousand HEK-Blue™ ISG cells were seeded per well of a 96-well-plate and stimulated with 180 μL of supernatant from BJ-hTERT fibroblasts in culture for 2–3 days, or from THP-1 cells transduced with sh-RNAs targeting *ATRX* or control sh-RNAs, and collected at day 8 post-transduction. Cells were stimulated for 24 h in a final volume of 200 μL. Two wells were tested per experimental condition. Culture supernatants were collected, and levels of embryonic alkaline phosphatase (SEAP) secreted by HEK-Blue™ ISG cells were determined with QUANTI-Blue™ Solution (turning purple/blue in the presence of SEAP), calculated from the OD at 650 nm read using a microplate reader. Readings were normalised to controls cells.

### Induced pluripotent stem cell (iPSC) culture and neural differentiation

The iPSC QOLG-1 cell line was obtained from Kathy Evans (Centre for Genomic and Experimental Medicine, Institute of Genetics and Cancer, Edinburgh, UK), acknowledging the Wellcome Trust Sanger Institute as the source of the HipSci HPSI1113i-qolg_1 human induced pluripotent cell line (ECACC 77650145). iPSCs were made using a CytoTune iPS Reprogramming kit obtained from Life Technologies by Sanger as the ‘the buyer’, under a Limited Use Label Licence. iPSCs were maintained on vitronectin (Stem Cell Technologies, 7180), with a complete media change every day in Essential 8 media (Life Technologies, A1517001). Zero point five millimolar EDTA in PBS was used for passaging. Accutase (Stem Cell Technologies 07920) and 10 μM Rho-associated kinase (ROCK) inhibitor (Sigma-Aldrich Y0503) were used for single-cell dissociation purposes. iPSCs were collected at passage 38–40.

Neural progenitor cells (NPCs) were established following the STEMdiff Neural System based on STEMdiff SMADi Neural Induction kit (Stem Cell Technologies, 08581). Briefly, embryoid bodies (EBs) were generated using AggreWell™800 plates, with each EB containing approximately 10,000 cells. EBs were fed daily for 4 days with STEMdiff Neural Induction Media + SMADi. At day 5, EBs were replated on Matrigel coated dishes and fed daily for 6 days with STEMdiff Neural Induction Media + SMADi. Neural Rosettes were dislodged from EBs using STEMdiff Neural Rosette Selection solution (Stem Cell Technologies, 05832), replated on Matrigel coated dishes, and fed daily with STEMdiff™ Neural Induction Media + SMADi. When cells reached confluency, they were split in two using Accutase, and then replated (passage 1). From passage one onwards, NPCs were fed daily with STEMdiff Neural Progenitor Media (Stem Cell Technologies, 05833).

NPCs were used from passage two to generate forebrain neurons following the instructions of STEMdiff Forebrain Neuron Differentiation Kit (Stem Cell Technologies, 08600) and STEMdiff Forebrain Neuron Maturation Kit (Stem Cell Technologies, 08605). Briefly, NPCs were plated on Poly-L-Ornithine/Laminin (Sigma L2020, P4957) coated dishes and fed daily with STEMdiff forebrain differentiation media for 6 days. Generated neural precursors were replated on Poly-L-Ornithine/Laminin coated dishes, fed with STEMdiff forebrain neuron maturation kit twice a week, and cultured for 3–4 weeks.

### Knock-down and over-expression of genes

Lentiviral constructs containing shRNA targeting *ATRX* (pLKO.1_shATRXn1: TRCN0000342811, pLKO.1_shATRXn2: TRCN0000013590, pLKO.1_shATRXn3: TRCN0000352788) were from Sigma-Aldrich. pLKO.1 empty vector (sh-control1) was from Open Biosystems, and pLKO.1 control shRNAs (scramble shSCR, SHC002 (sh-control2, and non-target shNTgt, SHC016, sh-control3) were from Sigma-Aldrich. Lentiviral particles were produced by Lipofectamine 2000 transfection of 293FT cells with shRNA constructs, psPAX2, and pCMV-VSV-G. Supernatant containing viral particles was used to infect THP-1 cells. Two days after transduction, cells were cultured with media containing 1.5 µg/mL puromycin (Sigma-Aldrich). Cells were collected for analysis 5 and 8 days after transduction.

Expression of *MAVS* was downregulated by using 20 nM siRNAs targeting *MAVS* (LifeTechnologies #s41746, #148763) or negative control#1 (LifeTechnologies, Silencer Select, Cat#4390843). All were delivered to cells using Lipofectamine RNAiMAX (Invitrogen) according to the manufacturer’s instructions. Cells were analysed 72 h after transfection.

For cGAS WT and D319A overexpression in BJ-hTERT fibroblasts, transfection was performed by using Lipofectamine LTX. Briefly, 250,000 cells were seeded in 1 well of a 6-well plate; 1:1 DNA/supplement ratio was used, and 0.82 μg of WT or 1.2 μg of D319A cGAS plasmids were complexed to 2.46 or 3.6 μL of Lipofectamine, respectively. Complexes were added on cells and incubated for 4 h. Supernatants were then removed and replaced with full growth media. Cells were collected for analysis 3 days after transfection. The plasmids used were pEFBOS-cGAS-WT-Flag-His and pEFBOS-cGAS-D319A and were kindly gifted by the laboratory of Andrea Ablasser (EPFL, Lausanne, Switzerland).

### Western blot

Proteins from whole-cell lysates were extracted using RIPA lysis buffer (25 mM Tris-HCl pH 8.0, 1% IGEPAL 60, 150 mM NaCl, 1.5 mM MgCl_2_, 0.05% SDS, 0.5% sodium deoxycholate, Benzonase (Sigma-Aldrich, E1014) 750 U/mL) supplemented with protease and phosphatase inhibitor tablets (ROCHE). Reducing Buffer was added to proteins and heated for5 min at 95 °C. Samples were resolved on 3–8% Tris-acetate or 4–12% Bis-Tris and then transferred to a nitrocellulose membrane using the iBlot 2 Dry Blotting System (LifeTechnologies). Membranes were blocked with Intercept (TBS) buffer (LI-COR, P/N: 927-60001) and primary antibodies incubated overnight at 4 °C in the blocking buffer. Membranes were washed and incubated with IRDye secondary antibodies (LI-COR, 1/5000) for 45 min at room temperature. Signal was detected using the Odyssey CLx System (LI-COR). Quantifications were performed using Image Studio 5.2. Primary antibodies and dilution used were: ATRX (NBP2-50111, 1/500); Ser172-TBK1 (CST 5483, 1/500), Tyr701-Stat1 (CST 7649, 1/700); STAT1 (CST 9176, 1/1000); Ser396-IRF3 (CST 4947, 1/700); vinculin (CST 13901, 1/1000), S366-STING (CST 19781, 1/1000), STING (Millipore MAB7169, 1/1000), NF-κB p65 (CST 6956, 1/1000), Phospho-NF-κB p65 (Ser536) (CST 3031, 1/1000), cGAS (CST 15102, 1/500), α-tubulin (Sigma-Aldrich T6074, 1/5000), Histone H3 (CST 4499, 1/5000), Histone H2B (CST 12364, 1/2000).

### Subcellular fractionation

For subcellular salt extraction fractionation, we followed a published protocol with minor changes^61^. Cells were pelleted, washed in PBS, resuspended in extraction buffer (10 mM Hepes pH 7.9, 10 mM KCl, 1.5 mM MgCl_2_, 0.34 M sucrose, 10% glycerol, 0.2% IGEPAL 60), and incubated on ice for 10 min with occasional vortexing. Nuclei were spun at 3500 × *g* for 5 min at 4 °C. The cytosolic fraction (supernatant) was collected for further analysis. Nuclei were then washed for 1 min on ice in extraction buffer without IGEPAL 60 and spun at 3500 × *g* for 5 min at 4 °C. Pelleted nuclei were resuspended in zero salt buffer (3 mM EDTA, 0.2 mM EGTA), vortexed 10 s on/10 s off for 1 min, and incubated on ice for 30 min, vortexing for 15 s every 15 min. Lysates were spun at 6500 × *g* for 5 min at 4 °C. The zero-salt supernatant was collected for further analysis. The remaining pellets were then resuspended in first salt buffer (50 mM Tris-HCl, pH 8.0, 0.05% IGEPAL 60, 250 mM NaCl), incubated on ice for 15 min with vortexing twice. Lysates were spun at 17,000 × *g* at 4 °C for 5 min. Supernatants were collected for further analysis. Likewise, 500 and 750 mM NaCl extractions were performed. All buffers were supplemented with complete Protease Inhibitor Cocktail (Roche). Fractions were analysed by Western blots following the protocol described above.

### Immunofluorescence staining and images analysis

Fibroblasts were grown on uncoated glass coverslips, iPSCs on Matrigel-coated glass coverslips, and NPCs and neurons were grown on Poly-L-Ornithine/Laminin-coated (Sigma L2020, P4957) glass coverslips in 24-well plates. Cells were grown on glass coverslips in a 24-well plate and fixed with 4% paraformaldehyde in PBS for 15 min, treated with 0.1 M glycine in PBS for 20 min, permeabilized with 0.2% Triton X-100 in PBS, followed by blocking in 5% normal goat serum in PBS for 1 h. Cells were incubated with primary antibody in 2% bovine serum albumin (BSA) in PBS, and incubated overnight in a humidified chamber at 4 °C (MAP2, Abcam ab5392, 1/2000); NeuN (Millipore MAB377, 1/20); OCT4 (Invitrogen MA1-104, 1/500); Pax6 (Biolegend 901302, 1/100); Nestin (Novus Biologicald NB300266, 1/500); γ-H2AX (Millipore 05-636, 1/250). Coverslips were washed and incubated for 1 h at room temperature with Alexa Fluor secondary antibodies (Life Technologies, 1/200), then incubated with DAPI in PBS (1 μg/mL) for 5 min and washed twice with PBS. Coverslips were mounted on slides using ProLong Gold Antifade Mountant. Multi-plane images were acquired with a Nikon A1R+ confocal microscope using 20 or 60× objectives for neurons or fibroblasts, respectively. Representative images are a z-stack projection of maximum intensity generated by ImageJ Fiji^68^.

For γ-H2AX foci quantification, maximum intensity projection was applied, and a nuclear mask was created. γ-H2AX foci count was performed using the plugin FindFoci (GDSC) and parameters ‘FindFoci’, ‘mask=(None) background_method=[Auto threshold] background_parameter=3.0 auto_threshold=Moments statistics_mode=Both search_method=[Fraction of peak - background] search_parameter=0.8 minimum_size=2 minimum_above_saddle minimum_peak_height=[Relative peak_parameter=0.7 sort_method=[Average height] intensity] maximum_peaks=1000 show_mask=[Peaks above saddle] overlay_mask fraction_parameter=0.5 show_table clear_table mark_-maxima show_peak_maxima_as_dots show_log_messages results_directory=[] gaussian_blur=2.0 centre_method=[Max value (search image)] centre_parameter=2.0’).

### Telomere DNA fluorescence in situ hybridisation

Cells grown on coverslips were fixed with 4% (w/v) paraformaldehyde for 10 min, washed three times with PBS, treated with 250 µg/mL RNase A (Sigma, cat. no. R4875) at 37 °C for 20 min to remove internal RNA. After three PBS washes, cell walls were degraded with prewarmed pepsin solution (1 mg/mL pepsin (Roche, 10108057001) in 10 mM HCl) for 10 min at 37 °C. Following a second fixation in 4% PFA and dehydration through a graded ethanol series (70%, 90%, and 100% v/v ethanol for 3 min each), coverslips were let to air-dry to completion. Then coverslips were hybridised with a TelC-Alexa488 PNA probe (PNA-Bio, F1004) diluted at 1:200 in a buffer (70% deionized formamide, 0.5% FISH blocking reagent, 10 mM Tris pH 7.5). The FISH blocking solution (10% w/v) was prepared by dissolving 10 g of blocking reagent in 100 mL of maleic acid buffer (100 mM maleic acid, 150 mM NaCl, pH 7.5). The mixture was heated to 60 °C with continuous shaking until fully dissolved. DNA denaturation was performed at 85 °C for 15 min, followed by a 16-h hybridisation at 37 °C. Two washes were conducted at 55 °C using 60% formamide/10 mM Tris pH 7.5; and 2X SSC/0.1% Tween-20, respectively. Nuclei were counter-stained with 0.5 μg/mL DAPI, and slides were mounted in Prolong Gold for subsequent imaging.

### cGAMP ELISA

For cGAMP quantification, cells were harvested and lysed in RIPA lysis buffer ((50 mM Tris, 150 mM NaCl, 1% sodium deoxycholate, 0.03% SDS, 0.005% Triton X-100, 5 mM EDTA and complete Protease Inhibitor Cocktail (Roche)) for 30 min on ice. Lysed cells were centrifuged for 10 min at 17,000 × *g* and 4 °C. The supernatant (50 μL*2) was used for the 2′3′-cGAMP ELISA assay (Cayman, 501700) according to the manufacturer’s instructions. Protein concentration in the supernatant was measured using the BCA Pierce Protein Assay Kit and used to normalise 2′3′-cGAMP levels per sample. The IC20 and IC80 values (upper and lower detection limits of the standard curve, respectively) were calculated following manufacturer guidelines, and levels falling outside this range were excluded from the analysis.

### CRISPR/Cas9

For genome editing, direct delivery of the CRISPR/Cas9 system as a ribonucleoprotein (RNP) complex consisting of Cas9 protein and single guide RNA (sgRNA) was used. For *ATRX*: A single Alt-R CRISPR-Cas9 sgRNA targeting *ATRX*, containing the target-specific crRNA region (TTCTCTCTTAGATCATTGTA) and the Cas9-interacting tracrRNA region, was used. To introduce the Y1758C mutation by homologous recombination, CRISPR-Cas9 was used with a single-stranded oligodeoxynucleotide (ssODN) of 200 bp (CTAATGCATGTGTTTTTGACATGAGCATTTCATTGGGGAATTTTTCTAAGTCTT TTTAACGATTGTTAATGTTGTTAATGAATAATGTCTTCTCT CTTAGGTCACTGCATGGTTAATTTTATCAAGGAAAATTTACTTGGATCCATTAAGGAGTTCAGGAATAGATTTATAAATCCAATTCAAAATGGT CAGTGTGCAGA). SgRNA, ssODN and Alt-R S.p. Cas9 Nuclease 3NLS (IDT 1081059) were purchased from IDT. RNP complex, with or without ssODN, was delivered to cells by electroporation using 4D-Nucleofector system. Clonal expansion was assessed after 2–4 weeks, and established single clones were genotyped for ATRX indels and knock-ins. PCR primers: TTCACAAGATAGCCTCACTGACC and GGTAGAATCTGCACACTGACCA. For *STING1*: the target-specific crRNA region was GCTGGGACTGCTGTTAAACG, and PCR primers AGGGGAACTGGGAGGAAGTGCC and CCAAGGCTTCCCAACCTGCTCC. For *DAXX*, the target-specific crRNA region was GCTCACATAGTCGCCCAAAG, and PCR primers CTGTTTTTGGCCTCGGCGGAGT and GCATCCCACCAACCCCCTACCT. Genome editing was assessed in pool cells prior to seeding for single-cell clonal expansion, using the ICE CRISPR Analysis Tool.

### Pathway enrichment analysis

GSEA^69^ desktop application was used to perform hallmark gene sets analysis from MSigDB^70^ and by using the recommended settings (parameters scoring_scheme classic and metric Signal2Noise). When specified, the STRING database online was used (https://string-db.org/) and the enrichment analysis of REACTOME pathways displayed.

### RNA sequencing and analysis

For iPSCs, three independent clones were used. For neurons, four replicates were used (three independent clones, of which one is repeated twice). RNA was extracted using RNeasy Micro Kit (Qiagen, 74004). RNA samples were assessed using the Fragment Analyser Automated Capillary Electrophoresis system (Agilent Technologies Inc., 5300) with the Standard Sensitivity RNA Analysis kit (DNF-471-0500) for quality and integrity of total RNA, and then quantified using the Qubit 2.0 Fluorometer (Thermo Fisher Scientific Inc., Q32866) with the Qubit RNA HS Assay kit (Q32855). Libraries were generated using the QuantSeq 3′mRNA-Seq Library Prep kit (FWD) protocol for Illumina (Lexogen Inc, #015). Single read (1 × 75bp) sequencing was performed on the NextSeq 550 platform (Illumina Inc., SY-415-1002) using the NextSeq 500/550 High-Output v2.5 kit (20024906). Libraries were combined in a single equimolar sample based on Qubit and Bioanalyser assay results, and run across a High Output v2.5 Flow Cell. The QuantSeq and differential data analyses were performed following pipeline integration on the Bluebee genomics analysis platform.

For anifrolumab treatment, three independent clones per genotype were used, and cells were treated for 24 h with 10 μg/mL of anifrolumab (MedChemExpress HY-199168) or water. Total RNA was extracted and processed as previously described to generate libraries using the QuantSeq 3′mRNA-Seq Library Prep kit (FWD) protocol for Illumina. As an initial processing step, the raw fastq files were pretrimmed from the 5′ end of the first poly-A sequence of length 10 or more. This trimming removed the poly-A sequences themselves along with any downstream nucleotides. The trimming was performed using a custom script implemented in Biopython^71^. The pipeline used is deposited on Zenodo with the following https://doi.org/10.5281/zenodo.22092978. Briefly, read counts over human genes were then obtained by running the pre-trimmed fastq files through the nf-core RNA-Seq pipeline using fastp^72^ for adapter trimming, STAR^73^ for alignment, and Salmon^74^ for quantification. The gene-level counts obtained were then used to perform pairwise differential expression analysis using DESeq2^75^.

For BJ-hTERT lines, four replicates were used (three independent clones, of which one is repeated twice). Libraries were prepared using the NEBNEXT Ultra II Directional RNA Library Prep kit (NEB #7760), and the Poly-A mRNA magnetic isolation module (NEB #E7490) used according to the manufacturer’s protocol. Final libraries had an average peak size of 286 bp. Fragment size and quantity measurements were used to calculate molarity for each library pool. Sequencing was performed on the NextSeq 2000 platform (Illumina Inc., #20038897) using NextSeq 2000 P3 Reagents (100 Cycles) (#20040559).

RNA-Seq read data were processed through the nf-core/rnaseq (v3.10.1) pipeline (doi: 10.5281/zenodo.1400710) using the genome reference GRCh38 (GRCh38.p13) and Ensembl gene models. We used the DESeq2 package (version 1.42.1) in R (version 4.3.3) to perform differential gene expression analysis between various genotypes^75^. We obtained gene counts from the nf-core/rnaseq pipeline output^76^ (salmon.merged.gene_counts_length_scaled.tsv) and imported them into DESeq2 using the DESeqDataSetFromMatrix method. We normalised the counts using the variance stabilising transformation (VST) and performed principal component analysis (PCA) to visualise the sample clustering. DESeq2 fitted a negative binomial generalized linear model with genotype as the main factor and used the Wald test to identify differentially expressed genes. We applied the ashr method to shrink the log2 fold changes for estimation stability and improved ranking of genes^77^.

### CUT&Tag processing and data analysis

Two independent clones and two technical replicates were used per genotype. Experiments were performed following the protocol described in ref. 39. Briefly, fresh cells (100,000 cells per reaction) were harvested and lysed. Nuclei were isolated and bound to Concanavalin A (ConA)-coated beads. Primary antibodies were: mAb IgG XP Isotype Control CST 66362, ATRX rabbit ab97508, DAXX Prestige-HPA065779, H3.3 Epicypher 13-0061. Zero point five micrograms of antibody in antibody buffer (0.01% BSA in wash buffer) was added to beads and incubated for 2 h at room temperature, then overnight at 4 °C. Beads were cleared, mixed with the secondary antibody (Guinea Pig anti-Rabbit IgG, antibodies-online ABIN101961) at a dilution of 1:100 in antibody buffer, and incubated for 1 h at room temperature. Beads were washed and resuspended in Protein A/G-Tn5 (Epicypher 15-1017) for 2 h. Tagmentation was performed for 1 h at 37 °C in 300-Wash buffer supplemented with 10 mM MgCl_2_. Beads were washed, and tagmented fragments released. Following 1 h incubation at 58 °C, SDS was neutralised with 0.67% Triton-X100. Barcoded i5 (v2_Ad1) and i7 (v2_Ad2) primer^78^ and NEBNext 2x Master Mix (NEB, M0541L) were added, and tagmented fragments were amplified by PCR. PCR clean-up was performed using AMPure XP bead slurry following the manufacturer’s instructions. Equimolar libraries were made, and sequencing (2 × 50) was performed on the NextSeq 2000 platform using the NextSeq 1000/2000 P2 Reagents (100 cycles) v3 Kit (#20046811).

Sequencing reads were processed with the nf-core/cutandrun pipeline (3.2.2) (doi: 10.5281/zenodo.10606804), implemented in Nextflow (v24.04.2). Briefly, Adapter and quality trimming of reads were performed using Trim Galore (0.6.6). Reads were aligned using Bowtie2 (2.4.4) to the human reference genome (GRCh38.p13) and to an *E. coli* K-12 MG1655 (spike-in). Both duplicate reads (Picard: 3.1.0) and reads aligning within ENCODE blacklist regions (hg38 blacklist v1) were removed prior to peak calling. Peaks were called with SEACR (1.3) (stringent, threshold = 0.01). Using Galaxy^79^, 1/BigWig files were generated using the bamCoverage tool, normalised to 1× sequencing depth (Reads Per Genomic Content (RPGC)), and averaged with the bigwigAverage tool^80^, 2/Peaks were annotated using Chipseeker^81^, 3/Diffbind^82^ tool was employed to identify differentially bound sites between two groups, and 4/computeMatrix and PlotHeatmap tools were used to generate enrichment profiles.

For principal component analysis (PCA) of ATRX, peaks from the two biological replicates were intersected using ‘bedtools intersect’ (version v2.31.1). The union of peaks of all genotypes, using ‘bedtools merge’, was made to create a combined list of positions. Reads mapped at to these coordinates were counted using featureCounts (from the subread package, version v2.0.8). DESeq2 package (version 1.34.0) in R (version 4.1.2) was used to assess differential peak coverage between various genotypes^75^. The counts were normalised using the variance stabilizing transformation (VST), and performed PCA to visualise the sample clustering. DESeq2 fitted a negative binomial generalized linear model with genotype as the main factor and used the Wald test to identify differentially bound regions.

For H3.3 PCA and differential binding on genes, read counts were obtained over gene bodies using positions of UniprotKB/Swiss-Prot genes, using Biomart (Ensembl version 113), adding 3 kb on each side of the gene position. Again, we used the DESeq2 package (version 1.34.0) in R (version 4.1.2) to perform differential gene body coverage analysis between various genotypes.

### Quantification and statistical analysis

Statistical analyses for all experiments, except sequencing data, were performed in GraphPad Prism v10 or v11. Statistical parameters are reported in the figure legends.

### Reporting summary

Further information on research design is available in the Nature Portfolio Reporting Summary linked to this article.

## Supplementary Material

**Supplementary information** The online version contains Supplementary Material available at https://doi.org/10.1038/s41467-026-78094-8.

Supplementary

## Figures and Tables

**Table 1:** 

Strain	Genotype	Source
yJF1	*MATa ade2-1 ura3-1 his3-11,15 trp1-1 leu2-3,112 can1-100 bar1Δ::hphNT pep4Δ::kanMX*	Frigola et al.^83^
yJF1-ATRX-WT	*MATa ade2-1 ura3-1 his3-11,15 trp1-1 leu2-3,112 can1-100 bar1Δ::hphNT pep4Δ::kanMX leu2::pATRX_Y_WT (Gal1,10-hATRX-FLAG)*	This study
yJF1-ATRX-K1600R	*MATa ade2-1 ura3-1 his3-11,15 trp1-1 leu2-3,112 can1-100 bar1Δ::hphNT pep4Δ::kanMX leu2::pATRX_Y_K1600R (Gal1,10-hATRX-FLAG)*	This study
yJF1-ATRX-Y1758C	*MATa ade2-1 ura3-1 his3-11,15 trp1-1 leu2-3,112 can1-100 bar1Δ::hphNT pep4Δ::kanMX leu2::pATRX_Y_Y1758C (Gal1,10-hATRX-FLAG)*	This study

## Data Availability

Sequencing data generated for this study are available through Gene Expression Omnibus (GEO) with the accession number GSE212923. Previously published transcriptional data were obtained from the GEO repository, with the accession numbers GSE107878, GSE12454 and GSE100464. All other data are available in the article and its Supplementary files or from the corresponding author upon request. Source data are provided with this paper.

## Code availability

The pipeline used to analyse the QuantSeq 3’ mRNA-Seq of ATRX-deficient BJ-hTERT fibroblasts under anifrolumab treatment is deposited on Zenodo with the following https://doi.org/10.5281/zenodo.22092978. Source data are provided as a Source Data file.
