# Supplementary material for: ATRX deficiency drives aberrant type I interferon signalling through cGAS-dependent transcriptional dysregulation
Source: Nat Commun. 2026 Oct 8;17:11551. doi: 10.1038/s41467-026-78094-8 (PMC7619637; doi:10.1038/s41467-026-78094-8)
Supplement: Supplementary file 1 [file 41467_2026_78094_MOESM1_ESM.pdf]

SUPPLEMENTARY FIGURES

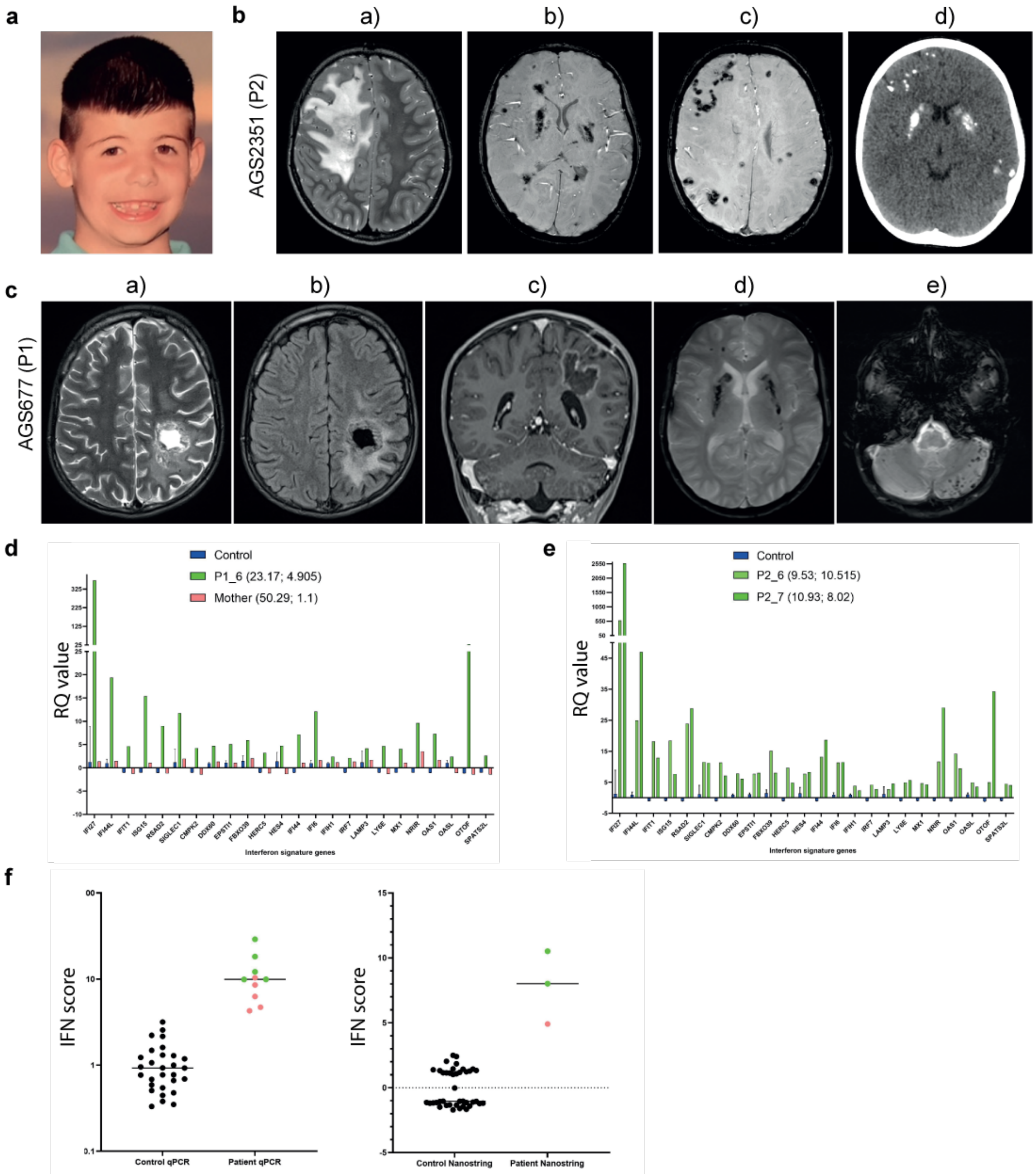

Figure S1. Clinical features and interferon scores in P1 and P2

a Facial appearance of P2. **b** Cerebral imaging in P2: MRI imaging at seven years of age shows a large area of abnormal white matter signal in the right fronto-parietal region, with a cystic area within it (a) (axial T2), and extensive, bilateral but asymmetric, mineralisation in the basal ganglia (b, c) (axial SWI), which was confirmed to be calcification by CT (d: taken at age of five years). **c** Cerebral imaging in P1 at 14 years of age: MRI imaging demonstrates a large cystic lesion in the left parietal region with heterogeneous signal intensity (a: axial T2, b: axial FLAIR) with surrounding contrast enhancement (c: coronal post-contrast T1). There is dense mineralisation of the basal ganglia bilaterally (d: axial GRE) and in the left cerebellar

hemisphere (e: axial GRE). **d-e** Relative quantification (RQ) values of 24 interferon stimulated genes (ISGs) measured on a NanoString platform in whole blood of P1 (d) and P2 (e). The first number in brackets is the decimalised age in years at sampling, the second number is the interferon (IFN) score, with scores above 2.75 designated as positive. Dark blue bars represent the composite data of 27 controls. Note that the IFN score in whole blood of the mother of P1 was normal on the single occasion tested. **f** Left: dot plot showing the IFN score calculated from the median fold change of six ISGs (*IFI27, IFI44L, IFIT1, ISG15, RSAD2, SIGLEC1*) measured by qPCR in whole blood of controls, P1 and P2, and related to Figure 1d. Right: dot plot representation of the data shown in d-e. Red = P1, green = P2. Blask bar: median.
Source data are provided as a Source Data file.

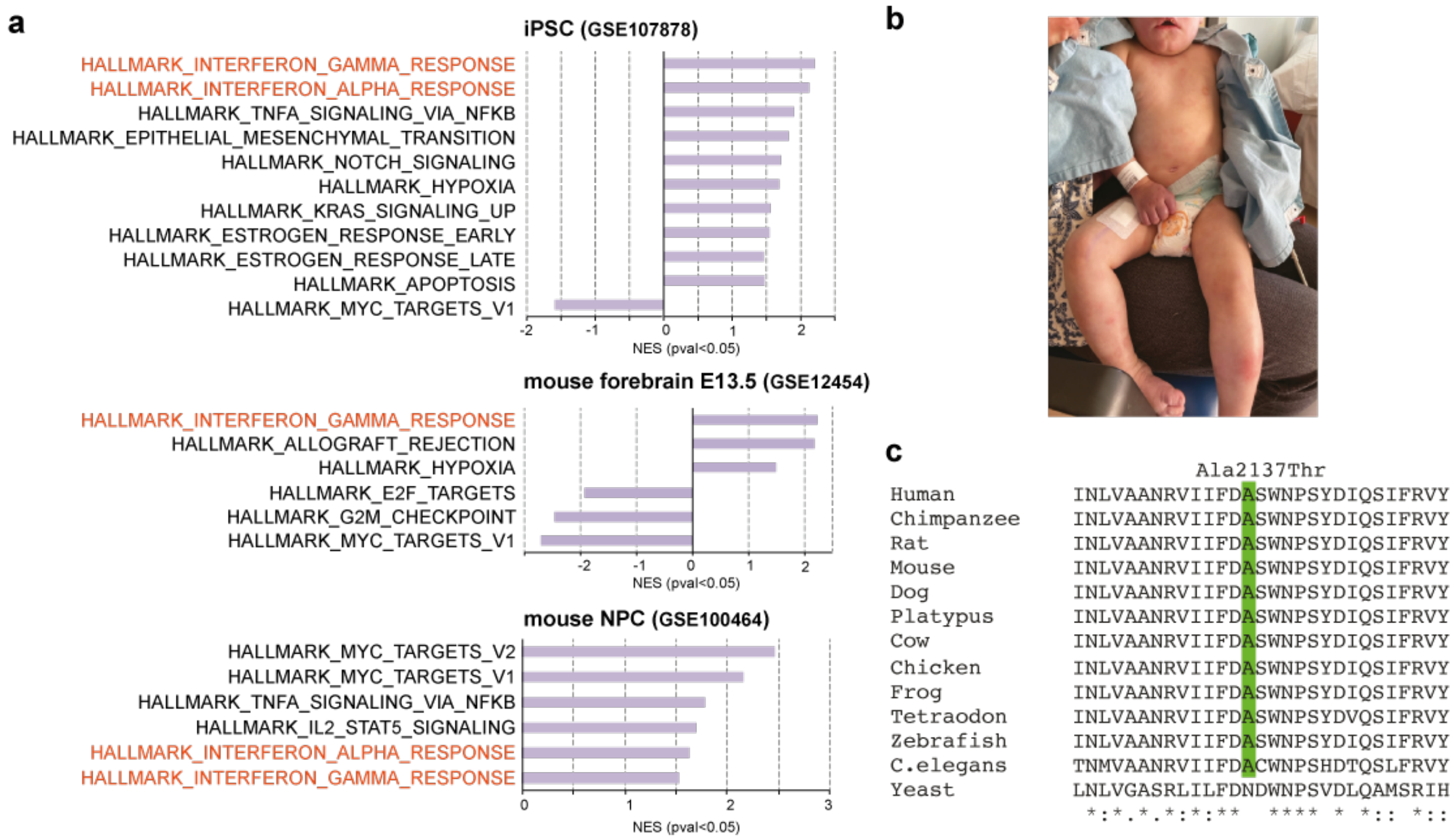

Figure S2. Gene set enrichment analysis (GSEA) of publicly available transcriptomic datasets relating to three ATRX deficient models, and clinical features of P3

a Normalised enrichment scores (NES) of GSEA values with a p value < 0.05 of RNA-Seq data in *ATRX*-null induced pluripotent stem cells (iPSCs) (top), microarray data in *Atrx*-null mouse forebrain (middle) and RNA-Seq data in *Atrx*-null mouse neuronal progenitor cells (NPC) (bottom). Interferon-related pathways are highlighted in orange. Note: genes from the interferon gamma dataset overlap with the interferon alpha dataset. Transcriptional data were obtained from the Gene Expression Omnibus (GEO) repository, with the accession numbers GSE107878[1], GSE12454[2] and GSE100464[3] as indicated above each data set. **b** Image showing the inflammatory skin lesions observed in P3. Note the appearance of the mouth, typical of ATR-X syndrome. **c** CLUSTAL Omega alignment of ATRX homologs. The position of the substituted A2137T in P3 is indicated in green.

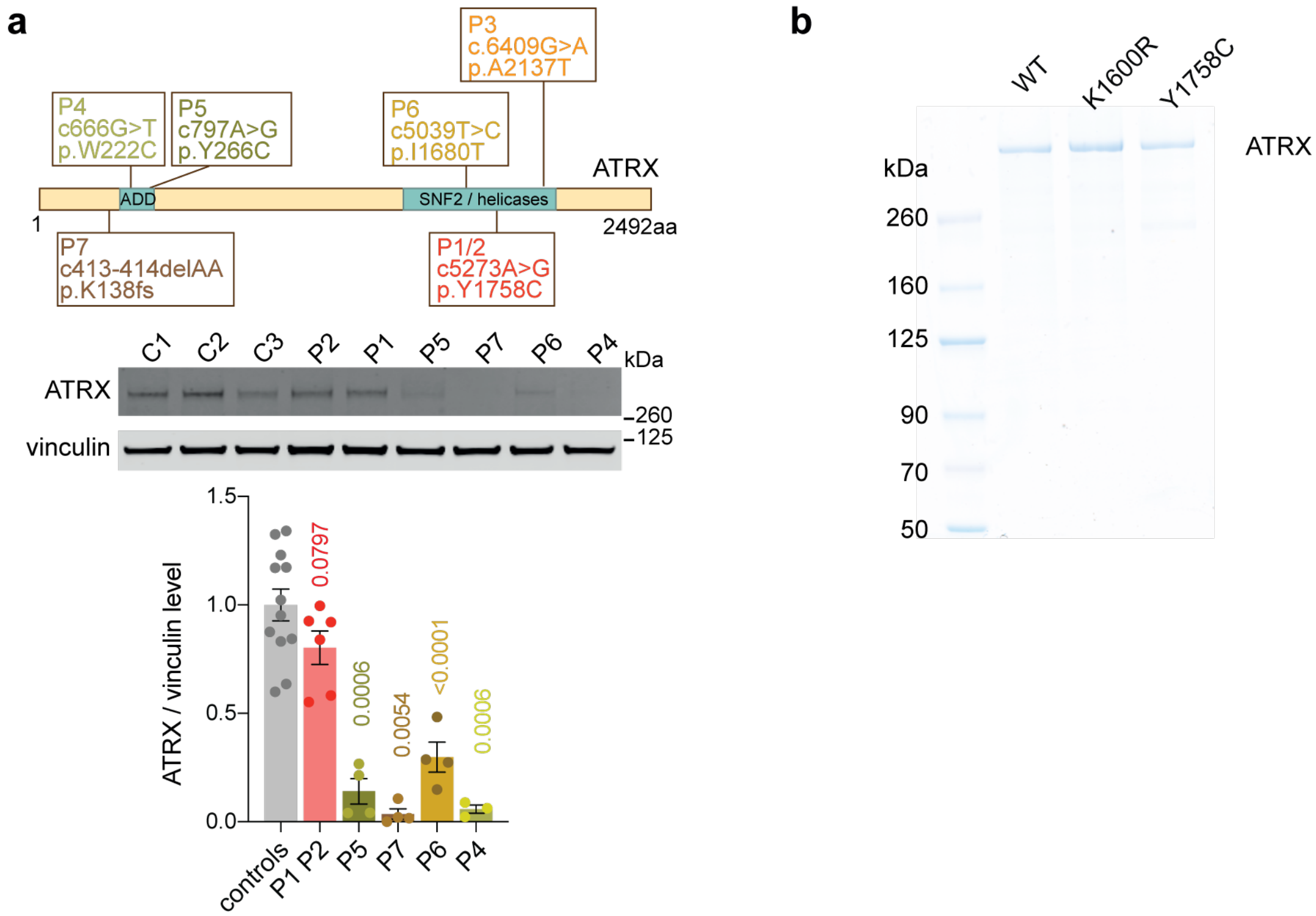

Figure S3. ATRX protein levels in patient primary fibroblasts and purified from *Saccharomyces Cerevisiae*

a Schematic of the ATRX protein (top) illustrating the mutations in P1 – P7; ADD = ATRX-DNMT3-DNMTL-like domain (amino acid (aa) residues 161-292; SNF2 (Sucrose Non-Fermentable) domain encompassing ATPase/Helicase domains, residues 1550-2226. Representative western blot (middle) and quantification (bottom) of ATRX protein in primary fibroblasts from controls (C) and indicated patients (P), [each circle represents an independent replicate, Kruskal-Wallis test, two-stage linear step-up procedure of Benjamin, Krieger and Yekutieli, q values are indicated, mean +/- SEM]. **b** Purification of recombinant human ATRX protein in budding yeast. SDS-PAGE of 1 µg of pooled fractions from WT, K1600R, and Y1758C ATRX, stained with InstantBlue Coomassie protein stain.
Source data are provided as a Source Data file**.**

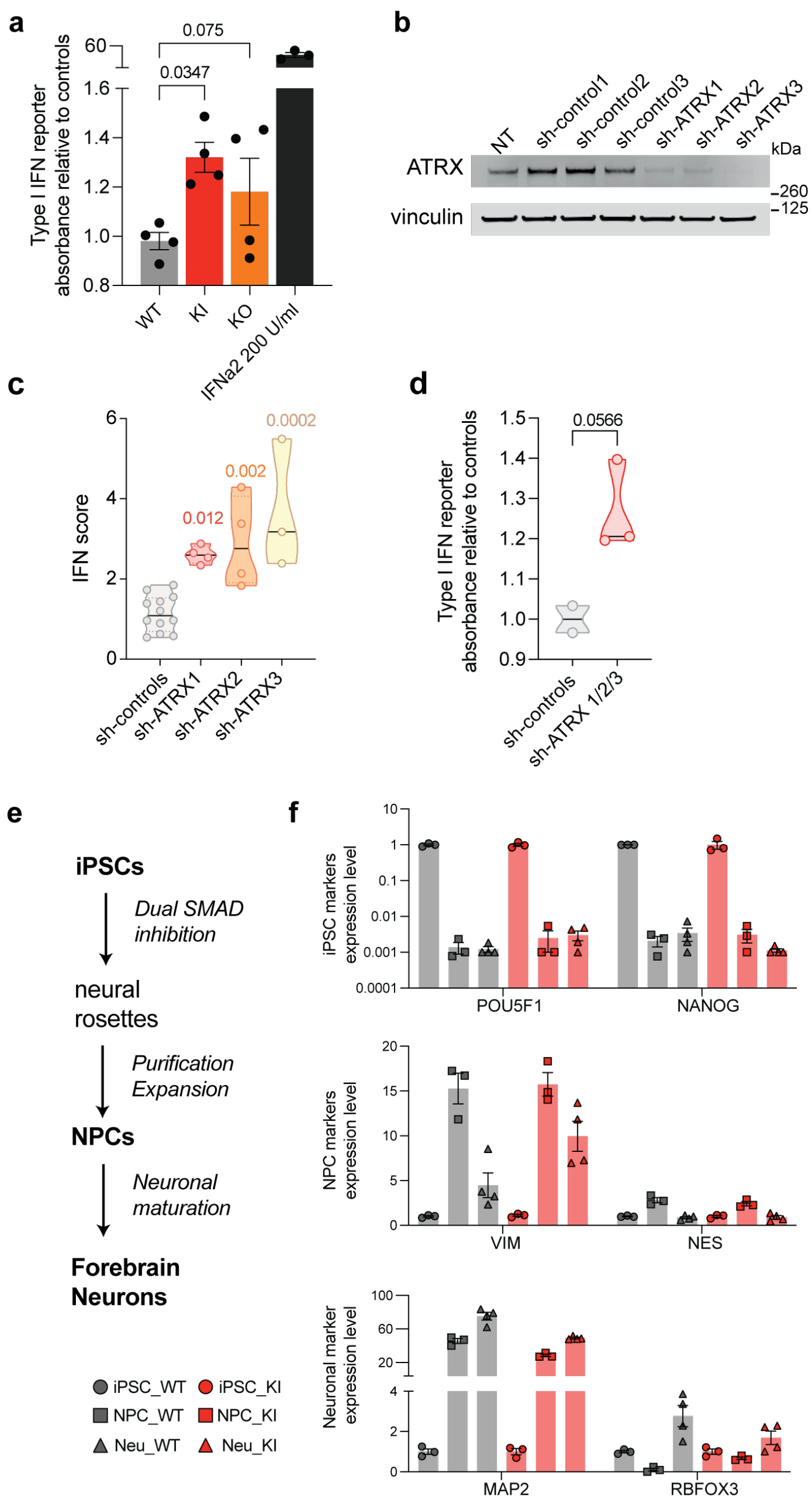

Figure S4. Characterisation of ATRX deficient cell lines

a Quantification of secreted type I interferon in supernatant of BJ-hTERT fibroblasts KI or KO for *ATRX*, measured using a secreted embryonic alkaline phosphatase assay in HEK-Blue^{TM} ISG reporter cells. As a positive control, HEK-Blue cells were treated with 200 U/mL IFNa2 for 24 hours, [four independent experiments, Ordinary one-way ANOVA, two-stage linear step-up procedure of Benjamin, Krieger and Yekutieli, q values are indicated, mean +/- SEM]. **b** ATRX protein expression assessed by western blot in THP-1 cells following knock-down of *ATRX* using three short-hairpin (sh) RNAs. **c** Interferon (IFN) score (median expression of *IFI27, RSAD2, ISG15, IFI44L, IFIT1*) in THP-1 cells five days post-transduction with control short-hairpin (sh-controls) or three different sh-RNAs targeting *ATRX* (sh-ATRX), [each circle represents an independent repeat of an sh-RNA, n=4, ordinary one-way ANOVA, Dunnett's multiple

comparisons test, p values are indicated, black bars represent the median]. **d** Quantification of secreted type I interferon activity in supernatant from THP-1 cells upon knock-down of *ATRX* by sh-RNA (day 8 post-transduction), same as (a), [each circle represents a different sh-RNA, n=1, unpaired t-test]. **e** Schematic of differentiation of human iPSCs (induced Pluripotent Stem cells) to NPCs (Neural Stem cells) and then to forebrain neurons following neural induction and neuronal maturation using an established StemCell protocol. **f** Relative gene expression levels (expression in iPSC is set to 1) of stem cell and neural subtype markers measured by 3' end mRNA sequencing in iPSCs, NPCs and neurons (Neu) WT or *ATRX* KI for the Y1758C substitution.
Source data are provided as a Source Data file.

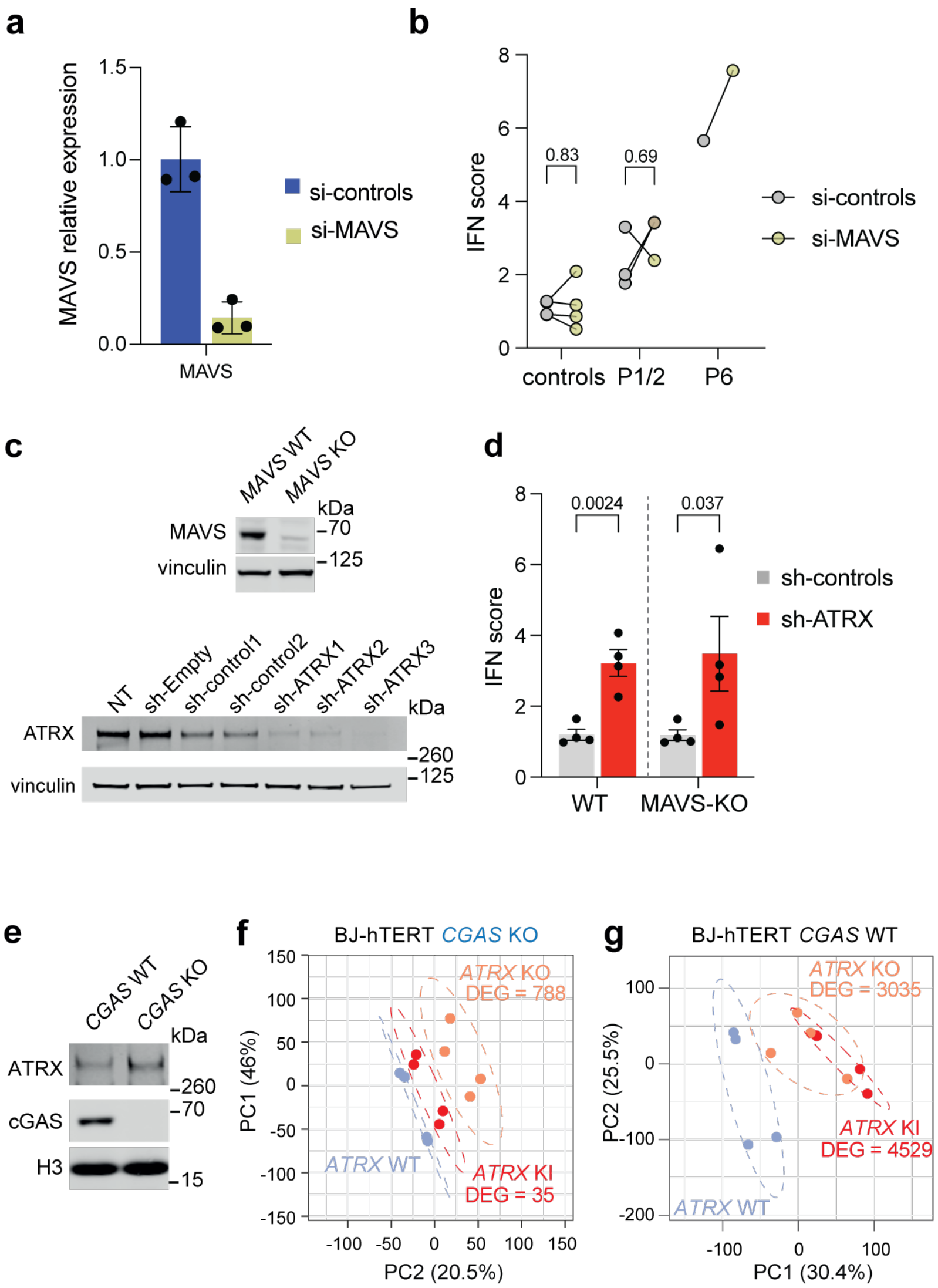

Figure S5. Interferon signalling pathway analysis

a Efficiency of *MAVS* RNA knock-down in primary fibroblasts [three independent experiment]. **b** Effect on the interferon (IFN) score (median expression of *TNFAIP3, RSAD2, Mx1, ISG15, IFI44L, IFIT1, IFI6*) of si-RNA knock-down of *MAVS* in primary fibroblasts from controls, Y1758C ATRX mutant (P1 and P2) and 'classical' ATRX (P6) mutant fibroblasts, [circles represent individual repeats, Ratio paired t tests, q values are indicated]. **c** Protein expression assessed by western blot of MAVS (top) and ATRX (bottom) in THP-1 cells KO for *MAVS*, following knock-down of *ATRX* using three short-hairpin (sh) RNAs. **d** Interferon (IFN) score (median expression of *IFI27*, *IFIT1*, *RSAD2*, *ISG15*) in THP-1 cells five days post-transduction with control sh-RNAs (sh-controls) or three different sh-RNAs targeting *ATRX* (sh-ATRX) [each circle represents the mean of an independent experiment, n=4; Unpaired t test, Two-stage linear step-up procedure of Benjamini, Krieger and Yekutieli, q values are indicated, mean +/- SEM]. **e** Protein levels assessed by western blot in nuclear fractions of BJ-hTERT clones WT or KO for *CGAS*. **f-g** Principal component (PC) analysis of RNA-Seq data derived from four BJ-hTERT fibroblasts clones KO (f) or WT (g) for *CGAS*, and WT, KI or KO for *ATRX*. The number of differentially expressed genes (DEG, p adjusted < 0.1) identified between WT and KI or WT and KO *ATRX* cells are noted on the graph.
Source data are provided as a Source Data file.

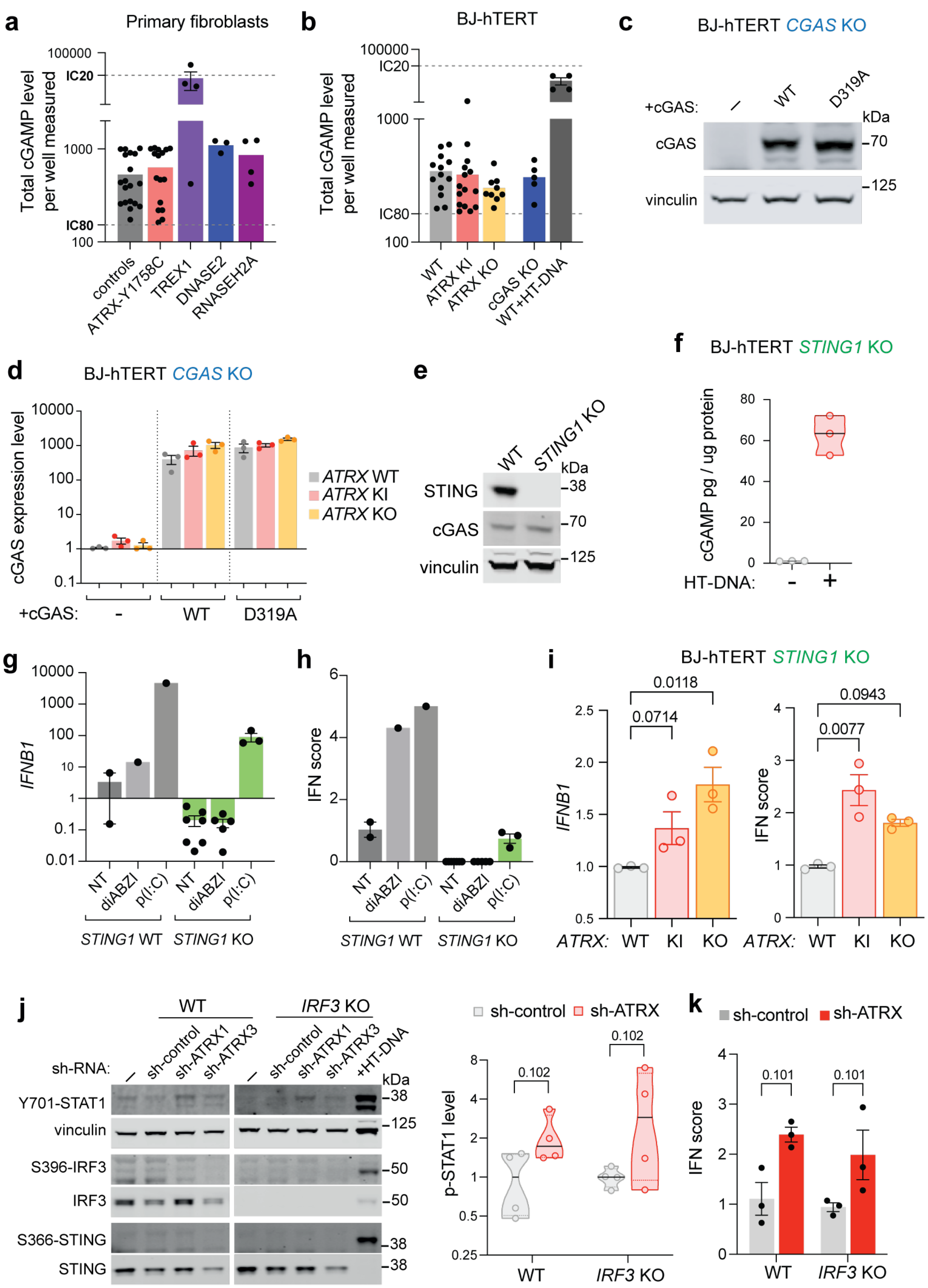

Figure S6. Canonical DNA sensing pathway analysis

a-b Graphs illustrating total cGAMP levels measured by ELISA in wells containing total cellular lysate obtained from primary fibroblasts (a) or BJ-hTERT clones (b): dotted lines indicate the level of IC20 and IC80, representing the upper and lower limits of detection of the standard curve, respectively. Dots represent

individual measurements and relate to Figure 4a and 4b. **c-d** Western blot (c) and qPCR (d) showing protein and RNA levels, respectively, of transfected WT or catalytic inactive (D319A mutant) cGAS constructs. **e** Protein levels assessed by western blot of BJ-hTERT fibroblasts clones WT or KO for *STING1*. **f** cGAMP levels measured in BJ-hTERT fibroblasts KO for *STING1* unstimulated (-) or stimulated (+) with HT-DNA for four hours, [one experiment, three replicates]. **g-h** *IFNB1* gene expression (g) and interferon (IFN) score (h) (median expression of *IFI27, IFI6, IFIT1, OAS1, ALDH1A1, IFIH1*) in BJ-hTERT WT or KO for *STING1*, unstimulated (NT), and stimulated with STING agonist diABZI or the MAVS agonist p (I:C) (synthetic analogue of double-stranded RNA polyinosinic-polycytidylic acid). **i** IFN score (median expression of *IFI27, IFI6, IFIT1, OAS1, ALDH1A1, IFIH1*) (left) and relative expression of *IFNB1* (right) measured by qPCR in BJ-hTERT fibroblasts KO for *STING1*, and WT, KI or KO for *ATRX* [each circle represents the mean of an individual clone, n=3, Kruskal-Wallis test, two-stage linear step-up procedure of Benjamin, Krieger and Yekutieli, mean +/- SEM]. **j** Left: Immunoblot of phosphorylated STAT1 (Y701), STING (S366), phosphorylated IRF3 (S396), total IRF3 and STING in THP-1 cells WT or KO for *IRF3*, transduced with control sh-RNAs or sh-RNAs targeting *ATRX*. The last lane relates to WT THP-1 cells stimulated with HT-DNA for four hours; Right: Quantification of STAT1 phosphorylation level over vinculin, [two independent experiments, circles are individual repeats, black bars indicate the means, Kruskal-Wallis test, two-stage linear step-up procedure of Benjamin, Krieger and Yekutieli, q values are indicated, black bars are median]. **k** IFN score (median expression of *IFI27*, *IFIT1*, *RSAD2*, *ISG15*) in THP-1 cells five days post-transduction with controls sh-RNA (sh-control) or three different sh-RNAs targeting *ATRX* (sh-ATRX) [each circle represents an sh-RNA, one independent experiment, Mann-Whitney tests, q values are indicated, mean +/- SEM]. Source data are provided as a Source Data file.

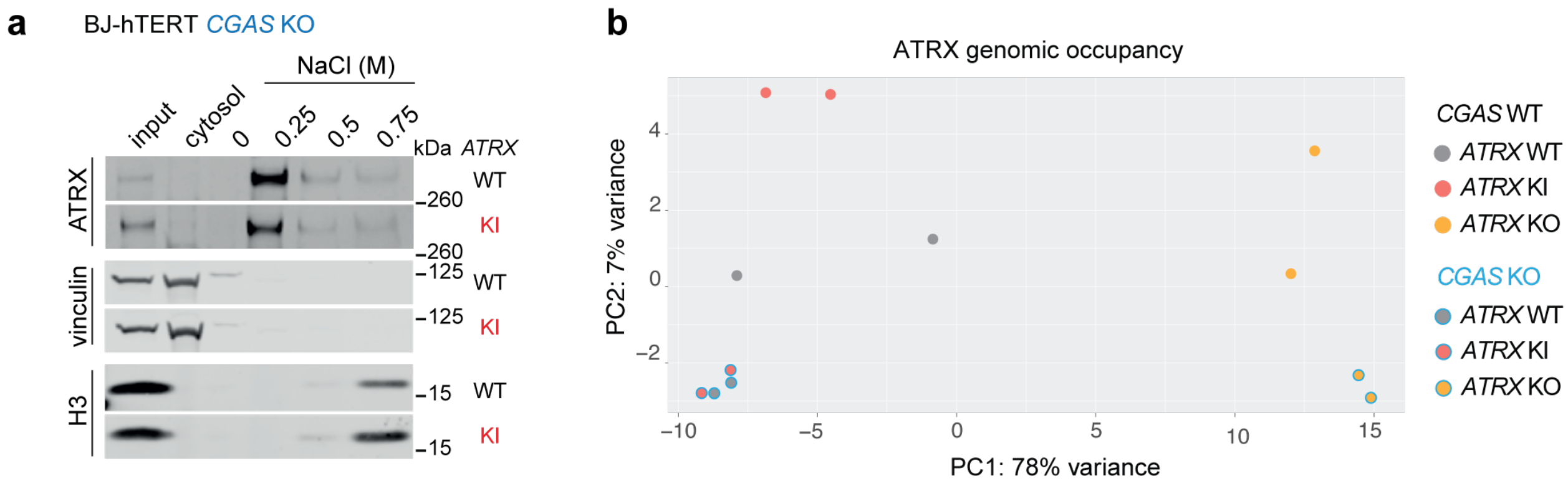

Figure S7. Characterization of ATRX Y1758C chromatin binding

a Subcellular fractionation of BJ-hTERT fibroblasts WT or KI for *ATRX*. Cells were separated into cytosolic and nuclear fractions, followed by sequential elution of nuclear pellets with the indicated concentrations of NaCl. ATRX, vinculin and H3 were assessed by western blot. **b** Principal component (PC) analysis comparing the ATRX genomic binding profiles between indicated genotypes.

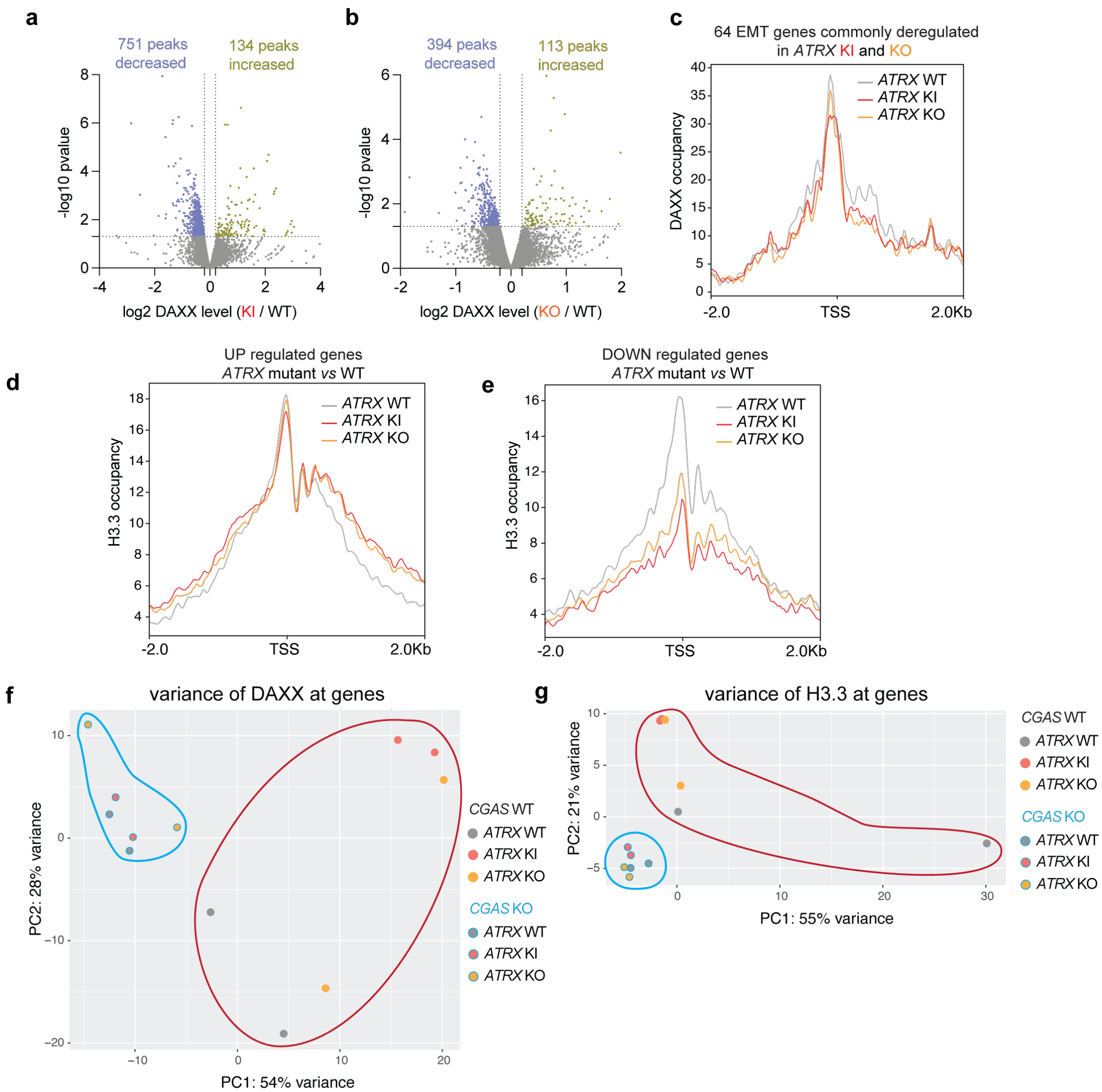

Figure S8. Loss of ATRX function disrupts DAXX and H3.3 occupancy at genes

a-b Volcano plots displaying the results of a genome-wide differential binding analysis of DAXX between KI (a) or KO (b) and WT *ATRX* BJ-hTERT fibroblasts; grey, purple and green dots represent biding sites (peaks) that are, respectively, unchanged, depleted or enriched (p value < 0.05) in ATRX KI or KO cells. **c** DAXX occupancy assessed by CUT&Tag at +/- 2 kb of transcription start sites (TSS) of EMT-related genes dysregulated in BJ-hTERT fibroblasts KI and KO for *ATRX*, as compared to WT. **d-e** H3.3 occupancy assessed by CUT&Tag at +/- 2 kb of transcription start sites (TSS) of genes upregulated (d) or downregulated (e) in BJ-hTERT fibroblasts KI and KO for *ATRX*, as compared to WT. **f-g** Principal component (PC) analysis comparing DAXX (f) and H3.3 (g) genomic binding profiles between indicated genotypes. Source Data are provided as a Source Data file.

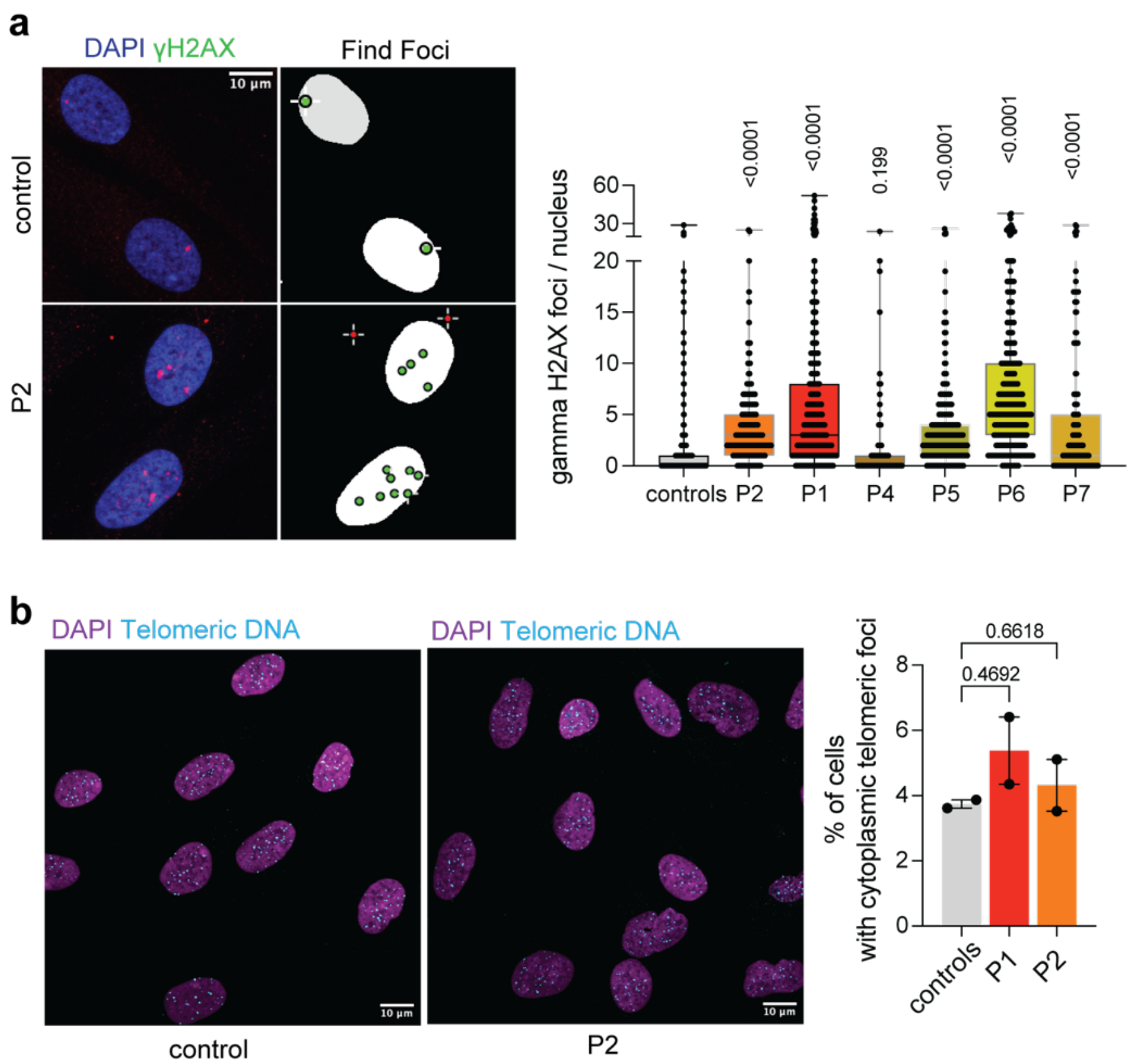

Figure S9. Features of genomic instability in ATRX patient fibroblasts

a Staining of gamma-H2AX (gH2AX) (left) and foci quantification (right) detected using the FindFoci (GDSC) plugin and measured in three controls and patient primary fibroblasts, [two independent experiments, total number of cells analysed are 941 in controls, 201 in P1, 505 in P2, 258 in P5, 293 in P6, 178 in P7, 110 in P4, Ordinary one-way ANOVA, two-stage linear step-up procedure of Benjamin, Krieger and Yekutieli, p values are indicated, violin plot, min to max, show all points]. **b** Analysis of telomeric DNA by fluorescence in situ hybridization (Tel-FISH) (cyan) in three controls and patients P1 and P2 primary fibroblasts, and nuclei were visualized by DAPI staining (magenta), [two independent experiments, total cells numbers are 340 in controls, 147 cells in P1 and 279 cells in P2, Ordinary one-way ANOVA, two-stage linear step-up procedure of Benjamin, Krieger and Yekutieli, q values are indicated, mean+/-SEM]. White bars represent a scale of 10 µm. Source data are provided as a Source Data file.

Uncropped western blots for supplementary figures.

For all blots, this ladder was used: Chameleon duo pre-stained protein ladder (Licor P/N: 928-60000).

Figure S3a.

Red channel ATRX (280 kDa)

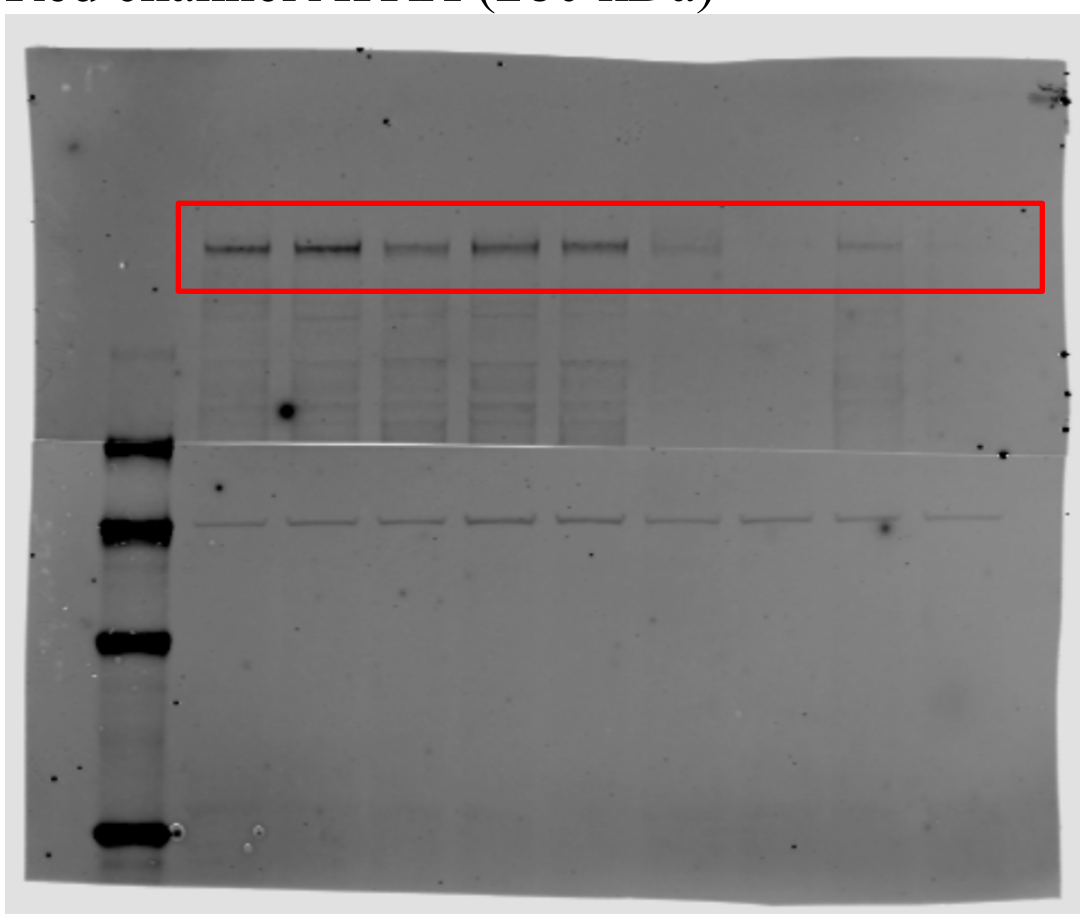

Green channel (vinculin: 120 kDa)

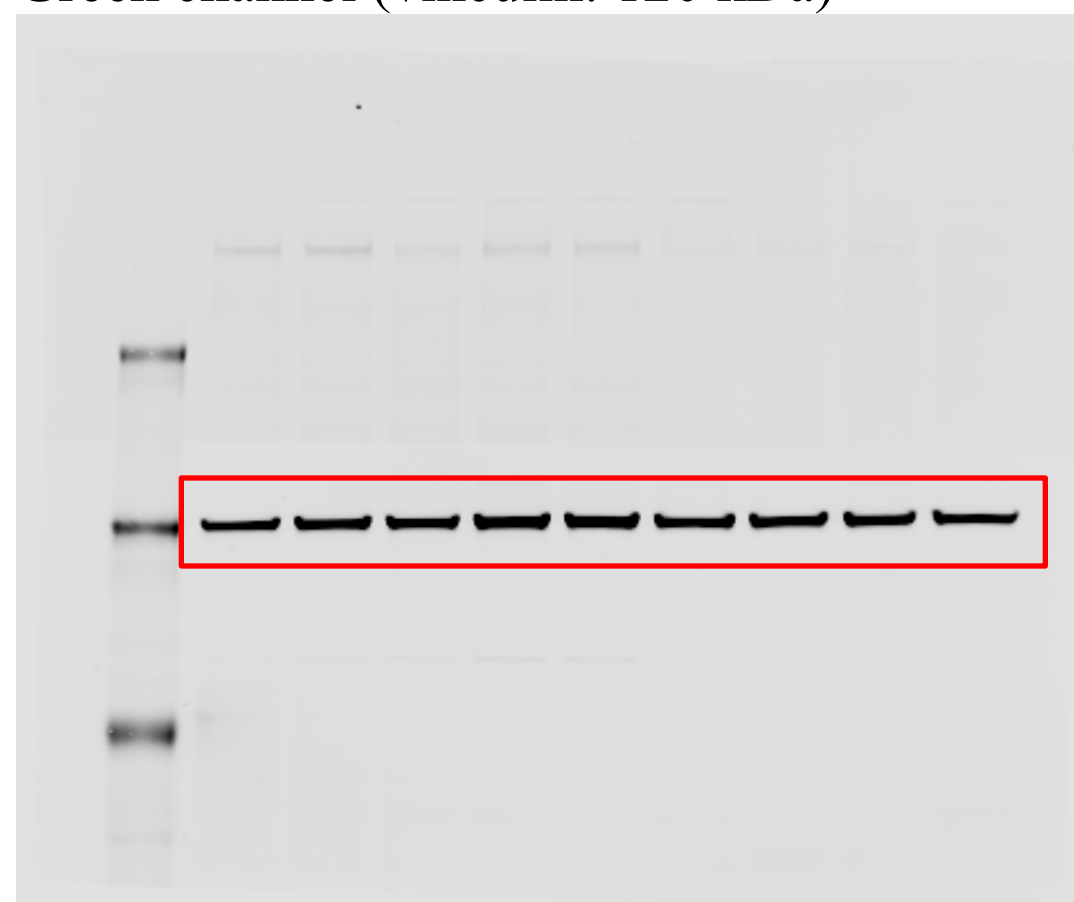

Figure S4b.

Green channel (vinculin: 120 kDa)

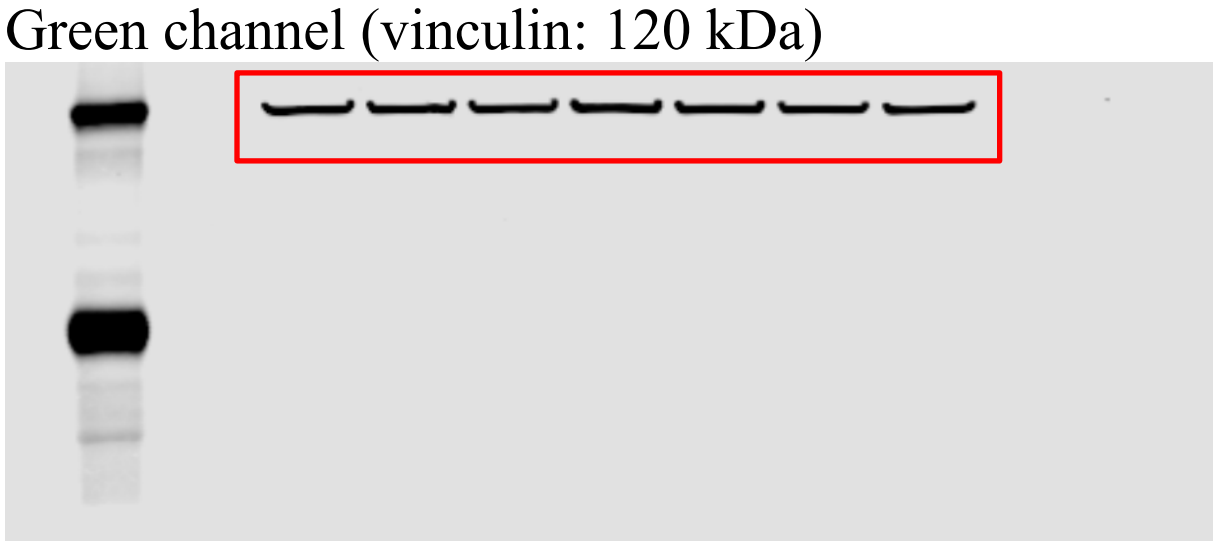

Green channel ATRX (280 kDa)

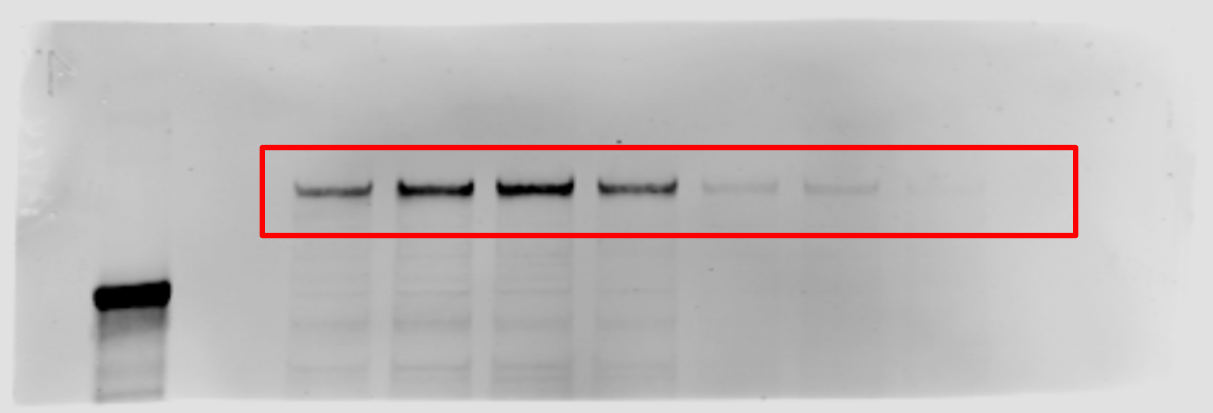

Figure S5c.

Green channel (MAVS: 70 kDa)

Low exposure

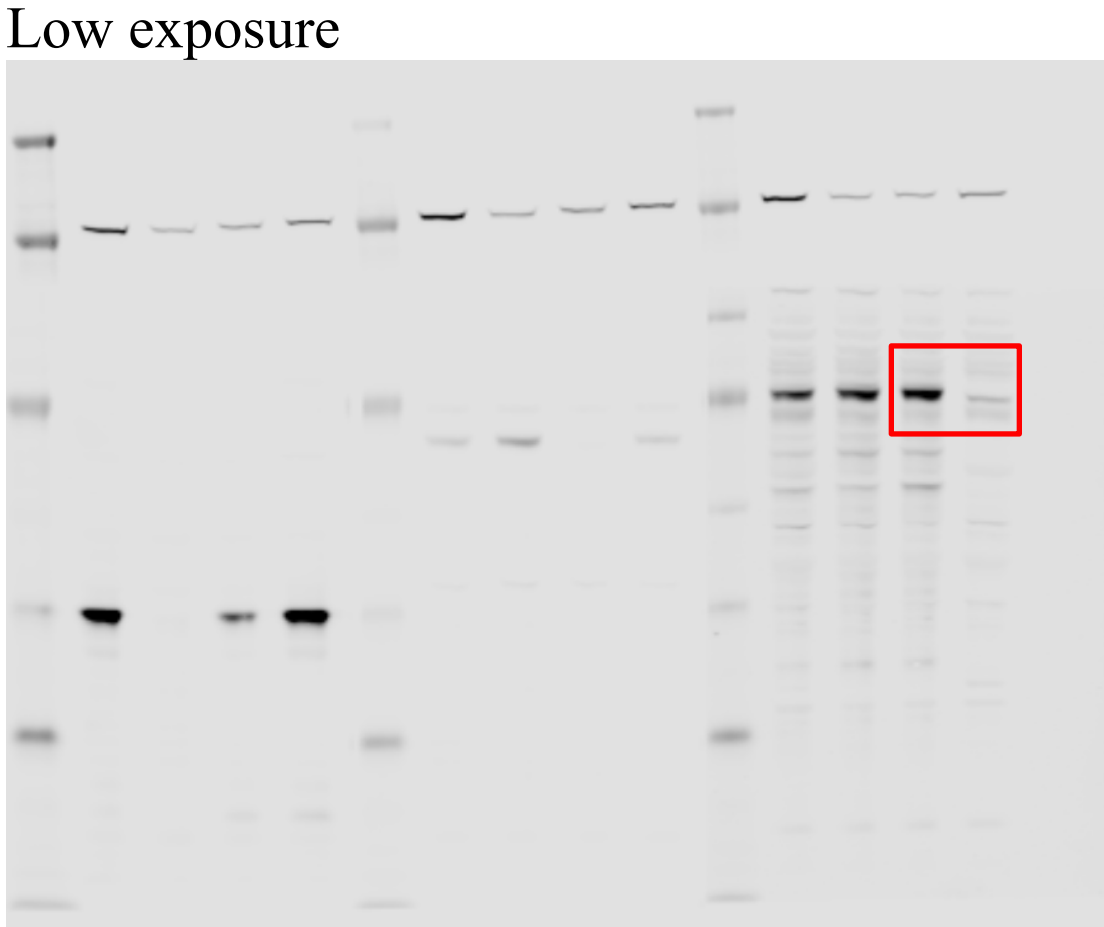

Green channel (vinculin: 120 kDa)

High exposure

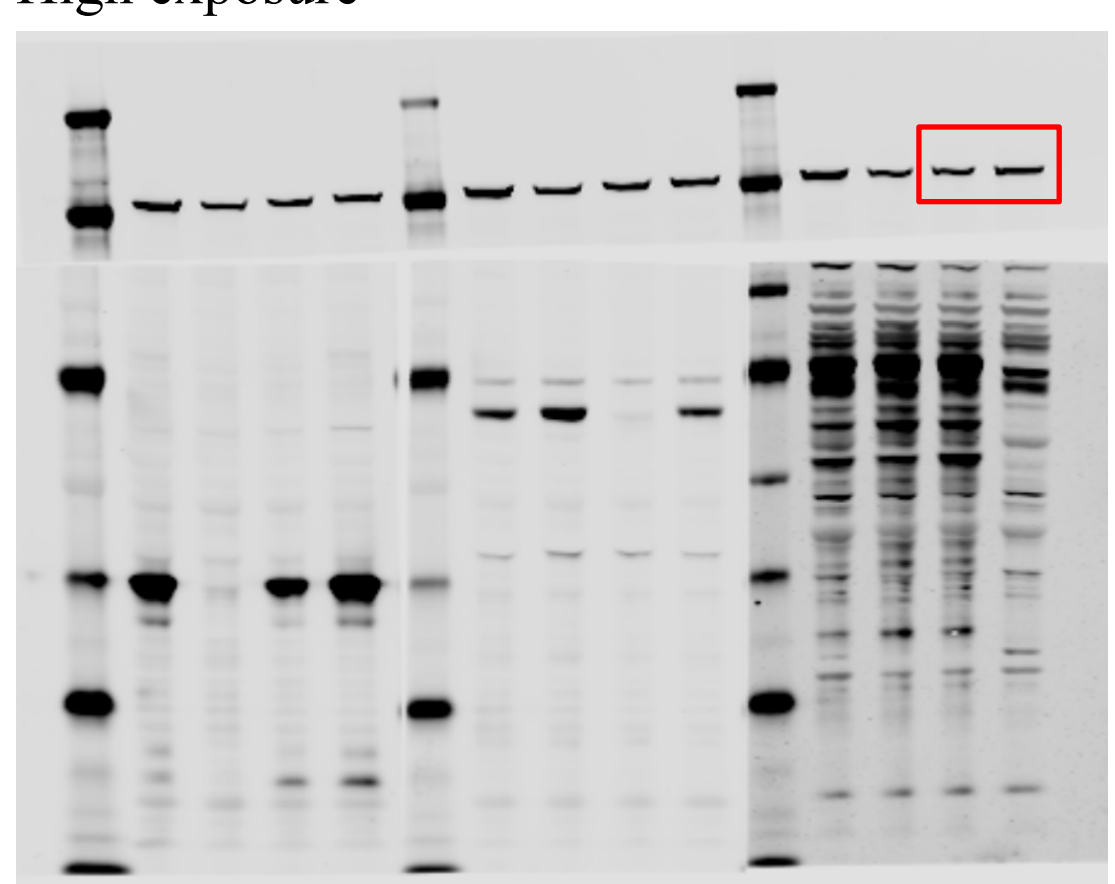

Green channel (vinculin: 120 kDa)

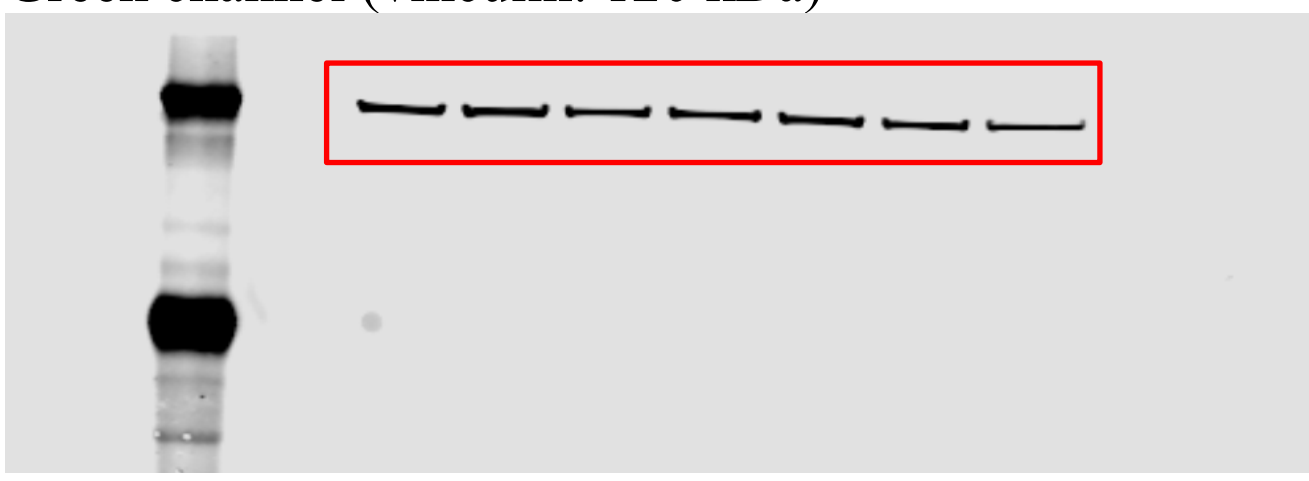

Red channel (ATRX: 280 kDa)

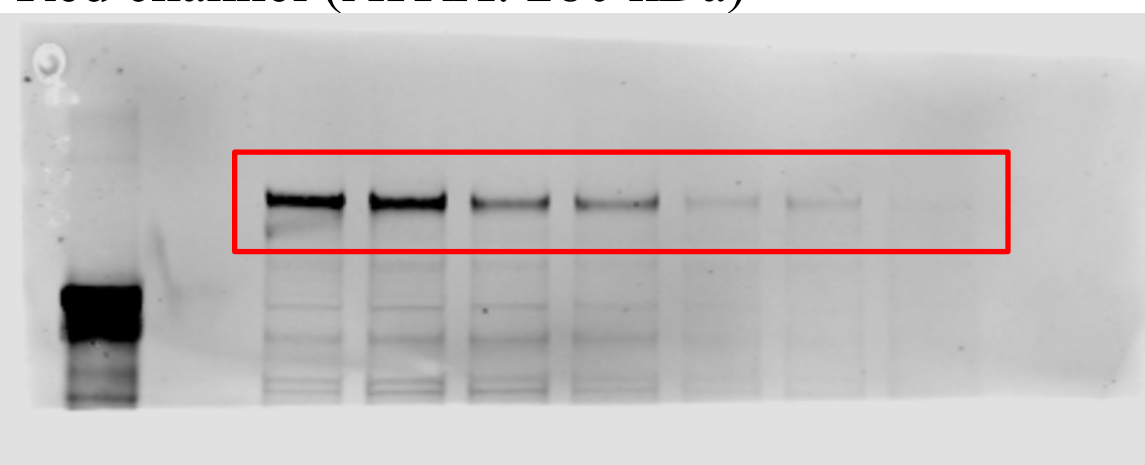

Figure S5e.

Green channel (cGAS: 62 kDa, H3: 17 kDa)

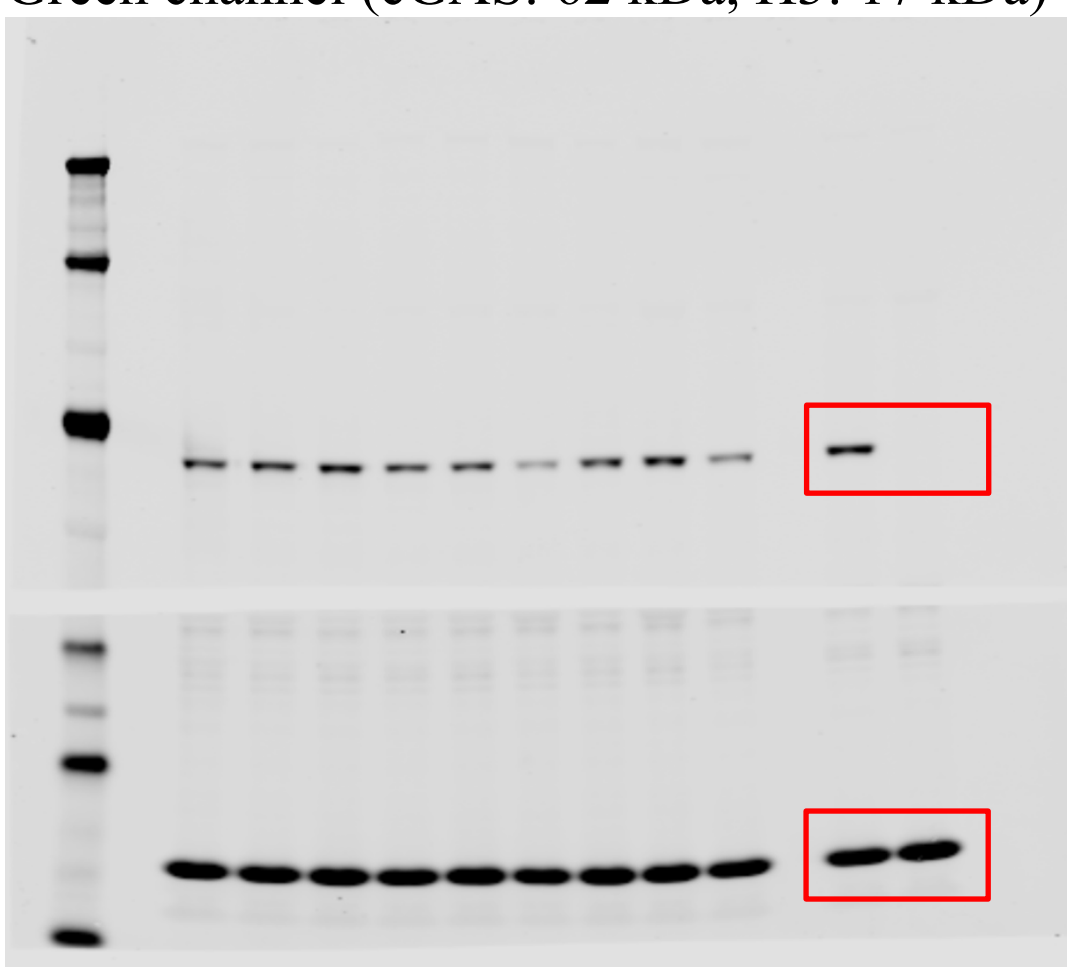

Red channel (ATRX: 280 kDa)

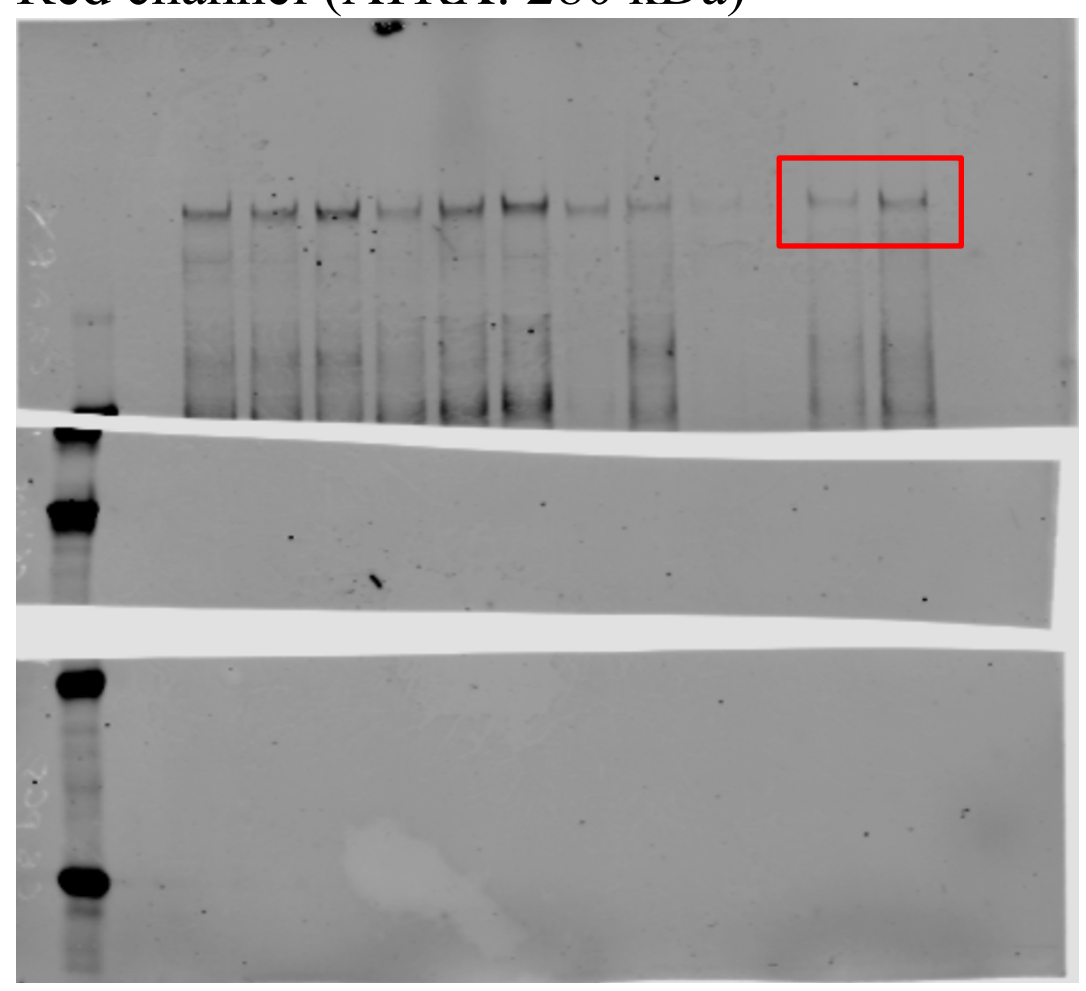

Figure S6c.

Green channel (cGAS: 62 kDa, high exposure)
exposure)

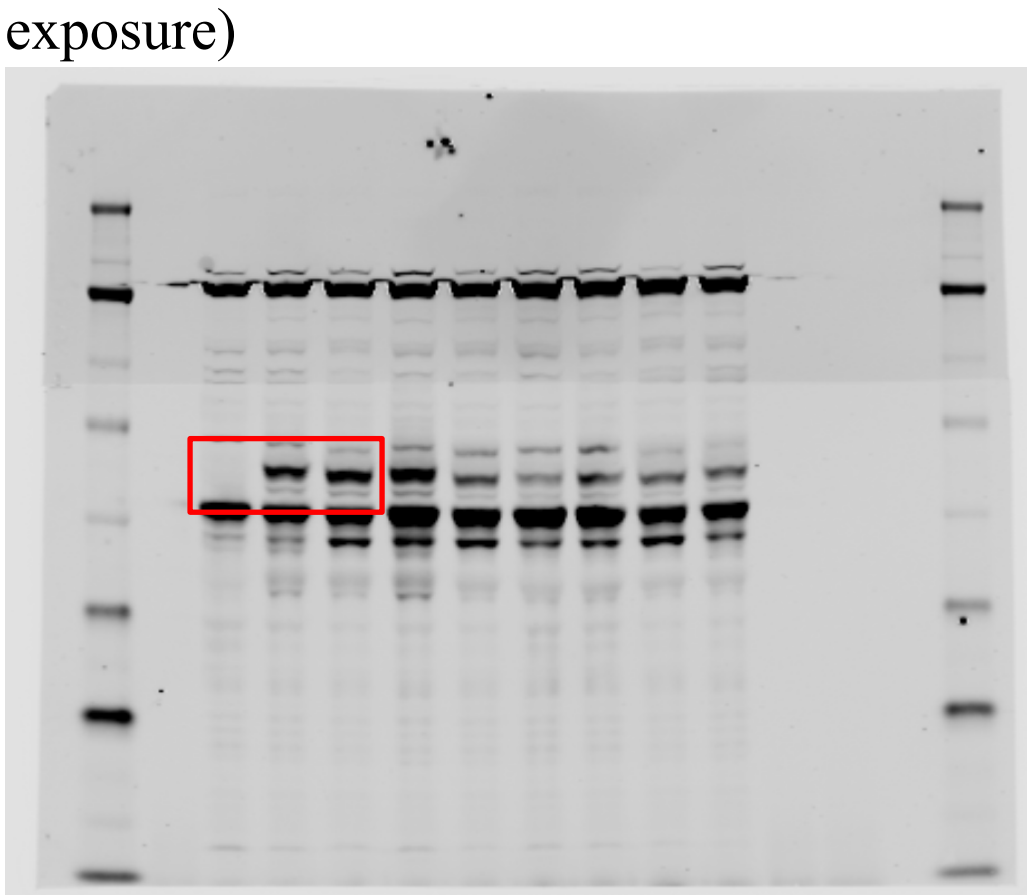

Green channel (vinculin: 120 kDa, low

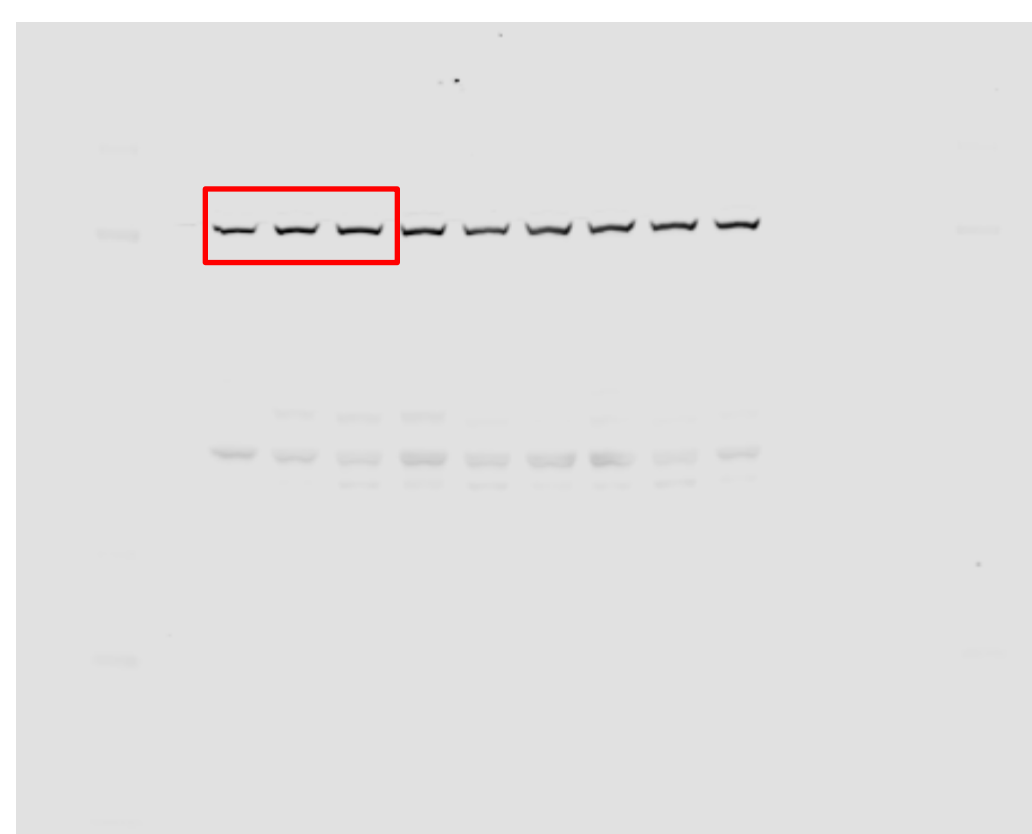

Figure S6e.

Green channel

vinculin: 120 kDa, STING 38 kDa, low exposure

cGAS: 62 kDa, high exposure

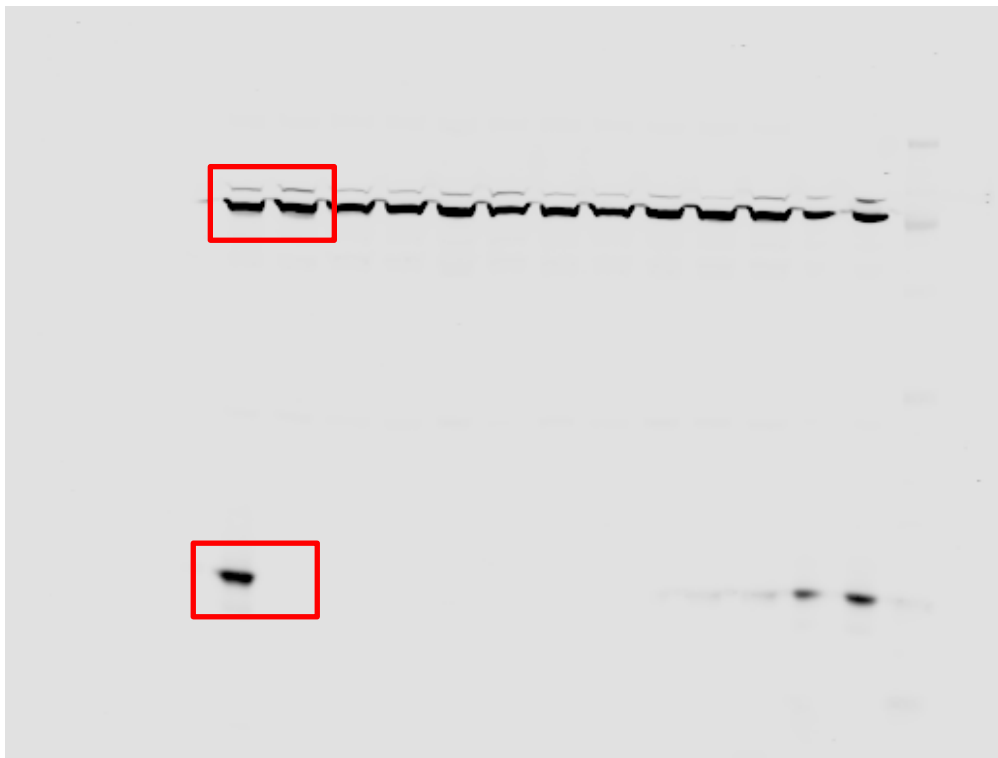

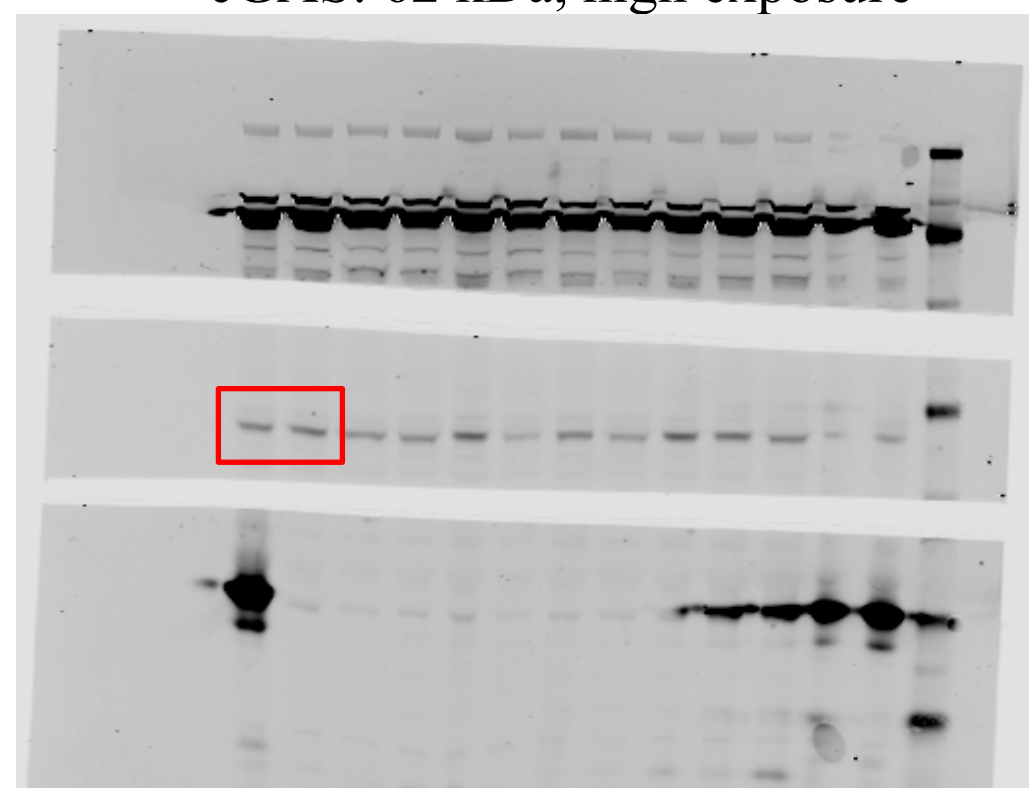

Figure S6j.

IRF3 WT

IRF3 KO

Green channel, vinculin: 120 kDa, IRF3 50 kDa

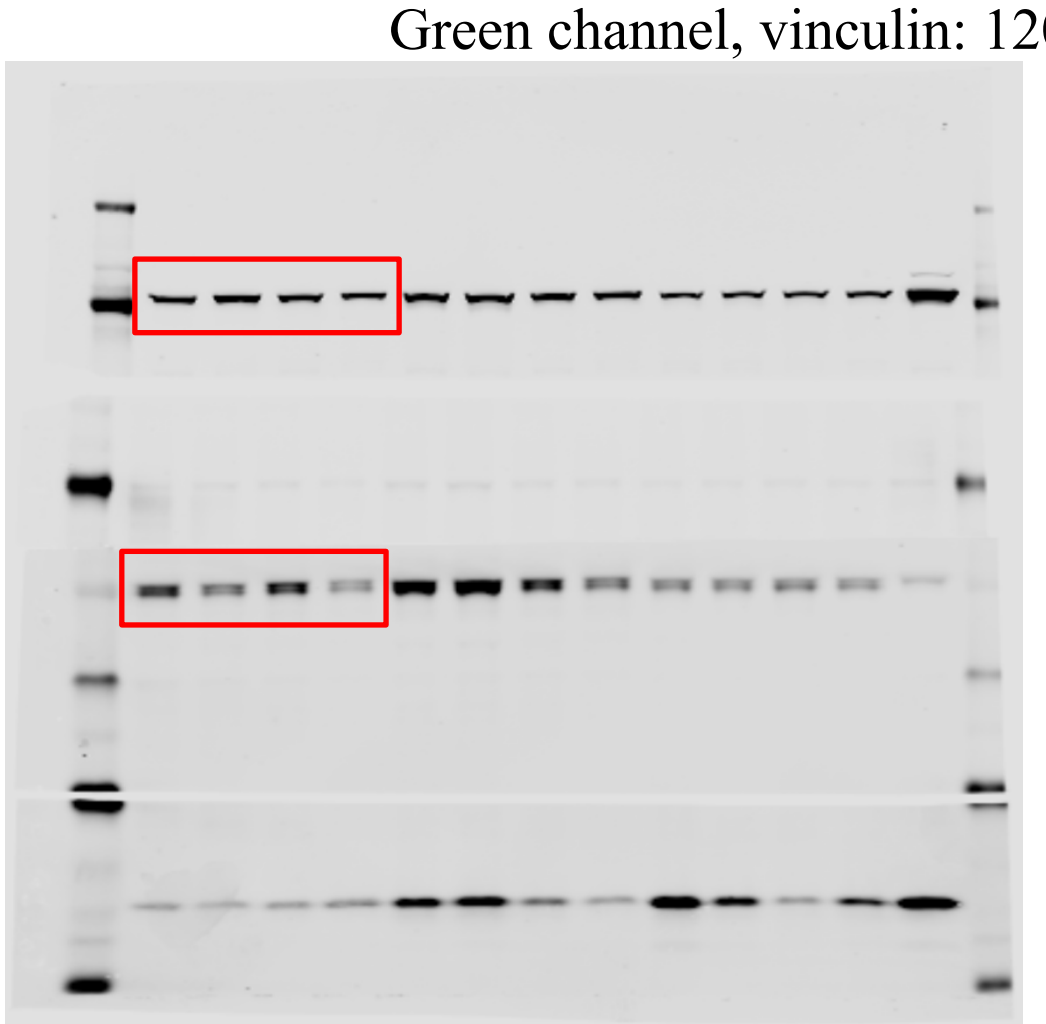

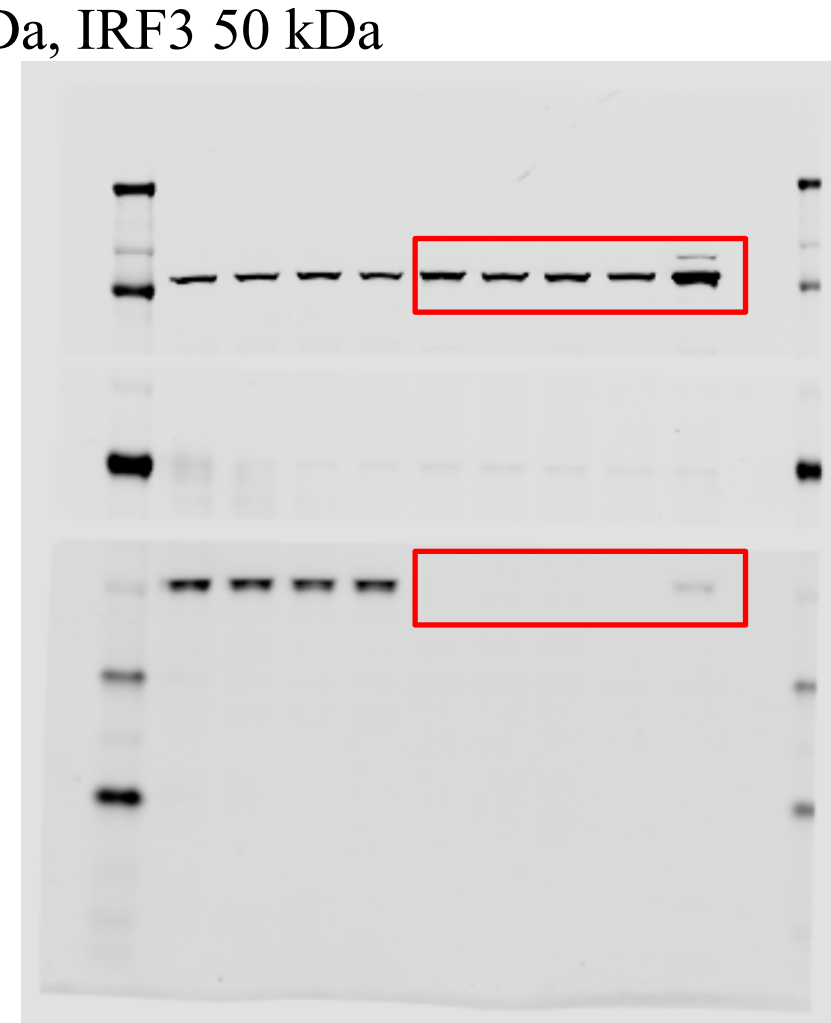

Green channel, P-STING: 40 kDa

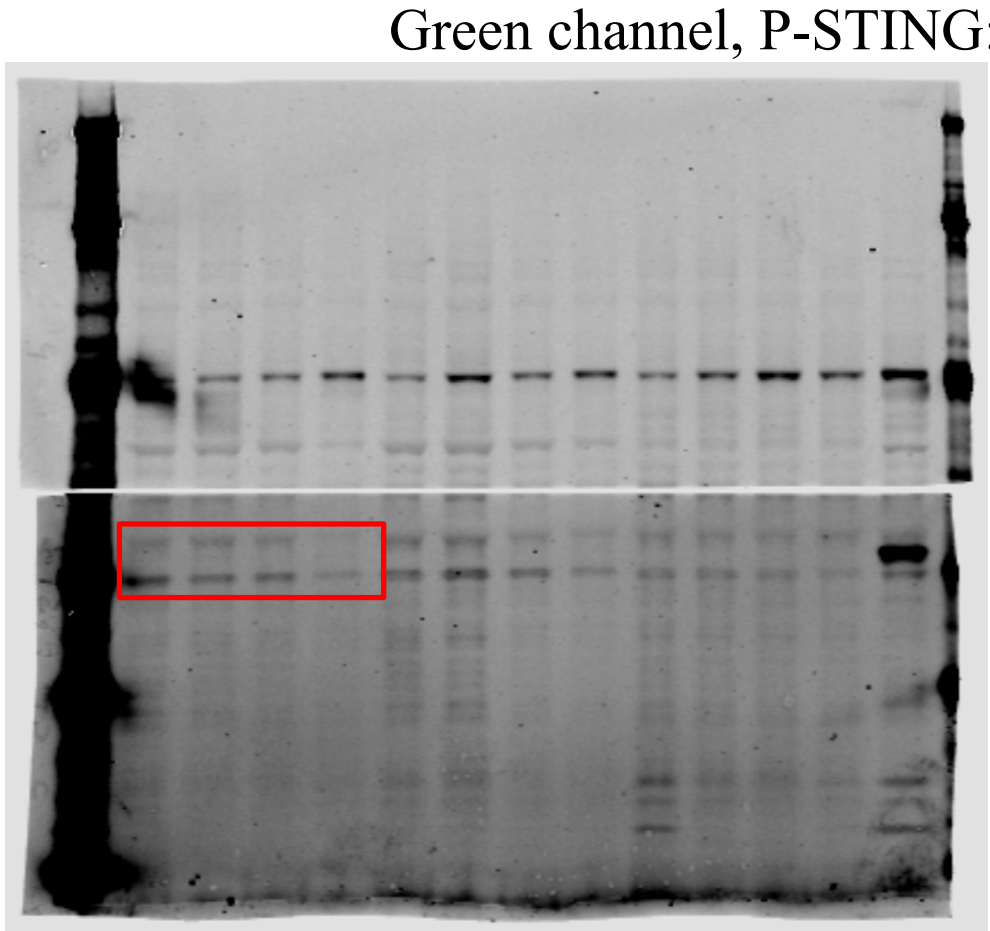

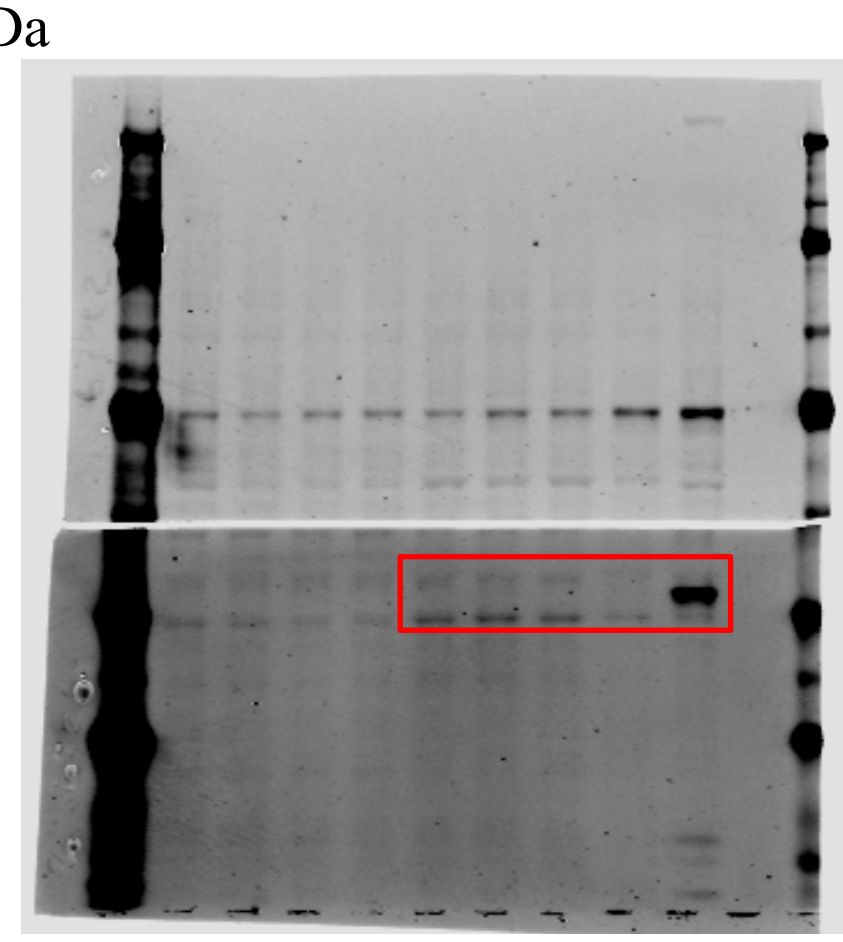

Red channel, STING: 40 kDa

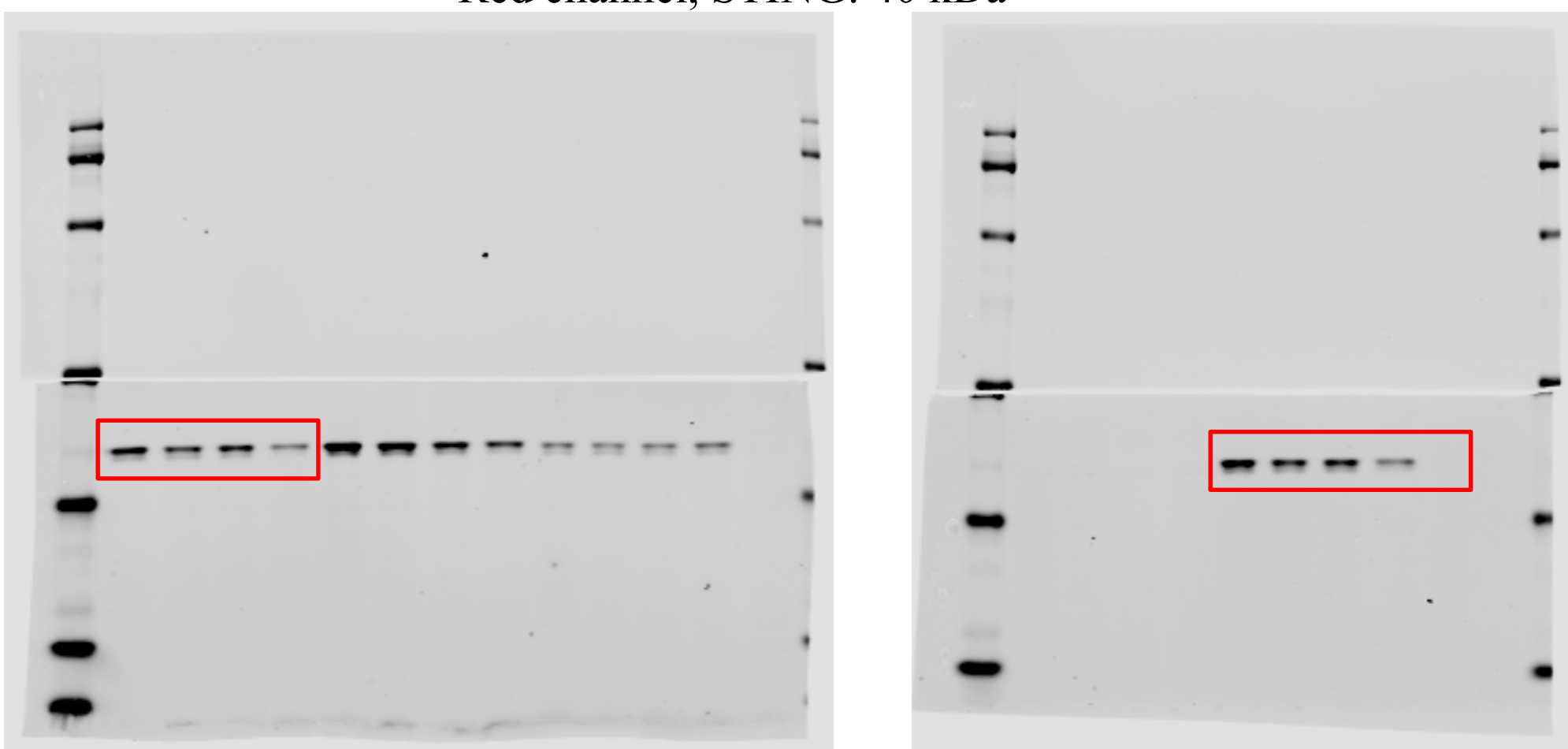

Green channel, P-STAT1: 90 kDa

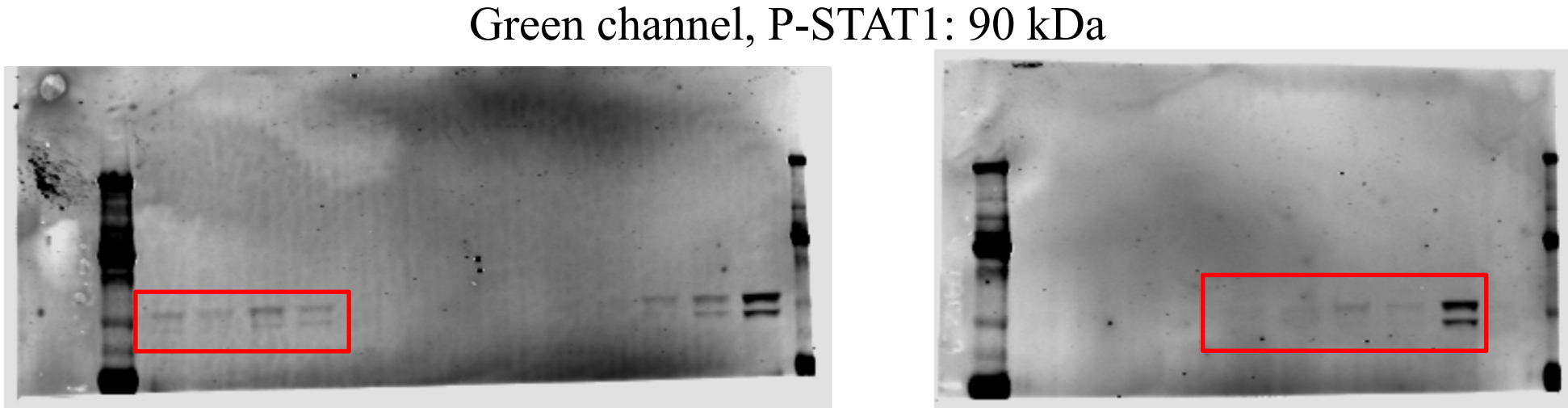

Green channel, P-IRF3: 50 kDa

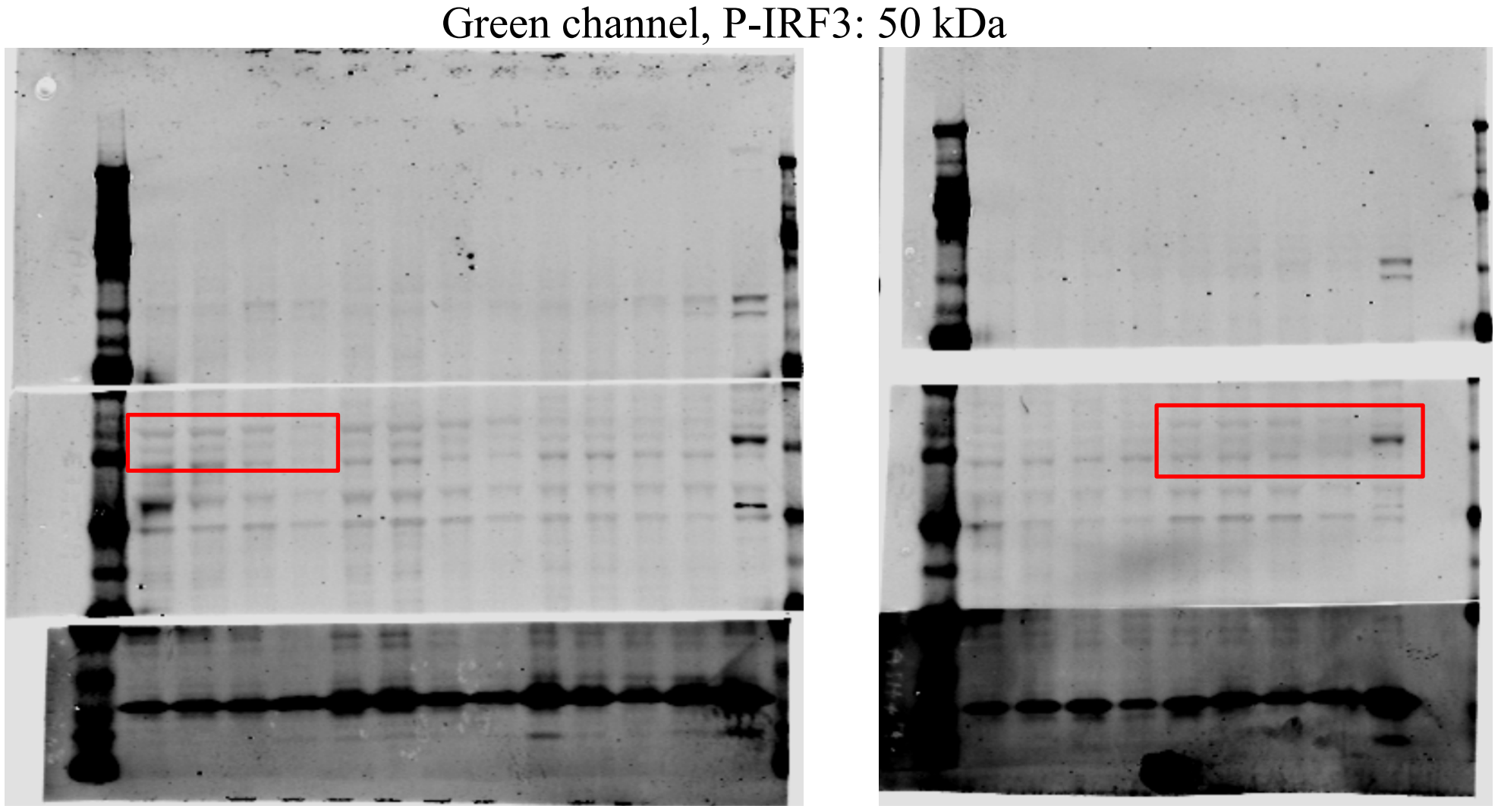

Fig. S7a

cGAS KO ATRX WT | cGAS KO ATRX KI

Red channel (ATRX: 280 kDa)

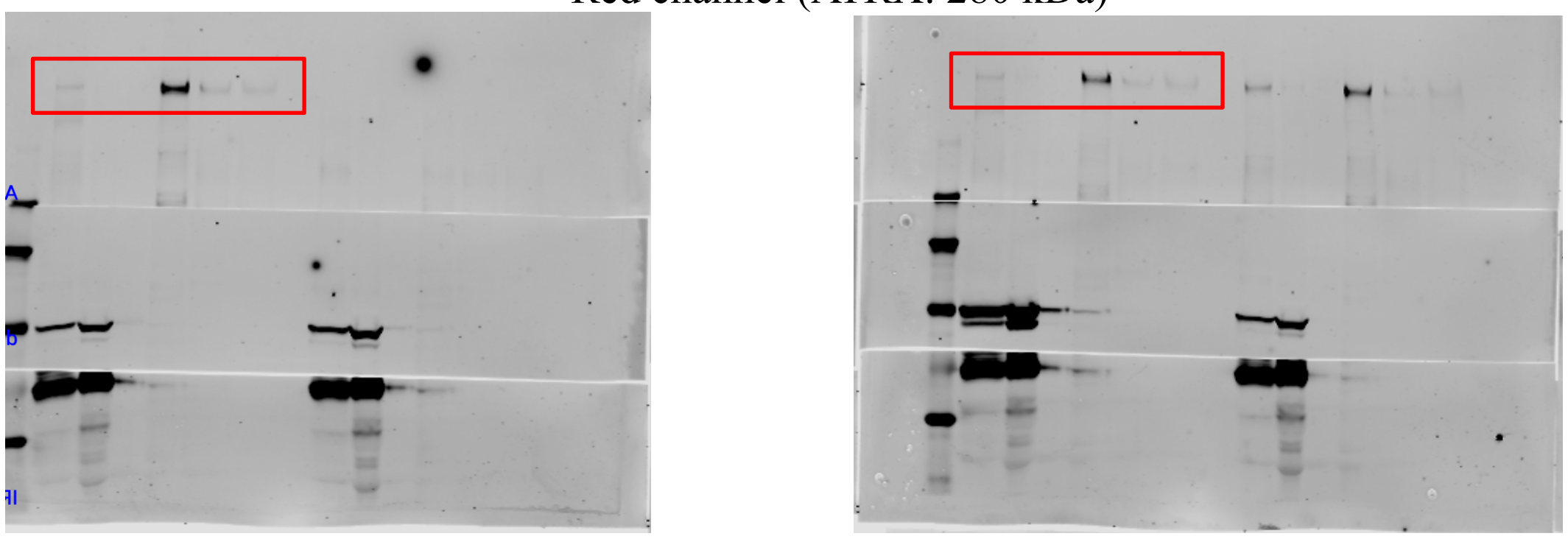

Red channel (H3: 17 kDa)

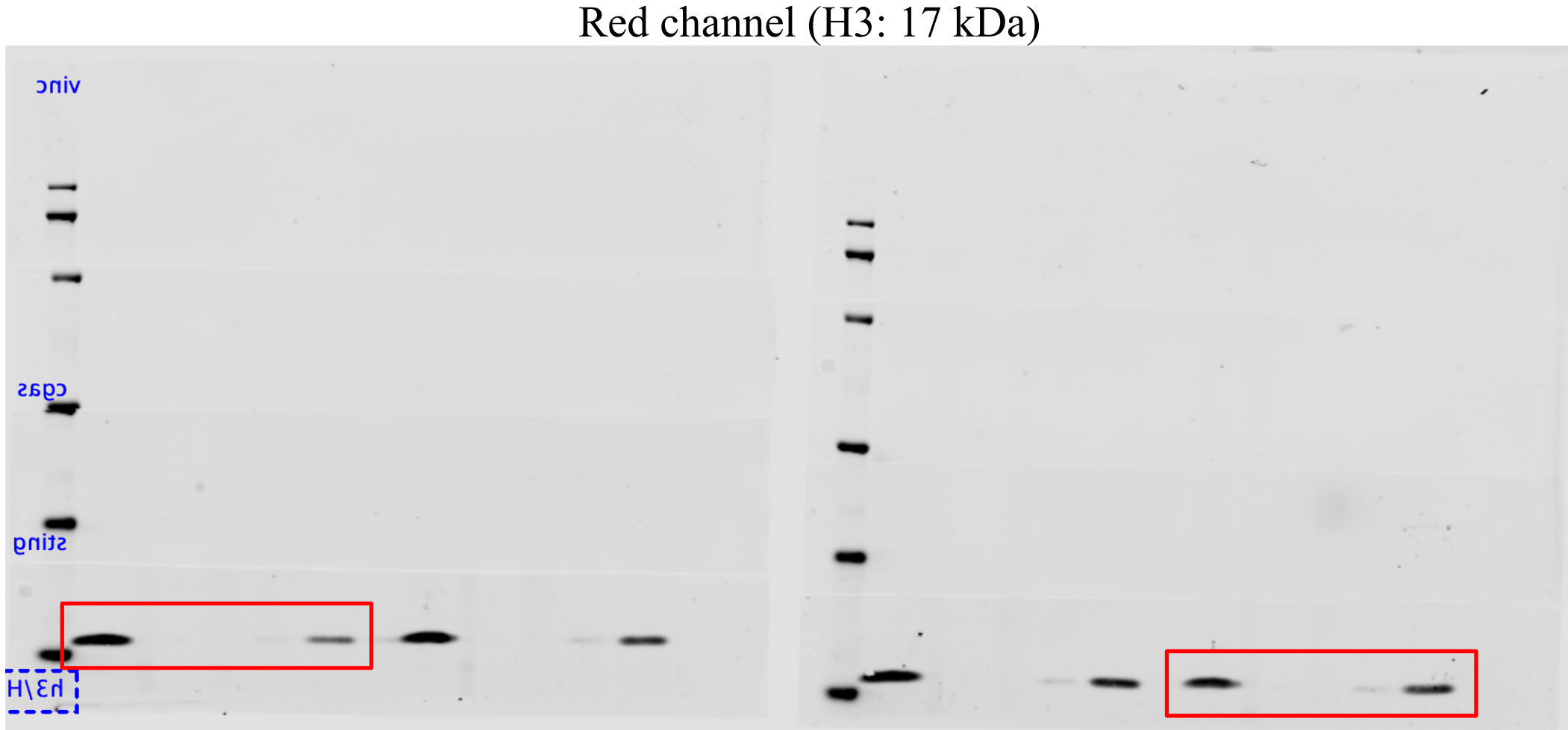

Greev channel (vinculin: 120 kDa)

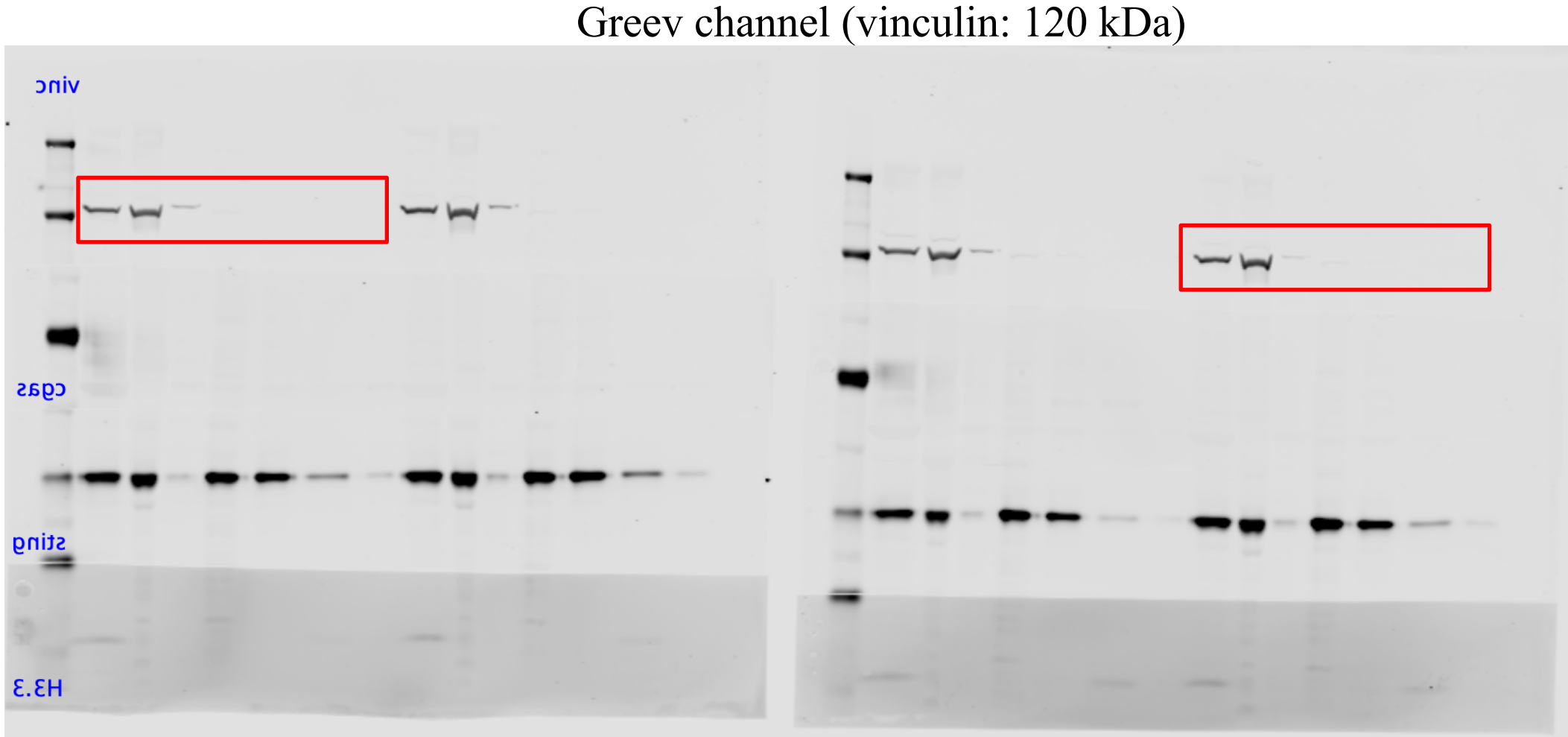

REFERENCES

1. Deneault, E. *et al.* Complete Disruption of Autism-Susceptibility Genes by Gene Editing Predominantly Reduces Functional Connectivity of Isogenic Human Neurons. *Stem Cell Reports* **11**, 1211-1225 (2018).

2. Levy, M.A., Fernandes, A.D., Tremblay, D.C., Seah, C. & Berube, N.G. The SWI/SNF protein ATRX co-regulates pseudoautosomal genes that have translocated to autosomes in the mouse genome. *BMC Genomics* **9**, 468 (2008).

3. Danussi, C. *et al.* Atrx inactivation drives disease-defining phenotypes in glioma cells of origin through global epigenomic remodeling. *Nat Commun* **9**, 1057 (2018).

SUPPLEMENTARY NOTES

- Full DNA sequence of the ATRX-Yeast plasmid generated in this study: ATRX_Y_Thermo (13889 bp), 1/23 to 23/23
- DNA sequence of the TFO-containing plasmid generated in this study: 25AEECQD_4160830_TFObindingsite_annotatedbyAF

ATRX_Y_Thermo (13889 bp)

Esp3I Esp3I
BsmBI BsmBI

TCGCGCGTTTCGGTGATGACGGTGAAAACCTCTGACACATGCAGCTCCCGGAGACGGTCACAGCTTGTCTGTAAGCGGATGCCGGGAGCAGACAAGCCCGTCAGGGC
AGCGCGCAAAGCCACTACTGCCACTTTTGGAGACTGTGTACGTCGAGGGCCTCTGCCAGTGTCGAACAGACATTCGCCTACGGCCCTCGTCTGTTCGGGCAGTCCCG

10 20 30 40 50 60 70 80 90 100

Tth111I
PflFI

GCGTCAGCGGGTGTTGGCGGGTGTCGGGGCTGGCTTAACTATGCGGCATCAGAGCAGATTGTACTGAGAGTGCACCATATCGACTACGTCGTAAGGCCGTTTCTGAC
CGCAGTCGCCCACAACCGCCCACAGCCCCGACCGAATTGATACGCCGTAGTCTCGTCTAACATGACTCTCACGTGGTATAGCTGATGCAGCATTCCGGCAAAGACTG
LEU

110 120 130 140 150 160 170 180 190 200 210

AGAGTAAAATTCTTGAGGGAACTTTCACCATTATGGGAAATGGTTCAAGAAGGTATTGACTTAAACTCCATCAAATGGTCAGGTCATTGAGTGTTTTTTATTTGTTG
TCTCATTTTAAGAACTCCCTTGAAAGTGGTAATACCCTTTACCAAGTTCTTCCATAACTGAATTTGAGGTAGTTTACCAGTCCAGTAACTCACAAAAAATAAACAAC
LEU

220 230 240 250 260 270 280 290 300 310 320

TATTTTTTTTTTTTTAGAGAAAATCCTCCAATATCAAATTAGGAATCGTAGTTTCATGATTTTCTGTTACACCTAACTTTTTGTGTGGTGCCCTCCTCCTTGTCAAT
ATAAAAAAAAAAAAATCTCTTTTAGGAGGTTATAGTTTAATCCTTAGCATCAAAGTACTAAAAGACAATGTGGATTGAAAAACACACCACGGGAGGAGGAACAGTTA
LEU

330 340 350 360 370 380 390 400 410 420

ATTAATGTTAAAGTGCAATTCTTTTTCCTTATCACGTTGAGCCATTAGTATCAATTTGCTTACCTGTATTCCTTTACTATCCTCCTTTTTCTCCTTCTTGATAAATG
TAATTACAATTTCACGTTAAGAAAAAGGAATAGTGCAACTCGGTAATCATAGTTAAACGAATGGACATAAGGAAATGATAGGAGGAAAAAGAGGAAGAACTATTTAC
LEU

430 440 450 460 470 480 490 500 510 520 530

TATGTAGATTGCGTATATAGTTTCGTCTACCCTATGAACATATTCCATTTTGTAATTTCGTGTCGTTTCTATTATGAATTTCATTTATAAAGTTTATGTACAAATAT
ATACATCTAACGCATATATCAAAGCAGATGGGATACTTGTATAAGGTAAAACATTAAAGCACAGCAAAGATAATACTTAAAGTAAATATTTCAAATACATGTTTATA
LEU

540 550 560 570 580 590 600 610 620 630 640

CATAAAAAAAGAGAATCTTTTTAAGCAAGGATTTTCTTAACTTCTTCGGCGACAGCATCACCGACTTCGGTGGTACTGTTGGAACCACCTAAATCACCAGTTCTGAT
GTATTTTTTTCTCTTAGAAAAATTCGTTCCTAAAAGAATTGAAGAAGCCGCTGTCGTAGTGGCTGAAGCCACCATGACAACCTTGGTGGATTTAGTGGTCAAGACTA
LEU

650 660 670 680 690 700 710 720 730 740

BspMI
BfuAI

ACCTGCATCCAAAACCTTTTTAACTGCATCTTCAATGGCCTTACCTTCTTCAGGCAAGTTCAATGACAATTTCAACATCATTGCAGCAGACAAGATAGTGGCGATAG
TGGACGTAGGTTTTGGAAAAATTGACGTAGAAGTTACCGGAATGGAAGAAGTCCGTTCAAGTTACTGTTAAAGTTGTAGTAACGTCGTCTGTTCTATCACCGCTATC

LEU

750 760 770 780 790 800 810 820 830 840 850

BtgI

GGTCAACCTTATTCTTTGGCAAATCTGGAGCAGAACCGTGGCATGGTTCGTACAAACCAAATGCGGTGTTCTTGTCTGGCAAAGAGGCCAAGGACGCAGATGGCAAC
CCAGTTGGAATAAGAAACCGTTTAGACCTCGTCTTGGCACCGTACCAAGCATGTTTGGTTTACGCCACAAGAACAGACCGTTTCTCCGGTTCCTGCGTCTACCGTTG

LEU

860 870 880 890 900 910 920 930 940 950 960

AAACCCAAGGAACCTGGGATAACGGAGGCTTCATCGGAGATGATATCACCAAACATGTTGCTGGTGATTATAATACCATTTAGGTGGGTTGGGTTCTTAACTAGGAT
TTTGGGTTCCTTGGACCCTATTGCCTCCGAAGTAGCCTCTACTATAGTGGTTTGTACAACGACCACTAATATTATGGTAAATCCACCCAACCCAAGAATTGATCCTA

LEU

970 980 990 1,000 1,010 1,020 1,030 1,040 1,050 1,060 1,070

CATGGCGGCAGAATCAATCAATTGATGTTGAACCTTCAATGTAGGGAATTCGTTCTTGATGGTTTCCTCCACAGTTTTTCTCCATAATCTTGAAGAGGCCAAAACAT
GTACCGCCGTCTTAGTTAGTTAACTACAACTTGGAAGTTACATCCCTTAAGCAAGAACTACCAAAGGAGGTGTCAAAAAGAGGTATTAGAACTTCTCCGGTTTTGTA

LEU

1,080 1,090 1,100 1,110 1,120 1,130 1,140 1,150 1,160 1,170

XcmI

TAGCTTTATCCAAGGACCAAATAGGCAATGGTGGCTCATGTTGTAGGGCCATGAAAGCGGCCATTCTTGTGATTCTTTGCACTTCTGGAACGGTGTATTGTTCACTA
ATCGAAATAGGTTCCTGGTTTATCCGTTACCACCGAGTACAACATCCCGGTACTTTCGCCGGTAAGAACACTAAGAAACGTGAAGACCTTGCCACATAACAAGTGAT

LEU

1,180 1,190 1,200 1,210 1,220 1,230 1,240 1,250 1,260 1,270 1,280

TCCCAAGCGACACCATCACCATCGTCTTCCTTTCTCTTACCAAAGTAAATACCTCCCACTAATTCTCTGACAACAACGAAGTCAGTACCTTTAGCAAATTGTGGCTT
AGGGTTCGCTGTGGTAGTGGTAGCAGAAGGAAAGAGAATGGTTTCATTTATGGAGGGTGATTAAGAGACTGTTGTTGCTTCAGTCATGGAAATCGTTTAACACCGAA

LEU

1,290 1,300 1,310 1,320 1,330 1,340 1,350 1,360 1,370 1,380 1,390

AflII

GATTGGAGATAAGTCTAAAAGAGAGTCGGATGCAAAGTTACATGGTCTTAAGTTGGCGTACAATTGAAGTTCTTTACGGATTTTTAGTAAACCTTGTTCAGGTCTAA
CTAACCTCTATTCAGATTTTCTCTCAGCCTACGTTTCAATGTACCAGAATTCAACCGCATGTTAACTTCAAGAAATGCCTAAAAATCATTTGGAACAAGTCCAGATT

LEU

1,400 1,410 1,420 1,430 1,440 1,450 1,460 1,470 1,480 1,490

KpnI
Acc65I

CACTACCGGTACCCCATTTAGGACCACCCACAGCACCTAACAAAACGGCATCAACCTTCTTGGAGGCTTCCAGCGCCTCATCTGGAAGTGGGACACCTGTAGCATCG
GTGATGGCCATGGGGTAAATCCTGGTGGGTGTCGTGGATTGTTTTGCCGTAGTTGGAAGAACCTCCGAAGGTCGCGGAGTAGACCTTCACCCTGTGGACATCGTAGC
LEU
1,500 1,510 1,520 1,530 1,540 1,550 1,560 1,570 1,580 1,590 1,600

ATAGCAGCACCACCAATTAAATGATTTTCGAAATCGAACTTGACATTGGAACGAACATCAGAAATAGCTTTAAGAACCTTAATGGCTTCGGCTGTGATTTCTTGACC
TATCGTCGTGGTGGTTAATTTACTAAAAGCTTTAGCTTGAACTGTAACCTTGCTTGTAGTCTTTATCGAAATTCTTGGAATTACCGAAGCCGACACTAAAGAACTGG
LEU
1,610 1,620 1,630 1,640 1,650 1,660 1,670 1,680 1,690 1,700 1,710

AACGTGGTCACCTGGCAAAACGACGATCTTCTTAGGGGCAGACATAGGGGCAGACATTAGAATGGTATATCCTTGAAATATATATATATATATTGCTGAAATGTAAAAG
TTGCACCAGTGGACCGTTTTGCTGCTAGAAGAATCCCCGTCTGTATCCCCGTCTGTAATCTTACCATATAGGAACTTTATATATATATATATAACGACTTTACATTTTC
LEU
1,720 1,730 1,740 1,750 1,760 1,770 1,780 1,790 1,800 1,810

PpuMI

GTAAGAAAAGTTAGAAAGTAAGACGATTGCTAACCACCTATTGGAAAAAACAATAGGTCCTTAAATAATATTGTCAACTTCAAGTATTGTGATGCAAGCATTTAGTC
CATTCTTTTCAATCTTTCATTCTGCTAACGATTGGTGGATAACCTTTTTTGTTATCCAGGAATTTATTATAACAGTTGAAGTTCATAACACTACGTTCGTAAATCAG
LEU
1,820 1,830 1,840 1,850 1,860 1,870 1,880 1,890 1,900 1,910 1,920

ATGAACGCTTCTCTATTCTATATGAAAAGCCGGTTCCGGCCTCTCACCTTTCCTTTTTCTCCCAATTTTTCAGTTGAAAAAGGTATATGCGTCAGGCGACCTCTGAA
TACTTGCGAAGAGATAAGATATACTTTTCGGCCAAGGCCGGAGAGTGGAAAGGAAAAAGAGGGTTAAAAAGTCAACTTTTTCCATATACGCAGTCCGCTGGAGACTT
LEU
1,930 1,940 1,950 1,960 1,970 1,980 1,990 2,000 2,010 2,020 2,030

ATTAACAAAAAATTTCCAGTCATCGAATTTGATTCTGTGCGATAGCGCCCCTGTGTGTTCTCGTTATGTTGAGGAAAAAAATAATGGTTGCTAAGAGATTCGAACTC
TAATTGTTTTTTAAAGGTCAGTAGCTTAAACTAAGACACGCTATCGCGGGGACACACAAGAGCAATACAACTCCTTTTTTTATTACCAACGATTCTCTAAGCTTGAG
LEU
2,040 2,050 2,060 2,070 2,080 2,090 2,100 2,110 2,120 2,130 2,140

PluTI
SfoI
NarI
BaeI KasI

TTGCATCTTACGATACCTGAGTATTCCCACAGTTAACTGCGGTCAAGATATTTCTTGAATCAGGCGCCTTAGACCGCTCGGCCAAACAACCAATTACTTGTTGAGAA
AACGTAGAATGCTATGGACTCATAAGGGTGTCAATTGACGCCAGTTCTATAAAGAACTTAGTCCGCGGAATCTGGCGAGCCGGTTTGTTGGTTAATGAACAACTCTT
LEU
2,150 2,160 2,170 2,180 2,190 2,200 2,210 2,220 2,230 2,240

ATAGAGTATAATTATCCTATAAATATAACGTTTTTGAACACACATGAACAAGGAAGTACAGGACAATTGATTTTGAAGAGAATGTGGATTTTGATGTAATTGTTGGG
TATCTCATATTAATAGGATATTTATATTGCAAAAACTTGTGTGTACTTGTTCCTTCATGTCCTGTTAACTAAAACTTCTCTTACACCTAAAACTACATTAACAACCC

LEU

2,250 2,260 2,270 2,280 2,290 2,300 2,310 2,320 2,330 2,340 2,350

ATTCCATTTTTAATAAGGCAATAATATTAGGTATGTGGATATACTAGAAGTTCTCCTCGACCGTCGATATGCGGTGTGAAATACCGCACAGATGCGTAAGGAGAAAA
TAAGGTAAAAATTATTCCGTTATTATAATCCATACACCTATATGATCTTCAAGAGGAGCTGGCAGCTATACGCCACACTTTATGGCGTGTCTACGCATTCCTCTTTT

LEU

2,360 2,370 2,380 2,390 2,400 2,410 2,420 2,430 2,440 2,450 2,460

TACCGCATCAGGAAATTGTAAACGTTAATATTTTGTTAAAATTCGCGTTAAATTTTTGTTAAATCAGCTCATTTTTTAACCAATAGGCCGAAATCGGCAAAATCCCT
ATGGCGTAGTCCTTTAACATTTGCAATTATAAAACAATTTTAAGCGCAATTTAAAAACAATTTAGTCGAGTAAAAAATTGGTTATCCGGCTTTAGCCGTTTTAGGGA

2,470 2,480 2,490 2,500 2,510 2,520 2,530 2,540 2,550 2,560

AloI

TATAAATCAAAAGAATAGACCGAGATAGGGTTGAGTGTTGTTCCAGTTTGGAACAAGAGTCCACTATTAAAGAACGTGGACTCCAACGTCAAAGGGCGAAAAACCGT
ATATTTAGTTTTCTTATCTGGCTCTATCCCAACTCACAACAAGGTCAAACCTTGTTCTCAGGTGATAATTTCTTGCACCTGAGGTTGCAGTTTCCCGCTTTTTGGCA

f1_origin

2,570 2,580 2,590 2,600 2,610 2,620 2,630 2,640 2,650 2,660 2,670

BtgZI
DraIII
BsaAI

CTATCAGGGCGATGGCCCACTACGTGAACCATCACCCTAATCAAGTTTTTTGGGGTCGAGGTGCCGTAAAGCACTAAATCGGAACCCTAAAGGGAGCCCCCGATTTA
GATAGTCCCGCTACCGGGTGATGCACTTGGTAGTGGGATTAGTTCAAAAAACCCCAGCTCCACGGCATTTCGTGATTTAGCCTTGGGATTTCCCTCGGGGGCTAAAT

f1_origin

2,680 2,690 2,700 2,710 2,720 2,730 2,740 2,750 2,760 2,770 2,780

NaeI
NgoMIV

GAGCTTGACGGGGAAAGCCGGCGAACGTGGCGAGAAAGGAAGGGAAGAAAGCGAAAGGAGCGGGCGCTAGGGCGCTGGCAAGTGTAGCGGTCACGCTGCGCGTAACC
CTCGAACTGCCCCTTTCGGCCGCTTGCACCGCTCTTTCCTTCCCTTCTTTCGCTTTCCTCGCCCGCGATCCCGCGACCGTTCACATCGCCAGTGCGACGCGCATTGG

f1_origin

2,790 2,800 2,810 2,820 2,830 2,840 2,850 2,860 2,870 2,880

FspI PvuI

ACCACACCCGCCGCGCTTAATGCGCCGCTACAGGGCGCGTCGCGCCATTCGCCATTCAGGCTGCGCAACTGTTGGGAAGGGCGATCGGTGCGGGCCTCTTCGCTATT
TGGTGTGGGCGGCGCGAATTACGCGGCGATGTCCCGCGCAGCGCGGTAAGCGGTAAGTCCGACGCGTTGACAACCCTTCCCGCTAGCCACGCCCGGAGAAGCGATAA

f1_origin lacZ_a

2,890 2,900 2,910 2,920 2,930 2,940 2,950 2,960 2,970 2,980 2,990

ACGCCAGCTGGCGAAGGGGGGATGTGCTGCAAGGCGATTAAGTTGGGTAACGCCAGGGTTTTCCCAGTCACGACGTTGTAAAACGACGGCCAGTGAATTGTAATACG
TGCGGTCGACCGCTTCCCCCCTACACGACGTTCCGCTAATTCAACCCATTGCGGTCCCAAAAGGGTCAGTGCTGCAACATTTTGCTGCCGGTCACTTAACATTATGC

lacZ_a

3,000 3,010 3,020 3,030 3,040 3,050 3,060 3,070 3,080 3,090 3,100

SacI
Eco53kI
NotI
EagI

ACTCACTATAGGGCGAATTGGAGCTCCTACTACTACTACTAGCGGCCGCTTATATTGAATTTTCAAAAATTCTTACTTTTTTTTTGGATGGACGCAAAGAAGTTTAA
TGAGTGATATCCCGCTTAACCTCGAGGATGATGATGATGATCGCCGGCGAATATAACTTAAAAGTTTTTAAGAATGAAAAAAAAACCTACCTGCGTTTCTTCAAATT

T7_...er
GAL1-10

3,110 3,120 3,130 3,140 3,150 3,160 3,170 3,180 3,190 3,200 3,210

TAATCATATTACATGGCATTACCACCATATACATATCCATATCTAATCTTACTTATATGTTGTGGAAATGTAAAGAGCCCCATTATCTTAGCCTAAAAAAACCTTCT
ATTAGTATAATGTACCGTAATGGTGGTATATGTATAGGTATAGATTAGAATGAATATACAACACCTTTACATTTCTCGGGGTAATAGAATCGGATTTTTTTGGAAGA

GAL1-10

3,220 3,230 3,240 3,250 3,260 3,270 3,280 3,290 3,300 3,310

CTTTGGAACTTTCAGTAATACGCTTAACTGCTCATTGCTATATTGAAGTACGGATTAGAAGCCGCCGAGCGGGCGACAGCCCTCCGACGGAAGACTCTCCTCCGTGC
GAAACCTTGAAAGTCATTATGCGAATTGACGAGTAACGATATAACTTCATGCCTAATCTTCGGCGGCTCGCCCGCTGTCGGGAGGCTGCCTTCTGAGAGGAGGCACG

GAL1-10

3,320 3,330 3,340 3,350 3,360 3,370 3,380 3,390 3,400 3,410 3,420

GTCCTCGTCTTCACCGGTCGCGTTCCTGAAACGCAGATGTGCCTCGCGCCGCACTGCTCCGAACAATAAAGATTCTACAATACTAGCTTTTATGGTTATGAAGAGGA
CAGGAGCAGAAGTGGCCAGCGCAAGGACTTTGCGTCTACACGGAGCGCGGCGTGACGAGGCTTGTTATTTCTAAGATGTTATGATCGAAAATACCAATACTTCTCCT

GAL1-10

3,430 3,440 3,450 3,460 3,470 3,480 3,490 3,500 3,510 3,520 3,530

AAAATTGGCAGTAACCTGGCCCCACAAACCTTCAAATTAACGAATCAAATTAACAACCATAGGATGATAATGCGATTAGTTTTTTAGCCTTATTTCTGGGGTAATTA
TTTTAACCGTCATTGGACCGGGGTGTTTGGAAGTTTAATTGCTTAGTTTAATTGTTGGTATCCTACTATTACGCTAATCAAAAAATCGGAATAAAGACCCCATTAAT

GAL1-10

3,540 3,550 3,560 3,570 3,580 3,590 3,600 3,610 3,620 3,630

BtgZI

ATCAGCGAAGCGATGATTTTTGATCTATTAACAGATATATAAATGGAAAAGCTGCATAACCACTTTAACTAATACTTTCAACATTTTCAGTTTGTATTACTTCTTAT
TAGTCGCTTCGCTACTAAAAACTAGATAATTGTCTATATATTTACCTTTTCGACGTATTGGTGAAATTGATTATGAAAGTTGTAAAAGTCAAACATAATGAAGAATA

GAL1-10

3,640 3,650 3,660 3,670 3,680 3,690 3,700 3,710 3,720 3,730 3,740

SpeI

TCAAATGTCATAAAAGTATCAACAAAAAATTGTTAATATACCTCTATACTTTAACGTCAAGGAGAAAAAACTATAACTAGTTATAAAACAATGACTGCTGAACCTAT
AGTTTACAGTATTTTCATAGTTGTTTTTTAACAATTATATGGAGATATGAAATTGCAGTTCCTCTTTTTTGATATTGATCAATATTTTGTTACTGACGACTTGGATA

2 4 6
M T A E P M
3836-11353
ATRX_Y_Thermo
GAL1-10

3,750 3,760 3,770 3,780 3,790 3,800 3,810 3,820 3,830 3,840 3,850

GTCCGAATCTAAGTTGAACACTTTGGTTCAAAAGCTGCATGATTTCTTGGCCCATTCATCTGAAGAATCCGAAGAAACATCTTCTCCACCAAGATTGGCTATGAATC
CAGGCTTAGATTCAACTTGTGAAACCAAGTTTTCGACGTACTAAAGAACCGGGTAAGTAGACTTCTTAGGCTTCTTTGTAGAAGAGGTGGTTCTAACCGATACTTAG

8 10 12 14 16 18 20 22 24 26 28 30 32 34 36 38 40
S E S K L N T L V Q K L H D F L A H S S E E S E E T S S P P R L A M N
3836-11353
ATRX_Y_Thermo

3,860 3,870 3,880 3,890 3,900 3,910 3,920 3,930 3,940 3,950

AAAACACCGATAAGATCTCTGGTTCCGGTTCTAATTCTGACATGATGGAAAACTCCAAAGAAGAAGGCACATCCTCCTCCGAAAAATCTAAATCTTCTGGTTCCTCC
TTTTGTGGCTATTCTAGAGACCAAGGCCAAGATTAAGACTGTACTACCTTTTGAGGTTTCTTCTTCCGTGTAGGAGGAGGCTTTTTAGATTTAGAAGACCAAGGAGG

42 44 46 48 50 52 54 56 58 60 62 64 66 68 70 72 74 76
Q N T D K I S G S G S N S D M M E N S K E E G T S S S E K S K S S G S S
3836-11353
ATRX_Y_Thermo

3,960 3,970 3,980 3,990 4,000 4,010 4,020 4,030 4,040 4,050 4,060

AGATCCAAGAGAAAACCATCTATCGTTACCAAGTACGTTGAATCCGATGACGAAAAACCATTGGATGATGAGACTGTTAACGAAGATGCTTCCAACGAAAACTCCGA
TCTAGGTTCTCTTTTGGTAGATAGCAATGGTTCATGCAACTTAGGCTACTGCTTTTTGGTAACCTACTACTCTGACAATTGCTTCTACGAAGGTTGCTTTTGAGGCT

78 80 82 84 86 88 90 92 94 96 98 100 102 104 106 108 110 112
R S K R K P S I V T K Y V E S D D E K P L D D E T V N E D A S N E N S E
3836-11353
ATRX_Y_Thermo

4,070 4,080 4,090 4,100 4,110 4,120 4,130 4,140 4,150 4,160 4,170

BstAPI BsaBI

AAACGATATTACCATGCAGTCTTTGCCAAAGGGTACTGTTATAGTTCAACCAGAACCAGTTTTGAACGAAGATAAGGATGATTTCAAGGGTCCAGAATTCAGATCCA
TTTGCTATAATGGTACGTCAGAAACGGTTTCCCATGACAATATCAAGTTGGTCTTGGTCAAAACTTGCTTCTATTCCTACTAAAGTTCCCAGGTCTTAAGTCTAGGT

114 116 118 120 122 124 126 128 130 132 134 136 138 140 142 144 146 148
N D I T M Q S L P K G T V I V Q P E P V L N E D K D D F K G P E F R S
3836-11353
ATRX_Y_Thermo

4,180 4,190 4,200 4,210 4,220 4,230 4,240 4,250 4,260 4,270 4,280

BarI

GATCTAAGATGAAGACCGAGAACTTGAAGAAAAGGGGTGAAGATGGTTTACACGGTATCGTTTCTTGTACTGCTTGTGGTCAACAAGTTAACCACTTCCAAAAGGAC
CTAGATTCTACTTCTGGCTCTTGAACTTCTTTTCCCCACTTCTACCAAATGTGCCATAGCAAAGAACATGACGAACACCAGTTGTTCAATTGGTGAAGGTTTTCCTG

150 152 154 156 158 160 162 164 166 168 170 172 174 176 178 180 182 184
R S K M K T E N L K K R G E D G L H G I V S C T A C G Q Q V N H F Q K D

3836-11353

ATRX_Y_Thermo

4,290 4,300 4,310 4,320 4,330 4,340 4,350 4,360 4,370 4,380

TCTATCTACAGACATCCATCCTTGCAAGTCTTGATCTGTAAGAACTGTTTCAAGTACTACATGTCCGACGACATTTCCAGAGATTCTGATGGTATGGATGAACAATG
AGATAGATGTCTGTAGGTAGGAACGTTCAGAACTAGACATTCTTGACAAAGTTCATGATGTACAGGCTGCTGTAAAGGTCTCTAAGACTACCATACCTACTTGTTAC

186 188 190 192 194 196 198 200 202 204 206 208 210 212 214 216 218 220
S I Y R H P S L Q V L I C K N C F K Y Y M S D D I S R D S D G M D E Q C

3836-11353

ATRX_Y_Thermo

4,390 4,400 4,410 4,420 4,430 4,440 4,450 4,460 4,470 4,480 4,490

BstAPI

TAGATGGTGTGCTGAAGGTGGTAACTTGATTTGTTGTGATTTCTGCCATAACGCTTTCTGCAAGAAGTGCATTTTGAGAAACTTGGGCCGTAAAGAATTGTCCACTA
ATCTACCACACGACTTCCACCATTGAACTAAACAACACTAAAGACGGTATTGCGAAAGACGTTCTTCACGTAAAACTCTTTGAACCCGGCATTTCTTAACAGGTGAT

222 224 226 228 230 232 234 236 238 240 242 244 246 248 250 252 254
R W C A E G G N L I C C D F C H N A F C K K C I L R N L G R K E L S T

3836-11353

ATRX_Y_Thermo

4,500 4,510 4,520 4,530 4,540 4,550 4,560 4,570 4,580 4,590 4,600

TTATGGACGAAAACAACCAGTGGTACTGCTACATTTGTCATCCAGAACCATTATTGGATTTGGTTACCGCTTGTAACTCCGTGTTCGAAAACTTGGAACAATTGCTG
AATACCTGCTTTTGTTGGTCACCATGACGATGTAAACAGTAGGTCTTGGTAATAACCTAAACCAATGGCGAACATTGAGGCACAAGCTTTTGAACCTTGTTAACGAC

256 258 260 262 264 266 268 270 272 274 276 278 280 282 284 286 288 290
I M D E N N Q W Y C Y I C H P E P L L D L V T A C N S V F E N L E Q L L

3836-11353

ATRX_Y_Thermo

4,610 4,620 4,630 4,640 4,650 4,660 4,670 4,680 4,690 4,700

CAACAGAACAAGAAGAAGATCAAGGTTGACTCTGAGAAGTCCAACAAGGTTTACGAACATACCTCTAGATTCTCCCCAAAGAAAACCTCTTCTAACTGTAATGGCGA
GTTGTCTTGTTCTTCTTCTAGTTCCAACTGAGACTCTTCAGGTTGTTCCAAATGCTTGTATGGAGATCTAAGAGGGGTTTCTTTTGGAGAAGATTGACATTACCGCT

292 294 296 298 300 302 304 306 308 310 312 314 316 318 320 322 324 326
Q Q N K K K I K V D S E K S N K V Y E H T S R F S P K K T S S N C N G E

3836-11353

ATRX_Y_Thermo

4,710 4,720 4,730 4,740 4,750 4,760 4,770 4,780 4,790 4,800 4,810

AGAGAAGAAATTGGATGACTCTTGTTCAGGTTCTGTCACCTATTCTTATTCCGCTTTGATCGTCCCAAAAGAGATGATTAAGAAGGCCAAGAAGTTGATTGAAACCA
TCTCTTCTTTAACCTACTGAGAACAAGTCCAAGACAGTGGATAAGAATAAGGCGAAACTAGCAGGGTTTTCTCTACTAATTCTTCCGGTTCTTCAACTAACTTTGGT

328 330 332 334 336 338 340 342 344 346 348 350 352 354 356 358 360 362

E K K L D D S C S G S V T Y S Y S A L I V P K E M I K K A K K L I E T

3836-11353

ATRX_Y_Thermo

4,820 4,830 4,840 4,850 4,860 4,870 4,880 4,890 4,900 4,910 4,920

CCGCTAATATGAACTCCTCCTACGTTAAGTTTTTGAAGCAAGCTACCGACAACTCCGAGATTTCTTCTGCTACAAAATTGAGACAGCTGAAGGCTTTCAAATCCGTT
GGCGATTATACTTGAGGAGGATGCAATTCAAAAACTTCGTTCGATGGCTGTTGAGGCTCTAAAGAAGACGATGTTTTAACTCTGTCGACTTCCGAAAGTTTAGGCAA

364 366 368 370 372 374 376 378 380 382 384 386 388 390 392 394 396 398

T A N M N S S Y V K F L K Q A T D N S E I S S A T K L R Q L K A F K S V

3836-11353

ATRX_Y_Thermo

4,930 4,940 4,950 4,960 4,970 4,980 4,990 5,000 5,010 5,020

TTGGCTGATATTAAGAAAGCCCATTTGGCCTTGGAAGAGGATTTGAATAGTGAATTCAGAGCTATGGATGCCGTCAACAAAGAAAAGAATACCAAAGAACACAAGGT
AACCGACTATAATTCTTTCGGGTAAACCGGAACCTTCTCCTAAACTTATCACTTAAGTCTCGATACCTACGGCAGTTGTTTCTTTTCTTATGGTTTCTTGTGTTCCA

400 402 404 406 408 410 412 414 416 418 420 422 424 426 428 430 432 434

L A D I K K A H L A L E E D L N S E F R A M D A V N K E K N T K E H K V

3836-11353

ATRX_Y_Thermo

5,030 5,040 5,050 5,060 5,070 5,080 5,090 5,100 5,110 5,120 5,130

CATCGATGCCAAGTTTGAAACTAAGGCTAGAAAAGGTGAAAAGCCATGTGCTTTGGAAAAGAAGGATATCTCTAAGTCTGAAGCCAAGTTGTCCAGAAAGCAAGTTG
GTAGCTACGGTTCAAACTTTGATTCCGATCTTTTCCACTTTTCGGTACACGAAACCTTTTCTTCCTATAGAGATTCAGACTTCGGTTCAACAGGTCTTTCGTTCAAC

436 438 440 442 444 446 448 450 452 454 456 458 460 462 464 466 468

I D A K F E T K A R K G E K P C A L E K K D I S K S E A K L S R K Q V

3836-11353

ATRX_Y_Thermo

5,140 5,150 5,160 5,170 5,180 5,190 5,200 5,210 5,220 5,230 5,240

NsiI

ATTCTGAACACATGCATCAAAACGTTCCAACCGAAGAACAAAGGACTAACAAATCTACTGGTGGTGAACACAAGAAGTCCGATAGAAAAGAAGAACCACAATACGAA
TAAGACTTGTGTACGTAGTTTTGCAAGGTTGGCTTCTTGTTTCCTGATTGTTTAGATGACCACCACTTGTGTTCTTCAGGCTATCTTTTCTTCTTGGTGTTATGCTT

470 472 474 476 478 480 482 484 486 488 490 492 494 496 498 500 502 504

D S E H M H Q N V P T E E Q R T N K S T G G E H K K S D R K E E P Q Y E

3836-11353

ATRX_Y_Thermo

5,250 5,260 5,270 5,280 5,290 5,300 5,310 5,320 5,330 5,340 5,350

CCAGCTAACACCTCTGAAGATTTGGATATGGATATTGTCTCCGTTCCATCTTCTGTTCCAGAAGATATTTTCGAGAATTTGGAAACCGCTATGGAAGTCCAATCTTC
GGTCGATTGTGGAGACTTCTAAACCTATACCTATAACAGAGGCAAGGTAGAAGACAAGGTCTTCTATAAAAGCTCTTAAACCTTTGGCGATACCTTCAGGTTAGAAG
506 508 510 512 514 516 518 520 522 524 526 528 530 532 534 536 538 540
P A N T S E D L D M D I V S V P S S V P E D I F E N L E T A M E V Q S S
3836-11353
ATRX_Y_Thermo
5,360 5,370 5,380 5,390 5,400 5,410 5,420 5,430 5,440 5,450

BclI
CGTTGATCATCAAGGTGATGGTTCTTCAGGTACTGAGCAAGAAGTAGAATCTTCCTCTGTCAAACTGAACATCTCCTCTAAGGATAATAGAGGTGGTATCAAGTCTA
GCAACTAGTAGTTCCACTACCAAGAAGTCCATGACTCGTTCTTCATCTTAGAAGGAGACAGTTTGACTTGTAGAGGAGATTCCTATTATCTCCACCATAGTTCAGAT
542 544 546 548 550 552 554 556 558 560 562 564 566 568 570 572 574 576
V D H Q G D G S S G T E Q E V E S S S V K L N I S S K D N R G G I K S
3836-11353
ATRX_Y_Thermo
5,460 5,470 5,480 5,490 5,500 5,510 5,520 5,530 5,540 5,550 5,560

PshAI
AGACTACTGCCAAGGTCACCAAAGAATTATACGTCAAATTGACCCCAGTCTCCTTGTCTAATTCTCCAATCAAAGGTGCTGATTGTCAAGAAGTTCCACAAGACAAA
TCTGATGACGGTTCCAGTGGTTTCTTAATATGCAGTTTAACTGGGGTCAGAGGAACAGATTAAGAGGTTAGTTTCCACGACTAACAGTTCTTCAAGGTGTTCTGTTT
578 580 582 584 586 588 590 592 594 596 598 600 602 604 606 608 610 612
K T T A K V T K E L Y V K L T P V S L S N S P I K G A D C Q E V P Q D K
3836-11353
ATRX_Y_Thermo
5,570 5,580 5,590 5,600 5,610 5,620 5,630 5,640 5,650 5,660 5,670

GATGGTTACAAGTCTTGTGGTTTGAACCCCAAATTGGAAAAATGTGGTTTGGGTCAAGAAAACTCTGACAACGAACATTTGGTCGAAAACGAAGTCTCTTTGTTGTT
CTACCAATGTTCAGAACACCAAACTTGGGGTTTAACCTTTTTACACCAAACCCAGTTCTTTTGAGACTGTTGCTTGTAAACCAGCTTTTGCTTCAGAGAAACAACAA
614 616 618 620 622 624 626 628 630 632 634 636 638 640 642 644 646 648
D G Y K S C G L N P K L E K C G L G Q E N S D N E H L V E N E V S L L L
3836-11353
ATRX_Y_Thermo
5,680 5,690 5,700 5,710 5,720 5,730 5,740 5,750 5,760 5,770

StuI
GGAAGAAAGCGACTTGAGAAGATCTCCAAGAGTTAAGACTACACCATTGAGAAGGCCTACTGAAACTAATCCAGTTACCTCTAACTCTGACGAAGAATGTAACGAAA
CCTTCTTTCGCTGAACTCTTCTAGAGGTTCTCAATTCTGATGTGGTAACTCTTCCGGATGACTTTGATTAGGTCAATGGAGATTGAGACTGCTTCTTACATTGCTTT
650 652 654 656 658 660 662 664 666 668 670 672 674 676 678 680 682
E E S D L R R S P R V K T T P L R R P T E T N P V T S N S D E E C N E
3836-11353
ATRX_Y_Thermo
5,780 5,790 5,800 5,810 5,820 5,830 5,840 5,850 5,860 5,870 5,880

CTGTCAAAGAGAAGCAGAAGTTGTCAGTTCCAGTTAGAAAGAAGGACAAGAGAAACTCTTCCGATTCTGCTATTGATAACCCAAAGCCAAACAAGTTGCCAAAGTCT
GACAGTTTCTCTTCGTCTTCAACAGTCAAGGTCAATCTTTCTTCCTGTTCTCTTTGAGAAGGCTAAGACGATAACTATTGGGTTTCGGTTTGTTCAACGGTTTCAGA
684 686 688 690 692 694 696 698 700 702 704 706 708 710 712 714 716 718
T V K E K Q K L S V P V R K K D K R N S S D S A I D N P K P N K L P K S
3836-11353
ATRX_Y_Thermo
5,890 5,900 5,910 5,920 5,930 5,940 5,950 5,960 5,970 5,980 5,990

MscI
AAGCAATCTGAAACCGTTGACCAAAATTCCGATTCAGACGAAATGTTGGCCATCTTGAAAGAAGTCTCTAGAATGTCCCATTCCTCTTCATCTGATACCGATATTAA
TTCGTTAGACTTTGGCAACTGGTTTTAAGGCTAAGTCTGCTTTACAACCGGTAGAACTTTCTTCAGAGATCTTACAGGGTAAGGAGAAGTAGACTATGGCTATAATT
720 722 724 726 728 730 732 734 736 738 740 742 744 746 748 750 752 754
K Q S E T V D Q N S D S D E M L A I L K E V S R M S H S S S S D T D I N
3836-11353
ATRX_Y_Thermo
6,000 6,010 6,020 6,030 6,040 6,050 6,060 6,070 6,080 6,090

CGAAATTCACACCAACCATAAGACCCTGTACGATTTGAAAACTCAAGCTGGTAAAGATGACAAGGGTAAGAGAAAGAGAAAGTCCTCTACATCTGGTTCTGATTTCG
GCTTTAAGTGTGGTTGGTATTCTGGGACATGCTAAACTTTTGAGTTCGACCATTTCTACTGTTCCCATTCTCTTTCTCTTTCAGGAGATGTAGACCAAGACTAAAGC
756 758 760 762 764 766 768 770 772 774 776 778 780 782 784 786 788 790
E I H T N H K T L Y D L K T Q A G K D D K G K R K R K S S T S G S D F
3836-11353
ATRX_Y_Thermo
6,100 6,110 6,120 6,130 6,140 6,150 6,160 6,170 6,180 6,190 6,200

Tth111I
PflFI
ATACCAAGAAGGGTAAATCTGCCAAGTCCTCCATTATTTCCAAAAAGAAGAGACAGACCCAGTCCGAATCATCTAATTACGATTCTGAATTGGAGAAAGAAATCAAG
TATGGTTCTTCCCATTTAGACGGTTCAGGAGGTAATAAAGGTTTTTCTTCTCTGTCTGGGTCAGGCTTAGTAGATTAATGCTAAGACTTAACCTCTTTCTTTAGTTC
792 794 796 798 800 802 804 806 808 810 812 814 816 818 820 822 824 826
D T K K G K S A K S S I I S K K K R Q T Q S E S S N Y D S E L E K E I K
3836-11353
ATRX_Y_Thermo
6,210 6,220 6,230 6,240 6,250 6,260 6,270 6,280 6,290 6,300 6,310

TCCATGTCTAAGATTGGTGCTGCTAGAACTACTAAGAAGAGAATTCCAAACACCAAGGACTTCGATTCTTCCGAAGATGAGAAACACTCTAAGAAAGGTATGGACAA
AGGTACAGATTCTAACCACGACGATCTTGATGATTCTTCTCTTAAGGTTTGTGGTTCCTGAAGCTAAGAAGGCTTCTACTCTTTGTGAGATTCTTTCCATACCTGTT
828 830 832 834 836 838 840 842 844 846 848 850 852 854 856 858 860 862
S M S K I G A A R T T K K R I P N T K D F D S S E D E K H S K K G M D N
3836-11353
ATRX_Y_Thermo
6,320 6,330 6,340 6,350 6,360 6,370 6,380 6,390 6,400 6,410 6,420

PstI

TCAAGGCCACAAGAACTTAAAGACTTCTCAAGAGGGTTCTTCTGATGATGCTGAAAGAAAACAAGAAAGGGAAACCTTCTCATCTGCAGAAGGTACTGTTGATAAGG
AGTTCCGGTGTTCTTGAATTTCTGAAGAGTTCTCCCAAGAAGACTACTACGACTTTCTTTTGTTCTTTCCCTTTGGAAGAGTAGACGTCTTCCATGACAACTATTCC

864 866 868 870 872 874 876 878 880 882 884 886 888 890 892 894 896

Q G H K N L K T S Q E G S S D D A E R K Q E R E T F S S A E G T V D K

3836-11353

ATRX_Y_Thermo

6,430 6,440 6,450 6,460 6,470 6,480 6,490 6,500 6,510 6,520

ATACCACCATTATGGAATTGAGAGACAGATTGCCAAAGAAGCAACAAGCTTCAGCTTCTACTGATGGTGTTGACAAATTGTCCGGTAAAGAAGAATCTTTCACCTCC
TATGGTGGTAATACCTTAACTCTCTGTCTAACGGTTTCTTCGTTGTTCGAAGTCGAAGATGACTACCACAACTGTTTAACAGGCCATTTCTTCTTAGAAAGTGGAGG

898 900 902 904 906 908 910 912 914 916 918 920 922 924 926 928 930 932

D T T I M E L R D R L P K K Q Q A S A S T D G V D K L S G K E E S F T S

3836-11353

ATRX_Y_Thermo

6,530 6,540 6,550 6,560 6,570 6,580 6,590 6,600 6,610 6,620 6,630

BspMI
BfuAI

TTGGAAGTTAGGAAAGTTGCCGAAACAAAAGAGAAGTCGAAGCACTTGAAAACTAAGACCTGCAAAAAGGTTCAAGACGGCTTGTCTGATATTGCCGAAAAATTCTT
AACCTTCAATCCTTTCAACGGCTTTGTTTTCTCTTCAGCTTCGTGAACTTTTGATTCTGGACGTTTTTCCAAGTTCTGCCGAACAGACTATAACGGCTTTTTAAGAA

934 936 938 940 942 944 946 948 950 952 954 956 958 960 962 964 966 968

L E V R K V A E T K E K S K H L K T K T C K K V Q D G L S D I A E K F L

3836-11353

ATRX_Y_Thermo

6,640 6,650 6,660 6,670 6,680 6,690 6,700 6,710 6,720 6,730 6,740

GAAAAAGGACCAGTCTGACGAAACCTCAGAGGATGATAAGAAGCAATCTAAAAAGGGCACCGAAGAGAAAAAGAAGCCATCCGATTTTAAGAAAAAGGTCATCAAAA
CTTTTTCCTGGTCAGACTGCTTTGGAGTCTCCTACTATTCTTCGTTAGATTTTTCCCGTGGCTTCTCTTTTTCTTCGGTAGGCTAAAATTCTTTTTCCAGTAGTTTT

970 972 974 976 978 980 982 984 986 988 990 992 994 996 998 1000 1002 1004

K K D Q S D E T S E D D K K Q S K K G T E E K K K P S D F K K K V I K

3836-11353

ATRX_Y_Thermo

6,750 6,760 6,770 6,780 6,790 6,800 6,810 6,820 6,830 6,840

TGGAACAGCAGTACGAGTCATCTAGTGATGGTACTGAAAAATTGCCAGAGAGGGAAGAAATTTGTCATTTCCCAAAAGGTATCAAGCAAATCAAGAACGGTACTACC
ACCTTGTCGTCATGCTCAGTAGATCACTACCATGACTTTTTAACGGTCTCTCCCTTCTTTAAACAGTAAAGGGTTTTCCATAGTTCGTTTAGTTCTTGCCATGATGG

1006 1008 1010 1012 1014 1016 1018 1020 1022 1024 1026 1028 1030 1032 1034 1036 1038 1040

M E Q Q Y E S S S D G T E K L P E R E E I C H F P K G I K Q I K N G T T

3836-11353

ATRX_Y_Thermo

6,850 6,860 6,870 6,880 6,890 6,900 6,910 6,920 6,930 6,940 6,950

```
GATGGTGAAAAGAAGTCAAAGAAGATTAGAGACAAGACCTCCAAGAAGAAAGACGAATTGTCTGATTACGCTGAAAAGTCTACTGGTAAAGGTGATTCTTGCGATTC
CTACCACTTTTCTTCAGTTTCTTCTAATCTCTGTTCTGGAGGTTCTTCTTTCTGCTTAACAGACTAATGCGACTTTTCAGATGACCATTTCCACTAAGAACGCTAAG
   1042   1044   1046   1048   1050   1052   1054   1056   1058   1060   1062   1064   1066   1068   1070   1072   1074   1076
D G E K K S K K I R D K T S K K K D E L S D Y A E K S T G K G D S C D S
3836-11353
ATRX_Y_Thermo
6,960  6,970  6,980  6,990  7,000  7,010  7,020  7,030  7,040  7,050  7,060
```

```
CAGTGAGGATAAGAAAAGTAAGAATGGTGCTTACGGCAGGGAAAAGAAACGTTGTAAGTTGTTGGGTAAGAGTTCCAGAAAAAGACAAGACTGTTCCTCATCCGATA
GTCACTCCTATTCTTTTCATTCTTACCACGAATGCCGTCCCTTTTCTTTGCAACATTCAACAACCCATTCTCAAGGTCTTTTTCTGTTCTGACAAGGAGTAGGCTAT
   1078   1080   1082   1084   1086   1088   1090   1092   1094   1096   1098   1100   1102   1104   1106   1108   1110
S E D K K S K N G A Y G R E K K R C K L L G K S S R K R Q D C S S S D
3836-11353
ATRX_Y_Thermo
7,070  7,080  7,090  7,100  7,110  7,120  7,130  7,140  7,150  7,160
```

```
CTGAGAAGTATTCTATGAAGGAAGATGGCTGTAACTCCTCCGATAAGAGATTGAAAAGAATCGAGTTGAGAGAGAGGCGTAACTTGTCTAGTAAAAGAAACACCAAA
GACTCTTCATAAGATACTTCCTTCTACCGACATTGAGGAGGCTATTCTCTAACTTTTCTTAGCTCAACTCTCTCTCCGCATTGAACAGATCATTTTCTTTGTGGTTT
1112   1114   1116   1118   1120   1122   1124   1126   1128   1130   1132   1134   1136   1138   1140   1142   1144   1146
T E K Y S M K E D G C N S S D K R L K R I E L R E R R N L S S K R N T K
3836-11353
ATRX_Y_Thermo
7,170  7,180  7,190  7,200  7,210  7,220  7,230  7,240  7,250  7,260  7,270
```

```
GAGATCCAGTCCGGTTCTTCATCATCTGATGCAGAAGAATCCTCAGAAGATAACAAAAAGAAAAAGCAGAGGACCAGCTCTAAAAAGAAGGCCGTTATTGTCAAAGA
CTCTAGGTCAGGCCAAGAAGTAGTAGACTACGTCTTCTTAGGAGTCTTCTATTGTTTTTCTTTTTCGTCTCCTGGTCGAGATTTTTCTTCCGGCAATAACAGTTTCT
1148   1150   1152   1154   1156   1158   1160   1162   1164   1166   1168   1170   1172   1174   1176   1178   1180   1182
E I Q S G S S S S D A E E S S E D N K K K K Q R T S S K K K A V I V K E
3836-11353
ATRX_Y_Thermo
7,280  7,290  7,300  7,310  7,320  7,330  7,340  7,350  7,360  7,370  7,380
```

```
GAAAAAGAGGAACTCTTTGAGGACTTCCACCAAAAGAAAGCAGGCAGATATTACCTCATCCTCCAGTTCAGATATCGAAGATGATGACCAAAACTCTATCGGTGAAG
CTTTTTCTCCTTGAGAAACTCCTGAAGGTGGTTTTCTTTCGTCCGTCTATAATGGAGTAGGAGGTCAAGTCTATAGCTTCTACTACTGGTTTTGAGATAGCCACTTC
 1184   1186   1188   1190   1192   1194   1196   1198   1200   1202   1204   1206   1208   1210   1212   1214   1216   1218
K K R N S L R T S T K R K Q A D I T S S S S S D I E D D D Q N S I G E
3836-11353
ATRX_Y_Thermo
7,390  7,400  7,410  7,420  7,430  7,440  7,450  7,460  7,470  7,480  7,490
```

GTAGTTCTGATGAGCAAAAGATTAAGCCAGTCACCGAAAATTTGGTGTTGTCATCTCATACTGGTTTCTGCCAATCTTCAGGTGATGAAGCTTTGTCTAAATCTGTC
CATCAAGACTACTCGTTTTCTAATTCGGTCAGTGGCTTTTAAACCACAACAGTAGAGTATGACCAAAGACGGTTAGAAGTCCACTACTTCGAAACAGATTTAGACAG
1220 1222 1224 1226 1228 1230 1232 1234 1236 1238 1240 1242 1244 1246 1248 1250 1252 1254
G S S D E Q K I K P V T E N L V L S S H T G F C Q S S G D E A L S K S V
3836-11353
ATRX_Y_Thermo
7,500 7,510 7,520 7,530 7,540 7,550 7,560 7,570 7,580 7,590

CCAGTTACTGTTGACGATGATGATGACGATAATGACCCAGAAAACAGAATCGCCAAAAAGATGTTGCTGGAAGAGATTAAGGCTAACTTGAGCAGTGATGAAGATGG
GGTCAATGACAACTGCTACTACTACTGCTATTACTGGGTCTTTTGTCTTAGCGGTTTTTCTACAACGACCTTCTCTAATTCCGATTGAACTCGTCACTACTTCTACC
1256 1258 1260 1262 1264 1266 1268 1270 1272 1274 1276 1278 1280 1282 1284 1286 1288 1290
P V T V D D D D D D N D P E N R I A K K M L L E E I K A N L S S D E D G
3836-11353
ATRX_Y_Thermo
7,600 7,610 7,620 7,630 7,640 7,650 7,660 7,670 7,680 7,690 7,700

ATCTTCTGACGATGAACCAGAGGAAGGTAAAAAGAGAACTGGTAAGCAAAACGAAGAAAACCCTGGTGATGAGGAAGCTAAGAATCAAGTCAATTCCGAATCTGACT
TAGAAGACTGCTACTTGGTCTCCTTCCATTTTTCTCTTGACCATTCGTTTTGCTTCTTTTGGGACCACTACTCCTTCGATTCTTAGTTCAGTTAAGGCTTAGACTGA
1292 1294 1296 1298 1300 1302 1304 1306 1308 1310 1312 1314 1316 1318 1320 1322 1324
S S D D E P E E G K K R T G K Q N E E N P G D E E A K N Q V N S E S D
3836-11353
ATRX_Y_Thermo
7,710 7,720 7,730 7,740 7,750 7,760 7,770 7,780 7,790 7,800 7,810

CTGACTCAGAAGAAAGTAAAAAGCCAAGATACAGACACAGGTTGTTGAGACATAAGTTGACTGTTTCAGATGGTGAATCTGGCGAGGAAAAGAAAACTAAGCCAAAA
GACTGAGTCTTCTTTCATTTTTCGGTTCTATGTCTGTGTCCAACAACTCTGTATTCAACTGACAAAGTCTACCACTTAGACCGCTCCTTTTCTTTTGATTCGGTTTT
1326 1328 1330 1332 1334 1336 1338 1340 1342 1344 1346 1348 1350 1352 1354 1356 1358 1360
S D S E E S K K P R Y R H R L L R H K L T V S D G E S G E E K K T K P K
3836-11353
ATRX_Y_Thermo
7,820 7,830 7,840 7,850 7,860 7,870 7,880 7,890 7,900 7,910

BarI
GAGCACAAAGAGGTCAAGGGTAGAAATAGACGTAAGGTTTCTTCAGAAGATTCCGAGGATTCTGACTTCCAAGAATCTGGTGTTTCAGAAGAAGTGTCTGAATCTGA
CTCGTGTTTCTCCAGTTCCCATCTTTATCTGCATTCCAAAGAAGTCTTCTAAGGCTCCTAAGACTGAAGGTTCTTAGACCACAAAGTCTTCTTCACAGACTTAGACT
1362 1364 1366 1368 1370 1372 1374 1376 1378 1380 1382 1384 1386 1388 1390 1392 1394 1396
E H K E V K G R N R R K V S S E D S E D S D F Q E S G V S E E V S E S E
3836-11353
ATRX_Y_Thermo
7,920 7,930 7,940 7,950 7,960 7,970 7,980 7,990 8,000 8,010 8,020

GGACGAACAAAGACCAAGAACTAGATCTGCTAAAAAGGCCGAATTGGAAGAGAATCAGAGATCCTACAAACAGAAGAAGAAACGTAGACGTATCAAGGTCCAAGAGG
CCTGCTTGTTTCTGGTTCTTGATCTAGACGATTTTTCCGGCTTAACCTTCTCTTAGTCTCTAGGATGTTTGTCTTCTTCTTTGCATCTGCATAGTTCCAGGTTCTCC
1398 1400 1402 1404 1406 1408 1410 1412 1414 1416 1418 1420 1422 1424 1426 1428 1430 1432
D E Q R P R T R S A K K A E L E E N Q R S Y K Q K K K R R R I K V Q E
3836-11353
ATRX_Y_Thermo
8,030 8,040 8,050 8,060 8,070 8,080 8,090 8,100 8,110 8,120 8,130

ATTCATCTTCCGAAAACAAAAGCAATTCTGAGGAAGAAGAGGAAGAGAAAGAAGAAGAAGAAGAGGAAGAGGAAGAAGAAGAAGAAGAGGAAGAGGACGAAAACGAC
TAAGTAGAAGGCTTTTGTTTTCGTTAAGACTCCTTCTTCTCCTTCTCTTTCTTCTTCTTCTTCTCCTTCTCCTTCTTCTTCTTCTTCTCCTTCTCCTGCTTTTGCTG
1434 1436 1438 1440 1442 1444 1446 1448 1450 1452 1454 1456 1458 1460 1462 1464 1466 1468
D S S S E N K S N S E E E E E E K E E E E E E E E E E E E E E E D E N D
3836-11353
ATRX_Y_Thermo
8,140 8,150 8,160 8,170 8,180 8,190 8,200 8,210 8,220 8,230

GATTCTAAATCTCCAGGTAAAGGTAGGAAAAAGATCCGTAAGATTTTGAAGGACGACAAGTTGAGAACTGAAACCCAAAATGCTCTGAAAGAAGAGGAAGAACGTAG
CTAAGATTTAGAGGTCCATTTCCATCCTTTTTCTAGGCATTCTAAAACTTCCTGCTGTTCAACTCTTGACTTTGGGTTTTACGAGACTTTCTTCTCCTTCTTGCATC
1470 1472 1474 1476 1478 1480 1482 1484 1486 1488 1490 1492 1494 1496 1498 1500 1502 1504
D S K S P G K G R K K I R K I L K D D K L R T E T Q N A L K E E E E R R
3836-11353
ATRX_Y_Thermo
8,240 8,250 8,260 8,270 8,280 8,290 8,300 8,310 8,320 8,330 8,340

AAAGAGAATTGCCGAAAGGGAACGTGAAAGGGAAAAATTGAGAGAAGTCATCGAAATCGAAGATGCCTCTCCAACTAAGTGTCCAATTACTACTAAGTTGGTCCTGG
TTTCTCTTAACGGCTTTCCCTTGCACTTTCCCTTTTTAACTCTCTTCAGTAGCTTTAGCTTCTACGGAGAGGTTGATTCACAGGTTAATGATGATTCAACCAGGACC
1506 1508 1510 1512 1514 1516 1518 1520 1522 1524 1526 1528 1530 1532 1534 1536 1538
K R I A E R E R E R E K L R E V I E I E D A S P T K C P I T T K L V L
3836-11353
ATRX_Y_Thermo
8,350 8,360 8,370 8,380 8,390 8,400 8,410 8,420 8,430 8,440 8,450

AAGAGCCTTTAGTTCAAGTCCA
ATGAGGATGAAGAGACAAAAGAGCCTTTAGTTCAAGTCCACAGAAACATGGTTATCAAGTTGAAGCCACACCAAGTTGATGGTGTACAATTCATGTGGGATTGCTGT
TACTCCTACTTCTCTGTTTTCTCGGAAATCAAGTTCAGGTGTCTTTGTACCAATAGTTCAACTTCGGTGTGGTTCAACTACCACATGTTAAGTACACCCTAACGACA
1540 1542 1544 1546 1548 1550 1552 1554 1556 1558 1560 1562 1564 1566 1568 1570 1572 1574
D E D E E T K E P L V Q V H R N M V I K L K P H Q V D G V Q F M W D C C
3836-11353
ATRX_Y_Thermo
8,460 8,470 8,480 8,490 8,500 8,510 8,520 8,530 8,540 8,550 8,560

TGTGAATCTGTCAAAAAGACCAAAAAGTCTCCAGGTTCAGGTTGTATTTTGGCTCATTGCATGGGTTTGGGTAAAACATTGCAAGTGGTTTCTTTCTTGCACACCGT
ACACTTAGACAGTTTTTCTGGTTTTTCAGAGGTCCAAGTCCAACATAAAACCGAGTAACGTACCCAAACCCATTTTGTAACGTTCACCAAAGAAAGAACGTGTGGCA
1576 1578 1580 1582 1584 1586 1588 1590 1592 1594 1596 1598 1600 1602 1604 1606 1608 1610
C E S V K K T K K S P G S G C I L A H C M G L G K T L Q V V S F L H T V
3836-11353
ATRX_Y_Thermo
8,570 8,580 8,590 8,600 8,610 8,620 8,630 8,640 8,650 8,660

TTTGTTGTGTGACAAGTTGGATTTTTCTACCGCTTTGGTTGTTTGTCCATTGAACACTGCTTTGAACTGGATGAACGAATTCGAAAAATGGCAAGAGGGTCTGAAAG
AAACAACACACTGTTCAACCTAAAAAGATGGCGAAACCAACAAACAGGTAACTTGTGACGAAACTTGACCTACTTGCTTAAGCTTTTTACCGTTCTCCCAGACTTTC
1612 1614 1616 1618 1620 1622 1624 1626 1628 1630 1632 1634 1636 1638 1640 1642 1644 1646
L L C D K L D F S T A L V V C P L N T A L N W M N E F E K W Q E G L K
3836-11353
ATRX_Y_Thermo
8,670 8,680 8,690 8,700 8,710 8,720 8,730 8,740 8,750 8,760 8,770

StuI
ACGACGAAAAATTGGAAGTTTCAGAATTGGCTACCGTTAAGAGGCCTCAAGAAAGATCTTATATGTTGCAAAGGTGGCAAGAGGATGGTGGTGTTATGATTATTGGT
TGCTGCTTTTTAACCTTCAAAGTCTTAACCGATGGCAATTCTCCGGAGTTCTTTCTAGAATATACAACGTTTCCACCGTTCTCCTACCACCACAATACTAATAACCA
1648 1650 1652 1654 1656 1658 1660 1662 1664 1666 1668 1670 1672 1674 1676 1678 1680 1682
D D E K L E V S E L A T V K R P Q E R S Y M L Q R W Q E D G G V M I I G
3836-11353
ATRX_Y_Thermo
8,780 8,790 8,800 8,810 8,820 8,830 8,840 8,850 8,860 8,870 8,880

TACGAGATGTACAGAAATTTGGCCCAAGGTAGAAACGTCAAGTCTAGGAAGTTGAAAGAGATTTTCAACAAGGCTTTGGTAGATCCAGGTCCAGATTTTGTTGTTTG
ATGCTCTACATGTCTTTAAACCGGGTTCCATCTTTGCAGTTCAGATCCTTCAACTTTCTCTAAAAGTTGTTCCGAAACCATCTAGGTCCAGGTCTAAAACAACAAAC
1684 1686 1688 1690 1692 1694 1696 1698 1700 1702 1704 1706 1708 1710 1712 1714 1716 1718
Y E M Y R N L A Q G R N V K S R K L K E I F N K A L V D P G P D F V V C
3836-11353
ATRX_Y_Thermo
8,890 8,900 8,910 8,920 8,930 8,940 8,950 8,960 8,970 8,980

TGATGAAGGTCACATCTTGAAGAATGAAGCTTCTGCTGTTTCTAAGGCCATGAACTCTATTAGATCCAGACGTAGAATTATCTTGACCGGTACTCCATTGCAAAACA
ACTACTTCCAGTGTAGAACTTCTTACTTCGAAGACGACAAAGATTCCGGTACTTGAGATAATCTAGGTCTGCATCTTAATAGAACTGGCCATGAGGTAACGTTTTGT
1720 1722 1724 1726 1728 1730 1732 1734 1736 1738 1740 1742 1744 1746 1748 1750 1752
D E G H I L K N E A S A V S K A M N S I R S R R R I I L T G T P L Q N
3836-11353
ATRX_Y_Thermo
8,990 9,000 9,010 9,020 9,030 9,040 9,050 9,060 9,070 9,080 9,090

ACTTGATCGAATACCACTGTATGGTCAACTTCATCAAAGAAAATCTGCTGGGCTCGATCAAAGAATTCCGTAACAGATTCATTAACCCAATCCAAAACGGTCAATGT
TGAACTAGCTTATGGTGACATACCAGTTGAAGTAGTTTCTTTTAGACGACCCGAGCTAGTTTCTTAAGGCATTGTCTAAGTAATTGGGTTAGGTTTTGCCAGTTACA

1754 1756 1758 1760 1762 1764 1766 1768 1770 1772 1774 1776 1778 1780 1782 1784 1786 1788

N L I E Y H C M V N F I K E N L L G S I K E F R N R F I N P I Q N G Q C

3836-11353

ATRX_Y_Thermo

9,100 9,110 9,120 9,130 9,140 9,150 9,160 9,170 9,180 9,190 9,200

BmtI
NheI

GCTGATTCTACTATGGTTGATGTTAGGGTTATGAAGAAGCGTGCTCATATCTTGTACGAAATGCTAGCTGGTTGTGTTCAAAGAAAGGATTATACTGCCTTGACCAA
CGACTAAGATGATACCAACTACAATCCCAATACTTCTTCGCACGAGTATAGAACATGCTTTACGATCGACCAACACAAGTTTCTTTCCTAATATGACGGAACTGGTT

GACCAACACAAGTTTCTTTCCT

1790 1792 1794 1796 1798 1800 1802 1804 1806 1808 1810 1812 1814 1816 1818 1820 1822 1824

A D S T M V D V R V M K K R A H I L Y E M L A G C V Q R K D Y T A L T K

3836-11353

ATRX_Y_Thermo

9,210 9,220 9,230 9,240 9,250 9,260 9,270 9,280 9,290 9,300

GTTCTTGCCTCCAAAACATGAATACGTTTTGGCCGTTAGAATGACCTCTATTCAATGCAAGTTGTACCAGTACTACTTGGATCATTTGACTGGTGTTGGTAACAACT
CAAGAACGGAGGTTTTGTACTTATGCAAAACCGGCAATCTTACTGGAGATAAGTTACGTTCAACATGGTCATGATGAACCTAGTAAACTGACCACAACCATTGTTGA

1826 1828 1830 1832 1834 1836 1838 1840 1842 1844 1846 1848 1850 1852 1854 1856 1858 1860

F L P P K H E Y V L A V R M T S I Q C K L Y Q Y Y L D H L T G V G N N

3836-11353

ATRX_Y_Thermo

9,310 9,320 9,330 9,340 9,350 9,360 9,370 9,380 9,390 9,400 9,410

NcoI
BtgI

CAGAAGGCGGTAGAGGTAAAGCTGGTGCTAAGTTGTTTCAAGACTTCCAGATGTTGTCTAGGATTTGGACTCATCCATGGTGTTTACAATTGGACTACATCTCCAAA
GTCTTCCGCCATCTCCATTTCGACCACGATTCAACAAAGTTCTGAAGGTCTACAACAGATCCTAAACCTGAGTAGGTACCACAAATGTTAACCTGATGTAGAGGTTT

1862 1864 1866 1868 1870 1872 1874 1876 1878 1880 1882 1884 1886 1888 1890 1892 1894 1896

S E G G R G K A G A K L F Q D F Q M L S R I W T H P W C L Q L D Y I S K

3836-11353

ATRX_Y_Thermo

9,420 9,430 9,440 9,450 9,460 9,470 9,480 9,490 9,500 9,510 9,520

BcgI

GAGAACAAGGGTTACTTCGACGAAGATAGCATGGACGAATTCATTGCTTCTGATTCAGATGAGACTTCCATGTCTTTGTCCTCTGATGATTACACCAAGAAAAAGAA
CTCTTGTTCCCAATGAAGCTGCTTCTATCGTACCTGCTTAAGTAACGAAGACTAAGTCTACTCTGAAGGTACAGAAACAGGAGACTACTAATGTGGTTCTTTTTCTT

1898 1900 1902 1904 1906 1908 1910 1912 1914 1916 1918 1920 1922 1924 1926 1928 1930 1932

E N K G Y F D E D S M D E F I A S D S D E T S M S L S S D D Y T K K K K

3836-11353

ATRX_Y_Thermo

9,530 9,540 9,550 9,560 9,570 9,580 9,590 9,600 9,610 9,620 9,630

GAAAGGCAAAAAGGGCAAGAAGGACTCTTCTAGTTCTGGTTCAGGTTCCGATAATGATGTTGAAGTTATCAAGGTGTGGAACTCTAGAAGCAGAGGTGGTGGCGAAG
CTTTCCGTTTTTCCCGTTCTTCCTGAGAAGATCAAGACCAAGTCCAAGGCTATTACTACAACTTCAATAGTTCCACACCTTGAGATCTTCGTCTCCACCACCGCTTC

1934 1936 1938 1940 1942 1944 1946 1948 1950 1952 1954 1956 1958 1960 1962 1964 1966

K G K K G K K D S S S S G S G S D N D V E V I K V W N S R S R G G G E

3836-11353

ATRX_Y_Thermo

9,640 9,650 9,660 9,670 9,680 9,690 9,700 9,710 9,720 9,730

XcmI

GTAACGTTGACGAAACTGGTAACAATCCATCTGTGTCTTTGAAGTTGGAAGAGTCTAAAGCTACCTCATCTTCTAATCCATCATCTCCAGCACCAGATTGGTACAAG
CATTGCAACTGCTTTGACCATTGTTAGGTAGACACAGAAACTTCAACCTTCTCAGATTTCGATGGAGTAGAAGATTAGGTAGTAGAGGTCGTGGTCTAACCATGTTC

1968 1970 1972 1974 1976 1978 1980 1982 1984 1986 1988 1990 1992 1994 1996 1998 2000 2002

G N V D E T G N N P S V S L K L E E S K A T S S S N P S S P A P D W Y K

3836-11353

ATRX_Y_Thermo

9,740 9,750 9,760 9,770 9,780 9,790 9,800 9,810 9,820 9,830 9,840

GATTTTGTTACTGATGCTGATGCCGAAGTGTTGGAACATTCTGGTAAAATGGTCTTGTTGTTCGAGATCTTGAGAATGGCTGAAGAAATCGGTGATAAGGTGCTAGT
CTAAAACAATGACTACGACTACGGCTTCACAACCTTGTAAGACCATTTTACCAGAACAACAAGCTCTAGAACTCTTACCGACTTCTTTAGCCACTATTCCACGATCA

2004 2006 2008 2010 2012 2014 2016 2018 2020 2022 2024 2026 2028 2030 2032 2034 2036 2038

D F V T D A D A E V L E H S G K M V L L F E I L R M A E E I G D K V L V

3836-11353

ATRX_Y_Thermo

9,850 9,860 9,870 9,880 9,890 9,900 9,910 9,920 9,930 9,940 9,950

CTTTTCCCAATCCTTGATTTCCTTGGATTTGATCGAAGATTTCCTGGAATTGGCCTCTAGAGAAAAGACAGAGGATAAGGACAAACCCTTGATCTACAAAGGTGAAG
GAAAAGGGTTAGGAACTAAAGGAACCTAAACTAGCTTCTAAAGGACCTTAACCGGAGATCTCTTTTCTGTCTCCTATTCCTGTTTGGGAACTAGATGTTTCCACTTC

2040 2042 2044 2046 2048 2050 2052 2054 2056 2058 2060 2062 2064 2066 2068 2070 2072 2074

F S Q S L I S L D L I E D F L E L A S R E K T E D K D K P L I Y K G E

3836-11353

ATRX_Y_Thermo

9,960 9,970 9,980 9,990 10,000 10,010 10,020 10,030 10,040 10,050

GCAAATGGTTGAGAAACATCGACTATTACAGATTGGATGGTTCTACTACTGCCCAATCTAGGAAGAAGTGGGCTGAAGAGTTTAACGACGAAACTAATGTTAGAGGC
CGTTTACCAACTCTTTGTAGCTGATAATGTCTAACCTACCAAGATGATGACGGGTTAGATCCTTCTTCACCCGACTTCTCAAATTGCTGCTTTGATTACAATCTCCG
2076 2078 2080 2082 2084 2086 2088 2090 2092 2094 2096 2098 2100 2102 2104 2106 2108 2110
G K W L R N I D Y Y R L D G S T T A Q S R K K W A E E F N D E T N V R G
3836-11353
ATRX_Y_Thermo
10,060 10,070 10,080 10,090 10,100 10,110 10,120 10,130 10,140 10,150 10,160

CGTCTGTTTATTATCTCTACTAAGGCTGGTTCCTTGGGCATTAACTTGGTTGCTGCAAATAGGGTTATTATCTTCGATGCTTCTTGGAACCCATCCTACGATATTCA
GCAGACAAATAATAGAGATGATTCCGACCAAGGAACCCGTAATTGAACCAACGACGTTTATCCCAATAATAGAAGCTACGAAGAACCTTGGGTAGGATGCTATAAGT
2112 2114 2116 2118 2120 2122 2124 2126 2128 2130 2132 2134 2136 2138 2140 2142 2144 2146
R L F I I S T K A G S L G I N L V A A N R V I I F D A S W N P S Y D I Q
3836-11353
ATRX_Y_Thermo
10,170 10,180 10,190 10,200 10,210 10,220 10,230 10,240 10,250 10,260 10,270

ATCTATCTTCAGGGTTTACAGATTCGGTCAAACAAAACCAGTTTACGTCTACAGATTTCTGGCTCAAGGTACTATGGAAGATAAGATATACGATAGGCAAGTCACCA
TAGATAGAAGTCCCAAATGTCTAAGCCAGTTTGTTTTGGTCAAATGCAGATGTCTAAAGACCGAGTTCCATGATACCTTCTATTCTATATGCTATCCGTTCAGTGGT
2148 2150 2152 2154 2156 2158 2160 2162 2164 2166 2168 2170 2172 2174 2176 2178 2180
S I F R V Y R F G Q T K P V Y V Y R F L A Q G T M E D K I Y D R Q V T
3836-11353
ATRX_Y_Thermo
10,280 10,290 10,300 10,310 10,320 10,330 10,340 10,350 10,360 10,370

AGCAGTCATTGAGTTTTAGAGTTGTCGATCAACAACAGGTCGAAAGACATTTCACCATGAATGAATTGACTGAGTTGTACACCTTCGAACCAGATTTGTTGGATGAT
TCGTCAGTAACTCAAAATCTCAACAGCTAGTTGTTGTCCAGCTTTCTGTAAAGTGGTACTTACTTAACTGACTCAACATGTGGAAGCTTGGTCTAAACAACCTACTA
2182 2184 2186 2188 2190 2192 2194 2196 2198 2200 2202 2204 2206 2208 2210 2212 2214 2216
K Q S L S F R V V D Q Q Q V E R H F T M N E L T E L Y T F E P D L L D D
3836-11353
ATRX_Y_Thermo
10,390 10,400 10,410 10,420 10,430 10,440 10,450 10,460 10,470 10,480

CCTAATTCCGAGAAGAAGAAGAAAAGGGATACTCCAATGTTGCCAAAGGATACAATTTTGGCAGAGTTGTTGCAGATCCACAAAGAACATATCGTTGGTTACCATGA
GGATTAAGGCTCTTCTTCTTCTTTTCCCTATGAGGTTACAACGGTTTCCTATGTTAAAACCGTCTCAACAACGTCTAGGTGTTTCTTGTATAGCAACCAATGGTACT
2218 2220 2222 2224 2226 2228 2230 2232 2234 2236 2238 2240 2242 2244 2246 2248 2250 2252
P N S E K K K K R D T P M L P K D T I L A E L L Q I H K E H I V G Y H E
3836-11353
ATRX_Y_Thermo
10,490 10,500 10,510 10,520 10,530 10,540 10,550 10,560 10,570 10,580 10,590

ACACGACAGTTTATTGGATCACAAAGAGGAAGAAGAGTTGACCGAAGAAGAGAGAAAGGCTGCTTGGGCTGAATATGAAGCTGAAAAGAAAGGTTTGACCATGAGGT
TGTGCTGTCAAATAACCTAGTGTTTCTCCTTCTTCTCAACTGGCTTCTTCTCTCTTTCCGACGAACCCGACTTATACTTCGACTTTTCTTTCCAAACTGGTACTCCA
2254 2256 2258 2260 2262 2264 2266 2268 2270 2272 2274 2276 2278 2280 2282 2284 2286 2288
H D S L L D H K E E E E L T E E E R K A A W A E Y E A E K K G L T M R
3836-11353
ATRX_Y_Thermo
10,600 10,610 10,620 10,630 10,640 10,650 10,660 10,670 10,680 10,690 10,700

TCAATATTCCAACCGGTACTAATTTGCCACCAGTCTCATTCAATTCTCAAACCCCATATATCCCATTCAACTTGGGTGCTTTGTCTGCCATGTCAAATCAACAACTG
AGTTATAAGGTTGGCCATGATTAAACGGTGGTCAGAGTAAGTTAAGAGTTTGGGGTATATAGGGTAAGTTGAACCCACGAAACAGACGGTACAGTTTAGTTGTTGAC
2290 2292 2294 2296 2298 2300 2302 2304 2306 2308 2310 2312 2314 2316 2318 2320 2322 2324
F N I P T G T N L P P V S F N S Q T P Y I P F N L G A L S A M S N Q Q L
3836-11353
ATRX_Y_Thermo
10,710 10,720 10,730 10,740 10,750 10,760 10,770 10,780 10,790 10,800

GAAGATTTGATTAACCAGGGTAGAGAGAAGGTTGTCGAAGCTACTAATTCTGTTACCGCTGTTAGAATCCAACCTTTGGAAGATATTATCTCCGCCGTTTGGAAAGA
CTTCTAAACTAATTGGTCCCATCTCTCTTCCAACAGCTTCGATGATTAAGACAATGGCGACAATCTTAGGTTGGAAACCTTCTATAATAGAGGCGGCAAACCTTTCT
2326 2328 2330 2332 2334 2336 2338 2340 2342 2344 2346 2348 2350 2352 2354 2356 2358 2360
E D L I N Q G R E K V V E A T N S V T A V R I Q P L E D I I S A V W K E
3836-11353
ATRX_Y_Thermo
10,810 10,820 10,830 10,840 10,850 10,860 10,870 10,880 10,890 10,900 10,910

AAACATGAACTTGTCAGAAGCTCAAGTTCAAGCTTTGGCCTTATCTAGACAAGCTAGTCAAGAATTGGATGTCAAAAGAAGGGAAGCCATCTACAATGATGTTTTGA
TTTGTACTTGAACAGTCTTCGAGTTCAAGTTCGAAACCGGAATAGATCTGTTCGATCAGTTCTTAACCTACAGTTTTCTTCCCTTCGGTAGATGTTACTACAAAACT
2362 2364 2366 2368 2370 2372 2374 2376 2378 2380 2382 2384 2386 2388 2390 2392 2394
N M N L S E A Q V Q A L A L S R Q A S Q E L D V K R R E A I Y N D V L
3836-11353
ATRX_Y_Thermo
10,920 10,930 10,940 10,950 10,960 10,970 10,980 10,990 11,000 11,010 11,020

BsaBI
CCAAGCAACAGATGTTGATCTCTTGCGTGCAAAGAATCTTGATGAACAGAAGGTTGCAACAACAGTACAACCAGCAACAACAACAGCAGATGACATATCAACAAGCT
GGTTCGTTGTCTACAACTAGAGAACGCACGTTTCTTAGAACTACTTGTCTTCCAACGTTGTTGTCATGTTGGTCGTTGTTGTTGTCGTCTACTGTATAGTTGTTCGA
2396 2398 2400 2402 2404 2406 2408 2410 2412 2414 2416 2418 2420 2422 2424 2426 2428 2430
T K Q Q M L I S C V Q R I L M N R R L Q Q Q Y N Q Q Q Q Q Q M T Y Q Q A
3836-11353
ATRX_Y_Thermo
11,030 11,040 11,050 11,060 11,070 11,080 11,090 11,100 11,110 11,120

ACTTTGGGTCACTTGATGATGCCTAAACCACCAAACTTGATTATGAACCCATCTAACTACCAACAGATCGACATGAGAGGAATGTATCAACCAGTTGCTGGTGGTAT
TGAAACCCAGTGAACTACTACGGATTTGGTGGTTTGAACTAATACTTGGGTAGATTGATGGTTGTCTAGCTGTACTCTCCTTACATAGTTGGTCAACGACCACCATA
2432 2434 2436 2438 2440 2442 2444 2446 2448 2450 2452 2454 2456 2458 2460 2462 2464 2466
T L G H L M M P K P P N L I M N P S N Y Q Q I D M R G M Y Q P V A G G M
3836-11353
ATRX_Y_Thermo
11,130 11,140 11,150 11,160 11,170 11,180 11,190 11,200 11,210 11,220 11,230

GCAACCTCCACCATTACAAAGGGCTCCACCACCAATGAGATCTAAAAATCCTGGTCCATCTCAAGGCAAGTCTATGGACTACAAGGATGATGATGGTGATTACAAGG
CGTTGGAGGTGGTAATGTTTCCCGAGGTGGTGGTTACTCTAGATTTTTAGGACCAGGTAGAGTTCCGTTCAGATACCTGATGTTCCTACTACTACCACTAATGTTCC
2468 2470 2472 2474 2476 2478 2480 2482 2484 2486 2488 2490 2492 2494 2496 2498 2500 2502
Q P P P L Q R A P P P M R S K N P G P S Q G K S M D Y K D D D G D Y K
3836-11353
ATRX_Y_Thermo
11,240 11,250 11,260 11,270 11,280 11,290 11,300 11,310 11,320 11,330 11,340

ATGACGACTAATAAATTGAATTGAATTGAAATCGATAGATCAATTTTTTTCTTTTCTCTTTCCCCATCCTTTACGCTAAAATAATAGTTTATTTTATTTTTTGAATA
TACTGCTGATTATTTAACTTAACTTAACTTTAGCTATCTAGTTAAAAAAAGAAAAGAGAAAGGGGTAGGAAATGCGATTTTATTATCAAATAAAATAAAAAACTTAT
2504 2506
D D D *
3836-1...
ATRX_Y_Thermo
11,350 11,360 11,370 11,380 11,390 11,400 11,410 11,420 11,430 11,440

XmaI
SmaI
AvaI
TspMI
BsoBI
SnaBI
BsaAI
TTTTTTATTTATATACGTATATATAGACTATTATTTATCTTTTAATGATTATTAAGATTTTTATTAAAAAAAAATTCGCTCCTCTTTTAATGCCTTTATGCCCCGGG
AAAAAATAAATATATGCATATATATCTGATAATAAATAGAAAATTACTAATAATTCTAAAAATAATTTTTTTTTAAGCGAGGAGAAAATTACGGAAATACGGGGCCC
ATRX_Y_Thermo
11,460 11,470 11,480 11,490 11,500 11,510 11,520 11,530 11,540 11,550

PaeR7I
PspXI
BsoBI
XhoI
TliI
Acc65I
ApaI
PstI SalI AvaI PspOMI KpnI
CTGCAGGAATTCGATATCAAGCTTATCGATACCGTCGACCTCGAGGGGGGGCCCGGTACCCAGCTTTTGTTCCCTTTAGTGAGGGTTAATTCCGAGCTTGGCGTAAT
GACGTCCTTAAGCTATAGTTCGAATAGCTATGGCAGCTGGAGCTCCCCCCCGGGCCATGGGTCGAAAACAAGGGAAATCACTCCCAATTAAGGCTCGAACCGCATTA
T3_promoter
11,560 11,570 11,580 11,590 11,600 11,610 11,620 11,630 11,640 11,650 11,660

CATGGTCATAGCTGTTTCCTGTGTGAAATTGTTATCCGCTCACAATTCCACACAACATAGGAGCCGGAAGCATAAAGTGTAAAGCCTGGGGTGCCTAATGAGTGAGG
GTACCAGTATCGACAAAGGACACACTTTAACAATAGGCGAGTGTTAAGGTGTGTTGTATCCTCGGCCTTCGTATTTCACATTTCGGACCCCACGGATTACTCACTCC

11,670 11,680 11,690 11,700 11,710 11,720 11,730 11,740 11,750 11,760 11,770

TAACTCACATTAATTGCGTTGCGCTCACTGCCCGCTTTCCAGTCGGGAAACCTGTCGTGCCAGCTGCATTAATGAATCGGCCAACGCGCGGGGAGAGGCGGTTTGCG
ATTGAGTGTAATTAACGCAACGCGAGTGACGGGCGAAAGGTCAGCCCTTTGGACAGCACGGTCGACGTAATTACTTAGCCGGTTGCGCGCCCCTCTCCGCCAAACGC

11,780 11,790 11,800 11,810 11,820 11,830 11,840 11,850 11,860 11,870

BspQI
SapI

TATTGGGCGCTCTTCCGCTTCCTCGCTCACTGACTCGCTGCGCTCGGTCGTTCGGCTGCGGCGAGCGGTATCAGCTCACTCAAAGGCGGTAATACGGTTATCCACAG
ATAACCCGCGAGAAGGCGAAGGAGCGAGTGACTGAGCGACGCGAGCCAGCAAGCCGACGCCGCTCGCCATAGTCGAGTGAGTTTCCGCCATTATGCCAATAGGTGTC

11,880 11,890 11,900 11,910 11,920 11,930 11,940 11,950 11,960 11,970 11,980

AATCAGGGGATAACGCAGGAAAGAACATGTGAGCAAAAGGCCAGCAAAAGGCCAGGAACCGTAAAAAGGCCGCGTTGCTGGCGTTTTTCCATAGGCTCGGCCCCCCT
TTAGTCCCCTATTGCGTCCTTTCTTGTACACTCGTTTTCCGGTCGTTTTCCGGTCCTTGGCATTTTTCCGGCGCAACGACCGCAAAAAGGTATCCGAGCCGGGGGA

pBR322_origin

11,990 12,000 12,010 12,020 12,030 12,040 12,050 12,060 12,070 12,080 12,090

GACGAGCATCACAAAAATCGACGCTCAAGTCAGAGGTGGCGAAACCCGACAGGACTATAAAGATACCAGGCGTTCCCCCCTGGAAGCTCCCTCGTGCGCTCTCCTGT
CTGCTCGTAGTGTTTTTAGCTGCGAGTTCAGTCTCCACCGCTTTGGGCTGTCCTGATATTTCTATGGTCCGCAAGGGGGGACCTTCGAGGGAGCACGCGAGAGGACA

pBR322_origin

12,100 12,110 12,120 12,130 12,140 12,150 12,160 12,170 12,180 12,190

TCCGACCCTGCCGCTTACCGGATACCTGTCCGCCTTTCTCCCTTCGGGAAGCGTGGCGCTTTCTCAATGCTCACGCTGTAGGTATCTCAGTTCGGTGTAGGTCGTTC
AGGCTGGGACGGCGAATGGCCTATGGACAGGCGGAAAGAGGGAAGCCCTTCGCACCGCGAAAGAGTTACGAGTGCGACATCCATAGAGTCAAGCCACATCCAGCAAG

pBR322_origin

12,200 12,210 12,220 12,230 12,240 12,250 12,260 12,270 12,280 12,290 12,300

GCTCCAAGCTGGGCTGTGTGCACGAACCCCCCGTTCAGCCCGACCGCTGCGCCTTATCCGGTAACTATCGTCTTGAGTCCAACCCGGTAAGACACGACTTATCGCCA
CGAGGTTCGACCCGACACACGTGCTTGGGGGGCAAGTCGGGCTGGCGACGCGGAATAGGCCATTGATAGCAGAACTCAGGTTGGGCCATTCTGTGCTGAATAGCGGT

pBR322_origin

12,310 12,320 12,330 12,340 12,350 12,360 12,370 12,380 12,390 12,400 12,410

CTGGCAGCAGCCACTGGTAACAGGATTAGCAGAGCGAGGTATGTAGGCGGTGCTACAGAGTTCTTGAAGTGGTGGCCTAACTACGGCTACACTAGAAGGACAGTATT
GACCGTCGTCGGTGACCATTGTCCTAATCGTCTCGCTCCATACATCCGCCACGATGTCTCAAGAACTTCACCACCGGATTGATGCCGATGTGATCTTCCTGTCATAA

pBR322_origin

12,420 12,430 12,440 12,450 12,460 12,470 12,480 12,490 12,500 12,510

TGGTATCTGCGCTCTGCTGAAGCCAGTTACCTTCGGAAAAAGAGTTGGTAGCTCTTGATCCGGCAAACAAACCACCGCTGGTAGCGGTGGTTTTTTTGTTTGCAAGC
ACCATAGACGCGAGACGACTTCGGTCAATGGAAGCCTTTTTCTCAACCATCGAGAACTAGGCCGTTTGTTTGGTGGCGACCATCGCCACCAAAAAAACAAACGTTCG
pBR322_origin
12,530 12,540 12,550 12,560 12,570 12,580 12,590 12,600 12,610 12,620

AGCAGATTACGCGCAGAAAAAAAGGATCTCAAGAAGATCCTTTGATCTTTTCTACGGGGTCTGACGCTCAGTGGAACGAAAACTCACGTTAAGGGATTTTGGTCATG
TCGTCTAATGCGCGTCTTTTTTTCCTAGAGTTCTTCTAGGAAACTAGAAAAGATGCCCCAGACTGCGAGTCACCTTGCTTTTGAGTGCAATTCCCTAAAACCAGTAC
pBR322_origin
12,630 12,640 12,650 12,660 12,670 12,680 12,690 12,700 12,710 12,720 12,730

AGATTATCAAAAAGGATCTTCACCTAGATCCTTTTAAATTAAAAATGAAGTTTTAAATCAATCTAAAGTATATATGAGTAAACTTGGTCTGACAGTTACCAATGCTT
TCTAATAGTTTTTCCTAGAAGTGGATCTAGGAAAATTTAATTTTTACTTCAAAATTTAGTTAGATTTCATATATACTCATTTGAACCAGACTGTCAATGGTTACGAA
Amp(R)
12,740 12,750 12,760 12,770 12,780 12,790 12,800 12,810 12,820 12,830 12,840

AATCAGTGAGGCACCTATCTCAGCGATCTGTCTATTTCGTTCATCCATAGTTGCCTGACTGCCCGTCGTGTAGATAACTACGATACGGGAGGGCTTACCATCTGGCC
TTAGTCACTCCGTGGATAGAGTCGCTAGACAGATAAAGCAAGTAGGTATCAACGGACTGACGGGCAGCACATCTATTGATGCTATGCCCTCCCGAATGGTAGACCGG
Amp(R)
12,850 12,860 12,870 12,880 12,890 12,900 12,910 12,920 12,930 12,940

BsaI
CCAGTGCTGCAATGATACCGCGAGACCCACGCTCACCGGCTCCAGATTTATCAGCAATAAACCAGCCAGCCGGAAGGGCCGAGCGCAGAAGTGGTCCTGCAACTTTA
GGTCACGACGTTACTATGGCGCTCTGGGTGCGAGTGGCCGAGGTCTAAATAGTCGTTATTTGGTCGGTCGGCCTTCCCGGCTCGCGTCTTCACCAGGACGTTGAAAT
Amp(R)
12,950 12,960 12,970 12,980 12,990 13,000 13,010 13,020 13,030 13,040 13,050

FspI
TCCGCCTCCATCCAGTCTATTAATTGTTGCCGGGAAGCTAGAGTAAGTAGTTCGCCAGTTAATAGTTTGCGCAACGTTGTTGCCATTGCTACAGGCATCGTGGTGTC
AGGCGGAGGTAGGTCAGATAATTAACAACGGCCCTTCGATCTCATTCATCAAGCGGTCAATTATCAAACGCGTTGCAACAACGGTAACGATGTCCGTAGCACCACAG
Amp(R)
13,060 13,070 13,080 13,090 13,100 13,110 13,120 13,130 13,140 13,150 13,160

ACGCTCGTCGTTTGGTATGGCTTCATTCAGCTCCGGTTCCCAACGATCAAGGCGAGTTACATGATCCCCCATGTTGTGAAAAAAAGCGGTTAGCTCCTTCGGTCCTC
TGCGAGCAGCAAACCATACCGAAGTAAGTCGAGGCCAAGGGTTGCTAGTTCCGCTCAATGTACTAGGGGGTACAACACTTTTTTTCGCCAATCGAGGAAGCCAGGAG
Amp(R)
13,170 13,180 13,190 13,200 13,210 13,220 13,230 13,240 13,250 13,260

PvuI

CGATCGTTGTCAGAAGTAAGTTGGCCGCAGTGTTATCACTCATGGTTATGGCAGCACTGCATAATTCTCTTACTGTCATGCCATCCGTAAGATGCTTTTCTGTGACT
GCTAGCAACAGTCTTCATTCAACCGGCGTCACAATAGTGAGTACCAATACCGTCGTGACGTATTAAGAGAATGACAGTACGGTAGGCATTCTACGAAAAGACACTGA

Amp(R)

13,270 13,280 13,290 13,300 13,310 13,320 13,330 13,340 13,350 13,360 13,370

BcgI

GGTGAGTACTCAACCAAGTCATTCTGAGAATAGTGTATGCGGCGACCGAGTTGCTCTTGCCCGGCGTCAATACGGGATAATACCGCGCCACATAGCAGAACTTTAAA
CCACTCATGAGTTGGTTCAGTAAGACTCTTATCACATACGCCGCTGGCTCAACGAGAACGGGCCGCAGTTATGCCCTATTATGGCGCGGTGTATCGTCTTGAAATTT

Amp(R)

13,380 13,390 13,400 13,410 13,420 13,430 13,440 13,450 13,460 13,470 13,480

AGTGCTCATCATTGGAAAACGTTCTTCGGGGCGAAAACTCTCAAGGATCTTACCGCTGTTGAGATCCAGTTCGATGTAACCCACTCGTGCACCCAACTGATCTTCAG
TCACGAGTAGTAACCTTTTGCAAGAAGCCCCGCTTTTGAGAGTTCCTAGAATGGCGACAACTCTAGGTCAAGCTACATTGGGTGAGCACGTGGGTTGACTAGAAGTC

Amp(R)

13,490 13,500 13,510 13,520 13,530 13,540 13,550 13,560 13,570 13,580

CATCTTTTACTTTCACCAGCGTTTCTGGGTGAGCAAAAACAGGAAGGCAAAATGCCGCAAAAAAGGGAATAAGGGCGACACGGAAATGTTGAATACTCATACTCTTC
GTAGAAAATGAAAGTGGTCGCAAAGACCCACTCGTTTTTGTCCTTCCGTTTTACGGCGTTTTTTCCCTTATTCCCGCTGTGCCTTTACAACTTATGAGTATGAGAAG

Amp(R)

13,600 13,610 13,620 13,630 13,640 13,650 13,660 13,670 13,680 13,690

CTTTTTCAATATTATTGAAGCATTTATCAGGGTTATTGTCTCATGAGCGGATACATATTTGAATGTATTTAGAAAAATAAACAAATAGGGGTTCCGCGCACATTTCC
GAAAAAGTTATAATAACTTCGTAAATAGTCCCAATAACAGAGTACTCGCCTATGTATAAACTTACATAAATCTTTTTATTTGTTTATCCCCAAGGCGCGTGTAAAGG

AmpR_promoter

13,700 13,710 13,720 13,730 13,740 13,750 13,760 13,770 13,780 13,790 13,800

AatII
ZraI

CCGAAAAGTGCCACCTGACGTCTAAGAAACCATTATTATCATGACATTAACCTATAAAAATAGGCGTATCACGAGGCCCTTTCGTC
GGCTTTTCACGGTGGACTGCAGATTCTTTGGTAATAATAGTACTGTAATTGGATATTTTTATCCGCATAGTGCTCCGGGAAAGCAG

13,810 13,820 13,830 13,840 13,850 13,860 13,870 13,880

25AEECQD_4160830_TFObindingsite_annotatedbyAF (...

CspCI SspI ApoI ApoI PsiI

CTAAATTGTAAGCGTTAATATTTTGTTAAAATTCGCGTTAAATTTTTGTTAAATCAGCTCATTTTTTAACCAATAGGCCGAAATCGGCAAAATCCCTTATAAATCAA
GATTTAACATTCGCAATTATAAAACAATTTTAAGCGCAATTTAAAAACAATTTAGTCGAGTAAAAAATTGGTTATCCGGCTTTAGCCGTTTTAGGGAATATTTAGTT

10 20 30 40 50 60 70 80 90 100

FspI FauI EarI

AAGAATAGACCGAGATAGGGTTGAGTGGCCGCTACAGGGCGCTCCCATTCGCCATTCAGGCTGCGCAACTGTTGGGAAGGGCGTTTCGGTGCGGGCCTCTTCGCTAT
TTCTTATCTGGCTCTATCCCAACTCACCGGCGATGTCCCGCGAGGGTAAGCGGTAAGTCCGACGCGTTGACAACCCTTCCCGCAAAGCCACGCCCGGAGAAGCGATA

110 120 130 140 150 160 170 180 190 200 210

PvuII BmrI

TACGCCAGCTGGCGAAAGGGGGATGTGCTGCAAGGCGATTAAGTTGGGTAACGCCAGGGTTTTCCCAGTCACGACGTTGTAAAACGACGGCCAGTGAGCGCGACGTA
ATGCGGTCGACCGCTTTCCCCCTACACGACGTTCCGCTAATTCAACCCATTGCGGTCCCAAAAGGGTCAGTGCTGCAACATTTTGCTGCCGGTCACTCGCGCTGCAT

220 230 240 250 260 270 280 290 300 310 320

CspCI BsaAI BanII PmlI SacI SfiI AflIII Eco53kI BsrFI NspI

ATACGACTCACTATAGGGCGAATTGGCGGAAGGCCGTCAAGGCCACGTGTCTTGTCCAGAGCTCCATCGGAAGCTCAATCATGTATTGCACCGGCATGTAGCTGTCA
TATGCTGAGTGATATCCCGCTTAACCGCCTTCCGGCAGTTCCGGTGCACAGAACAGGTCTCGAGGTAGCCTTCGAGTTAGTACATAACGTGGCCGTACATCGACAGT

TFObindingsite

330 340 350 360 370 380 390 400 410 420

TCTGGGTTTTGCAAGTCGTACCCTGAATGATGTTGAAGGCTCCAAGAAAAGAAAGAAGAAAGAAACAGGATGTACCCGTACCAGCACCATTCATACCTGTTTCCTGG
AGACCCAAAACGTTCAGCATGGGACTTACTACAACTTCCGAGGTTCTTTTCTTTCTTCTTTCTTTGTCCTACATGGGCATGGTCGTGGTAAGTATGGACAAAGGACC

TFO_bind...kedbyCs

TFObindingsite

430 440 450 460 470 480 490 500 510 520 530

KpnI SfiI Acc65I BpmI BsrBI FauI PvuII

GTACCTGGAGCACAAGACTGGCCTCATGGGCCTTCCGCTCACTGCCCGCTTTCCAGTCGGGAAACCTGTCGTGCCAGCTGCATTAACATGGTCATAGCTGTTTCCTT
CATGGACCTCGTGTTCTGACCGGAGTACCCGGAAGGCGAGTGACGGGCGAAAGGTCAGCCCTTTGGACAGCACGGTCGACGTAATTGTACCAGTATCGACAAAGGAA

540 550 560 570 580 590 600 610 620 630 640

GCGTATTGGGCGCTCTCCGCTTCCTCGCTCACTGACTCGCTGCGCTCGGTCGTTCGGGTAAAGCCTGGGGTGCCTAATGAGCAAAAGGCCAGCAAAAGGCCAGGAAC
CGCATAACCCGCGAGAGGCGAAGGAGCGAGTGACTGAGCGACGCGAGCCAGCAAGCCCATTTCGGACCCCACGGATTACTCGTTTTCCGGTCGTTTTCCGGTCCTTG

Col E1 origin

650 660 670 680 690 700 710 720 730 740

TaqαI
TaqI DrdI

CGTAAAAAGGCCGCGTTGCTGGCGTTTTTCCATAGGCTCCGCCCCCCTGACGAGCATCACAAAAATCGACGCTCAAGTCAGAGGTGGCGAAACCCGACAGGACTATA
GCATTTTTCCGGCGCAACGACCGCAAAAAGGTATCCGAGGCGGGGGGACTGCTCGTAGTGTTTTTAGCTGCGAGTTCAGTCTCCACCGCTTTGGGCTGTCCTGATAT

Col E1 origin

750 760 770 780 790 800 810 820 830 840 850

BssSαI
BssSI BciVI MmeI

AAGATACCAGGCGTTTCCCCCTGGAAGCTCCCTCGTGCGCTCTCCTGTTCCGACCCTGCCGCTTACCGGATACCTGTCCGCCTTTCTCCCTTCGGGAAGCGTGGCGC
TTCTATGGTCCGCAAAGGGGGACCTTCGAGGGAGCACGCGAGAGGACAAGGCTGGGACGGCGAATGGCCTATGGACAGGCGGAAAGAGGGAAGCCCTTCGCACCGCG

Col E1 origin

860 870 880 890 900 910 920 930 940 950 960

BaeGI
BseYI ApaLI

TTTCTCATAGCTCACGCTGTAGGTATCTCAGTTCGGTGTAGGTCGTTCGCTCCAAGCTGGGCTGTGTGCACGAACCCCCCGTTCAGCCCGACCGCTGCGCCTTATCC
AAAGAGTATCGAGTGCGACATCCATAGAGTCAAGCCACATCCAGCAAGCGAGGTTCGACCCGACACACGTGCTTGGGGGGCAAGTCGGGCTGGCGACGCGGAATAGG

Col E1 origin

970 980 990 1,000 1,010 1,020 1,030 1,040 1,050 1,060 1,070

MmeI AlwNI

GGTAACTATCGTCTTGAGTCCAACCCGGTAAGACACGACTTATCGCCACTGGCAGCAGCCACTGGTAACAGGATTAGCAGAGCGAGGTATGTAGGCGGTGCTACAGA
CCATTGATAGCAGAACTCAGGTTGGGCCATTCTGTGCTGAATAGCGGTGACCGTCGTCGGTGACCATTGTCCTAATCGTCTCGCTCCATACATCCGCCACGATGTCT

Col E1 origin

1,080 1,090 1,100 1,110 1,120 1,130 1,140 1,150 1,160 1,170

AcuI

GTTCTTGAAGTGGTGGCCTAACTACGGCTACACTAGAAGAACAGTATTTGGTATCTGCGCTCTGCTGAAGCCAGTTACCTTCGGAAAAAGAGTTGGTAGCTCTTGAT
CAAGAACTTCACCACCGGATTGATGCCGATGTGATCTTCTTGTCATAAACCATAGACGCGAGACGACTTCGGTCAATGGAAGCCTTTTTCTCAACCATCGAGAACTA

Col E1 origin

1,180 1,190 1,200 1,210 1,220 1,230 1,240 1,250 1,260 1,270 1,280

CCGGCAAACAAACCACCGCTGGTAGCGGTGGTTTTTTTGTTTGCAAGCAGCAGATTACGCGCAGAAAAAAAGGATCTCAAGAAGATCCTTTGATCTTTTCTACGGGG
GGCCGTTTGTTTGGTGGCGACCATCGCCACCAAAAAAACAAACGTTCGTCGTCTAATGCGCGTCTTTTTTTCCTAGAGTTCTTCTAGGAAACTAGAAAAGATGCCCC

Col E1 origin

1,290 1,300 1,310 1,320 1,330 1,340 1,350 1,360 1,370 1,380 1,390

BspHI

TCTGACGCTCAGTGGAACGAAAACTCACGTTAAGGGATTTTGGTCATGAGATTATCAAAAAGGATCTTCACCTAGATCCTTTTAAATTAAAAATGAAGTTTTAAATC
AGACTGCGAGTCACCTTGCTTTTGAGTGCAATTCCCTAAAACCAGTACTCTAATAGTTTTTCCTAGAAGTGGATCTAGGAAAATTTAATTTTTACTTCAAAATTTAG

1,400 1,410 1,420 1,430 1,440 1,450 1,460 1,470 1,480 1,490

AATCTAAAGTATATATGAGTAAACTTGGTCTGACAGTTACCAATGCTTAATCAGTGAGGCACCTATCTCAGCGATCTGTCTATTTCGTTCATCCATAGTTGCCTGAC
TTAGATTTCATATATACTCATTTGAACCAGACTGTCAATGGTTACGAATTAGTCACTCCGTGGATAGAGTCGCTAGACAGATAAAGCAAGTAGGTATCAACGGACTG

AmpR

1,500 1,510 1,520 1,530 1,540 1,550 1,560 1,570 1,580 1,590 1,600

AhdI BmrI BsrDI BpmI BsrFI

TCCCCGTCGTGTAGATAACTACGATACGGGAGGGCTTACCATCTGGCCCCAGTGCTGCAATGATACCGCGAGAACCACGCTCACCGGCTCCAGATTTATCAGCAATA
AGGGGCAGCACATCTATTGATGCTATGCCCTCCCGAATGGTAGACCGGGGTCACGACGTTACTATGGCGCTCTTGGTGCGAGTGGCCGAGGTCTAAATAGTCGTTAT

AmpR

1,610 1,620 1,630 1,640 1,650 1,660 1,670 1,680 1,690 1,700 1,710

AvaII NmeAIII AseI

AACCAGCCAGCCGGAAGGGCCGAGCGCAGAAGTGGTCCTGCAACTTTATCCGCCTCCATCCAGTCTATTAATTGTTGCCGGGAAGCTAGAGTAAGTAGTTCGCCAGT
TTGGTCGGTCGGCCTTCCCGGCTCGCGTCTTCACCAGGACGTTGAAATAGGCGGAGGTAGGTCAGATAATTAACAACGGCCCTTCGATCTCATTCATCAAGCGGTCA

AmpR

1,720 1,730 1,740 1,750 1,760 1,770 1,780 1,790 1,800 1,810

AclI
FspI BsrDI

TAATAGTTTGCGCAACGTTGTTGCCATTGCTACAGGCATCGTGGTGTCACGCTCGTCGTTTGGTATGGCTTCATTCAGCTCCGGTTCCCAACGATCAAGGCGAGTTA
ATTATCAAACGCGTTGCAACAACGGTAACGATGTCCGTAGCACCACAGTGCGAGCAGCAAACCATACCGAAGTAAGTCGAGGCCAAGGGTTGCTAGTTCCGCTCAAT

AmpR

1,820 1,830 1,840 1,850 1,860 1,870 1,880 1,890 1,900 1,910 1,920

AvaII PvuI

CATGATCCCCCATGTTGTGCAAAAAAGCGGTTAGCTCCTTCGGTCCTCCGATCGTTGTCAGAAGTAAGTTGGCCGCAGTGTTATCACTCATGGTTATGGCAGCACTG
GTACTAGGGGGTACAACACGTTTTTTCGCCAATCGAGGAAGCCAGGAGGCTAGCAACAGTCTTCATTCAACCGGCGTCACAATAGTGAGTACCAATACCGTCGTGAC

AmpR

1,930 1,940 1,950 1,960 1,970 1,980 1,990 2,000 2,010 2,020 2,030

ScaI
TatI BcgI

CATAATTCTCTTACTGTCATGCCATCCGTAAGATGCTTTTCTGTGACTGGTGAGTACTCAACCAAGTCATTCTGAGAATAGTGTATGCGGCGACCGAGTTGCTCTTG
GTATTAAGAGAATGACAGTACGGTAGGCATTCTACGAAAAGACACTGACCACTCATGAGTTGGTTCAGTAAGACTCTTATCACATACGCCGCTGGCTCAACGAGAAC

AmpR

2,040 2,050 2,060 2,070 2,080 2,090 2,100 2,110 2,120 2,130 2,140

XmnI
BsaHI AclI

CCCGGCGTCAATACGGGATAATACCGCGCCACATAGCAGAACTTTAAAAGTGCTCATCATTGGAAAACGTTCTTCGGGGCGAAAACTCTCAAGGATCTTACCGCTGT
GGGCCGCAGTTATGCCCTATTATGGCGCGGTGTATCGTCTTGAAATTTTCACGAGTAGTAACCTTTTGCAAGAAGCCCCGCTTTTGAGAGTTCCTAGAATGGCGACA

AmpR

2,150 2,160 2,170 2,180 2,190 2,200 2,210 2,220 2,230 2,240

ApaLI
AcuI
TaqαI BssSαI
TaqI BssSI BaeGI

TGAGATCCAGTTCGATGTAACCCACTCGTGCACCCAACTGATCTTCAGCATCTTTTACTTTCACCAGCGTTTCTGGGTGAGCAAAAACAGGAAGGCAAAATGCCGCA
ACTCTAGGTCAAGCTACATTGGGTGAGCACGTGGGTTGACTAGAAGTCGTAGAAAATGAAAGTGGTCGCAAAGACCCACTCGTTTTTGTCCTTCCGTTTTACGGCGT

AmpR

2,250 2,260 2,270 2,280 2,290 2,300 2,310 2,320 2,330 2,340 2,350

BsrBI
BsmAI
BcoDI
BciVI
EarI SspI BspHI

AAAAAGGGAATAAGGGCGACACGGAAATGTTGAATACTCATACTCTTCCTTTTTCAATATTATTGAAGCATTTATCAGGGTTATTGTCTCATGAGCGGATACATATT
TTTTTCCCTTATTCCCGCTGTGCCTTTACAACTTATGAGTATGAGAAGGAAAAAGTTATAATAACTTCGTAAATAGTCCCAATAACAGAGTACTCGCCTATGTATAA

AmpR

2,360 2,370 2,380 2,390 2,400 2,410 2,420 2,430 2,440 2,450 2,460

TGAATGTATTTAGAAAAATAAACAAATAGGGGTTCCGCGCACATTTCCCCGAAAAGTGCCAC
ACTTACATAAATCTTTTTATTTGTTTATCCCCAAGGCGCGTGTAAAGGGGCTTTTCACGGTG

2,470 2,480 2,490 2,500 2,510 2,520
