# Supplementary material for: ATRX deficiency drives aberrant type I interferon signalling through cGAS-dependent transcriptional dysregulation
Source: Nat Commun. 2026 Oct 8;17:11551. doi: 10.1038/s41467-026-78094-8 (PMC7619637; doi:10.1038/s41467-026-78094-8)
Supplement: Supplementary file 2 [file 41467_2026_78094_MOESM2_ESM.pdf]

Description of Additional Supplementary Files

Supplementary Data 1. Genomic coordinates of 444 interferon-stimulated genes

Columns 1/5/9/13 show chromosome number, columns 2/6/10/14 show gene Transcription Starting Site (TSS), columns 3/7/11/15 show TSS+1 nucleotide, and columns 4/8/12/16 show gene name.

Supplementary Data 2. Description of yeast strains used in this study

A Saccharomyces cerevisiae optimised-codon plasmid was designed to express human cDNA of ATRX. Plasmids were integrated into yJF1 budding yeast strain, resulting in yJF1-ATRX-WT, yJF1-ATRX-K1600R and yJF1-ATRX-Y1758C strains.
