# Supplementary material for: ATRX deficiency drives aberrant type I interferon signalling through cGAS-dependent transcriptional dysregulation
Source: Nat Commun. 2026 Oct 8;17:11551. doi: 10.1038/s41467-026-78094-8 (PMC7619637; doi:10.1038/s41467-026-78094-8)
Supplement: Supplementary file 3 [file 41467_2026_78094_MOESM5_ESM.pdf]

nature portfolio

Corresponding author(s): Marie-Therese El-Daher, Yanick Crow

Last updated by author(s): 26 August 2026

Reporting Summary

Nature Portfolio wishes to improve the reproducibility of the work that we publish. This form provides structure for consistency and transparency in reporting. For further information on Nature Portfolio policies, see our Editorial Policies and the Editorial Policy Checklist.

Statistics

For all statistical analyses, confirm that the following items are present in the figure legend, table legend, main text, or Methods section.

n/a	Confirmed	
☐	☒	The exact sample size (*n*) for each experimental group/condition, given as a discrete number and unit of measurement
☐	☒	A statement on whether measurements were taken from distinct samples or whether the same sample was measured repeatedly
☐	☒	The statistical test(s) used AND whether they are one- or two-sided<br>*Only common tests should be described solely by name; describe more complex techniques in the Methods section.*
☒	☐	A description of all covariates tested
☐	☒	A description of any assumptions or corrections, such as tests of normality and adjustment for multiple comparisons
☐	☒	A full description of the statistical parameters including central tendency (e.g. means) or other basic estimates (e.g. regression coefficient) AND variation (e.g. standard deviation) or associated estimates of uncertainty (e.g. confidence intervals)
☐	☒	For null hypothesis testing, the test statistic (e.g. *F*, *t*, *r*) with confidence intervals, effect sizes, degrees of freedom and *P* value noted<br>*Give P values as exact values whenever suitable.*
☒	☐	For Bayesian analysis, information on the choice of priors and Markov chain Monte Carlo settings
☒	☐	For hierarchical and complex designs, identification of the appropriate level for tests and full reporting of outcomes
☒	☐	Estimates of effect sizes (e.g. Cohen's *d*, Pearson's *r*), indicating how they were calculated

Our web collection on statistics for biologists contains articles on many of the points above.

Software and code

Policy information about availability of computer code

Data collection	not relevant to this study
Data analysis	Statistical analyses for all experiments, except sequencing data, were performed in GraphPad Prism v10 or v11. RNA-Seq read data were processed through the nf-core/rnaseq (v3.10.1) pipeline (doi: 10.5281/zenodo.1400710) using genome reference, GRCh38 (GRCh38.p13) and Ensembl gene models. We used the DESeq2 package (version 1.42.1) in R (version 4.3.3) to perform differential gene expression analysis. For CUT&Tag analysis, reads were processed with the nf-core/cutandrun pipeline (3.2.2) (doi: 10.5281/zenodo.10606804), implemented in Nextflow. For QuantSeq 3'mRNA-Seq, differential data analyses were performed following pipeline integration on the Bluebee genomics analysis platform, or by following the pipeline deposited on Zenodo with the following DOI: https://doi.org/10.5281/zenodo.22092978

For manuscripts utilizing custom algorithms or software that are central to the research but not yet described in published literature, software must be made available to editors and reviewers. We strongly encourage code deposition in a community repository (e.g. GitHub). See the Nature Portfolio guidelines for submitting code & software for further information.

Data

Policy information about availability of data

All manuscripts must include a data availability statement. This statement should provide the following information, where applicable:

- Accession codes, unique identifiers, or web links for publicly available datasets
- A description of any restrictions on data availability
- For clinical datasets or third party data, please ensure that the statement adheres to our policy

Sequencing data generated for this study are available through Gene Expression Omnibus (GEO) with the accession number GSE212923 [https://www.ncbi.nlm.nih.gov/geo/query/acc.cgi?acc=GSE212923]. This SuperSeries has been made public and it is composed of the SubSeries listed below: GSE344583 ,GSE344333, GSE212922, GSE337574, GSE329644, GSE337571.
Previously published transcriptional data were obtained GEO and processed data downloaded, and with the accession numbers GSE107878 [https://www.ncbi.nlm.nih.gov/geo/query/acc.cgi?acc=GSE107878], GSE12454 [https://www.ncbi.nlm.nih.gov/geo/query/acc.cgi?acc=GSE12454] and GSE100464 [https://www.ncbi.nlm.nih.gov/geo/query/acc.cgi?acc=GSE100464].
Source data are provided as a zip with this paper.

Research involving human participants, their data, or biological material

Policy information about studies with human participants or human data. See also policy information about sex, gender (identity/presentation), and sexual orientation and race, ethnicity and racism.

Reporting on sex and gender	Pedigree structure of two families are represented in Figure 1. Circles and squares indicate female and male family members respectively. Female and male refer to the sex (biological attribute). This paper reports three male patients with a rare genetic syndrome caused by mutations in the X-linked ATRX gene, female carriers are asymptomatic, likely due to complete skewing of X-inactivation. No sex- and gender-based analyses was performed in this study, as not relevant.
Reporting on race, ethnicity, or other socially relevant groupings	This paper reports three male patients with a rare genetic syndrome caused by mutations in the ATRX gene. No race, ethnicity, or other socially groupings were relevant for this study.
Population characteristics	Three young males exhibiting a clinical phenotype consistent with a diagnosis of ATR-X syndrome, accompanied by inflammation.
Recruitment	Patients conforming to a clinical diagnosis of an interferonopathy were recruited through the physician responsible for their care.
Ethics oversight	Clinical information and samples were obtained with informed consent. The study was approved by the Comite de Protection des Personnes (ID-RCB/EUDRACT: 2014-A01017-40) in France, and the Leeds (East) Research Ethics Committee (REC reference: 10/H1307/2; IRAS project ID: 62971) in the UK.

Note that full information on the approval of the study protocol must also be provided in the manuscript.

Field-specific reporting

Please select the one below that is the best fit for your research. If you are not sure, read the appropriate sections before making your selection.

[x] Life sciences [ ] Behavioural & social sciences [ ] Ecological, evolutionary & environmental sciences

For a reference copy of the document with all sections, see nature.com/documents/nr-reporting-summary-flat.pdf

Life sciences study design

All studies must disclose on these points even when the disclosure is negative.

Sample size	When using patients derived cells, sample size was limited by the number of patients available to us in whom we identified pathogenic variants. When using CRISPR-ed cell lines, experiments included at least three independent clones, representing biological repeats, and clones' repeats are indicated in the legends.
Data exclusions	No data were excluded from the analysis.
Replication	Experiments were performed with a minimum of three biological or multiple independent replicates, as detailed in figure legend, and were reliably reproduced (see Source Data File).
Randomization	No randomization was used in this study.
Blinding	Investigators were not blinded during data collection/analysis.

Reporting for specific materials, systems and methods

We require information from authors about some types of materials, experimental systems and methods used in many studies. Here, indicate whether each material, system or method listed is relevant to your study. If you are not sure if a list item applies to your research, read the appropriate section before selecting a response.

Materials & experimental systems

n/a	Involved in the study
☐	☒ Antibodies
☐	☒ Eukaryotic cell lines
☒	☐ Palaeontology and archaeology
☒	☐ Animals and other organisms
☐	☒ Clinical data
☒	☐ Dual use research of concern
☒	☐ Plants

Methods

n/a	Involved in the study
☒	☐ ChIP-seq
☒	☐ Flow cytometry
☒	☐ MRI-based neuroimaging

Antibodies

Antibodies used

Primary antibodies: mAb lgG XP lsotype Control (cell signaling technology 66362); ATRX (Novus Biologicals, NBP2-50111 for WB, or abcam ab97508 for CUT& Tag); DAXX (Sigma Prestige-HPA065779), H3.3 (Epicypher 13-0061); Ser172-TBK1 (cell signaling technology 5483); Tyr701-STAT1 (Cell signaling technology 7649); STAT1 (cell signaling technology 9176); Ser396-IRF3 (cell signaling technology 4947); IRF3 (cell signaling technology 11904); vinculin (cell signaling technology 13901); S366-STING (cell signaling technology 19781), STING (Millipore MAB7169), NF- B p65 (cell signaling technology 6956), Phospho-NF- B p65 (Ser536) (cell signaling technology 3031), cGAS (cell signaling technology 15102); MAVS (Enzo ALX-210-929); atubulin (Sigma T6074); Histone H3 (cell signaling technology 4499); Histone H2B (cell signaling technology 12364); MAP2 (abcam ab5392); NeuN (Millipore MAB377); OCT4 (Invitrogen MA1-104); Pax6 (Biolegend 901302); Nestin (Novus Biologicals NB300266); γ-H2AX (Millipore 05-636), and anifrolumab (MedChemExpress HY-199168).

Secondary antibodies: for immunofluorescence, Alexa Fluor conjugated antibodies were used and purchased from Life Technologies; for western blots, Goat anti-mouse lgG IRDye 800CW (Li-COR, 926-32210) and Goat anti-rabbit lgG IRDye 800CW (Li-COR, 926-32211) were used and purchased from Li-COR; for CUT&Tag, the secondary antibody Guinea Pig anti-Rabbit lgG (ABIN101961) was used and purchased from antibodies-online.

Validation

All commercially available antibodies have been validated by the manufacturer with supporting publications found on manufacturer websites.

Eukaryotic cell lines

Policy information about cell lines and Sex and Gender in Research

Cell line source(s)

BJ-hTERT fibroblasts were a kind gift from James Chen (University of Texas at Dallas); 293FT (Invitrogen, R70007); Interferon Regulatory Factor (IRF)-lnducible SEAP Reporter HEK293 (HEK-Blue'" ISG) cells (InvivoGen, hkb-isg-1); THP-1 WT (ATCC, TIB-202); THP-1 MAVS KO were a gift from Jan Rehwinkel. The iPSC QOLG-1 cell line was obtained from Kathy Evans (Centre for Genomic and Experimental Medicine Institute of Genetics and Cancer, Edinburgh, UK), acknowledging the Wellcome Trust Sanger Institute as the source ofthe HipSci HPSl1113i-qolg_l human induced pluripotent cell line (ECACC 77650145). All budding yeast cells are based on yJFl strain, genotypes and details of origin explained in the methods section.

Authentication

Cell lines were not authenticated.

Mycoplasma contamination

Cells were tested when obtained and negative for mycoplasma.

Commonly misidentified lines (See ICLAC register)

No commonly misidentified lines were used in this study.

Clinical data

Policy information about clinical studies

All manuscripts should comply with the ICMJE guidelines for publication of clinical research and a completed CONSORT checklist must be included with all submissions.

Clinical trial registration

not a clinical trial

Study protocol

Note where the full trial protocol can be accessed OR if not available, explain why.

Data collection

Describe the settings and locales of data collection, noting the time periods of recruitment and data collection.

Outcomes

Describe how you pre-defined primary and secondary outcome measures and how you assessed these measures.

Plants

Seed stocks

not a plant study

Novel plant genotypes

Describe the methods by which all novel plant genotypes were produced. This includes those generated by transgenic approaches, gene editing, chemical/radiation-based mutagenesis and hybridization. For transgenic lines, describe the transformation method, the number of independent lines analyzed and the generation upon which experiments were performed. For gene-edited lines, describe the editor used, the endogenous sequence targeted for editing, the targeting guide RNA sequence (if applicable) and how the editor was applied.

Authentication

Describe any authentication procedures for each seed stock used or novel genotype generated. Describe any experiments used to assess the effect of a mutation and, where applicable, how potential secondary effects (e.g. second site T-DNA insertions, mosiacism, off-target gene editing) were examined.
