# Supplementary material for: ATRX deficiency drives aberrant type I interferon signalling through cGAS-dependent transcriptional dysregulation
Source: Nat Commun. 2026 Oct 8;17:11551. doi: 10.1038/s41467-026-78094-8 (PMC7619637; doi:10.1038/s41467-026-78094-8)
Supplement: Supplementary file 4 [file 41467_2026_78094_MOESM6_ESM.pdf]

nature portfolio

Peer Review File

ATRX Deficiency Drives Aberrant Type I Interferon Signalling Through cGAS-Dependent Transcriptional Dysregulation

Corresponding Author: Dr Marie-Therese El-Daher

Parts of this Peer Review File have been redacted as indicated to remove third-party material.

This file contains all reviewer reports in order by version, followed by all author rebuttals in order by version.

Version 0:

Reviewer comments:

Reviewer #1

(Remarks to the Author)
El-Daher et al. present a detailed analysis of the mechanisms that account for increased expression of interferon-beta and type I interferon stimulated genes (ISG) in several individuals with a mutation, Y1758C, in the ATRX gene, an important epigenetic regulator. The two unrelated males demonstrated persistently increased ISGs and multiple cerebral calcifications, consistent with other interferonopathies.

Through a careful dissection of the intracellular pathways known to drive transcription of IFN-beta and ISGs, the authors identified a non-canonical pathway that involves cGAS but is independent of cGAMP and STING. Using fibroblasts and other cells expressing a knock-in of ATRX with the indicated mutation or knock-out of ATRX, they determine that the impact on transcription of ISGs involves a modification of the assembly of DAXX and H3.3, particularly at ISGs.

The manuscript is beautifully and clearly written, and experimental data are clearly presented. While the authors acknowledge that the specific mechanisms that account for the impact of the altered ATRX on DAXX assembly on chromatin at selected genes will require further investigation, their discussion provides a well-informed description of potential mechanisms.

With regard to the described clinical features of the two patients expressing the mutation, they are varied, as they are likely to be impacted by the distinct background genetic features of each of those individuals, as briefly indicated by the authors. However, the elevated ISGs in the setting of cerebral pathology, particularly the multiple calcifications, provide a common picture consistent with other interferonopathies. This paper will draw new attention to the diverse roles of cGAS in driving altered and sustained type I IFN and ISG transcription and should stimulate new investigation of the mechanisms that account for selectivity of chromatin regulators such as ATRX for the ISGs.

Reviewer #2

(Remarks to the Author)
Review comments
Summary

ATRX syndrome is an X-linked alpha-thalassaemia associated with intellectual disability and other neurological conditions, caused by dysfunction of the chromatin remodelling factor ATRX. The authors screened undiagnosed patients showing Aicardi-Goutières syndrome (AGS)-like features and persistent activation of the IFN-I pathway, and identified a maternally inherited ATRX p.Y1758C variant of unknown significance (VUS) in two unrelated individuals. AGS is a primordial segmental syndrome characterised by neurological abnormalities resulting from defective clearance of exogenous nucleic acids and toxic endogenous DNA/RNA species. AGS presents with white-matter disease and intracranial calcification, some of which overlap with features of ATRX syndrome. Recent studies have classified several neurodevelopmental disorders with defects in nucleic acid metabolism or DNA repair as type I interferonopathies, in which aberrant interferon signalling, rather than DNA damage itself, represents the primary pathogenic mechanism.

According to the manuscript, the main conclusions are as follows. First, ATRX p.Y1758C is proposed to be a disease-causing variant in the two AGS-like cases, acting through a cGAS-dependent but non-canonical, DNA damage sensor-independent route to activate IFN-I signalling. Second, the ATRX mutant retains chromatin-binding activity, but alters the expression of ATRX-regulated genes through a cGAS-dependent mechanism.

Major concerns
(1) Pathogenicity assignment of ATRX p.Y1758C
I am not fully convinced that ATRX p.Y1758C is clearly established as a pathogenic variant. ATRX protein levels in the patients P1/P2 are comparable to those of the wild type, arguing against a simple loss-of-function mechanism (Figure 2A). The authors propose that p.Y1758C acts as a gain-of-function variant that activates cGAS-dependent type I interferon signalling without canonical DNA sensing, while appearing hypomorphic in the context of X-linked recessive inheritance (as suggested by unaffected carrier mothers). This model is interesting but appears overly complicated and not fully supported by the data (Figure 1A). In addition, previous studies, some of which also cited by the authors, have reported that loss of ATRX suppresses rather than enhances type-I interferon responses (PMID: 37693131; PMID: 35939517; PMID: 37200088); the authors attribute this discrepancy to cell-type specificity. Even allowing for some degree of cell-type dependence, I find that in this study, the correlation between ATRX genotype and the extent of type I interferon induction among patient-derived primary cells (P1-P6) is not entirely consistent (Figure 2B/C). Given that the two cases are unrelated and the variant is not de novo, it is also important to consider the possibility that p.Y1758C exists in the population at a low frequency and that the interferon phenotype may be caused by other genetic factors. A dominant-negative mechanism appears unlikely, given the recessive mode of inheritance and the absence of symptoms in carrier females. Before concluding pathogenicity, please exclude alternative genetic causes typical of AGS and related interferonopathies.
I recommend performing RNA-seq and immunoblotting to verify that canonical AGS genes (e.g., SAMHD1, TREX1, RNASEH2A/C) are expressed at normal levels, and conducting whole-genome sequencing to rule out pathogenic or likely pathogenic variants, including deep intronic or regulatory variants, in AGS-related genes and other interferonopathy-associated loci.

(2) Genotype–phenotype resolution for ATRX deficiency versus p.Y1758C
The authors propose that p.Y1758C induces cell type-restricted activation of type I interferon via a cGAS-dependent but STING/IRF3-independent mechanism, while ATRX chromatin binding remains intact and only a subset of ATRX-bound genes is misregulated. This model derives from the conventional expectation that a missense ATRX variant with preserved expression and chromatin binding behaves as a partial loss-of-function allele, producing a milder phenotype resembling ATRX deficiency. However, in this case, the two p.Y1758C patients are proposed to exhibit interferon activation that could, in principle, result in a phenotype more severe than that associated with classical hypomorphic ATRX mutations.

I suggest performing in vivo validation, given the systemic involvement of ATRX and AGS. Although technically challenging, generating an isogenic animal model (p.Y1758C knock-in) would be compelling to establish X-linked recessive inheritance, tissue specificity, and neuroinflammatory phenotypes, and to test whether the IFN-I response exceeds that observed with ATRX haploinsufficiency or hypomorphic mutations.

I also propose providing a clearer side-by-side comparison of WT, ATRX knockout (conditional), and p.Y1758C knock-in cells in the same genetic background across multiple cell types, quantifying IFN-I output (associated protein and gene expressions) together with chromatin binding metrics (ATRX-regulated genes, DAXX and H3.3 occupancy) at the same loci. This would help determine whether p.Y1758C represents a separable function of ATRX rather than simply recapitulating loss-of-function.

To further strengthen the mechanistic basis for the conclusion that IFN-I activation is cGAS-dependent yet independent of canonical DNA-sensing in the cells with ATRX p.Y1758C mutation, additional experiments could be considered. These may include investigating conventional DNA damage response pathways implicated in AGS and related interferonopathies, to determine whether specific genotoxic stressors or repair deficiencies contribute to IFN activation in this context.

Overall, this study provides an intriguing observation linking ATRX dysfunction to neuroinflammation via a non-canonical role of cGAS in transcriptional regulation. The work will be of interest to researchers in the field. However, publication in Nature Communications requires stronger evidence on two fronts. First, a more rigorous demonstration that ATRX p.Y1758C is the causal variant in these patients, independent of perturbations in other AGS-related pathways. Second, a clearer genotype-phenotype relationship that distinguishes the effects of p.Y1758C from ATRX loss-of-function mutations, supported by in vivo animal models and deeper mechanistic insight. Pending these additions, I find the current mechanistic and genetic evidence insufficient to support acceptance.

Reviewer #3

(Remarks to the Author)
In this manuscript, El-Daher et al. suggest a novel mechanistic link between ATRX deficiency and type I interferon activation mediated via a non-canonical cGAS-dependent pathway. The authors identify a recurrent ATRX Y1758C mutation in two unrelated patients with neuroinflammatory phenotypes, then demonstrate that ATRX deficiency contributes to type I interferon-mediated inflammation in a cGAS-dependent manner, accompanied by chromatin remodeling characterized by occupancy of the histone chaperone DAXX and the histone variant H3.3. The experimental design and results are generally robust, and the manuscript is clearly structured. This manuscript presents an intriguing and original concept that ATRX

deficiency enhances type I IFN signaling through a non-canonical cGAS-dependent pathway. However, the proposed mechanism currently lacks sufficient evidence, and the clinical relevance remains limited. A more refined elucidation of the noncanonical functions of cGAS, along with additional functional validation, would substantially strengthen the completeness and impact of the study.

Major Points

1) In the Discussion section, the authors have appropriately addressed the significance and limitations of their study as follows: 1) How this mutation affects ATRX activity requires further study…….We speculate that the Y1758C substitution might also affect the helicase activity of ATRX, and thereby its ability to remodel chromatin function 2) Elucidating the pathways involved in the altered dynamics of DAXX and H3.3 at chromatin, and determining if these changes work alongside the recruitment of STATs/IRFs to boost the expression of inflammatory genes, will be necessary. 3)……should explore how cGAS contributes to changes in chromatin remodelling consequent upon ATRX deficiency. However, these key questions are essential to explaining the conclusions of this study. To substantiate the work's novelty, at least part of these discussion points should be experimentally validated.

2) Partial effect of IFNAR blockade requires deeper interpretation. Anifrolumab treatment reduces only 35 of 183 ISGs (Fig. 3B), suggesting the effect of IFNAR blockade is very partial. This may weaken confidence in the contribution of IFNAR downstream signaling to ATRX-mediated inflammation. In addition, one consistent finding from this study is that pSTAT1 is increased. Thus, the authors need to address 1) the protein level of IFNs, 2) the binding sites of STAT1 (ChIP-qPCR or ChIP-seq).

3) In relation to the above comments, the rigor of the experiment on the data in Fig. 3F needs to be checked, and the results need to be reinterpreted. 1) There are definitely signals for all proteins in the 7th lane, which should be blank. 2) The last lane in Fig. 3A is agonist-treated ATRX WT cells, but it is unclear what the last lane in Fig. 3F is. In fig. 3F, the amount of ATRX is significantly reduced, and while pSTAT1 increases, the amount of STAT1 remains unchanged. This requires further elucidation.

4) Still, the clinical relevance remains limited. In samples from patients with p3 and p4, expression of ATRX was significantly reduced, but ISG expression was not increased, so the main mechanism proposed by the authors is unlikely to apply to all patients. Thus, the conclusion that this finding defines a "novel interferonopathy" appears overly definitive and should be presented with greater caution. Otherwise, the key findings should be reconfirmed using iPSC-derived neurons, as the authors note: "Moreover, the immunogenicity of ATRX deficiency is apparently cell type specific."

5) It would be important to quantitatively assess whether the observed changes in DAXX and H3.3 are restricted to specific ISGs or represent a more global phenomenon across the transcriptome.

Minor Points

1) The authors utilized a curated list of 444 ISGs. Please provide the source, curation criteria in the Methods.

2) It should be clearly demonstrated whether the expression levels of WT and D319A cGAS in Fig. 4E–F are comparable, as differences in expression could significantly affect the interpretation of the results. In addition, the use of cGAS-D319A mutant supports enzymatic independence, but residual or compensatory effects cannot be fully excluded. Quantification of nuclear localization, stability, and expression levels between WT and D319A constructs should be provided to exclude artifacts.

Version 1:

Reviewer comments:

Reviewer #1

(Remarks to the Author)

This revised manuscript from El-Daher et al., along with additional data and detailed responses to reviewer comments, satisfactorily supports the impact of the ATRX mutation on transcriptional regulation, with data supporting disruption of ATPase and translocase activities and reduced DAXX binding that particularly impacts type I interferon-regulated genes - in contrast to EMT-regulated genes. The authors have also incorporated a third patient with a distinct mutation in ATRX in the manuscript.

The authors have provided an extensive response to reviewer #2, making the case that the clinical presentation of the featured patients is not attributable to mutations in genes associated with Aicardi-Goutieres syndrome.

They have also responded to each of the comments of reviewer 3, with acknowledgement that the effects of the ATRX mutation are only partial, with apparent contributions of cell intrinsic factors to degree of activation of the interferon-regulated genes as well as the stochastic nature of these and other ATRX mutations, resulting in variable clinical phenotypes. As requested by the reviewer, the authors have documented the ATPase and translocase functions that are impaired by the mutation, and they have provided a cleaner immunoblot for figure 3.f. All together, the authors have provided a detailed and extensive analysis of the implications of the ATRX mutation for gene regulation, the dependence of its effects on cGAS but not on signaling downstream from cGAS, and its actions related to chromatin remodeling. While there remain opportunities for further characterization of the interactions of cGAS, ATRX and DAXX with regard to epigenetic remodeling and gene transcription, the impact of the mutation on the interferon pathway, even if manifest to different degrees in distinct individual patients, provides new insights into the interactions required for transcriptional regulation of type I interferon, with implications for innate immune activation contributing to pathology, particularly in the CNS.

Reviewer #2

(Remarks to the Author)
After carefully reviewing the authors' rebuttal, I remain unconvinced that the novelty of the ATRX-Y1758C mutation -namely that it causes disease through a mechanism distinct from canonical ΔATRX-null type deficiency- has been adequately demonstrated.
To justify publication in Nature Communications, the authors need to show at the molecular level that the disease mechanism associated with ATRX-Y1758C is functionally distinguishable from ATRX-KO mutations (null variants such as stopgain or frameshift mutations abolishing protein expression) as well as from previously reported ATRX missense mutations.

A key issue is whether the disease mechanism in the alpha-thalassaemia patients P1/P2 (ATRX-Y1758C) is fundamentally distinct from that in the ATRX syndrome patients P4/P7 used as controls, and from previously reported ATRX cases (and models).
As also noted by the authors, the impact of ATRX abnormalities on IFN responses varies widely depending on cell type and context. Consistent with this, prior studies report both suppression and activation of IFN signalling upon ATRX loss, indicating that the field remains controversial (Floyd, J Clin Invest. 2023; Wang, Am J Cancer Res 2023; Whitford, Nature Communications 2025).

This variability is clearly illustrated by the comparison between P4 and P7 in Fig1. Fig.1a and Fig.1b show no clear correlation between ATRX protein levels (Fig. S3) and IFN score or IFNB1 expression. Although both P4 (W222C, associated with protein instability) and P7 (a frameshift null mutation) lack detectable ATRX protein (ΔATRX), their IFN responses are opposite (P4 suppressed, P7 enhanced). Thus, IFN transcriptional responses are not uniquely determined by ATRX protein status, and IFN readouts should be interpreted with caution as mechanistic endpoints. While these data support context-dependent regulation of IFN signalling downstream of ATRX dysfunction, they do not by themselves establish an ATRX-Y1758C-specific disease mechanism. If IFN responses cannot be clearly distinguished between ΔATRX-null mutations and ATRX-Y1758C, support for novelty specific to ATRX-Y1758C remains limited.

The authors classify ATRX-Y1758C as a loss-of-function mutation. My earlier use of the term 'gain-of-function' reflected wording rather than intent. The key point is that ATRX-Y1758C retains protein stability and chromatin binding comparable to wild-type ATRX, yet activates IFN signalling in a cGAS-dependent, canonical-pathway-independent manner. While the authors interpret this as a hypomorphic loss-of-function, it remains unclear from the data presented how ATRX-Y1758C is functionally distinct from previously reported ΔATRX-null, or hypomorphic mutations.

This distinction lies at the core of the manuscript. The novelty depends on demonstrating that ATRX-Y1758C differs mechanistically from both conventional missense mutations and complete loss-of-functon(ΔATRX). From this perspective, a clear comparison between ATRX-KI and ATRX-KO models (and patient cells) is essential.
If DEG analyses (and other cellular/molecular experiments as well) reveal differences in IFN responses or activation mechanisms between KI and KO, direct KI-KO or WT/KI/KO comparisons should be presented (e.g. S5fg/S7b). Conversely, if transcriptional responses are largely shared, the rationale for emphasising Y1758C as a special case is weak.

Despite the availability of patient-derived primary cells, no experiments directly compare control cells, P1/P2 (ATRX-Y1758C), and P4/P7 (ΔATRX) within the same experimental framework. For example, Fig.4a and Fig.S6a analyse patient-derived fibroblasts but exclude ΔATRX-null cases such as P4 and P7, making it difficult to directly assess whether the reported phenotypes extend to canonical ATRX deficiency.

Concerns regarding the pathogenicity of ATRX-Y1758C are further strengthened by the absence of basic validation experiments that could be easily feasible given the available patient material, such as whole-genome sequencing or western blot analyses of AGS relevant factors to exclude alternative diagnoses of interferonopathy. These issues were not resolved in the revison, where the authors state that additional experiments are unnecessary based on their prior experience. I fully recognise the authors' strong standing in this field; however, when claiming a non-canonical or atypical case, differential diagnosis remains important to exclude other possibilities.

Finally, if ATRX-Y1758C constitutively enhances IFN signalling, the functional dominance between wild-type ATRX and ATRX-Y1758C requires careful consideration. Although the carrier mother is reported to be unaffected (heterozygous), the relative dominance of wild-type versus mutant ATRX in the IFN signalling pathway has not been clearly indicated, and no explanation is provided for the absence of phenotype in the carrier.

For these reasons, I remain unconvinced that ATRX-Y1758C represents a mechanistically novel disease entity distinct from conventional ΔATRX-null cases. Although acceptance might be considered if additional evidence were provided, the current data leave open the possibility that key conclusions regarding pathogenicity and disease mechanism may not be robust.

Reviewer #3

(Remarks to the Author)
The revised manuscript has been significantly improved, and the authors have successfully addressed the key concerns raised during the initial review. The additional data and clarified explanations provided in this version have strengthened the overall impact and clarity of the study.

Reviewer #4

(Remarks to the Author)
This paper by A El-Daher and colleagues describes three patients with specific missense mutations in the ATRX ATPase domain. As a specific feature of these ATRX patients they report on a persistent upregulation of interferon-stimulated gene expression in the peripheral blood and in patient-derived fibroblasts. They go on to use CRISPR/Cas-modified cells to demonstrate that cGAS is relevant for ISG upregulation, however, it is postulated that cGAS mediates this effect via a non-conventional nuclear mechanism that is independent from DNA sensing.
I have the following concerns with the results of this paper:
1. Although the identification of ATRX patients with upregulation of ISGs and IFNb is interesting, the increases as reported in figure 1 are rather weak. The IFN scores are in the range of 3 to a maximum of 6fold which is considerably lower compared to a panel of patients with well characterized interferonopathies (see eg PMID: 24183309). The authors associate these slightly increased scores to features of neuroinflammation detected in the respective patients. The only potentially causal link is the response to ruxolitinib as observed in a singular patient. Furthermore, only a subset of ATRX patients exhibits signs of ISG upregulation.
2. Mechanistically, the authors propose that ATRX deficiency disrupts the pattern of DAXX and H3.3 and this critically depends on a non-canonical nuclear role of cGAS. However, it remains entirely unclear how cGAS mediates this effect. Furthermore, the differences shown in figure 3 are not very impressive.
3. To my opinion, alternative mechanisms could account for the increased ISG scores observed in this study and the authors need to address this:
- ATRX is well known to act as a repressor of endogenous retroviruses (e.g. PMID: 26012739). Thus, loss of ATRX function may upregulate ERVs and this, in turn, could lead to ISG upregulation independent of RIG1 or cGAS (see PMID: 26941318).
- Loss of ATRX is known to be the prerequisite for the development of ALT. ALT, in turn, generates extrachromosomal telomere repeat DNA which acts as a potent stimulator of IFNb and the type I IFN response (PMID: 29106411).
Both of these alternative mechanisms might also explain why only some ATRX patients show signs of ISG upregulation since ERVs are very diverse between individuals and the development of ALT also depends on (so far unknown) secondary events (see PMID: 40292316). In this context, it might turn out to be problematic that the authors used telomerase immortalized BJ fibroblasts for all their CRISPR/Cas engineering since expression of telomerase affects the formation of c-circle DNA known to act pro-inflammatory. Furthermore, CRISPR Cas engineering requires a prolonged selection of cells. Thus, not ATRX deficiency by itself but secondary events fostered by the well documented genome destabilizing effects of ATRX depletion (PMID: 40292316) might account for the “stochastic” occurrence of ISG upregulation as described in this study.

Version 2:

Reviewer comments:

Reviewer #2

(Remarks to the Author)
In the final revision, there are few substantive additional experiments or analyses addressing the major concerns raised previously by Reviewers#2 and #4.

This revision partially addresses Reviewer#4's concern regarding ALT/telomere DNA sensing. However, I do not consider the other concerns shared by Reviewers#2 and #4 to have been substantially resolved. The authors' response consists mainly of post hoc explanations in the rebuttal, and the central concerns of Reviewers#2 and #4 are not sufficiently addressed.

At present, the data support only a limited conclusion: cGAS-dependent and cGAMP/STING-independent ISG transcriptional dysregulation is observed in some cells and patients with ATRX dysfunction. These data do not establish that this is a phenotype specific to ATRX-Y1758C, nor do they justify generalisation to all patients with ATR-X syndrome. An overlooked variant in a known interferonopathy-related gene has not been fully excluded.

By contrast, stronger conclusions such as "ATRX-Y1758C defines a mechanistically distinct new interferonopathy", "ATRX deficiency generally drives type I interferon signalling", or "cGAS directly regulates ATRX-mediated chromatin remodelling" are not sufficiently supported by the current data.

Therefore, I cannot support acceptance in the current form. At minimum, the title, abstract and conclusion should be substantially toned down, and the unresolved alternative mechanisms and limitations in differential diagnosis should be clearly stated. If additional experiments are requested, WGS and RNA-seq of P1/P2 should at least be performed, together with confirmation of the expression of representative AGS/interferonopathy-related proteins.

The final decision as to whether the ATRX-Y1758C variant and its associated phenotype have sufficient novelty for publication in Nature Communications should then be left to the editor.

Concerns shared by Reviewers#2 and #4
1. A novel disease mechanism specific to ATRX-Y1758C has not been demonstrated

One of the fundamental concerns shared by the Reviewers is that it remains unclear whether the central claim of this study is a novel disease mechanism specific to ATRX-Y1758C, or an IFN abnormality associated with ATRX dysfunction that may also be observed more generally in known ATRX-deficient conditions.

In the rebuttal, the authors state that they do not claim Y1758C to be mechanistically distinct. The title and abstract still state "ATRX Deficiency Drives Aberrant Type I Interferon Signalling Through cGAS-Dependent Transcriptional Dysregulation", and strongly describe ATRX loss-of-function mutations as driving enhanced type I interferon signalling. The novelty is clearly different depending on whether ATRX-Y1758C can be defined as a new disease entity, or whether IFN abnormalities represent an additional phenotype that may occur more generally in ATRX deficiency. In addition, several previous studies, including Floyd JCI (2023), have reported impaired cGAS/STING signalling in ATRX deficiency, which is opposite to the authors' claim, as also stated in my previous comments. Therefore, a rigorous assessment is required, and the authors' explanation based on cell-type dependence is not sufficient. If this explanation is accepted, it becomes difficult to positively interpret experimental results obtained using BJ-hTERT knockout cells other than patient-derived cells. A mechanism specific to Y1758C has not been shown. Rather, the data should be interpreted as part of pre-existing ATRX dysfunction. Thus, the novelty has been weakened in the rebuttal, but this limitation is not sufficiently reflected in the manuscript.

2. The relationship among ATRX genotype, ATRX protein level and IFN response remains unresolved

Both reviewers raised concerns that ATRX protein levels or mutation type do not correlate with IFN score or IFNB1 expression. The manuscript states that P1/P2 retain ATRX protein expression but show increased IFN score and IFNB1 expression. It also states that, among primary fibroblasts from patients with classical ATR-X syndrome, IFN score and IFNB1 expression were also increased in two of four cases. The discrepancy in loss of function between ATRX-Y1758C and canonical ATRX mutations is not sufficiently explained at the level of patient-cell phenotypes. The authors mention genetic background and stochastic chromatin accessibility/gene expression in the rebuttal and Discussion, but this remains an unvalidated explanation rather than an experimentally supported conclusion. Therefore, the conclusion that ATRX deficiency generally drives IFN signalling should be treated with caution. Again, given that data contradictory to the authors' model have been reported, including Floyd JCI (2023), the authors should avoid explaining critical patient-cell phenotypes mainly by differences in cell type.

3. The mechanism of the non-canonical nuclear function of cGAS remains unresolved

Both reviewers were concerned that it remains unclear how cGAS alters DAXX/H3.3 distribution or ISG transcription. The authors explain cGAS dependence and cGAMP/STING independence in the rebuttal. However, this is an interpretation of data that had already been presented previously, and the current revision does not newly clarify the direct mechanism of cGAS. The authors state that how cGAS contributes to chromatin remodelling associated with ATRX deficiency remains to be addressed in future studies. The issue is not sufficiently resolved. The mechanistic conclusion that cGAS regulates transcription through ATRX/DAXX/H3.3 remains not directly demonstrated.

4. Alternative mechanisms have not been sufficiently excluded

Both reviewers raised concerns that the observed IFN upregulation may not be due to ATRX-Y1758C itself, but could instead be explained by other interferonopathy causes, ERV/repeats, ALT/telomere DNA, genome instability, or cell culture/CRISPR selection artifacts. The only addition in the current manuscript is the statement in the Discussion regarding Telomere-FISH, namely that cytoplasmic telomere foci were not detected and that canonical DNA sensing through cytosolic leakage of telomere fragments is therefore unlikely. However, this only addresses part of the possibility of ALT/telomere-derived DNA sensing, and other experimental examinations are not sufficiently presented. Many of the major alternative mechanisms raised by Reviewers#2 and #4 have not been experimentally excluded.

5. The link between the clinical phenotype and IFN signature remains insufficiently established

Both reviewers raised concerns that the manuscript overstates the extent to which the IFN signature explains neuroinflammation or systemic inflammation in patients with ATR-X syndrome. The manuscript shows persistent ISG upregulation in P1/P2 and describes systemic inflammation, elevated CSF neopterin and a treatment course including ruxolitinib in P3. However, P3 is also described as having a diagnosis of subcutaneous panniculitis-like T-cell lymphoma, making it difficult to attribute the inflammatory phenotype simply to the ATRX/IFN axis. The manuscript shows that an IFN signature is observed in a subset of patients with ATR-X syndrome, but it does not sufficiently demonstrate that this is the main cause of neuroinflammation or systemic inflammation.

Concerns specific to Reviewer#2

6. Exclusion of other AGS/interferonopathy causes for the IFN phenotype in P1/P2

The most important concern of Reviewer#2 on this point is whether the IFN phenotype in P1/P2 can be attributed to ATRX-Y1758C, rather than to an independent defect in another AGS/interferonopathy-related gene. Genetic tests have increasingly revealed cases in which more than one rare disorder coexists in the same patient. The manuscript states that WES identified no putative mutations other than ATRX. However, WGS and RNA-seq were not performed, and even confirmation of the expression of some interferonopathy-related proteins, which does not appear technically difficult, was not performed. The authors argue that it is not practical to comprehensively examine for more than 50 interferonopathy-related genes. However, the concerns could be addressed by only using the available patient materials. WGS using patient gDNA may identify overlooked variants in known interferonopathy-related genes. RNA-seq could also directly assess reduced

expression or splicing abnormalities of these genes. Deep intronic variants, regulatory variants, structural variants and splicing abnormalities cannot be sufficiently detected by WES. Therefore, the absence of obvious coding pathogenic variants in AGS1-9 by WES is not sufficient to exclude alternative interferonopathy causes. The authors provide explanations in the rebuttal, but no experimental differential diagnosis has been added.

7. Functional distinction between Y1758C and ATRX-null/previously reported missense mutations

Reviewer#2 asked whether Y1758C can be distinguished from ATRX-KO/null or previously reported missense mutations. The manuscript presents comparisons among WT, Y1758C-KI and ATRX-KO in an isogenic BJ-hTERT model, showing increased ISG/IFNB1 expression and shared DEGs in both KI and KO cells. This is a useful comparison, but it does not show that Y1758C has a novel mechanism distinct from ATRX-null. Rather, the manuscript suggests that Y1758C largely recapitulates ATRX loss of function, similarly to other ATRX-deficient states. Therefore, a disease mechanism specific to Y1758C cannot be claimed.

Reviewer #5

(Remarks to the Author)
In light of the comments by reviewer 1 and 3 and the extensive revisions to address the comments by reviewer 2 I would support publication of the manuscript. The key issues outlined regarding mechanism can be discussed in the discussion sections and highlight the potential mechanisms cGAS uses to drive the IFN phenotype.

Reviewer #6

(Remarks to the Author)
The authors carefully addressed Reviewer 4´s concerns and clarified potential misunderstandings.

Open Access This Peer Review File is licensed under a Creative Commons Attribution 4.0 International License, which permits use, sharing, adaptation, distribution and reproduction in any medium or format, as long as you give appropriate credit to the original author(s) and the source, provide a link to the Creative Commons license, and indicate if changes were made.
In cases where reviewers are anonymous, credit should be given to 'Anonymous Referee' and the source.
The images or other third party material in this Peer Review File are included in the article’s Creative Commons license, unless indicated otherwise in a credit line to the material. If material is not included in the article’s Creative Commons license and your intended use is not permitted by statutory regulation or exceeds the permitted use, you will need to obtain permission directly from the copyright holder.
To view a copy of this license, visit https://creativecommons.org/licenses/by/4.0/

Reviewer #1 (Remarks to the Author):

El-Daher et al. present a detailed analysis of the mechanisms that account for increased expression of interferon-beta and type I interferon stimulated genes (ISG) in several individuals with a mutation, Y1758C, in the ATRX gene, an important epigenetic regulator. The two unrelated males demonstrated persistently increased ISGs and multiple cerebral calcifications, consistent with other interferonopathies.

Through a careful dissection of the intracellular pathways known to drive transcription of IFN-beta and ISGs, the authors identified a non-canonical pathway that involves cGAS but is independent of cGAMP and STING. Using fibroblasts and other cells expressing a knock-in of ATRX with the indicated mutation or knock-out of ATRX, they determine that the impact on transcription of ISGs involves a modification of the assembly of DAXX and H3.3, particularly at ISGs.

The manuscript is beautifully and clearly written, and experimental data are clearly presented. While the authors acknowledge that the specific mechanisms that account for the impact of the altered ATRX on DAXX assembly on chromatin at selected genes will require further investigation, their discussion provides a well-informed description of potential mechanisms.

With regard to the described clinical features of the two patients expressing the mutation, they are varied, as they are likely to be impacted by the distinct background genetic features of each of those individuals, as briefly indicated by the authors. However, the elevated ISGs in the setting of cerebral pathology, particularly the multiple calcifications, provide a common picture consistent with other interferonopathies. This paper will draw new attention to the diverse roles of cGAS in driving altered and sustained type I IFN and ISG transcription and should stimulate new investigation of the mechanisms that account for selectivity of chromatin regulators such as ATRX for the ISGs.

Response to Reviewer #1:

We thank the reviewer for their highly favourable opinion.

Reviewer #2 (Remarks to the Author):

Review comments

Summary

ATRX syndrome is an X-linked alpha-thalassaemia associated with intellectual disability and other neurological conditions, caused by dysfunction of the chromatin remodelling factor ATRX. The authors screened undiagnosed patients showing Aicardi-Goutières syndrome (AGS)-like features and persistent activation of the IFN-I pathway, and identified a maternally inherited ATRX p.Y1758C variant of unknown significance (VUS) in two unrelated individuals. AGS is a primordial segmental syndrome characterised by neurological abnormalities resulting from defective clearance of exogenous nucleic acids and toxic endogenous DNA/RNA species. AGS presents with white-matter disease and intracranial calcification, some of which overlap with features of ATRX syndrome. Recent studies have classified several neurodevelopmental disorders with defects in nucleic acid metabolism or DNA repair as type I interferonopathies, in which aberrant interferon signalling, rather than DNA damage itself, represents the primary pathogenic mechanism.

According to the manuscript, the main conclusions are as follows. First, ATRX p.Y1758C is proposed to be a disease-causing variant in the two AGS-like cases, acting through a cGAS-dependent but non-canonical, DNA damage sensor-independent route to activate IFN-I signalling. Second, the ATRX mutant retains chromatin-binding activity, but alters the expression of ATRX-regulated genes through a cGAS-dependent mechanism.

Major concerns

(1) Pathogenicity assignment of ATRX p.Y1758C

I am not fully convinced that ATRX p.Y1758C is clearly established as a pathogenic variant. ATRX protein levels in the patients P1/P2 are comparable to those of the wild type, arguing against a simple loss-of-function mechanism (Figure 2A). The authors propose that p.Y1758C acts as a gain-of-function variant that activates cGAS-dependent type I interferon signalling without canonical DNA sensing, while appearing hypomorphic in the context of X-linked recessive inheritance (as suggested by unaffected carrier mothers). This model is interesting but appears overly complicated and not fully supported by the data (Figure 1A). In addition, previous studies, some of which also cited by the authors, have reported that loss of ATRX suppresses rather than enhances type-I interferon responses (PMID: 37693131; PMID: 35939517; PMID: 37200088); the authors attribute this discrepancy to cell-type specificity. Even allowing for some degree of cell-type dependence, I find that in this study, the correlation between ATRX genotype and the extent of type I interferon induction among patient-derived primary cells (P1-P6) is not entirely consistent (Figure 2B/C). Given that the two cases are unrelated and the variant is not de novo, it is also important to consider the possibility that p.Y1758C exists in the population at a low frequency and that the interferon phenotype may be caused by other genetic factors. A dominant-negative mechanism appears unlikely, given the recessive mode of inheritance and the absence of symptoms in carrier females. Before concluding pathogenicity, please exclude alternative genetic causes typical of AGS and related interferonopathies.

I recommend performing RNA-seq and immunoblotting to verify that canonical AGS genes (e.g., SAMHD1, TREX1, RNASEH2A/C) are expressed at normal levels, and conducting whole-genome sequencing to rule out pathogenic or likely pathogenic variants, including deep intronic or regulatory variants, in AGS-related genes and other interferonopathy-associated loci.

(2) Genotype–phenotype resolution for ATRX deficiency versus p.Y1758C

The authors propose that p.Y1758C induces cell type-restricted activation of type I interferon via a cGAS-dependent but STING/IRF3-independent mechanism, while ATRX chromatin binding remains intact and only a subset of ATRX-bound genes is misregulated. This model derives from the conventional expectation that a missense ATRX variant with preserved expression and chromatin binding behaves as a partial loss-of-function allele, producing a milder phenotype resembling ATRX deficiency. However, in this case, the two p.Y1758C patients are proposed to exhibit interferon activation that could, in principle, result in a phenotype more severe than that associated with classical hypomorphic ATRX mutations.

I suggest performing in vivo validation, given the systemic involvement of ATRX and AGS. Although technically challenging, generating an isogenic animal model (p.Y1758C knock-in) would be compelling to establish X-linked recessive inheritance, tissue specificity, and neuroinflammatory phenotypes, and to test whether the IFN-I response exceeds that observed with ATRX haploinsufficiency or hypomorphic mutations.

I also propose providing a clearer side-by-side comparison of WT, ATRX knockout (conditional), and p.Y1758C knock-in cells in the same genetic background across multiple cell types, quantifying IFN-I output (associated protein and gene expressions) together with chromatin binding metrics (ATRX-regulated genes, DAXX and H3.3 occupancy) at the same loci. This would help determine whether p.Y1758C represents a separable function of ATRX rather than simply recapitulating loss-of-function.

To further strengthen the mechanistic basis for the conclusion that IFN-I activation is cGAS-dependent yet independent of canonical DNA-sensing in the cells with ATRX p.Y1758C mutation, additional experiments could be considered. These may include investigating conventional DNA damage response pathways implicated in AGS and related interferonopathies, to determine whether specific genotoxic stressors or repair deficiencies contribute to IFN activation in this context.

Overall, this study provides an intriguing observation linking ATRX dysfunction to neuroinflammation via a non-canonical role of cGAS in transcriptional regulation. The work will be of interest to researchers in the field. However, publication in Nature Communications requires stronger evidence on two fronts. First, a more rigorous demonstration that ATRX p.Y1758C is the causal variant in these patients, independent of perturbations in other AGS-related pathways. Second, a clearer genotype-phenotype relationship that distinguishes the effects of p.Y1758C from ATRX loss-of-function mutations, supported by in vivo animal models and deeper mechanistic insight. Pending these additions, I find the current mechanistic and genetic evidence insufficient to support acceptance.

Response to Reviewer #2:

"I am not fully convinced that ATRX p.Y1758C is clearly established as a pathogenic variant" & "consider the possibility that p.Y1758C exists in the population at a low frequency" & "please exclude alternative genetic causes typical of AGS and related interferonopathies"

Response: We appreciate the importance placed by the reviewer on proof of pathogenicity of the Y1758C substitution. We are pleased to report then, that, in addition to the data that we presented in our initial submission, we have now been able to demonstrate that Y1758C ATRX protein has reduced ATPase activity compared to wildtype protein, and translocase activity equivalent to a catalytically dead K1600R ATRX mutant (Fig.1e-f).

Summarising, there are multiple lines of evidence supporting the pathogenicity of Y1758C: ***Genetic***: The variant is **very rare** i.e. it is **not present on more than 1,100,000 alleles** sequenced on gnomAD. In contrast, we identified two unrelated individuals (one from the UK, one from France) with this variant, both of whom manifest a similar neuroinflammatory phenotype associated with enhanced type I interferon signalling in blood. ***Phenotypic***: Patient 2 has **classical facial features** of ATRX syndrome. Further, this patient demonstrates a genomic **DNA methylation signature <u>diagnostic</u> of ATRX syndrome** (see PMIDs: 29304373; 28293299). ***Experimental***: Our functional studies show that the **Y1758C induces enhanced type I interferon signalling ex vivo** (using patient-derived cells) **and in vitro** (using a variety of cell models). Importantly, we have now expressed and purified full-length ATRX protein and used in vitro assays to show that the **Y1758C substitution is hypomorphic for ATPase activity** (as described in Mitson et al. (PMID: 21505078) and Tang et al. (PMID: 14990586) for other mutations of ATRX associated with retained protein expression). Further, we show that this loss of ATPase activity is associated with a **marked reduction of translocase activity**. The results of these experiments are now included in our manuscript (Fig.1e-f).

We have extensive, possibly unmatched, expertise in the study of Aicardi-Goutières syndrome (AGS) - having made the original identification of all currently assigned AGS-related genes (PMIDs:16845398; 16845400; 19525956; 23001123; 24686847; 33230297), pioneered the development of both the 'interferon score' (PMID: 24183309) and the interferon alpha digital ELISA assay (PMID: 28420733), authored the major published genotype-phenotype series relating to AGS (e.g. see PMIDs: 25604658; 28561207; 31898846), and executed the first registered clinical trial in AGS (PMID: 30566312). Regarding other type I interferonopathies, having introduced this concept into the medical lexicon (PMID: 22129056), we have been responsible for the identification of close to one half of the ~ 50 currently recognised such disorders (see Table S2 in PMID: 38968338), and have deep knowledge of the genetic and phenotypic spectrum of these diseases (as examples published in 2024/5 alone, see: PMIDs: 38324161; 38869500; 39196411; 39986310; 39424561; 41049574). As stated in the Methods, both patients described in our original submission underwent **whole exome sequencing**, with no suspicious pathogenic variants identified in AGS1-9 (or any other type I interferonopathy associated gene). Further, the two cases that we describe **do not present as typically seen in AGS, or other type I interferonopathies**. Finally, we are fully cognisant of the need to consider the possibility of cryptic mutations, including intronic (PMID: 36775013) and synonymous variants (PMID: 23592335). Given this background, and – most importantly - the strength of the functional data that we have derived relating to the Y1758C substitution in ATRX, we are confident that we have excluded relevant alternative diagnoses in our patients.

"The authors propose that p.Y1758C acts as a gain-of-function variant" & "the correlation between ATRX genotype and the extent of type I interferon induction among patient-derived primary cells (P1-P6) is not entirely consistent" & "the conventional expectation that a missense ATRX variant with preserved expression and chromatin binding behaves as a partial loss-of-function allele"

Response: It is important to state that **nowhere in our manuscript do we argue that the Y1758C allele acts as a gain-of-function at the molecular level**. Indeed, in line with our previously described ex vivo characterisation of patient fibroblasts, and our in vitro data showing that Y1758C knock-in (KI) and ATRX knock-out (KO) result in similar effects in terms of inducing interferon signalling, our new in vitro activity assay using full-length expressed ATRX protein shows that **Y1758C leads to a loss of ATPase activity as described for other 'classical' ATR-X syndrome associated mutations** (PMID: 21505078; 14990586). Rather, as argued previously, the available evidence strongly suggests that variability in inflammatory signalling reflects the **stochastic nature** of perturbed chromatin accessibility and gene expression. This phenomenon is already well-established in the context of ATRX; best illustrated by the finding that only a proportion of patients with germline mutations in *ATRX* manifest indices of alpha thalassemia (PMID: 18409179). Following up on this observation, in demonstrating a function for ATRX on both heterochromatin **and euchromatin**, Richard Gibbons (a co-author on our paper) (PMID: 35710802) went on to show that the role of ATRX at the well-characterized α-globin locus in erythroid cells leads to **stochastic changes in gene expression** and cell fate **in only a subset of cells** (mediated through

interdependent effects on DNA replication, chromatin modification and chromatin accessibility). Summarising, **we propose neither qualitative nor quantitative differences between the Y1758C substitution and other ATR-X syndrome associated mutations per se**.

With the advent of unbiased exome sequencing, **variability in disease expression** among patients with mutations in ATRX is now better appreciated. Thus, in a recent review of more than 200 individuals with ATR-X syndrome, intellectual disability was the only clinical feature shared by all patients (PMID: 34524523). Further, recognition of the **breadth of phenotypic spectrum** related to germline mutations in ATRX is also increasing: for example, we note the description of **osteosarcoma** in six patients with ATR-X syndrome (PMIDs: 28371217; 28371197; 29706636; 32191290), a previously unappreciated association. As mentioned in our original submission, cerebral white matter disease reminiscent of type I interferon driven neuroinflammation has been reported by other authors in a subset of patients with ATR-X syndrome (PMIDs: 23681356; 25936994). In line with our contention that inflammation is likely an under-recognised feature of ATR-X syndrome, we now include description of a **new patient with classical ATR-X syndrome** (severe developmental delay; characteristic facial features; **diagnostic** methylation pattern) who presented in the first year of life with **persistent fever, hepatitis and lymphocytic panniculitis** associated with **enhanced type I interferon signalling** in blood and **highly elevated neopterin in the cerebrospinal fluid indicating neuroinflammation**. Notably, these features have responded to 'anti-interferon' therapy using the JAK1/2 inhibitor ruxolitinib. The maternally inherited c.6409G>A / p.Ala2137Thr mutation seen in this new patient has been recorded twice on DECIPHER (Patient 281787: https://www.deciphergenomics.org/patient/281787/genotype/205426/browser; Patient 531448: https://www.deciphergenomics.org/patient/531448/genotype/309651/browser) as a cause of classical ATR-X syndrome in the absence of reported features of inflammation. These observations strongly support our contention that **the link between mutations in *ATRX* and engagement of innate immune signalling is not mutation-specific, but reflective of a stochastic phenomenon linked to ATRX dysfunction**. A description of this new patient is now included in our manuscript.

"loss of ATRX suppresses rather than enhances type-I interferon responses"

Response: As the reviewer states, the three papers cited (PMIDs: 37693131; 35939517; 37200088) indicate that ATRX acts, in some cases, to 'facilitate' type I interferon signalling (with loss of ATRX 'suppressing' type I interferon responses). Indeed we referenced PMID: 37200088 in our original submission (ref. 44), stating that: *"… the immunogenicity of ATRX deficiency is apparently cell type specific; thus, while ATRX null sarcomas demonstrate impaired adaptive and innate immune signalling after ionizing radiation[44], ATRX mutations mediate an immunogenic phenotype with macrophage infiltration in neuroblastoma[45]*." Notably, PMID: 39892705 (our original ref. 45) shows that KO of ATRX in neuroblastoma lines results in significant **upregulation** of interferon signalling (see their Figure 2A, B). Further, as also noted in our original submission, gene set enrichment pathway analysis of publicly available datasets in ***ATRX*-null induced pluripotent stem cells**, ***Atrx*-null mouse forebrain** and ***Atrx*-null mouse neuronal progenitor cells** (PMIDs: 30392976; 18842153; 29535300 respectively), identifies an **upregulation of interferon responses** in the context of *ATRX* loss (Fig. S2a). More recently, the group of Nathalie Berubé, an expert in ATRX biology (https://pubmed.ncbi.nlm.nih.gov/?term=berube+atrx&sort=pubdate&size=200) reported an **upregulation of type I interferon signalling in *Atrx*-null microglia** (PMID: 40938932: we draw particular attention to their Figure 4F, L and M). These examples, together with the data that we present, clearly illustrate a complex situation where loss of ATRX can lead to variable effects on type I interferon signalling. We hold that our results are of particular importance because they are **derived in humans** at the l**evel of the whole organism**, once again illustrating the potential of Mendelian disease to deconvolute complex biology in the human context. We now include reference to PMID: 40938932 in our revision.

"generating an isogenic animal model (p.Y1758C knock-in) would be compelling"

Response: The only ATR-X syndrome-associated substitution modelled in a mouse yet described was generated by Rebekah Tillotson, a co-author on our paper (PMID: 37171606). While we agree that a mouse model harbouring the Y1758C mutation might be informative from a mechanistic perspective (also allowing for comparison with the R246C mouse built by Rebekah), we do not believe that a mouse model is necessary to confirm the pathogenicity of the Y1758C substitution. In this regard, we are grateful to Dr Powell for excluding the generation of an animal model as a prerequisite for resubmission. As a point of note, it is striking that none of the AGS-associated mouse models described to date reliably recapitulate the neuroinflammatory phenotype seen in humans (e.g. while the *Trex1* KO mouse manifests systemic inflammation, the brain is not involved; meanwhile, the *Samhd1* KO mouse demonstrates no overt clinical phenotype at all, and the same is true of the KI models of Rnase-H2 patient-associated mutations). These

findings suggest important differences between mouse and human in terms of engagement of type I interferon signalling.

"a clearer side-by-side comparison of WT, ATRX knockout (conditional), and p.Y1758C knock-in cells in the same genetic background across multiple cell types"

Response: In our original submission we provided multiple side-by-side comparisons of WT, Y1758C and KO ATRX BJ-fibroblasts. We also provided a side-by-side comparison of WT and Y1758C induced pluripotent stem cells (iPSCs) and neurons, with ATRX-null iPSCs non-viable in our hands. We submit a composite figure ("KI-KO-SideBySide-part1" and "2") for review purposes only comprised of all these results, while continuing to present these comparisons according to the analyses being described in the text. These data strongly support our contention that the consequences of the Y1758C mutation are broadly similar those of ATRX KO.

"*investigating conventional DNA damage response pathways.*"

Response: We thank the author for this suggestion. It is well known that genomic stability is compromised by the loss of ATRX (e.g. PMIDs: 18227278; 23563309; 24651726), and we are aware of the link between DNA damage and interferon signalling through our own work and that of others. Indeed, at the start of this project, we hypothesised that DNA derived from DNA damage was the most likely explanation for the upregulation of type I interferon signalling that we observed in patients. As expected then, we find ATRX patient fibroblasts to exhibit an increased number of double-stranded DNA breaks (data which we now include in a new supplementary (Fig. S9). However, **the absence of elevated cGAMP** in patient cells, and our demonstration of a dependency of interferon signalling on cGAS **but independent of its enzymatic function**, indicates that genome instability does not contribute majorly to the enhanced interferon signalling observed in the ATRX deficient cells that we have studied.

Reviewer #3 (Remarks to the Author):

In this manuscript, El-Daher et al. suggest a novel mechanistic link between ATRX deficiency and type I interferon activation mediated via a non-canonical cGAS-dependent pathway. The authors identify a recurrent ATRX Y1758C mutation in two unrelated patients with neuroinflammatory phenotypes, then demonstrate that ATRX deficiency contributes to type I interferon-mediated inflammation in a cGAS-dependent manner, accompanied by chromatin remodeling characterized by occupancy of the histone chaperone DAXX and the histone variant H3.3. The experimental design and results are generally robust, and the manuscript is clearly structured. This manuscript presents an intriguing and original concept that ATRX deficiency enhances type I IFN signaling through a non-canonical cGAS-dependent pathway. However, the proposed mechanism currently lacks sufficient evidence, and the clinical relevance remains limited. A more refined elucidation of the noncanonical functions of cGAS, along with additional functional validation, would substantially strengthen the completeness and impact of the study.

Major Points

1) In the Discussion section, the authors have appropriately addressed the significance and limitations of their study as follows: 1) How this mutation affects ATRX activity requires further study…….We speculate that the Y1758C substitution might also affect the helicase activity of ATRX, and thereby its ability to remodel chromatin function 2) Elucidating the pathways involved in the altered dynamics of DAXX and H3.3 at chromatin, and determining if these changes work alongside the recruitment of STATs/IRFs to boost the expression of inflammatory genes, will be necessary. 3)……should explore how cGAS contributes to changes in chromatin remodelling consequent upon ATRX deficiency. However, these key questions are essential to explaining the conclusions of this study. To substantiate the work's novelty, at least part of these discussion points should be experimentally validated.

2) Partial effect of IFNAR blockade requires deeper interpretation. Anifrolumab treatment reduces only 35 of 183 ISGs (Fig. 3B), suggesting the effect of IFNAR blockade is very partial. This may weaken confidence in the contribution of IFNAR downstream signaling to ATRX-mediated inflammation. In addition, one consistent finding from this study is that pSTAT1 is increased. Thus, the authors need to address 1) the protein level of IFNs, 2) the binding sites of STAT1 (ChIP-qPCR or ChIP-seq).

3) In relation to the above comments, the rigor of the experiment on the data in Fig. 3F needs to be checked, and the results need to be reinterpreted. 1) There are definitely signals for all proteins in the 7th lane, which should be blank. 2) The last lane in Fig. 3A is agonist-treated ATRX WT cells, but it is unclear what the last

lane in Fig. 3F is. In fig. 3F, the amount of ATRX is significantly reduced, and while pSTAT1 increases, the amount of STAT1 remains unchanged. This requires further elucidation.

4) Still, the clinical relevance remains limited. In samples from patients with p3 and p4, expression of ATRX was significantly reduced, but ISG expression was not increased, so the main mechanism proposed by the authors is unlikely to apply to all patients. Thus, the conclusion that this finding defines a “novel interferonopathy” appears overly definitive and should be presented with greater caution. Otherwise, the key findings should be reconfirmed using iPSC-derived neurons, as the authors note: "Moreover, the immunogenicity of ATRX deficiency is apparently cell type specific."

5) It would be important to quantitatively assess whether the observed changes in DAXX and H3.3 are restricted to specific ISGs or represent a more global phenomenon across the transcriptome.

Minor Points

1) The authors utilized a curated list of 444 ISGs. Please provide the source, curation criteria in the Methods.

2) It should be clearly demonstrated whether the expression levels of WT and D319A cGAS in Fig. 4E–F are comparable, as differences in expression could significantly affect the interpretation of the results. In addition, the use of cGAS-D319A mutant supports enzymatic independence, but residual or compensatory effects cannot be fully excluded. Quantification of nuclear localization, stability, and expression levels between WT and D319A constructs should be provided to exclude artifacts.

Response to Reviewer #3:

1) In the Discussion section, the authors have appropriately addressed the significance and limitations of their study as follows: 1) How this mutation affects ATRX activity requires further study. We speculate that the Y1758C substitution might also affect the helicase activity of ATRX, and thereby its ability to remodel chromatin function 2) Elucidating the pathways involved in the altered dynamics of DAXX and H3.3 at chromatin, and determining if these changes work alongside the recruitment of STATs/IRFs to boost the expression of inflammatory genes, will be necessary. 3) .... should explore how cGAS contributes to changes in chromatin remodelling consequent upon ATRX deficiency. However, these key questions are essential to explaining the conclusions of this study. To substantiate the work novelty, ***at least part of these discussion points should be experimentally validated****.*

Response: We thank the reviewer for this summary. As noted above, we are very pleased to report that we have now been able to express wild-type (WT), Y1758C and helicase dead (K1600R) ATRX mutant full-length protein. The results of this technically challenging experiment (to our knowledge patient-associated ATRX mutants have only ever been analysed in this way twice before (PMIDs: 21505078; 14990586)) show that the Y1758 substitution is hypomorphic for ATPase activity. We further show that the mutant exhibits markedly reduced translocase activity (Fig.1e-f). These new data, together with our ascertainment of a further patient with ATR-X syndrome and inflammation associated with indices of enhanced type I interferon signalling, and the recent publication from Natalie Berubé’s group showing upregulation of type I interferon signalling in microglia with targeted loss of *Atrx* substantiate our findings that mutations in ATRX can result in engagement of the innate immune system.

Partial effect of IFNAR blockade requires deeper interpretation*. Anifrolumab treatment reduces only 35 of 183 ISGs (Fig. 3B), suggesting the effect of IFNAR blockade is very partial. This may weaken confidence in the contribution of IFNAR downstream signaling to ATRX-mediated inflammation. In addition, one consistent finding from this study is that pSTAT1 is increased. Thus,* ***the authors need to address 1) the protein level of IFNs, 2) the binding sites of STAT1 (ChIP-qPCR or ChIP-seq)****.*

Response: We completely agree with the reviewer that the contribution of IFNAR-mediated downstream signalling to inflammation related to ATRX dysfunction is partial, and that additional **cell-intrinsic processes are also relevant** - with our data suggesting that the remodelling activity of ATRX is essential to maintain the homeostatic expression of ISGs by regulating the chromatin landscape at genes. We now state this interpretation explicitly in the text (and apologise for not having done so in our original submission). As suggested by the reviewer, we also now assess type 1 interferon protein levels through the use of an interferon activity assay. The HEK-Blue ISG system involves cells expressing a secreted embryonic alkaline phosphatase (SEAP) reporter under the control of an interferon regulatory factor (IRF)-inducible promoter, specifically containing ISRE (IFN-stimulated response elements) fused to an ISG54 minimal promoter, with SEAP secretion assessed by colorimetric assay and absorbance at 405 nm. In

agreement with our previously provided qPCR data showing increased expression of *IFNB1* in ATRX-deficient BJ fibroblasts (Fig. 2e), and consistent with the higher levels of phosphorylated STAT1 in these cells (Fig. 3a), the SEAP reporter assay confirms elevated levels of interferon protein activity in cell supernatants. These data are now included in a new supplementary figure (Fig. S4a).

Relating to ChIP-qPCR and ChIP-seq, we are not able to perform such ChIP experiments because of the large number of cells required (given that our experiments have been undertaken in fibroblasts). An alternative might be to use Cut&Tag. However, such an experiment, involving at least two clones each of WT, ATRX-KI and ATRX-KO cells, represents a major undertaking taking several months, with no guarantee of success (given that antibody optimisation is often difficult in this system). While we appreciate that assessing the presence of STAT1 at gene promoters might provide further insights into the ATRX-related phenomenon that we report (and is surely therefore worthy of further study), we politely argue that such data are not integral to the important message that our results already convey.

*3) In relation to the above comments, **the rigor of the experiment on the data in Fig. 3F needs to be checked, and the results need to be reinterpreted**. 1) There are definitely signals for all proteins in the 7th lane, which should be blank. 2) The last lane in Fig. 3A is agonist-treated ATRX WT cells, but it is unclear what the last lane in Fig. 3F is. In fig. 3F, the amount of ATRX is significantly reduced, and while pSTAT1 increases, the amount of STAT1 remains unchanged. This requires further elucidation.*

Response: We thank the reviewer for this comment and sincerely apologise for the quality of the immunoblot provided in our original submission (with the feint bands in the 'blank' lane we believe likely due to inadvertent 'spill-over'). We now include a different representative blot (still numbered as Fig. 3f). To be clear, the last lane in Figures 3a and 3f both relate to treatment with the STING agonist diABZI, serving as a positive control. This is now stated explicitly in the figure legend (and we apologise for not doing so in the first instance).

*4) Still, the clinical relevance remains limited. In samples from patients with p3 and p4, expression of ATRX was significantly reduced, but ISG expression was not increased, so **the main mechanism proposed by the authors is unlikely to apply to all patients**. Thus, **the conclusion that this finding defines a novel interferonopathy appears overly definitive and should be presented with greater caution**. Otherwise, the **key findings should be reconfirmed using iPSC-derived neurons**, as the authors note: "Moreover, the immunogenicity of ATRX deficiency is apparently cell type specific."*

Response: We agree with the reviewer that enhanced type I interferon signalling may not be relevant in all patients with ATR-X syndrome. Analogous to our demonstration that mutations in ATAD3A can result in both a loss of mitochondrial function per se, and an induction of innate immune signalling (PMID: 34387651), we suggest that disturbance of ATRX function has more than one consequence, one of which can relate to engagement of the type I interferon signalling machinery in some cases. As suggested by the reviewer, we have revised the text accordingly. Relating to iPSCs and iPSC-derived neurons, while we have not undertaken detailed chromatin studies equivalent of those we explored in BJ fibroblasts, we do show that mutation of ATRX leads to the emergence of an interferon response in Y1758C KI neurons (Fig. 2j). Noting that these data are consistent with our gene set enrichment pathway analysis of publicly available datasets in *ATRX*-null induced pluripotent stem cells, *Atrx*-null mouse forebrain and *Atrx*-null mouse neuronal progenitor cells (PMIDs: 30392976; 18842153; 29535300 respectively) (Fig. S2a), and the recently published data from the Berubé group reporting an upregulation of type I interferon signalling in *Atrx*-null microglia (PMID: 40938932: drawing particular attention to their Figure 4F, L and M).

5) It would be important to quantitatively assess whether the observed changes in DAXX and H3.3 are restricted to specific ISGs or represent a more global phenomenon across the transcriptome.

Response: We thank the reviewer for raising this interesting point. To quantitively assess the genome-wide re-distribution of DAXX in ATRX deficient cells, we have now re-analysed our Cut&Tag data – using differential binding analysis (PMID: 22217937) (Fig. S8a-b) followed by an assessment of functional enrichment using STRING (https://string-db.org/). Notably, within the group of genes showing reduced DAXX binding and increased expression, we observe a statistically significant enrichment for genes involved in interferon/immune signalling (Fig. 6d-g). In contrast, while EMT-related genes – like ISGs - show increased expression in ATRX deficient cells, there is no significant change in DAXX occupancy (Fig. S8c). As such, these data suggest a specificity relating to the homeostatic regulation of ISG expression by DAXX. Regarding H3.3, our previous analysis showed that its occupancy at genes positively correlates with gene expression (Fig. 7a), and we have now further validated this result by performing plot profile analysis at

TSS of up- or down-regulated genes in ATRX deficiency (Fig. S8d-e). Thus, in contrast to DAXX, change in H3.3 occupancy appears to reflect a more global phenomenon of transcription.

Minor points:

The authors utilized a curated list of 444 ISGs. Please provide the source, curation criteria in the Methods.

Response: We sincerely apologise for this omission in our original submission. The complete list of 444 validated ISGs is now included in a new Supplementary table 1. This list was derived from http://www.pdg.cnb.uam.es/cursos/BioInfo2002/pages/extras/software/DatabasesListNAR2002/summary/16.html), which includes the ~380 ISGs assessed by Schoggins et al. (PMID: 21478870), those that we see consistently upregulated in datasets derived from patients with Aicardi-Goutières syndrome and other type I interferonopathies (e.g. PMID: 31836668), and the 'interferon programme' annotated in GSEA (https://www.gsea-msigdb.org/gsea/msigdb/collection_details.jsp).

It should be clearly demonstrated whether the expression levels of WT and D319A cGAS in Fig. 4E-F are comparable, as differences in expression could significantly affect the interpretation of the results. In addition, the use of cGAS-D319A mutant supports enzymatic independence, but residual or compensatory effects cannot be fully excluded. Quantification of nuclear localization, stability, and expression levels between WT and D319A constructs should be provided to exclude artifacts.

Response: If we understand this point correctly, we already included a western blot data showing similar expression/stability of WT and D319A cGAS in BJ cGAS null cells. Further, the D319A mutant protein shows no activity whatsoever (Fig. 4e). In addition to this, we now include expression data showing equivalent RNA levels of WT and mutant D319A cGAS (Fig. S6d).

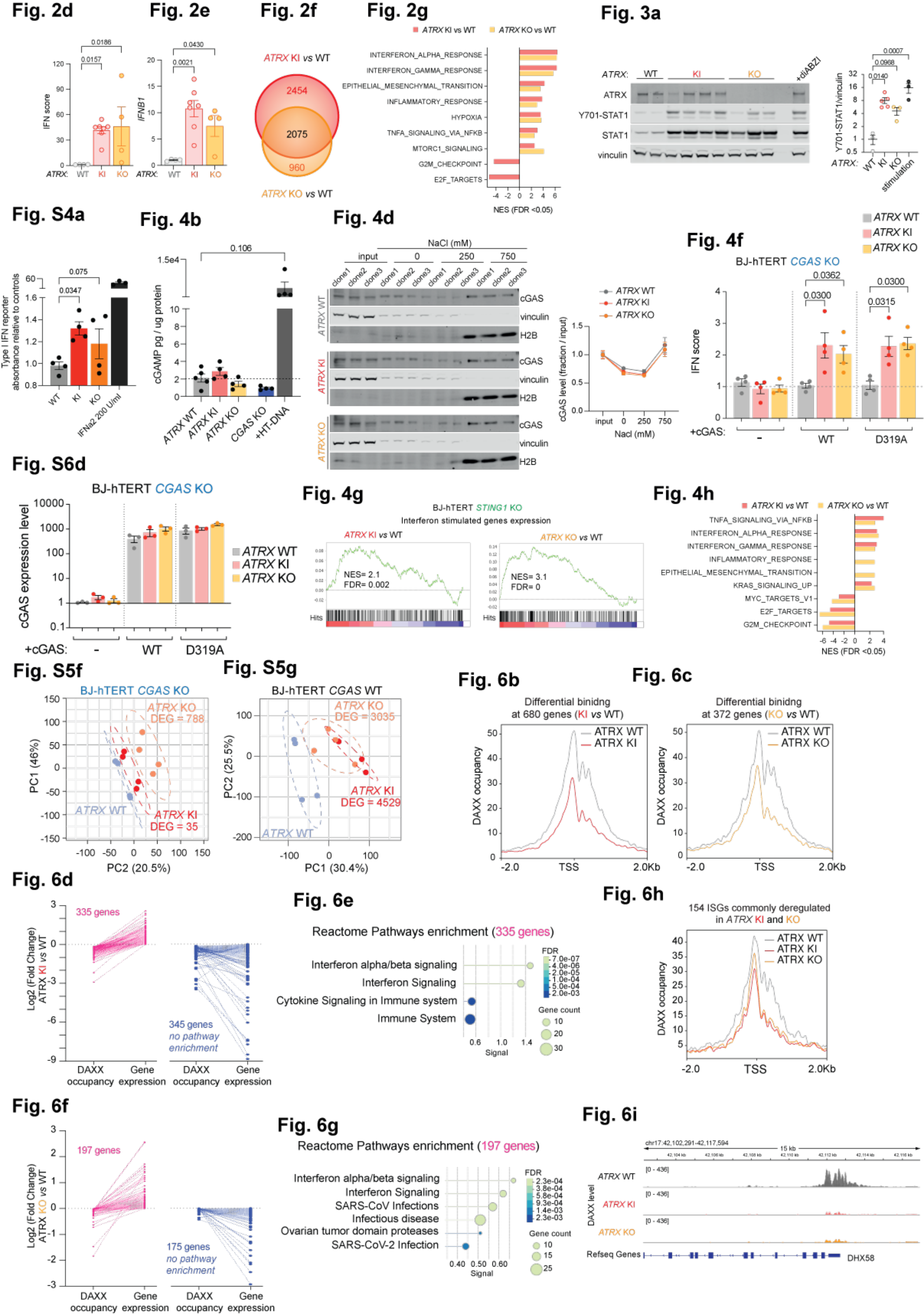

Fig. 2d
IFN score
ATRX: WT KI KO
0.0157
0.0186
Fig. 2e
IFNB1
0.0021
0.0430
Fig. 2f
ATRX KI vs WT
2454
2075
960
ATRX KO vs WT
Fig. 2g
ATRX KI vs WT
ATRX KO vs WT
INTERFERON_ALPHA_RESPONSE
INTERFERON_GAMMA_RESPONSE
EPITHELIAL_MESENCHYMAL_TRANSITION
INFLAMMATORY_RESPONSE
HYPOXIA
TNFA_SIGNALING_VIA_NFKB
MTORC1_SIGNALING
G2M_CHECKPOINT
E2F_TARGETS
NES (FDR <0.05)
Fig. 3a
ATRX: WT KI KO +diABZI
ATRX
Y701-STAT1
STAT1
vinculin
Y701-STAT1/vinculin
0.0140
0.0968
0.0007
stimulation
Fig. S4a
Type I IFN reporter absorbance relative to controls
0.0347
0.075
WT KI KO IFNα2 200 U/ml
Fig. 4b
cGAMP pg / ug protein
0.106
ATRX WT ATRX KI ATRX KO CGAS KO +HT-DNA
Fig. 4d
NaCl (mM)
input 0 250 750
clone1 clone2 clone3
ATRX WT
ATRX KI
ATRX KO
cGAS
vinculin
H2B
cGAS level (fraction / input)
Nacl (mM)
Fig. 4f
ATRX WT
ATRX KI
ATRX KO
BJ-hTERT CGAS KO
IFN score
0.0362
0.0300
0.0300
0.0315
+cGAS: - WT D319A
Fig. S6d
BJ-hTERT CGAS KO
cGAS expression level
ATRX WT
ATRX KI
ATRX KO
+cGAS: - WT D319A
Fig. 4g
BJ-hTERT STING1 KO
Interferon stimulated genes expression
ATRX KI vs WT
ATRX KO vs WT
NES= 2.1
FDR= 0.002
NES= 3.1
FDR= 0
Hits
Fig. 4h
ATRX KI vs WT
ATRX KO vs WT
TNFA_SIGNALING_VIA_NFKB
INTERFERON_ALPHA_RESPONSE
INTERFERON_GAMMA_RESPONSE
INFLAMMATORY_RESPONSE
EPITHELIAL_MESENCHYMAL_TRANSITION
KRAS_SIGNALING_UP
MYC_TARGETS_V1
E2F_TARGETS
G2M_CHECKPOINT
NES (FDR <0.05)
Fig. S5f
BJ-hTERT CGAS KO
ATRX KO
DEG = 788
ATRX WT
ATRX KI
DEG = 35
PC1 (46%)
PC2 (20.5%)
Fig. S5g
BJ-hTERT CGAS WT
ATRX KO
DEG = 3035
ATRX KI
DEG = 4529
ATRX WT
PC2 (25.5%)
PC1 (30.4%)
Fig. 6b
Differential binidng
at 680 genes (KI vs WT)
ATRX WT
ATRX KI
DAXX occupancy
-2.0 TSS 2.0Kb
Fig. 6c
Differential binidng
at 372 genes (KO vs WT)
ATRX WT
ATRX KO
DAXX occupancy
-2.0 TSS 2.0Kb
Fig. 6d
335 genes
Log2 (Fold Change) ATRX KI vs WT
345 genes
no pathway enrichment
DAXX occupancy
Gene expression
Fig. 6e
Reactome Pathways enrichment (335 genes)
Interferon alpha/beta signaling
Interferon Signaling
Cytokine Signaling in Immune system
Immune System
FDR
Gene count
Signal
Fig. 6h
154 ISGs commonly deregulated
in ATRX KI and KO
ATRX WT
ATRX KI
ATRX KO
DAXX occupancy
-2.0 TSS 2.0Kb
Fig. 6f
197 genes
Log2 (Fold Change) ATRX KO vs WT
175 genes
no pathway enrichment
DAXX occupancy
Gene expression
Fig. 6g
Reactome Pathways enrichment (197 genes)
Interferon alpha/beta signaling
Interferon Signaling
SARS-CoV Infections
Infectious disease
Ovarian tumor domain proteases
SARS-CoV-2 Infection
FDR
Gene count
Signal
Fig. 6i
chr17:42,102,291-42,117,594
15 kb
DAXX level
ATRX WT
ATRX KI
ATRX KO
[0 - 436]
Refseq Genes
DHX58

Fig. 7b

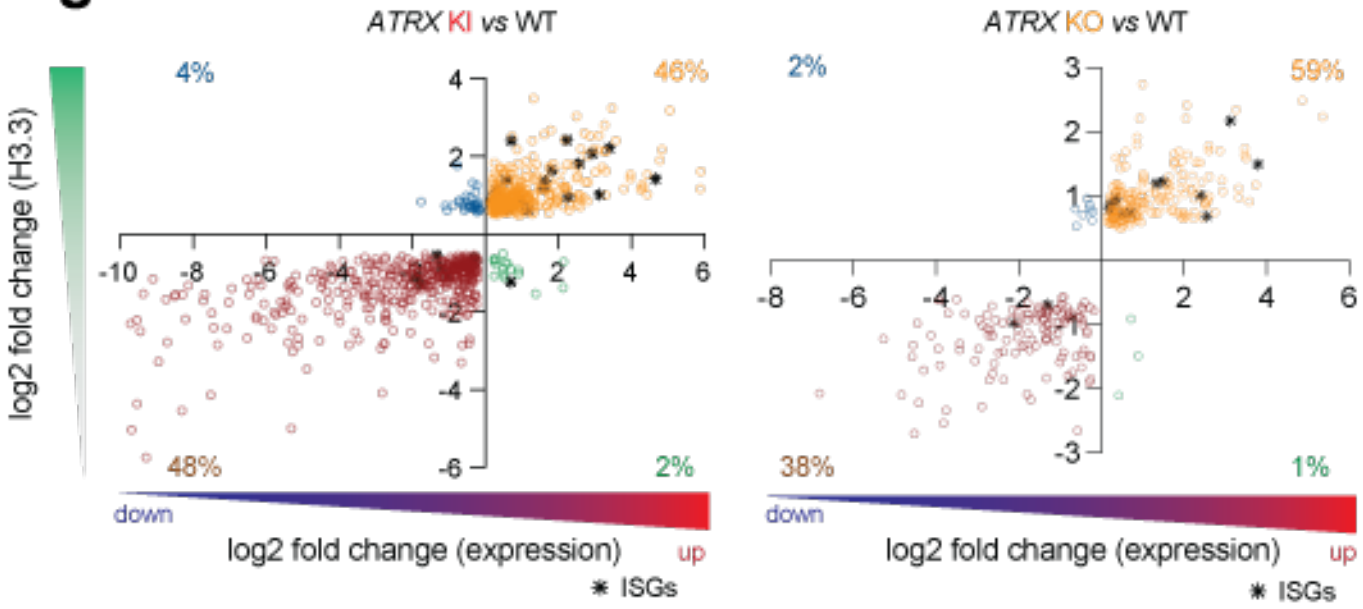

Fig. 7b

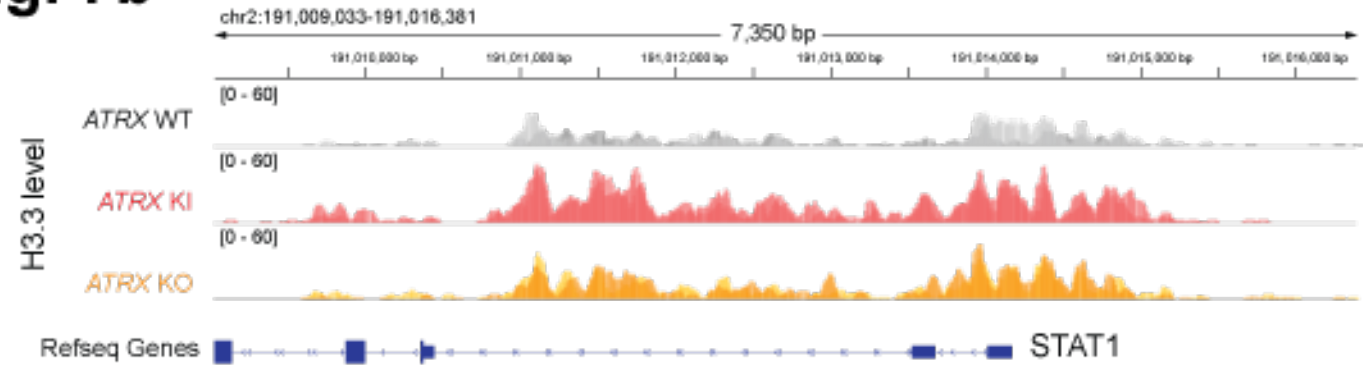

Fig. 7d

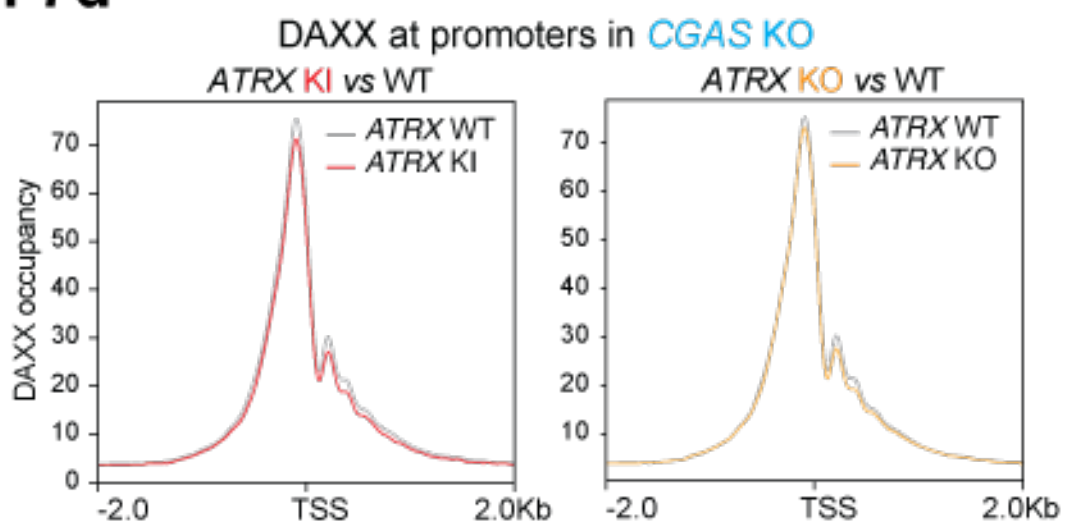

Fig. S8a

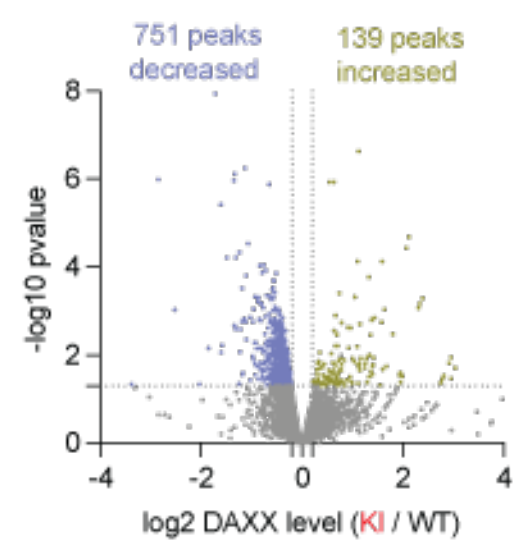

Fig. S8b

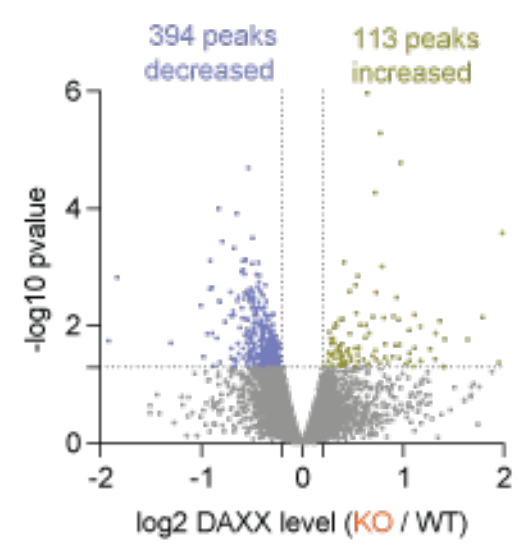

Fig. S8c

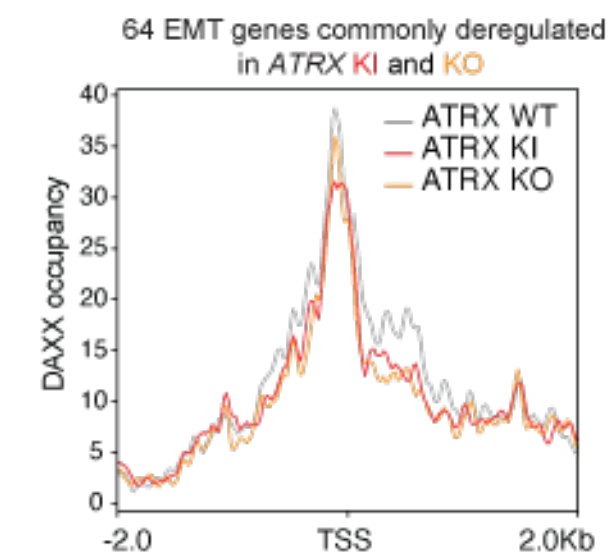

Fig. S8d

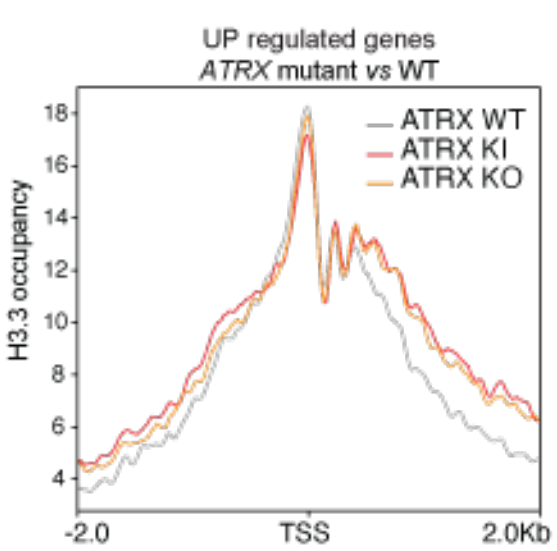

Fig. S8e

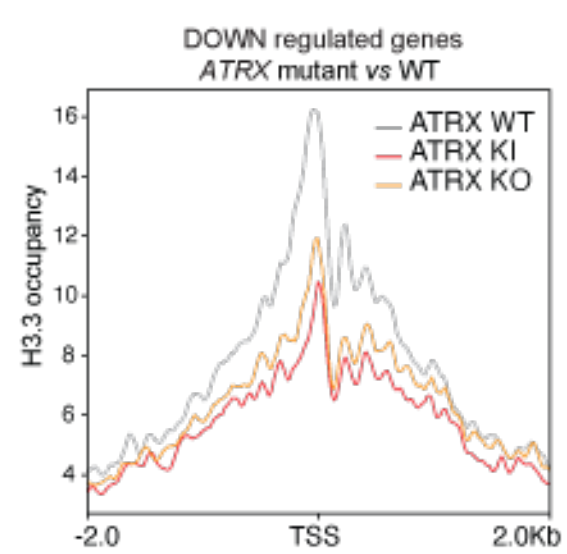

Fig. S8f

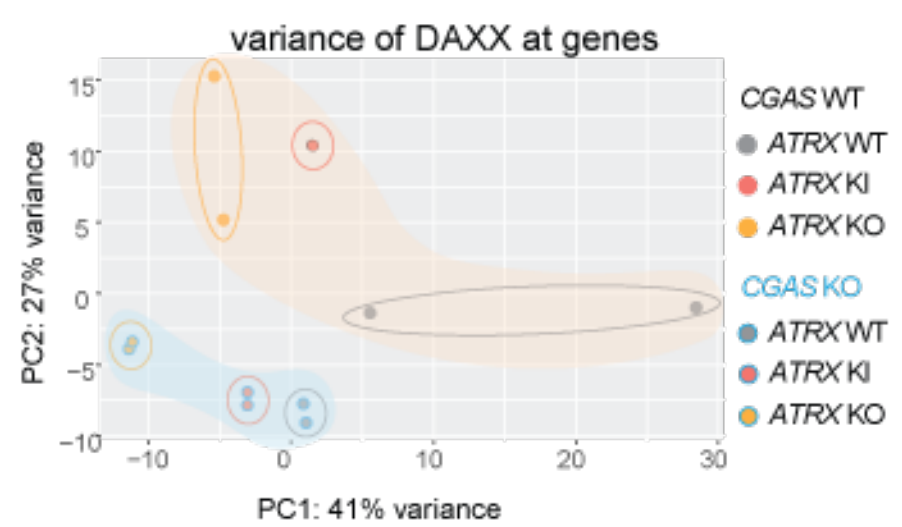

Fig. S8g

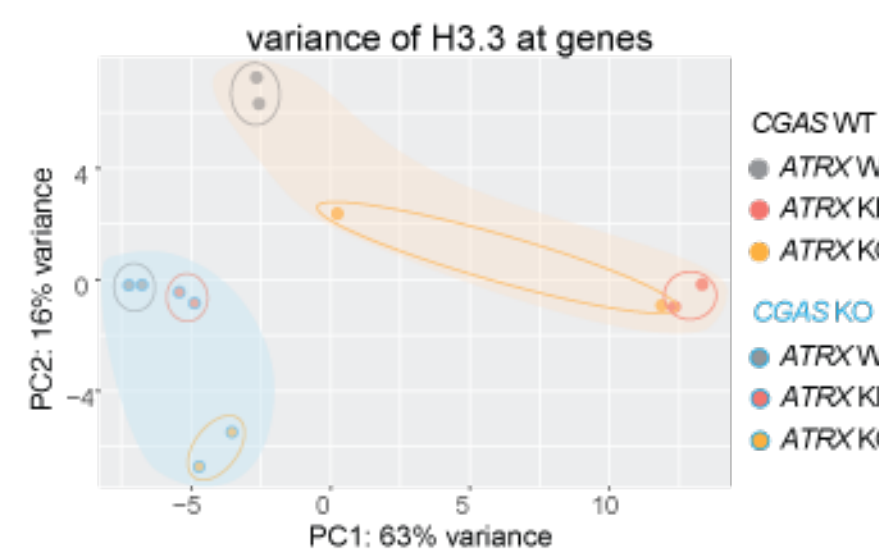

Fig. 2h

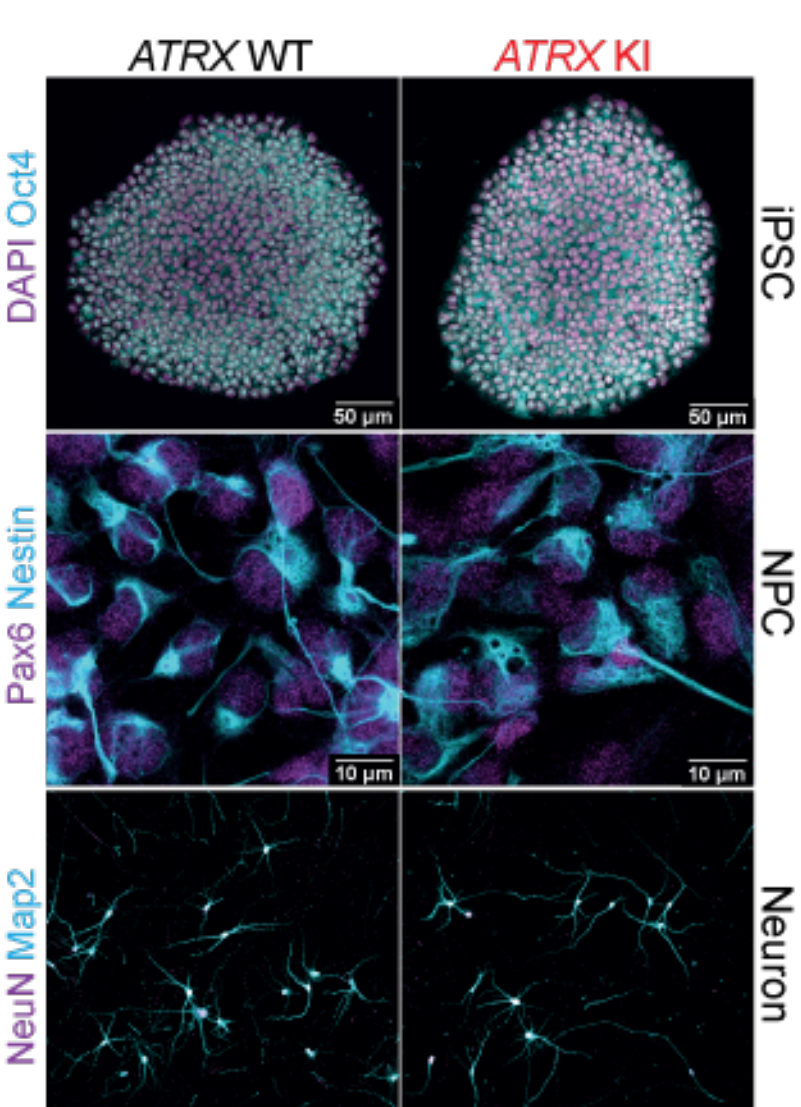

Fig. 2j

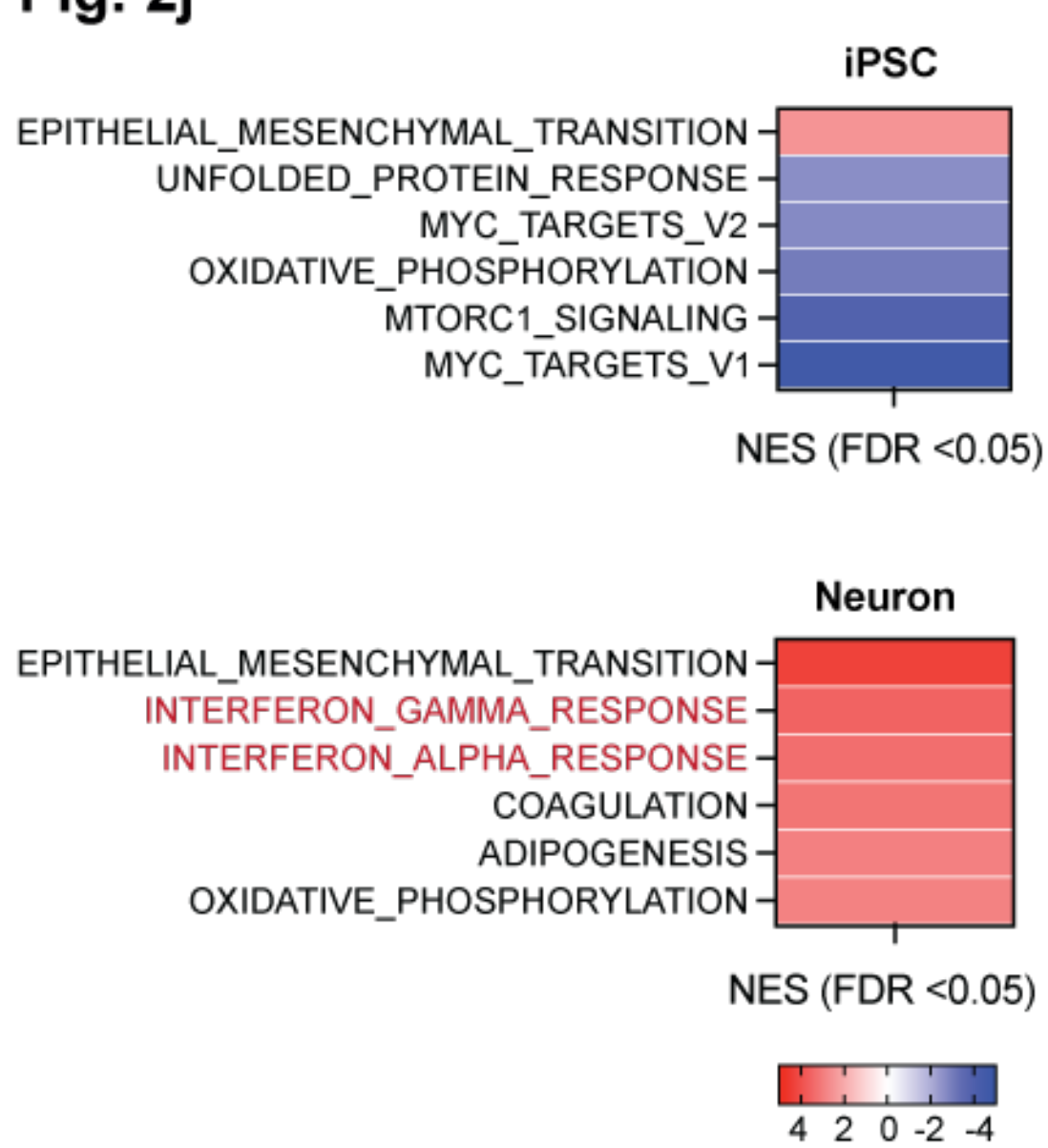

Fig. S3f

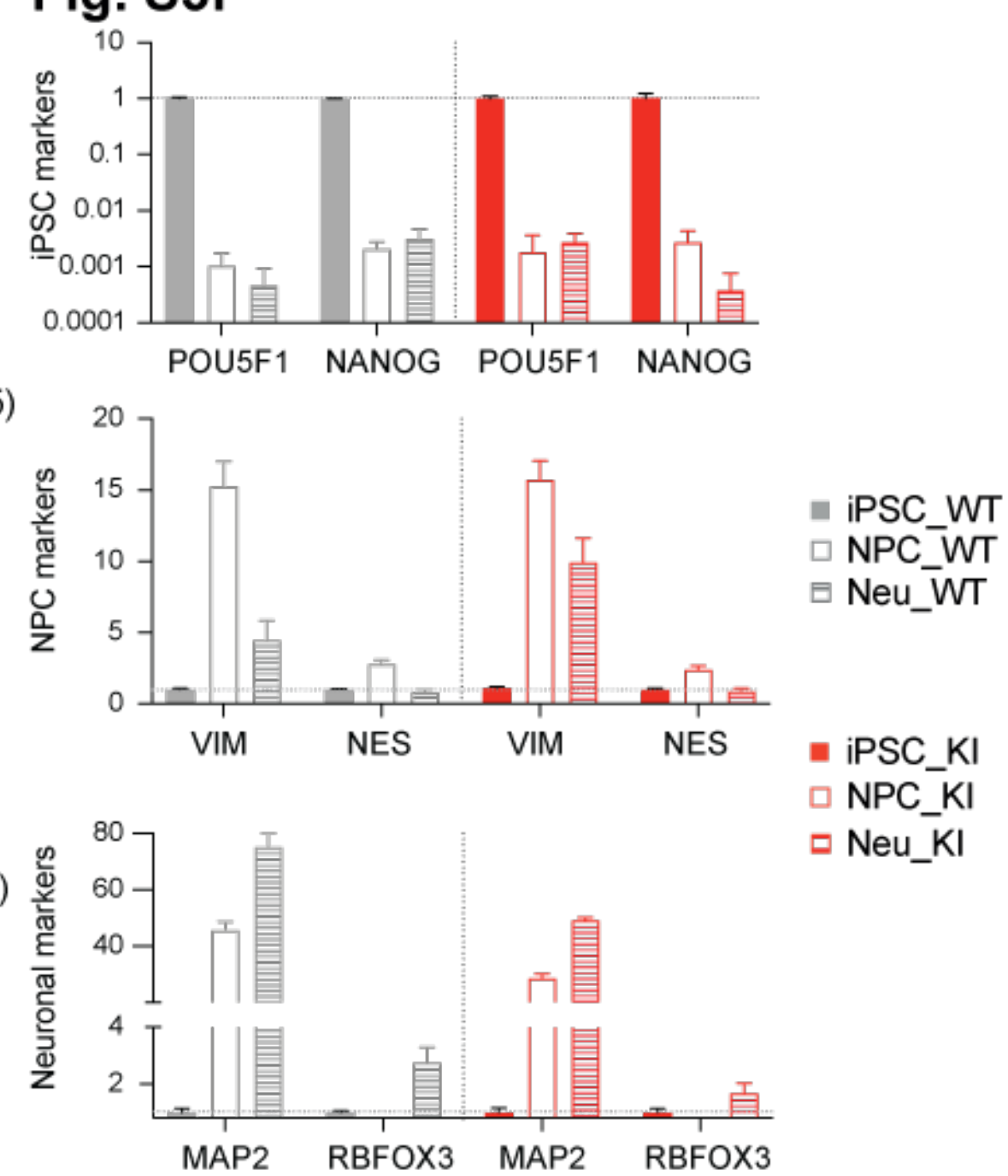

Reviewer #1 (Remarks to the Author):

This revised manuscript from El-Daher et al., along with additional data and detailed responses to reviewer comments, satisfactorily supports the impact of the ATRX mutation on transcriptional regulation, with data supporting disruption of ATPase and translocase activities and reduced DAXX binding that particularly impacts type I interferon-regulated genes - in contrast to EMT-regulated genes. The authors have also incorporated a third patient with a distinct mutation in ATRX in the manuscript.

The authors have provided an extensive response to reviewer #2, making the case that the clinical presentation of the featured patients is not attributable to mutations in genes associated with Aicardi-Goutieres syndrome.

They have also responded to each of the comments of reviewer 3, with acknowledgement that the effects of the ATRX mutation are only partial, with apparent contributions of cell intrinsic factors to degree of activation of the interferon-regulated genes as well as the stochastic nature of these and other ATRX mutations, resulting in variable clinical phenotypes. As requested by the reviewer, the authors have documented the ATPase and translocase functions that are impaired by the mutation, and they have provided a cleaner immunoblot for figure 3.f. All together, the authors have provided a detailed and extensive analysis of the implications of the ATRX mutation for gene regulation, the dependence of its effects on cGAS but not on signaling downstream from cGAS, and its actions related to chromatin remodeling. While there remain opportunities for further characterization of the interactions of cGAS, ATRX and DAXX with regard to epigenetic remodeling and gene transcription, the impact of the mutation on the IFN pathway, even if manifest to different degrees in distinct individual patients, provides new insights into the interactions required for transcriptional regulation of type I interferon, with implications for innate immune activation contributing to pathology, particularly in the CNS**.**

Response to reviewer #1: We sincerely thank the reviewer for their favourable opinion, and also for their assessment of our responses to the issues raised by Reviewer #2 and Reviewer #3.

Reviewer #2 (Remarks to the Author):

After carefully reviewing the authors' rebuttal, I remain unconvinced that the novelty of the ATRX-Y1758C mutation -namely that it causes disease through a mechanism distinct from canonical ΔATRX-null type deficiency- has been adequately demonstrated.

To justify publication in Nature Communications, the authors need to show at the molecular level that the disease mechanism associated with ATRX-Y1758C is functionally distinguishable from ATRX-KO mutations (null variants such as stopgain or frameshift mutations abolishing protein expression) as well as from previously reported ATRX missense mutations.

A key issue is whether the disease mechanism in the alpha-thalassaemia patients P1/P2 (ATRX-Y1758C) is fundamentally distinct from that in the ATRX syndrome patients P4/P7 used as controls, and from previously reported ATRX cases (and models). As also noted by the authors, the impact of ATRX abnormalities on IFN responses varies widely depending on cell type and context. Consistent with this, prior studies report both suppression and activation of IFN signalling upon ATRX loss, indicating that the field remains controversial (Floyd, J Clin Invest. 2023; Wang, Am J Cancer Res 2023; Whitford, Nature Communications 2025).

This variability is clearly illustrated by the comparison between P4 and P7 in Fig1. Fig.1a and Fig.1b show no clear correlation between ATRX protein levels (Fig. S3) and IFN score or IFNB1 expression. Although both P4 (W222C, associated with protein instability) and P7 (a frameshift null mutation) lack detectable ATRX protein (ΔATRX), their IFN responses are opposite (P4 suppressed, P7 enhanced). Thus, IFN transcriptional responses are not uniquely determined by ATRX protein status, and IFN readouts should be interpreted with caution as mechanistic endpoints. While these data support context-dependent regulation of IFN signalling downstream of ATRX dysfunction, they do not by themselves establish an ATRX-Y1758C-specific disease mechanism. If IFN responses cannot be clearly distinguished between ΔATRX-null mutations and ATRX-Y1758C, support for novelty specific to ATRX-Y1758C remains limited.

The authors classify ATRX-Y1758C as a loss-of-function mutation. My earlier use of the term 'gain-of-function' reflected wording rather than intent. The key point is that ATRX-Y1758C retains protein stability and chromatin binding comparable to wild-type ATRX, yet activates IFN signalling in a cGAS-dependent, canonical-pathway-independent manner. While the authors interpret this as a hypomorphic loss-of-function, it remains unclear from the data presented how ATRX-Y1758C is functionally distinct from previously reported ΔATRX-null, or hypomorphic mutations.

This distinction lies at the core of the manuscript. The novelty depends on demonstrating that ATRX-Y1758C differs mechanistically from both conventional missense mutations and complete loss-of-functon(ΔATRX). From this perspective, a clear comparison between ATRX-KI and ATRX-KO models (and patient cells) is essential. If DEG analyses (and other cellular/molecular experiments as well) reveal differences in IFN responses or activation mechanisms between KI and KO, direct KI-KO or WT/KI/KO comparisons should be presented (e.g. S5fg/S7b). Conversely, if transcriptional responses are largely shared, the rationale for emphasising Y1758C as a special case is weak.

Despite the availability of patient-derived primary cells, no experiments directly compare control cells, P1/P2 (ATRX-Y1758C), and P4/P7 (ΔATRX) within the same experimental framework. For example, Fig.4a and Fig.S6a analyse patient-derived fibroblasts but exclude ΔATRX-null cases such as P4 and P7, making it difficult to directly assess whether the reported phenotypes extend to canonical ATRX deficiency.

Concerns regarding the pathogenicity of ATRX-Y1758C are further strengthened by the absence of basic validation experiments that could be easily feasible given the available patient material, such as whole-genome sequencing or western blot analyses of AGS relevant factors to exclude alternative diagnoses of interferonopathy. These issues were not resolved in the revison, where the authors state that additional experiments are unnecessary based on their prior experience. I fully recognise the authors' strong standing in this field; however, when claiming a non-canonical or atypical case, differential diagnosis remains important to exclude other possibilities.

Finally, if ATRX-Y1758C constitutively enhances IFN signalling, the functional dominance between wild-type ATRX and ATRX-Y1758C requires careful consideration. Although the carrier mother is reported to be unaffected (heterozygous), the relative dominance of wild-type versus mutant ATRX in the IFN signalling pathway has not been clearly indicated, and no explanation is provided for the absence of phenotype in the carrier.

For these reasons, I remain unconvinced that ATRX-Y1758C represents a mechanistically novel disease entity distinct from conventional ΔATRX-null cases. Although acceptance might be considered if additional evidence were provided, the current data leave open the possibility that key conclusions regarding pathogenicity and disease mechanism may not be robust.

Response to reviewer #2:

1. A major concern of the reviewer appears to focus on the assertion that the novelty of our work hinges on showing that the Y1758 mutation acts through a mechanism distinct from other ATRX mutations. To the best of our knowledge, this concern was not raised by the reviewer previously (and was not raised by any of the other three reviewers). Thus, the reviewer states:

"*I remain unconvinced that the novelty of the ATRX-Y1758C mutation -namely that it causes disease through a mechanism distinct from canonical ΔATRX-null type deficiency- has been adequately demonstrated.*"

"*… the authors need to show at the molecular level that the disease mechanism associated with ATRX-Y1758C is functionally distinguishable from ATRX-KO mutations …. as well as from previously reported ATRX missense mutations…*"

"*A key issue is whether the disease mechanism in the alpha-thalassaemia patients P1/P2 (ATRX-Y1758C) is fundamentally distinct from that in the ATRX syndrome patients P4/P7 used as controls, and from previously reported ATRX cases…*"

"*While these data support context-dependent regulation of IFN signalling downstream of ATRX dysfunction, they do not by themselves establish an ATRX-Y1758C-specific disease mechanism.*"

"*If IFN responses cannot be clearly distinguished between ΔATRX-null mutations and ATRX-Y1758C, support for novelty specific to ATRX-Y1758C remains limited.*"

"*…it remains unclear from the data presented how ATRX-Y1758C is functionally distinct from previously reported ∆ATRX-null, or hypomorphic mutations*."

"*The novelty depends on demonstrating that ATRX-Y1758C differs mechanistically from both conventional missense mutations and complete loss-of-functon(ΔATRX). From this perspective, a clear comparison between ATRX-KI and ATRX-KO models (and patient cells) is essential.*"

"*…if transcriptional responses are largely shared, the rationale for emphasising Y1758C as a special case is weak.*"

"*…I remain unconvinced that ATRX-Y1758C represents a mechanistically novel disease entity distinct from conventional ΔATRX-null cases.*"

Response: Beyond the fact that this mutation is associated with retained levels of ATRX protein, we do not claim that the Y1758 mutation is 'mechanistically distinct'. Rather, we show that this mutation acts as a loss-of-function, similar to other ATR-X syndrome causing mutations (here, for brevity, we highlight the data in Figure 1e and 1f where we show that, as previously reported for two other ATR-X syndrome causing mutations in the SNF2 domain, V1552F and K1650N, Y1758C ATRX is defective in ATPase and translocase activity). Thus, as stated in our initial rebuttal, "*Y1758C leads to a loss of ATPase activity as described for other 'classical' ATR-X syndrome associated mutations (PMID: 21505078; 14990586)*", also stating that *"we propose neither qualitative nor quantitative differences between the Y1758C substitution and other ATR-X syndrome associated mutations per se.*" Consistent with this, looking at transcriptional changes in BJ-hTERT fibroblasts KI (Y1758C) or KO for *ATRX*, we observed significant overlap in differentially expressed genes compared to control cells (Fig. 2f).

Overall, the novelty of our paper is twofold: 1) our demonstration, for the first time, that ATR-X syndrome causing mutations can be associated with an induction of type I IFN signalling (which induction is likely responsible for some aspects of the pathology shown in certain patients); 2) that, while this induced type I IFN signalling is cGAS-dependent, its induction is independent of cGAMP – thereby highlighting a previously unrecognised non-canonical role for cGAS in transcriptional regulation.

2. A second issue raised by the reviewer relates to the pathogenicity of the mutation. Thus, the reviewer states:

"*Concerns regarding the pathogenicity of ATRX-Y1758C are further strengthened by the absence of basic validation experiments that could be easily feasible given the available patient material, such as whole-genome sequencing or western blot analyses of AGS relevant factors to exclude alternative diagnoses of interferonopathy.*"

Response: With politely disagree that "*basic validation experiments*" might be reasonably employed to comprehensively "*exclude*" all known type I interferonopathies (assuming that the reviewer is envisaging studies of RNA, protein and activity levels relating to 50 + genotypes). We point out that both patients underwent whole exome sequencing, with no suspicious pathogenic variants identified in *AGS1-9* (or any other known type I interferonopathy associated gene). Further, the two cases that we describe do not present as typically seen in AGS or any other type I interferonopathy known to us.

More relevant, as detailed in our initial rebuttal, the evidence in favour of the variants in *ATRX* being causative are overwhelming, noting here that all three patients have had confirmatory genetic testing in accredited diagnostic laboratories in the UK, France and the Netherlands, and that the two patients tested have also been shown to exhibit a genomic DNA methylation signature diagnostic of ATRX syndrome (performed and signed off by an accredited diagnostic laboratory in the UK i.e. the NHS, and in the Netherlands). For the sake of completeness, I reproduce here the multiple arguments in favour of pathogenicity given in our original rebuttal letter:

Summarising, there are multiple lines of evidence supporting the pathogenicity of Y1758C: ***Genetic***: The variant is **very rare** i.e. it is **not present on more than 1,100,000 alleles** sequenced on gnomAD. In contrast, we identified two unrelated individuals (one from the UK, one from France) with this variant, both of whom manifest a similar neuroinflammatory phenotype associated with enhanced type I IFN signalling in blood. ***Phenotypic***: Patient 2 has **classical facial features** of ATRX syndrome. Further, this patient demonstrates a genomic **DNA methylation signature <u>diagnostic</u> of ATRX syndrome** (see PMIDs: 29304373; 28293299). ***Experimental***: Our functional studies show that the **Y1758C induces enhanced type I IFN signalling ex vivo** (using patient-derived cells) **and in vitro** (using a variety of cell models). Importantly, we have now expressed and purified full-length ATRX protein and used in vitro assays to show that the **Y1758C substitution is hypomorphic for ATPase activity** (as described in Mitson et al. (PMID: 21505078) and Tang et al. (PMID: 14990586) for other mutations of

ATRX associated with retained protein expression). Further, we show that this loss of ATPase activity is associated with a **marked reduction of translocase activity**.

3. "*As also noted by the authors, the impact of ATRX abnormalities on IFN responses varies widely depending on cell type and context. Consistent with this, prior studies report both suppression and activation of IFN signalling upon ATRX loss, indicating that the field remains controversial (Floyd, J Clin Invest. 2023; Wang, Am J Cancer Res 2023; Whitford, Nature Communications 2025)*."

Response: Rather than these observations being 'controversial', we consider them interesting. The divergent effects on IFN signalling likely reflect a fundamental role of ATRX in shaping chromatin homeostasis, with the specific chromatin state of a given cell (type) likely determining how ATRX loss-of-function ultimately impacts transcription.

4. Another concern of the reviewer is the lack of correlation between ATRX protein levels in primary fibroblasts and the activation status of IFN signalling. Thus, the reviewer states:

"*This variability is clearly illustrated by the comparison between P4 and P7 in Fig1. Fig.1a and Fig.1b show no clear correlation between ATRX protein levels (Fig. S3) and IFN score or IFNB1 expression. Although both P4 (W222C, associated with protein instability) and P7 (a frameshift null mutation) lack detectable ATRX protein (ΔATRX), their IFN responses are opposite (P4 suppressed, P7 enhanced). Thus, IFN transcriptional responses are not uniquely determined by ATRX protein status, and IFN readouts should be interpreted with caution as mechanistic endpoints*."

"*This variability is clearly illustrated by the comparison between P4 and P7 in Fig1. Fig.1a and Fig.1b show no clear correlation between ATRX protein levels (Fig. S3) and IFN score or IFNB1 expression. Although both P4 (W222C, associated with protein instability) and P7 (a frameshift null mutation) lack detectable ATRX protein (ΔATRX), their IFN responses are opposite (P4 suppressed, P7 enhanced). Thus, IFN transcriptional responses are not uniquely determined by ATRX protein status, and IFN readouts should be interpreted with caution as mechanistic endpoints*."

Response: We believe that the reviewer is referring to Figure 2, and the data in Figure S3. We do not claim a direct inverse correlation between ATRX protein levels and IFN signalling. We agree that the data on IFN signalling in patient primary fibroblasts show some variability, which we suggest relates to two factors: firstly, primary cells are notoriously difficult to work with, leading us to turn to more 'controlled' cellular models. Thus, in our manuscript we state that "*Given the potential variability of genetic background in available patient material, we introduced the Y1758C mutation (referred to as knock-in (KI)) into BJ-5ta immortalised fibroblasts (referred to as BJ-hTERT), and also generated ATRX knock-out (KO) clones using CRISPR/Cas9 technology*."; secondly, as discussed in our original rebuttal, the available evidence suggests that variability in inflammatory signalling reflects the stochastic nature of perturbed chromatin accessibility and gene expression. This phenomenon is already well-established in the context of ATRX; best illustrated by the finding that only a proportion of patients with germline mutations in *ATRX* manifest indices of alpha thalassemia (PMID: 18409179). Following up on this observation, in demonstrating a function for ATRX on both heterochromatin and euchromatin, Richard Gibbons (a co-author on our paper) (PMID: 35710802) went on to show that the role of ATRX at the well-characterized α-globin locus in erythroid cells leads to stochastic changes in gene expression and cell fate in only a subset of cells (mediated through interdependent effects on DNA replication, chromatin modification and chromatin accessibility).

5. A final comment is made about the absence of a phenotype in female carriers:

"*if ATRX-Y1758C constitutively enhances IFN signalling, the functional dominance between wild-type ATRX and ATRX-Y1758C requires careful consideration. Although the carrier mother is reported to be unaffected (heterozygous), the relative dominance of wild-type versus mutant ATRX in the IFN signalling pathway has not been clearly indicated, and no explanation is provided for the absence of phenotype in the carrier.*"

Response: We do not understand the term "functional dominance" used by the reviewer, which is not a term that we use in the manuscript. Rather the mutations studied in our manuscript confer a loss of ATRX function. We state in the introduction of our paper that "*mutations in ATRX cause a sex-linked recessive disorder*" and note here that skewing of X-chromosome inactivation is the rule in ATRX (PMID: 17503331).

Reviewer #3 (Remarks to the Author):

The revised manuscript has been significantly improved, and the authors have successfully addressed the key concerns raised during the initial review. The additional data and clarified explanations provided in this version have strengthened the overall impact and clarity of the study.

Response to reviewer #3: We sincerely thank the reviewer for their favourable opinion.

Reviewer #4 (Remarks to the Author):

This paper by A El-Daher and colleagues describes three patients with specific missense mutations in the ATRX ATPase domain. As a specific feature of these ATRX patients they report on a persistent upregulation of interferon-stimulated gene expression in the peripheral blood and in patient-derived fibroblasts. They go on to use CRISPR/Cas-modified cells to demonstrate that cGAS is relevant for ISG upregulation, however, it is postulated that cGAS mediates this effect via a non-conventional nuclear mechanism that is independent from DNA sensing.

I have the following concerns with the results of this paper:

1. Although the identification of ATRX patients with upregulation of ISGs and IFNb is interesting, the increases as reported in figure 1 are rather weak. The IFN scores are in the range of 3 to a maximum of 6fold which is considerably lower compared to a panel of patients with well characterized interferonopathies (see eg PMID: 24183309). The authors associate these slightly increased scores to features of neuroinflammation detected in the respective patients. The only potentially causal link is the response to ruxolitinib as observed in a singular patient. Furthermore, only a subset of ATRX patients exhibits signs of ISG upregulation.

2. Mechanistically, the authors propose that ATRX deficiency disrupts the pattern of DAXX and H3.3 and this critically depends on a non-canonical nuclear role of cGAS. However, it remains entirely unclear how cGAS mediates this effect. Furthermore, the differences shown in figure 3 are not very impressive.

3. To my opinion, alternative mechanisms could account for the increased ISG scores observed in this study and the authors need to address this:

- ATRX is well known to act as a repressor of endogenous retroviruses (e.g. PMID: 26012739). Thus, loss of ATRX function may upregulate ERVs and this, in turn, could lead to ISG upregulation independent of RIG1 or cGAS (see PMID: 26941318).

- Loss of ATRX is known to be the prerequisite for the development of ALT. ALT, in turn, generates extrachromosomal telomere repeat DNA which acts as a potent stimulator of IFNb and the type I IFN response (PMID: 29106411).

Both of these alternative mechanisms might also explain why only some ATRX patients show signs of ISG upregulation since ERVs are very diverse between individuals and the development of ALT also depends on (so far unknown) secondary events (see PMID: 40292316). In this context, it might turn out to be problematic that the authors used telomerase immortalized BJ fibroblasts for all their CRISPR/Cas engineering since expression of telomerase affects the formation of c-circle DNA known to act pro-inflammatory.

4. Furthermore, CRISPR Cas engineering requires a prolonged selection of cells. Thus, not ATRX deficiency by itself but secondary events fostered by the well documented genome destabilizing effects of ATRX depletion (PMID: 40292316) might account for the “stochastic” occurrence of ISG upregulation as described in this study.

Response to reviewer #4:

1. “*Although the identification of ATRX patients with upregulation of ISGs and IFNb is interesting, the increases as reported in figure 1 are rather weak. The IFN scores are in the range of 3 to a maximum of 6fold which is considerably lower compared to a panel of patients with well characterized interferonopathies (see eg PMID: 24183309)*”

Response: We politely disagree with the reviewer on this point. The level of ISG signalling observed in whole blood of P1 and P2 is dramatic and sustained, with IFN scores (Figure 1d and Figure S1f) as high or higher than in any of the other type I interferonopathies that we study. Differences in IFN score

and *IFNB1* expression in fibroblasts are less striking (Figure 2a, b), a well-recognised phenomenon – see the figure below adapted from Lepelley et al. J Exp Med 2021 (PMID: 34387651) relating to a type I interferonopathy due to mutations in *ATAD3A* – likely reflecting the fact that fibroblasts are not 'primary immune cells.'

[editorial note: third party material redacted]

"*only a subset of ATRX patients exhibits signs of ISG upregulation.*"

Response: We agree that the inflammatory phenotype in ATRX patients is variable, which we suggest relates to two factors: firstly, primary cells are notoriously difficult to work with, leading us to turn to more 'controlled' cellular models. Thus, in our manuscript we state that "*Given the potential variability of genetic background in available patient material, we introduced the Y1758C mutation (referred to as knock-in (KI)) into BJ-5ta immortalised fibroblasts (referred to as BJ-hTERT), and also generated ATRX knock-out (KO) clones using CRISPR/Cas9 technology.*"; secondly, as discussed in our original rebuttal, the available evidence suggests that variability in inflammatory signalling reflects the stochastic nature of perturbed chromatin accessibility and gene expression. This phenomenon is already well-established in the context of ATRX; best illustrated by the finding that only a proportion of patients with germline mutations in ATRX manifest indices of alpha thalassemia (PMID: 18409179). Following up on this observation, in demonstrating a function for ATRX on both heterochromatin and euchromatin, Richard Gibbons (a co-author on our paper) (PMID: 35710802) went on to show that the role of ATRX at the well-characterized α-globin locus in erythroid cells leads to stochastic changes in gene expression and cell fate in only a subset of cells (mediated through interdependent effects on DNA replication, chromatin modification and chromatin accessibility).

2. "*Mechanistically, the authors propose that ATRX deficiency disrupts the pattern of DAXX and H3.3 and this critically depends on a non-canonical nuclear role of cGAS. However, it remains entirely unclear how cGAS mediates this effect. Furthermore, the differences shown in figure 3 are not very impressive.*"

Response: While we agree that how precisely cGAS mediates the changes seen in the context of ATRX dysfunction remains unclear, we consider that our observations represent a considerable advance in highlighting this previously unrecognised non-canonical function. We are uncertain to what exactly the reviewer is referring in relation to Figure 3, since we consider the data in this figure to clearly demonstrate a requirement for cGAS in mediating the enhanced IFN response observed in the context of ATRX deficiency (for example, note that the increased IFN score of ~ 40 times in cGAS proficient cells shown in Figure 2d is completely abrogated in cGAS depleted cells (Fig. 3c).

3. "*To my opinion, alternative mechanisms could account for the increased ISG scores observed in this study and the authors need to address this:*

We agree with the reviewer that other mechanisms might contribute to the induction of type I IFN signalling in the context that we are studying in our manuscript. However, we do hold that we present compelling data that the IFN response that we see is cGAS-dependent but independent of cGAMP.

Considering the mechanisms suggested by the reviewer:

a. *– "ATRX is well known to act as a repressor of endogenous retroviruses (e.g. PMID: 26012739). Thus, loss of ATRX function may upregulate ERVs and this, in turn, could lead to ISG upregulation independent of RIG1 or cGAS (see PMID: 26941318)."*

Response: In the paper cited by the reviewer (PMID: 26941318), ERVs are described as interferon-inducible enhancers, where STAT1/IRF1 can bind and therefore enhance the expression of certain immune genes. Our understanding is that this paper does not focus on the transcriptional expression of the ERVs themselves.

While ATRX is well known to repress ERVs, this function was mostly observed in stem cells, where the role of ATRX in maintaining DNA methylation at early developmental stages is critical to silence repetitive elements (PMIDs: 25938714, 26340527). Given that the functions of ATRX are likely to be cell-type and context specific, we have no evidence that ATRX loss activates ERVs in terminally differentiated cells, such as the fibroblasts that we use. More relevant still, in most situations currently described to date, ERVs trigger IFN signalling through a cGAMP-dependent mechanism (e.g. PMIDs: 36610399; 38991060; 39446896), a scenario that we show does not apply in the situation that we describe.

While we agree with the reviewer that IFN signalling might, theoretically, be triggered through pathways independent of RIG-I or cGAS, our experimental data consistently demonstrate a clear dependency on cGAS in the context that we have studied (Fig. 3c-f).

b. – *"Loss of ATRX is known to be the prerequisite for the development of ALT. ALT, in turn, generates extrachromosomal telomere repeat DNA which acts as a potent stimulator of IFNb and the type I IFN response (PMID: 29106411)… it might turn out to be problematic that the authors used telomerase immortalized BJ fibroblasts for all their CRISPR/Cas engineering since expression of telomerase affects the formation of c-circle DNA known to act pro-inflammatory*."

Response: The reviewer is of course correct to highlight the link between ATRX and ALT. However, we point out that the extrachromosomal telomere repeat (ECTR) DNA inducing type I IFN signalling described in PMID 29106411 is cGAS-STING and phospho-IRF3 mediated (as shown in their Figure 3) – which is not the case in our data. In agreement, we did not detect cytoplasmic telomere foci by Telomere-FISH (Fluorescence *In Situ* Hybridization). This contrasts with the ALT positive cells shown in Figure 1a in PMID 29106411. These data, now added to our revised manuscript as Fig.S9b, argue against the leakage of telomere fragments into the cytosol activating canonical DNA sensing.

4. "*Furthermore, CRISPR Cas engineering requires a prolonged selection of cells. Thus, not ATRX deficiency by itself but secondary events fostered by the well documented genome destabilizing effects of ATRX depletion (PMID: 40292316) might account for the "stochastic" occurrence of ISG upregulation as described in this study*."

Response: We appreciate the reviewer perspective regarding the potential for secondary events, such as genome instability or clonal selection, to influence the upregulation of type I IFN signalling. We have addressed these concerns as follows:

- Genome instability: We acknowledge that ATRX loss can compromise genomic stability (PMIDs: 18227278, 23563309), and we have explored this in our own work (Figure S9). However, several lines of evidence in our study suggest that DNA damage is not the primary driver of the ISG upregulation observed here. Thus, in both patient-derived cells and our ATRX-deficient models, we did not detect an increase in cGAMP levels, which would be expected if genomic DNA fragments were activating the canonical cGAS enzymatic pathway. Moreover, we demonstrate that the IFN signalling in our model is independent of cGAS catalytic activity (the canonical DNA-sensing function of cGAS typically relies on its enzymatic activity). Together, these results indicate that genome instability does not contribute majorly to the enhanced IFN signalling observed in the ATRX deficient cells that we have studied.

- Clonal selection and stochasticity: To mitigate the risk of clonal selection bias or stochastic secondary mutations, we utilized 5 to 6 independent CRISPR-engineered clones for each genotype and experiment. By prioritizing only the phenotypes and transcriptomic changes that were consistently present across all independent clones, we have minimized the likelihood that our observations are the result of random mutational burden or prolonged selection pressure.

Fig. 2d

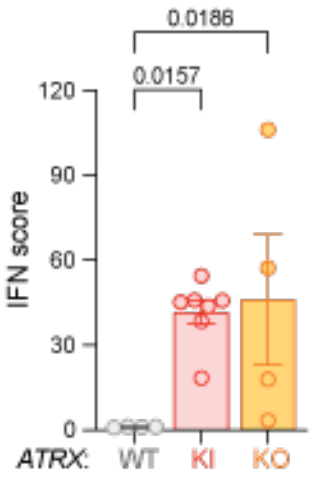

Fig. 2e

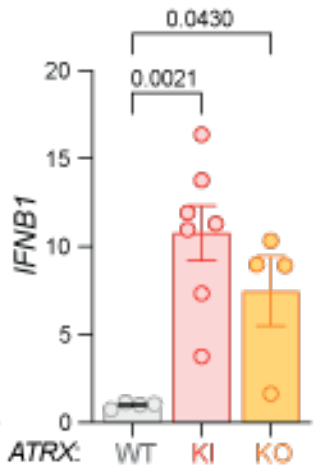

Fig. 2f

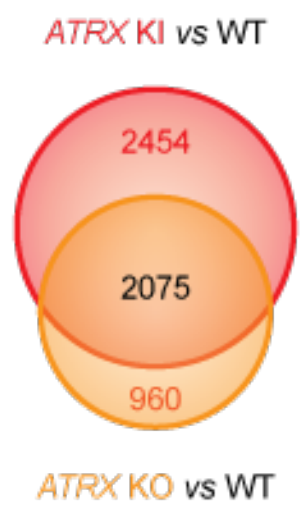

Fig. 2g

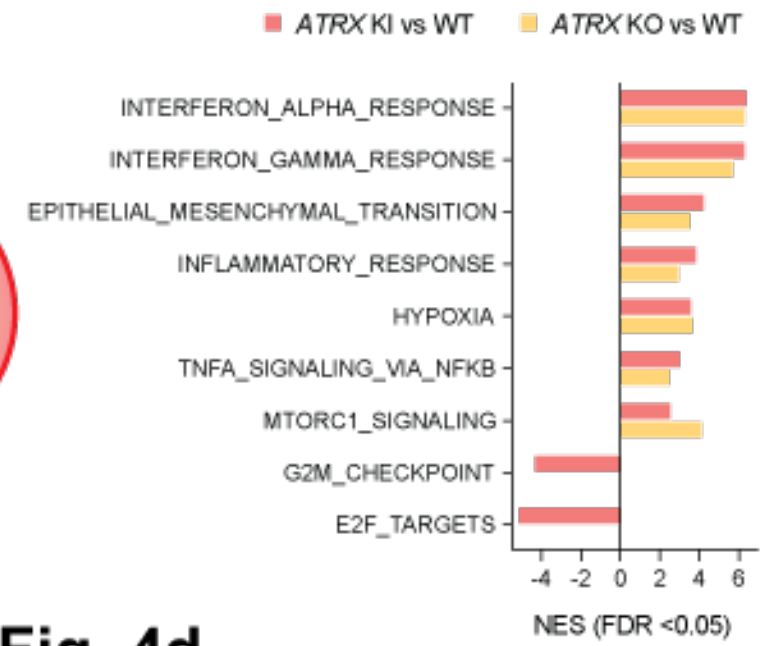

Fig. 3a

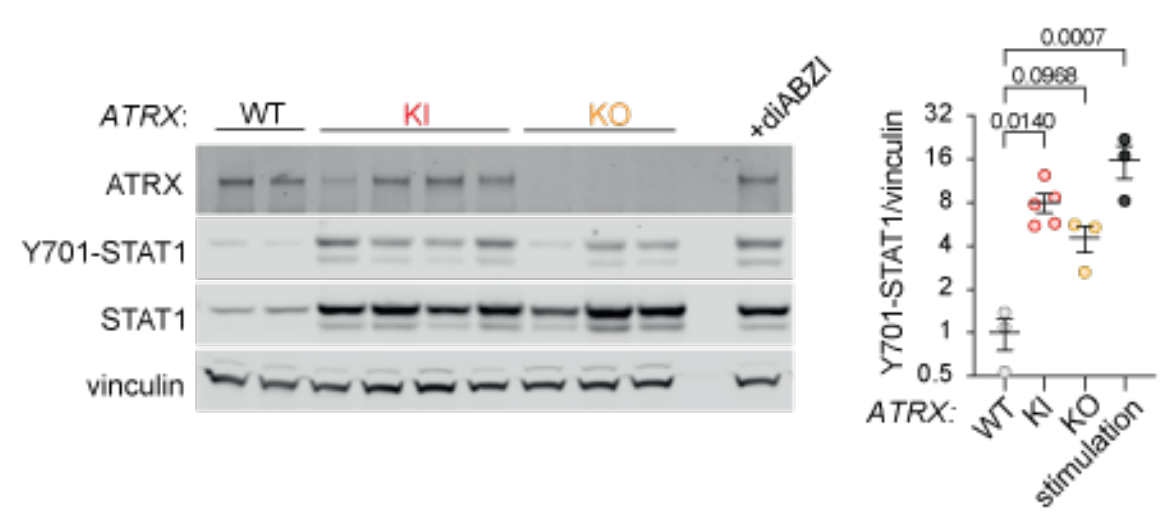

Fig. S4a

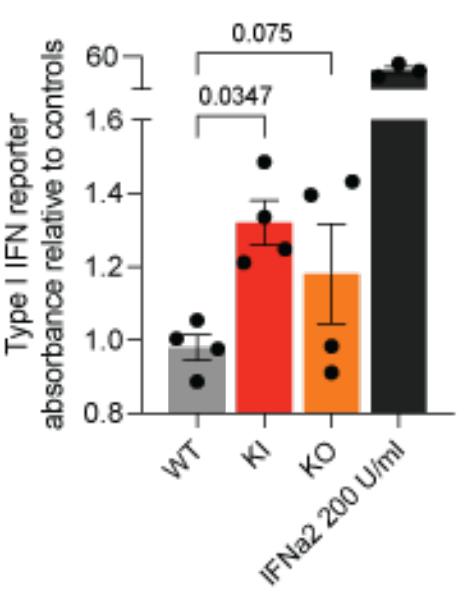

Fig. 4b

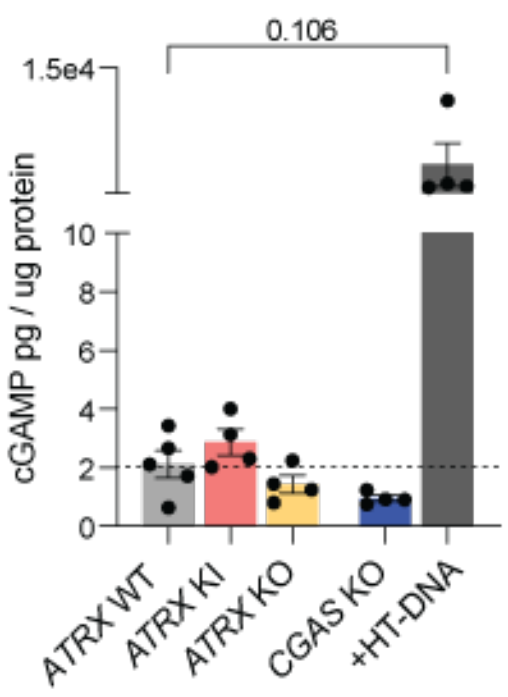

Fig. 4d

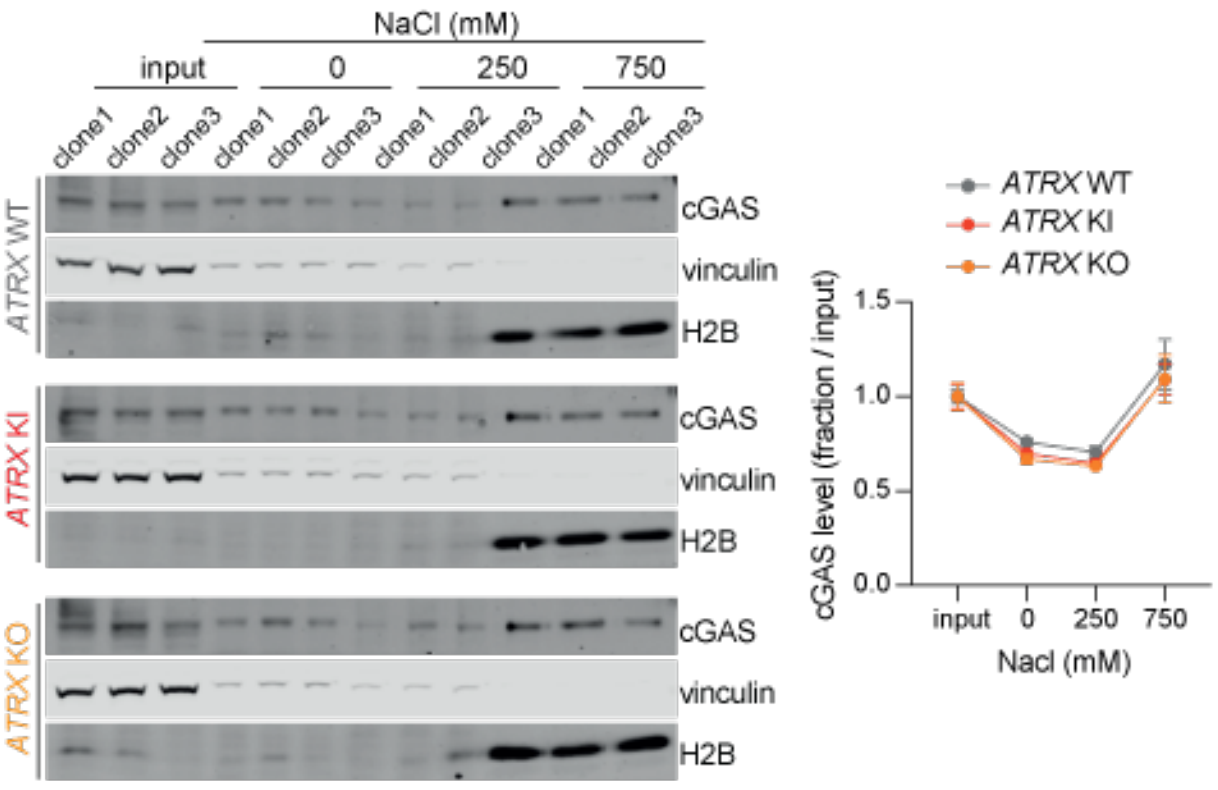

Fig. 4f

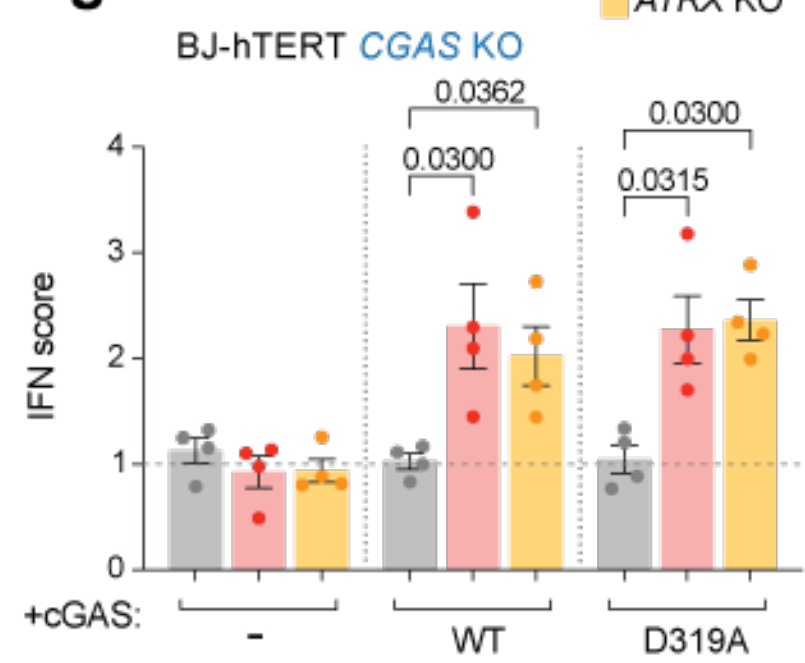

Fig. S6d

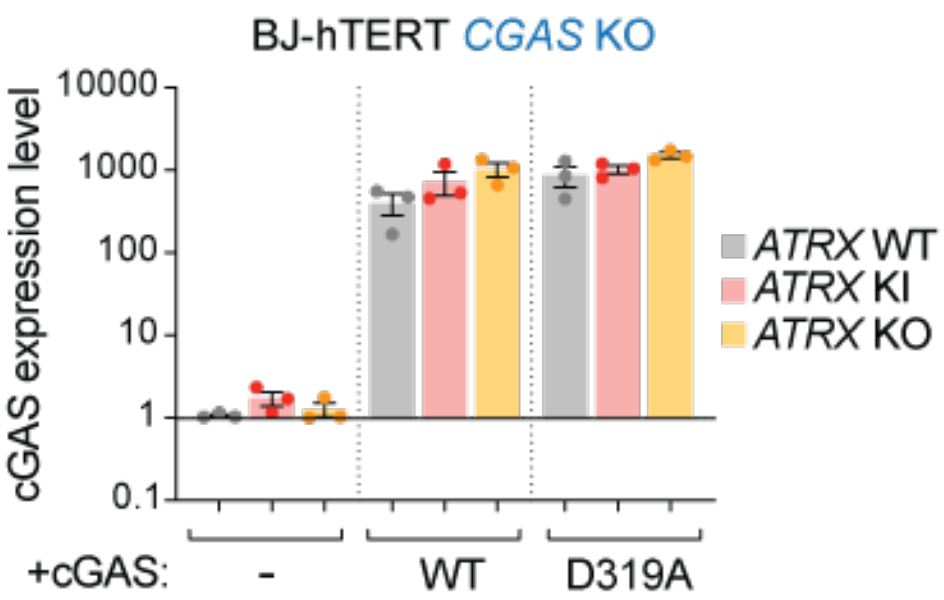

Fig. 4g

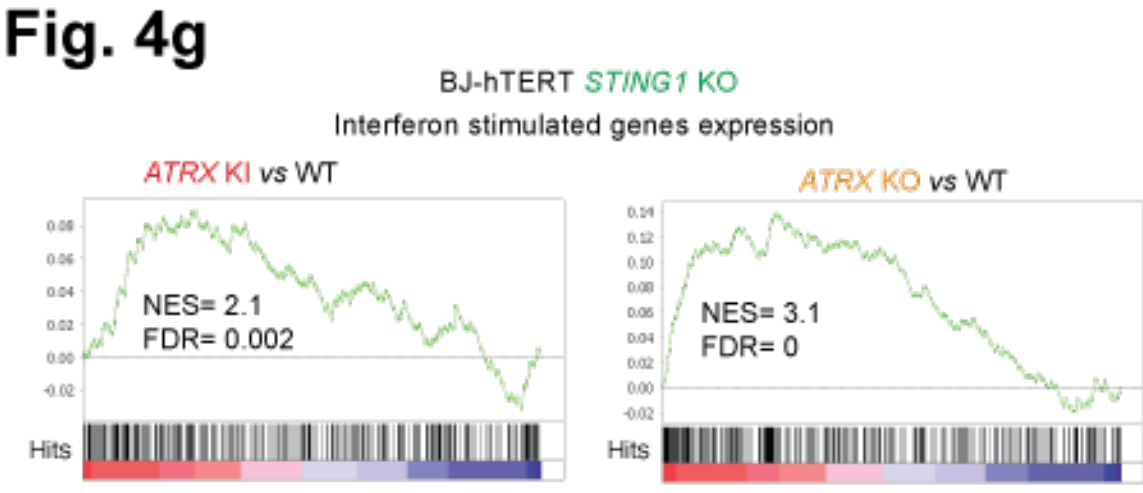

Fig. 4h

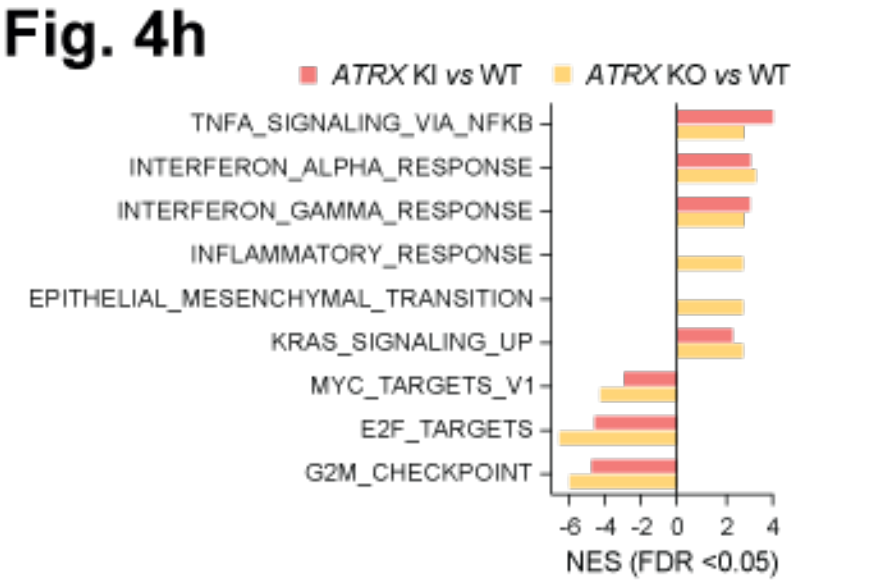

Fig. S5f

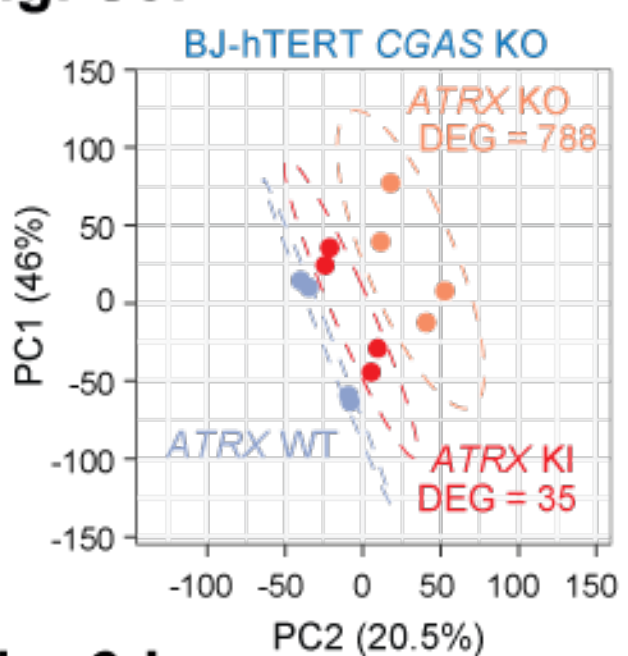

Fig. S5g

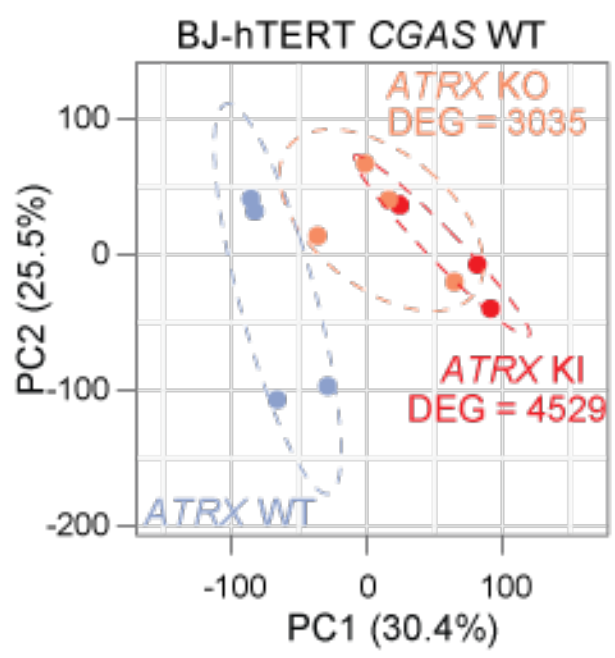

Fig. 6b

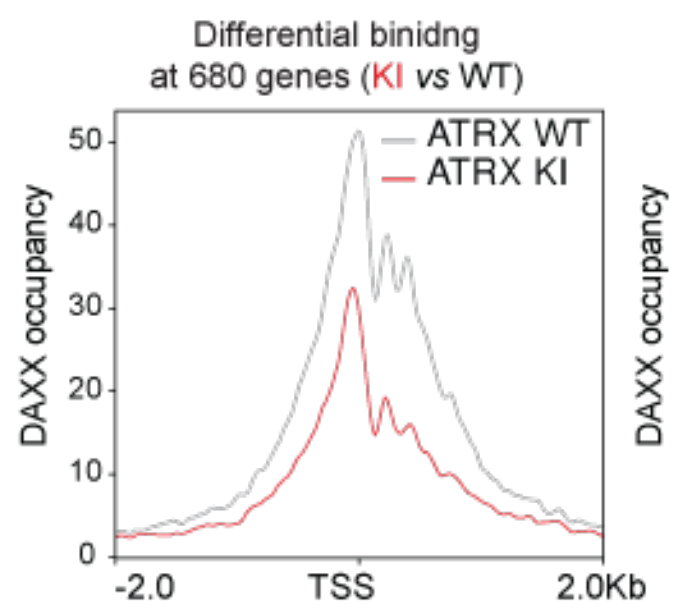

Fig. 6c

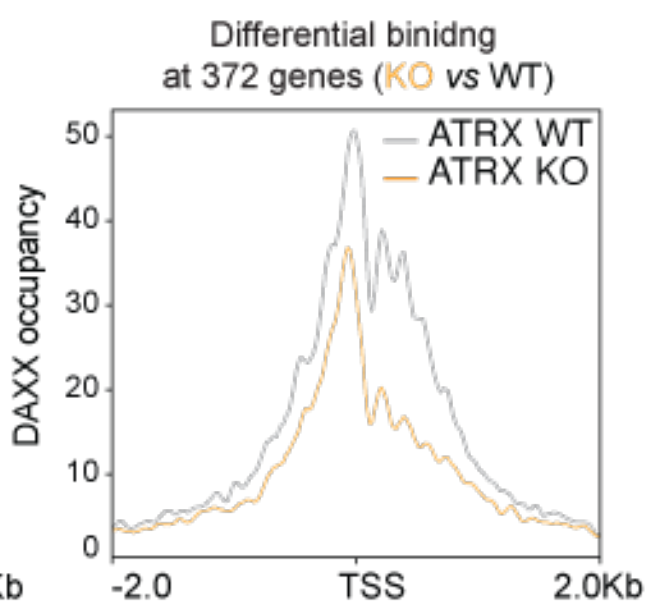

Fig. 6d

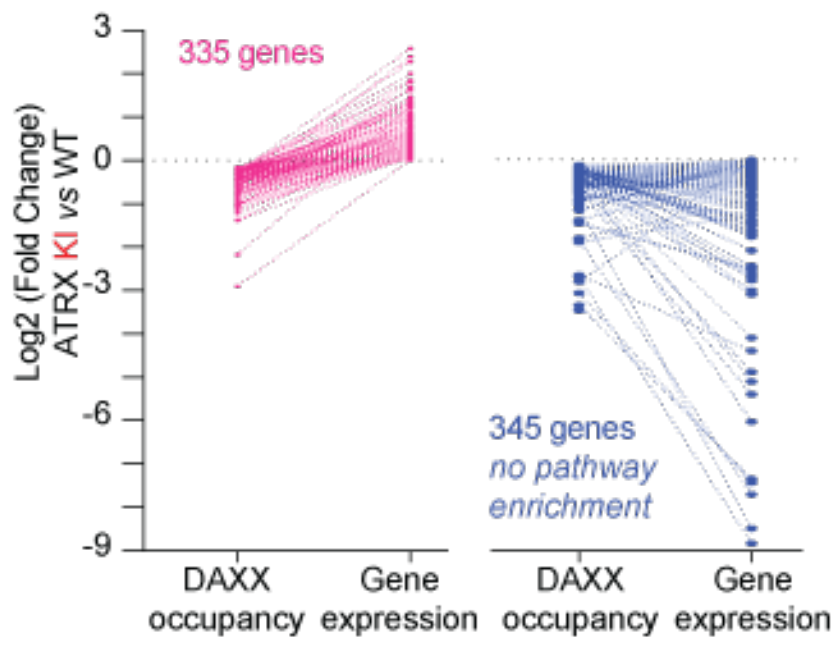

Fig. 6e

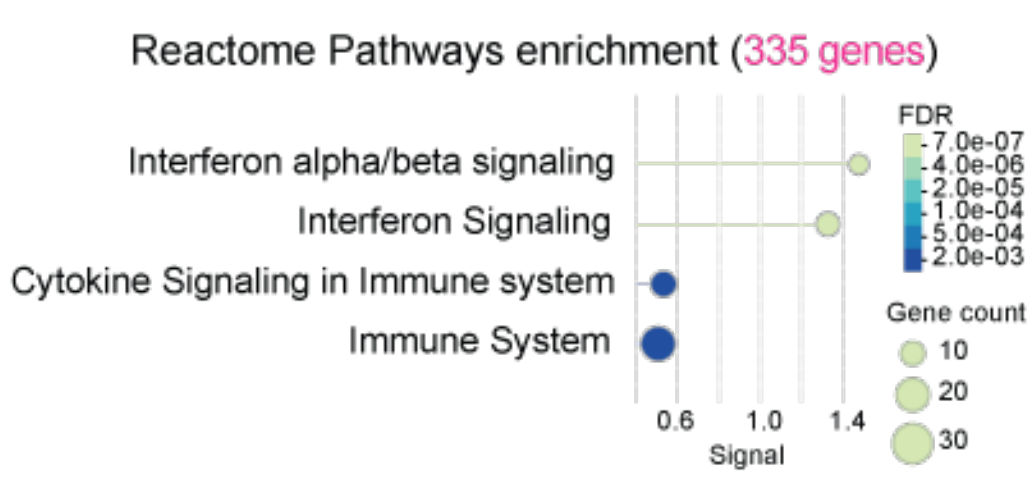

Fig. 6h

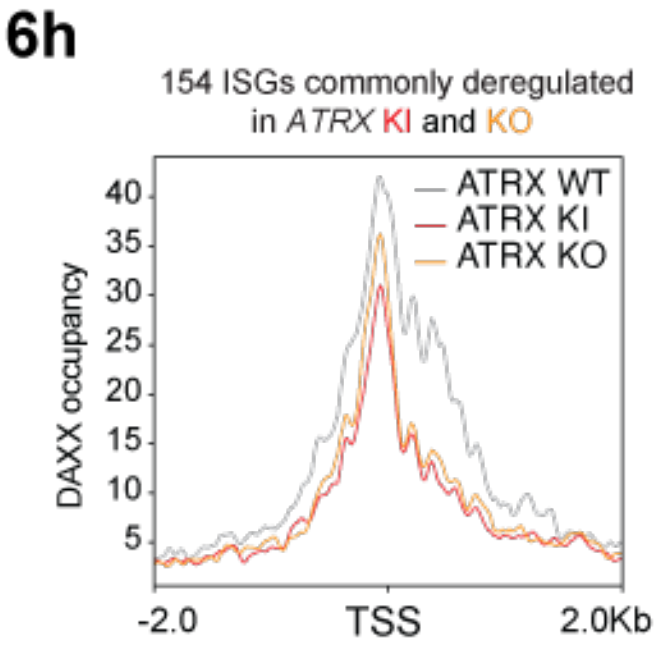

Fig. 6f

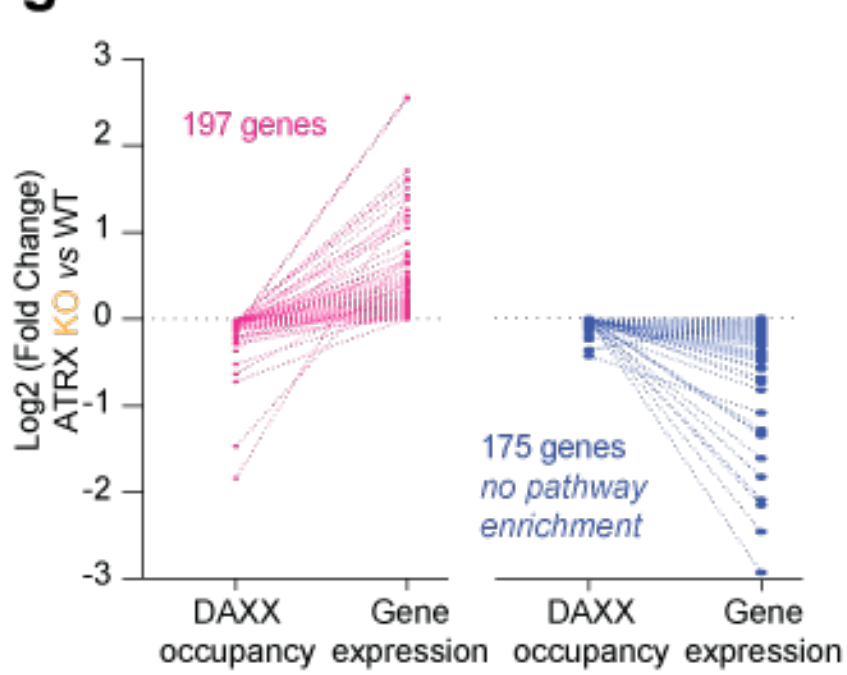

Fig. 6g

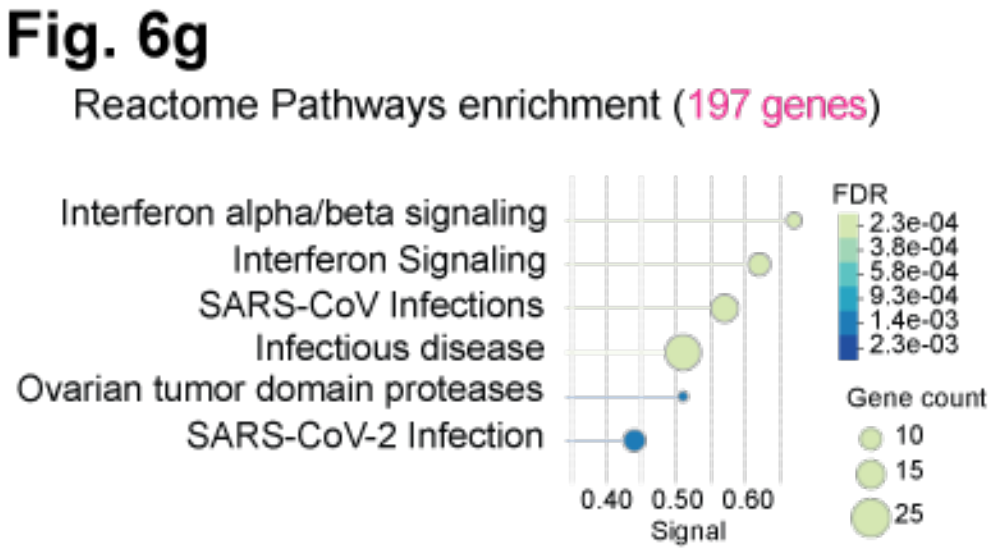

Fig. 6i

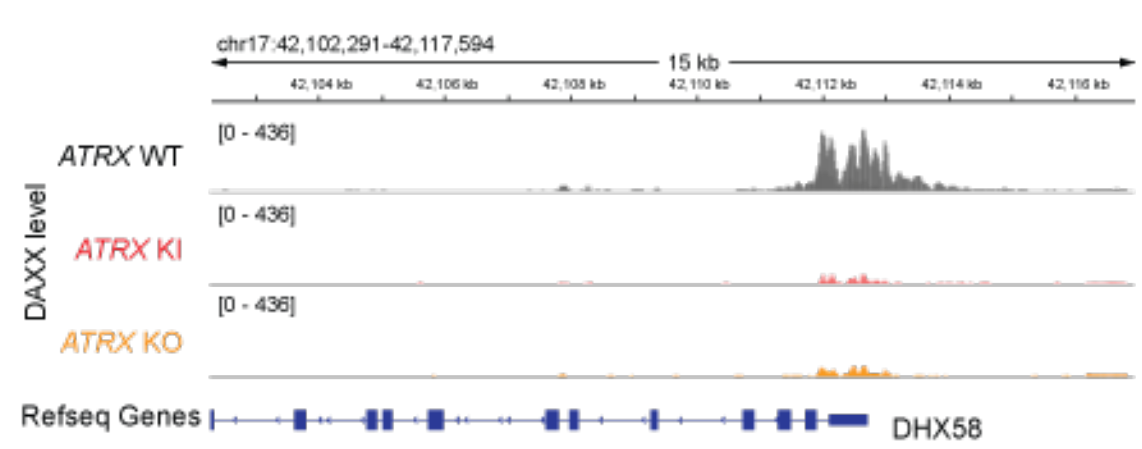

Fig. 7b

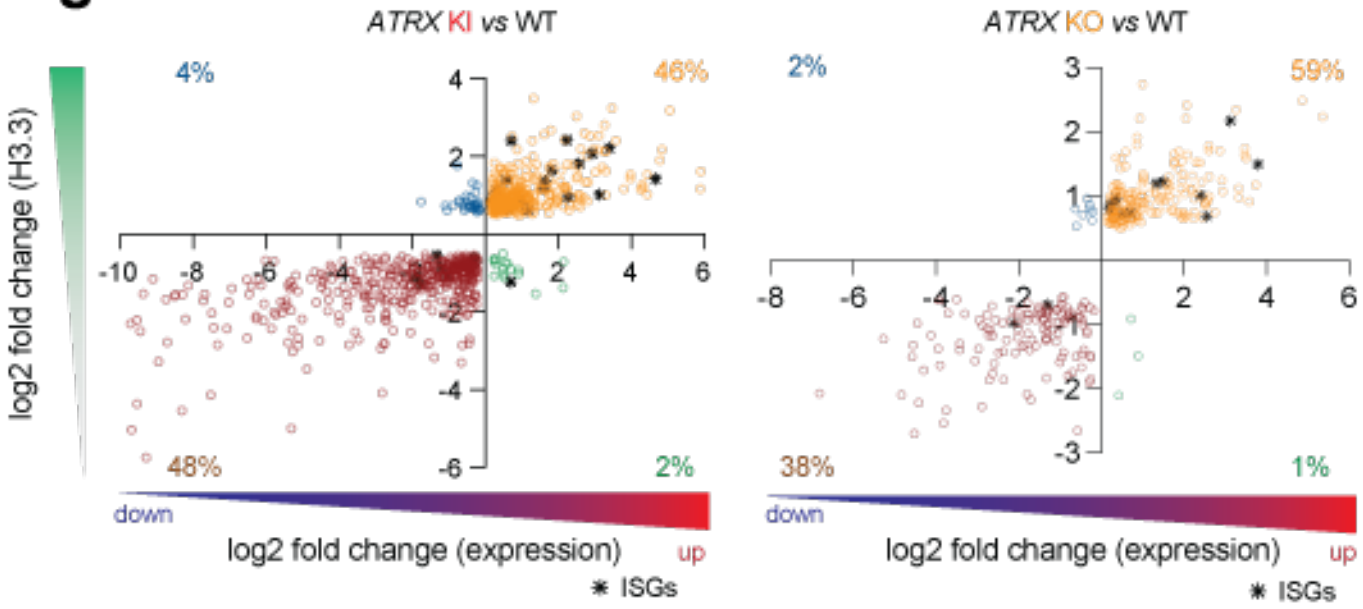

Fig. 7b

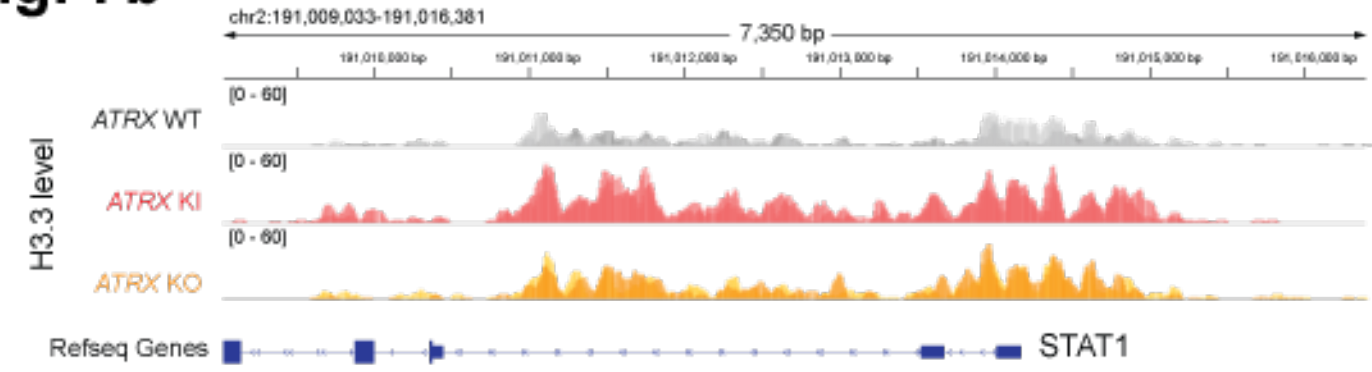

Fig. 7d

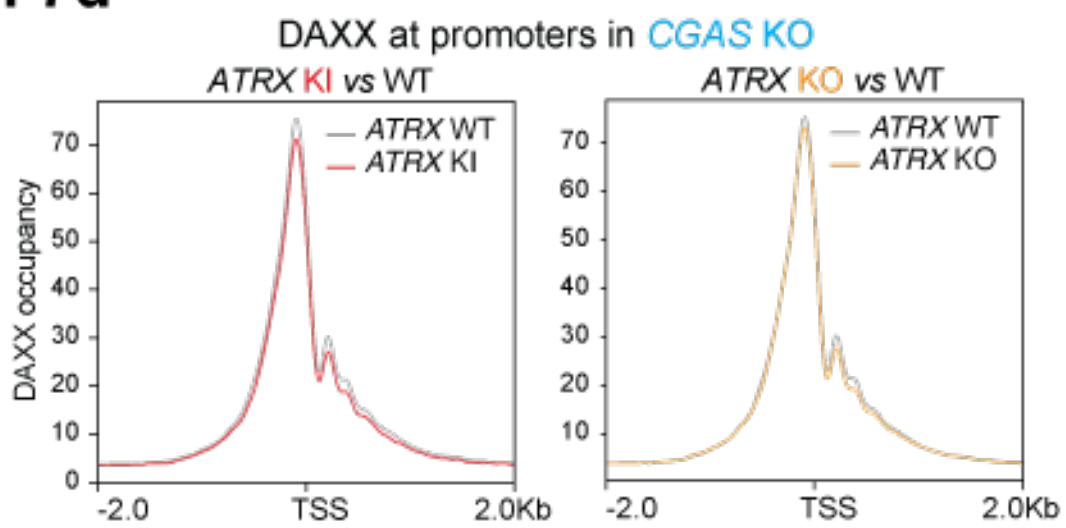

Fig. S8a

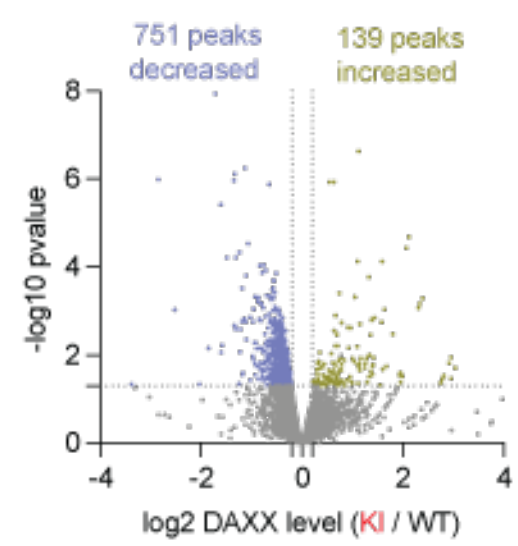

Fig. S8b

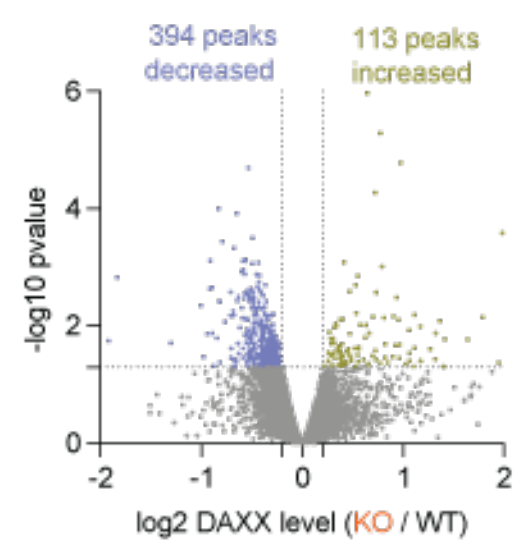

Fig. S8c

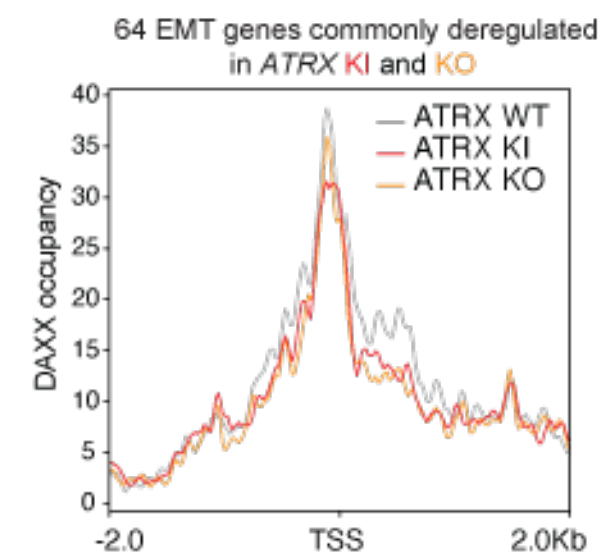

Fig. S8d

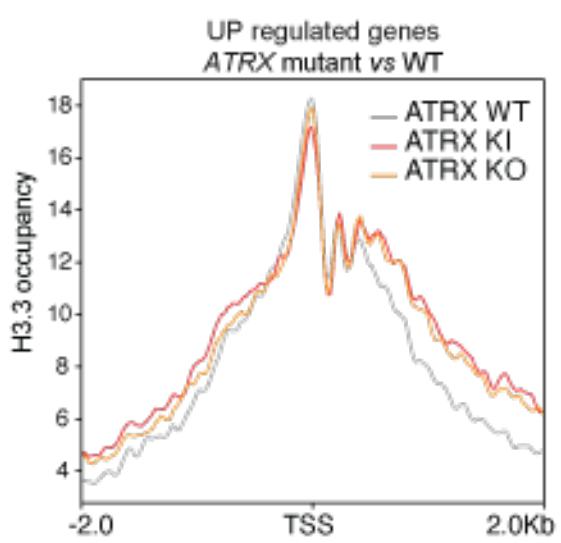

Fig. S8e

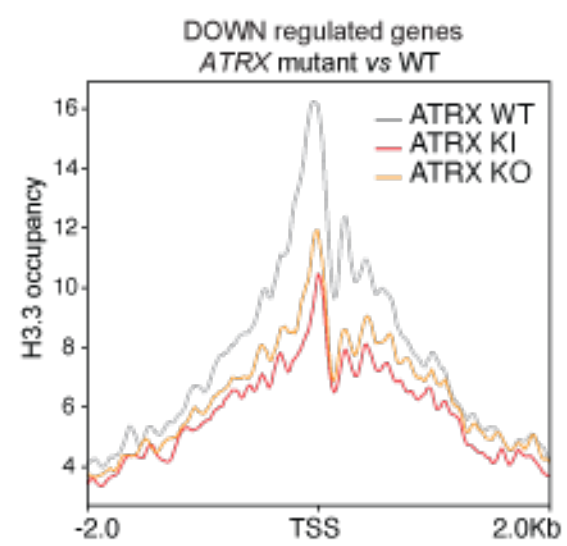

Fig. S8f

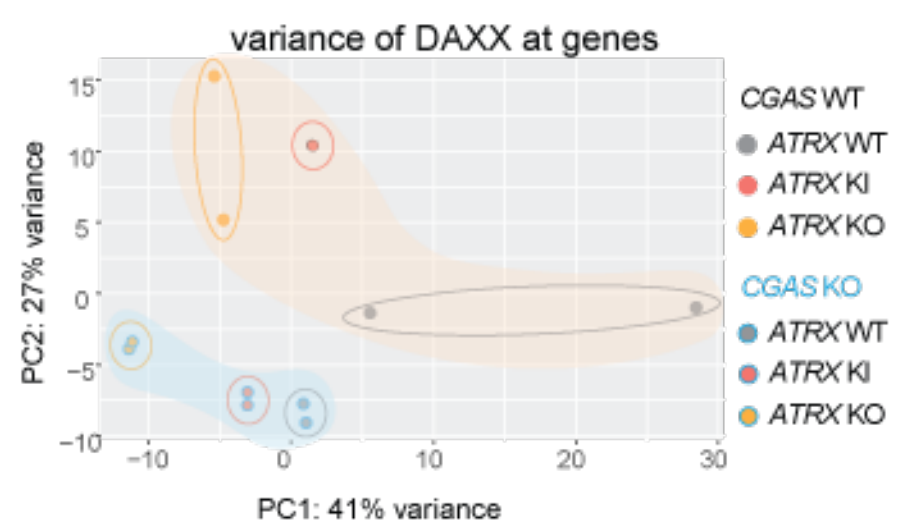

Fig. S8g

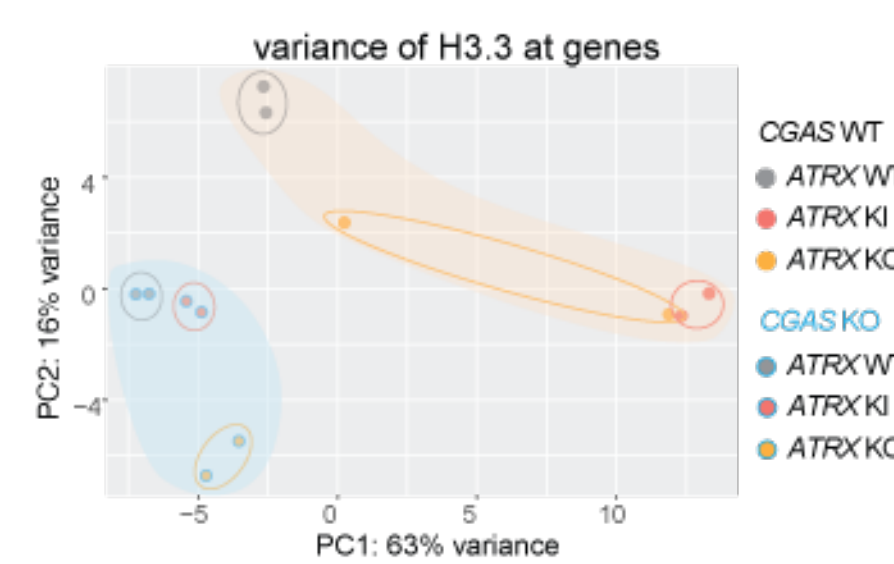

Fig. 2h

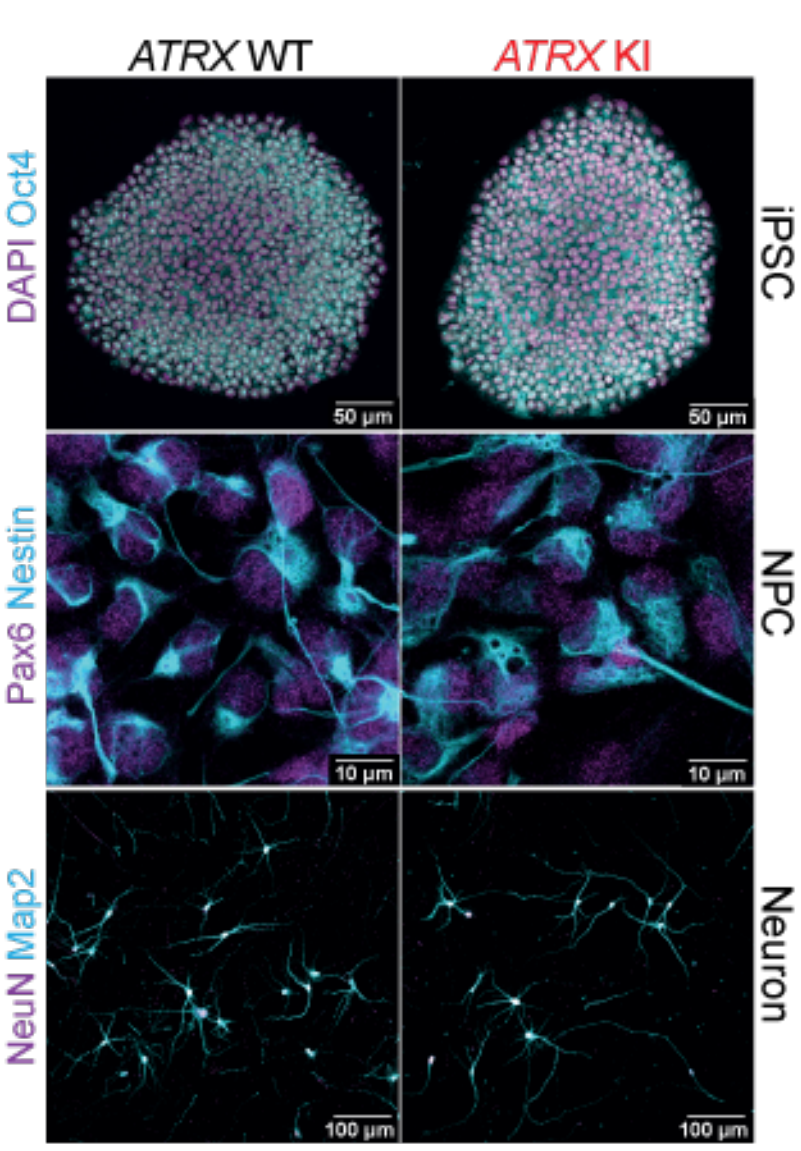

Fig. 2j

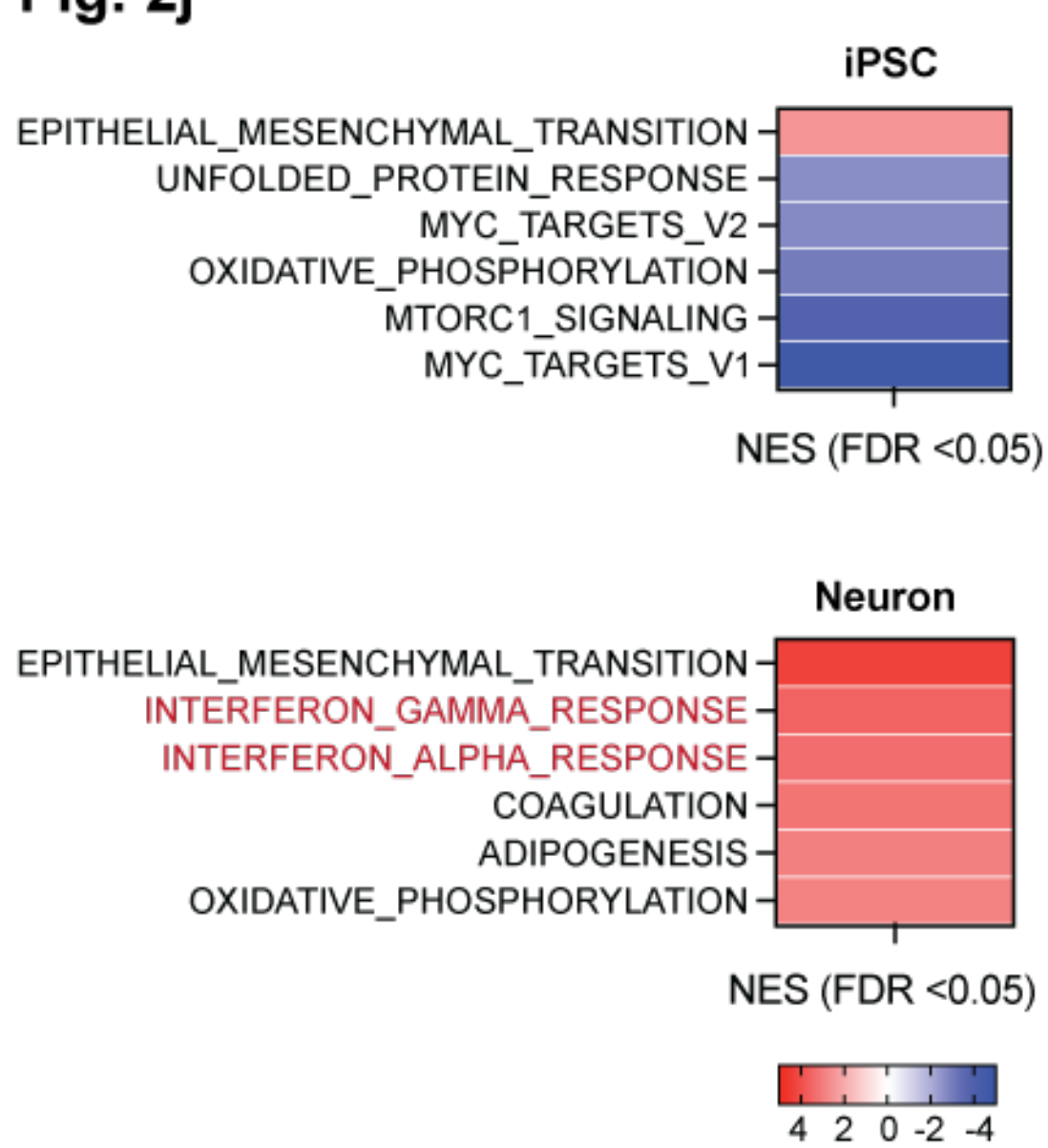

Fig. S3f

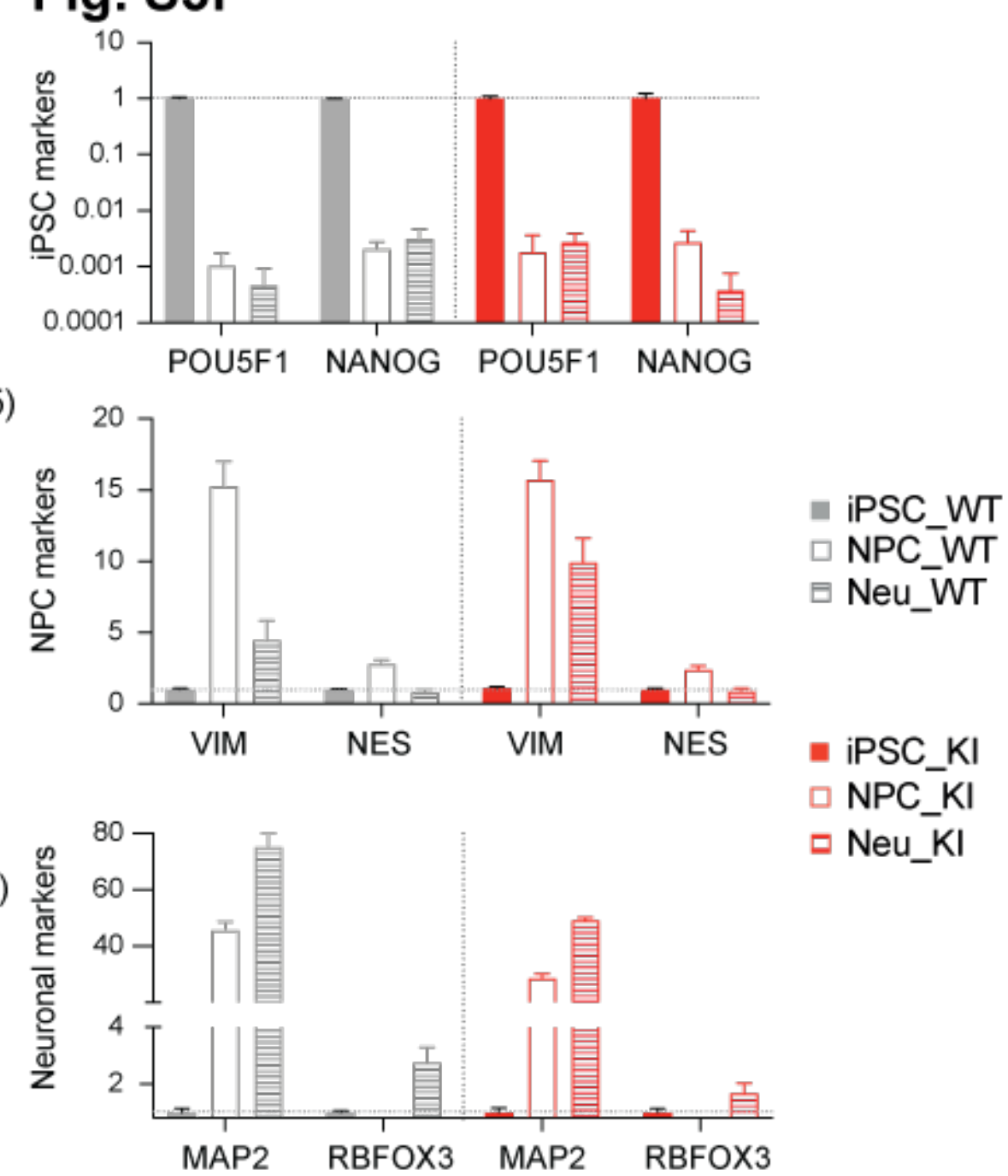

Reviewer #2 (Remarks to the Author):

In the final revision, there are few substantive additional experiments or analyses addressing the major concerns raised previously by Reviewers#2 and #4.

This revision partially addresses Reviewer#4's concern regarding ALT/telomere DNA sensing. However, I do not consider the other concerns shared by Reviewers#2 and #4 to have been substantially resolved. The authors' response consists mainly of post hoc explanations in the rebuttal, and the central concerns of Reviewers#2 and #4 are not sufficiently addressed.

At present, the data support only a limited conclusion: cGAS-dependent and cGAMP/STING-independent ISG transcriptional dysregulation is observed in some cells and patients with ATRX dysfunction. These data do not establish that this is a phenotype specific to ATRX-Y1758C, nor do they justify generalisation to all patients with ATR-X syndrome. An overlooked variant in a known interferonopathy-related gene has not been fully excluded.

By contrast, stronger conclusions such as "ATRX-Y1758C defines a mechanistically distinct new interferonopathy", "ATRX deficiency generally drives type I interferon signalling", or "cGAS directly regulates ATRX-mediated chromatin remodelling" are not sufficiently supported by the current data.

Therefore, I cannot support acceptance in the current form. At minimum, the title, abstract and conclusion should be substantially toned down, and the unresolved alternative mechanisms and limitations in differential diagnosis should be clearly stated. If additional experiments are requested, WGS and RNA-seq of P1/P2 should at least be performed, together with confirmation of the expression of representative AGS/interferonopathy-related proteins.

The final decision as to whether the ATRX-Y1758C variant and its associated phenotype have sufficient novelty for publication in Nature Communications should then be left to the editor.

Concerns shared by Reviewers#2 and #4

1. A novel disease mechanism specific to ATRX-Y1758C has not been demonstrated

One of the fundamental concerns shared by the Reviewers is that it remains unclear whether the central claim of this study is a novel disease mechanism specific to ATRX-Y1758C, or an IFN abnormality associated with ATRX dysfunction that may also be observed more generally in known ATRX-deficient conditions.

In the rebuttal, the authors state that they do not claim Y1758C to be mechanistically distinct. The title and abstract still state "ATRX Deficiency Drives Aberrant Type I Interferon Signalling Through cGAS-Dependent Transcriptional Dysregulation", and strongly describe ATRX loss-of-function mutations as driving enhanced type I interferon signalling. The novelty is clearly different depending on whether ATRX-Y1758C can be defined as a new disease entity, or whether IFN abnormalities represent an additional phenotype that may occur more generally in ATRX deficiency. In addition, several previous studies, including Floyd JCI (2023), have reported impaired cGAS/STING signalling in ATRX deficiency, which is opposite to the authors' claim, as also stated in my previous comments. Therefore, a rigorous assessment is required, and the authors' explanation based on cell-type dependence is not sufficient. If this explanation is accepted, it becomes difficult to positively interpret experimental results obtained using BJ-hTERT knockout cells other than patient-derived cells. A mechanism specific to Y1758C has not been shown. Rather, the data should be interpreted as part of pre-existing ATRX dysfunction. Thus, the novelty has been weakened in the rebuttal, but this limitation is not sufficiently reflected in the manuscript.

2. The relationship among ATRX genotype, ATRX protein level and IFN response remains unresolved

Both reviewers raised concerns that ATRX protein levels or mutation type do not correlate with IFN score or IFNB1 expression. The manuscript states that P1/P2 retain ATRX protein expression but show increased IFN score and IFNB1 expression. It also states that, among primary fibroblasts from patients with classical ATR-X syndrome, IFN score and IFNB1 expression were also increased in two of four cases. The discrepancy in loss of function between ATRX-Y1758C and canonical ATRX mutations is not sufficiently explained at the level of patient-cell phenotypes. The authors mention genetic background and stochastic chromatin accessibility/gene expression in the rebuttal and Discussion, but this remains an unvalidated explanation rather than an experimentally supported conclusion. Therefore, the conclusion that ATRX deficiency generally drives IFN signalling should be treated with caution. Again, given that data contradictory to the authors' model have been reported, including Floyd JCI (2023), the authors should avoid explaining critical patient-cell phenotypes mainly by differences in cell type.

3. The mechanism of the non-canonical nuclear function of cGAS remains unresolved

Both reviewers were concerned that it remains unclear how cGAS alters DAXX/H3.3 distribution or ISG transcription. The authors explain cGAS dependence and cGAMP/STING independence in the rebuttal. However, this is an interpretation of data that had already been presented previously, and the current revision does not newly clarify the direct mechanism of cGAS. The authors state that how cGAS contributes to chromatin remodelling associated with ATRX deficiency remains to be addressed in future studies. The issue is not sufficiently resolved. The mechanistic conclusion that cGAS regulates transcription through ATRX/DAXX/H3.3 remains not directly demonstrated.

4. Alternative mechanisms have not been sufficiently excluded

Both reviewers raised concerns that the observed IFN upregulation may not be due to ATRX-Y1758C itself, but could instead be explained by other interferonopathy causes, ERV/repeats, ALT/telomere DNA, genome instability, or cell culture/CRISPR selection artifacts. The only addition in the current manuscript is the statement in the Discussion regarding Telomere-FISH, namely that cytoplasmic telomere foci were not detected and that canonical DNA sensing through cytosolic leakage of telomere fragments is therefore unlikely. However, this only addresses part of the possibility of ALT/telomere-derived DNA sensing, and other experimental examinations are not sufficiently presented. Many of the major alternative mechanisms raised by Reviewers#2 and #4 have not been experimentally excluded.

5. The link between the clinical phenotype and IFN signature remains insufficiently established

Both reviewers raised concerns that the manuscript overstates the extent to which the IFN signature explains neuroinflammation or systemic inflammation in patients with ATR-X syndrome. The manuscript shows persistent ISG upregulation in P1/P2 and describes systemic inflammation, elevated CSF neopterin and a treatment course including ruxolitinib in P3. However, P3 is also described as having a diagnosis of subcutaneous panniculitis-like T-cell lymphoma, making it difficult to attribute the inflammatory phenotype simply to the ATRX/IFN axis. The manuscript shows that an IFN signature is observed in a subset of patients with ATR-X syndrome, but it does not sufficiently demonstrate that this is the main cause of neuroinflammation or systemic inflammation.

Concerns specific to Reviewer#2

6. Exclusion of other AGS/interferonopathy causes for the IFN phenotype in P1/P2

The most important concern of Reviewer#2 on this point is whether the IFN phenotype in P1/P2 can be attributed to ATRX-Y1758C, rather than to an independent defect in another AGS/interferonopathy-related gene. Genetic tests have increasingly revealed cases in which more than one rare disorder coexists in the same patient. The manuscript states that WES identified no putative mutations other than ATRX. However, WGS and RNA-seq were not performed, and even confirmation of the expression of some interferonopathy-related proteins, which does not appear technically difficult, was not performed. The authors argue that it is not practical to comprehensively examine for more than 50 interferonopathy-related genes. However, the concerns could be addressed by only using the available patient materials. WGS using patient gDNA may identify overlooked variants in known interferonopathy-related genes. RNA-seq could also directly assess reduced expression or splicing abnormalities of these genes. Deep intronic variants, regulatory variants, structural variants and splicing abnormalities cannot be sufficiently detected by WES. Therefore, the absence of obvious coding pathogenic variants in AGS1-9 by WES is not sufficient to exclude alternative interferonopathy causes. The authors provide explanations in the rebuttal, but no experimental differential diagnosis has been added.

7. Functional distinction between Y1758C and ATRX-null/previously reported missense mutations

Reviewer#2 asked whether Y1758C can be distinguished from ATRX-KO/null or previously reported missense mutations. The manuscript presents comparisons among WT, Y1758C-KI and ATRX-KO in an isogenic BJ-hTERT model, showing increased ISG/IFNB1 expression and shared DEGs in both KI and KO cells. This is a useful comparison, but it does not show that Y1758C has a novel mechanism distinct from ATRX-null. Rather, the manuscript suggests that Y1758C largely recapitulates ATRX loss of function, similarly to other ATRX-deficient states. Therefore, a disease mechanism specific to Y1758C cannot be claimed.

Reviewer #5 (Remarks to the Author):

In light of the comments by reviewer 1 and 3 and the extensive revisions to address the comments by reviewer 2 I would support publication of the manuscript. The key issues outlined regarding mechanism can be discussed in the discussion sections and highlight the potential mechanisms cGAS uses to drive the IFN phenotype.

Reviewer #6 (Remarks to the Author):

The authors carefully addressed Reviewer 4´s concerns and clarified potential misunderstandings.

Responses to reviewers' comments

Reviewer #2:

1. A novel disease mechanism specific to ATRX-Y1758C has not been demonstrated

Response: Beyond the fact that this mutation is associated with retained levels of ATRX protein, we do not claim that the Y1758 mutation is 'mechanistically distinct'. Rather, we show that this mutation acts as a loss-of-function, similar to other ATR-X syndrome causing mutations (here, for brevity, we highlight the data in Fig. 1e and 1f where we show that, as previously reported for two other ATR-X syndrome causing mutations in the SNF2 domain, V1552F and K1650N, Y1758C ATRX is defective in ATPase and translocase activity. Thus, as stated in our initial rebuttal, "*Y1758C leads to a loss of ATPase activity as described for other 'classical' ATR-X syndrome associated mutations (PMID: 21505078; 14990586)*", also stating

that *“we propose neither qualitative nor quantitative differences between the Y1758C substitution and other ATR-X syndrome associated mutations per se.*” Consistent with this, looking at transcriptional changes in BJ-hTERT fibroblasts KI (Y1758C) or KO for *ATRX*, we observed significant overlap in differentially expressed genes compared to control cells (Fig. 2f).

Overall, the novelty of our paper is twofold: 1) our demonstration, for the first time, that ATR-X syndrome causing mutations can be associated with an induction of type I IFN signalling (which induction is likely responsible for some aspects of the pathology shown in certain patients); 2) that, while this induced type I IFN signalling is cGAS-dependent, its induction is independent of cGAMP – thereby highlighting a previously unrecognised non-canonical role for cGAS in transcriptional regulation.

2. The relationship among ATRX genotype, ATRX protein level and IFN response remains unresolved

Response: We do not claim a direct inverse correlation between ATRX protein levels and IFN signalling. We agree that the data on IFN signalling in patient primary fibroblasts show some variability, which we suggest relates to two factors: firstly, primary cells are notoriously difficult to work with, leading us to turn to more ‘controlled’ cellular models. Thus, in our manuscript we state that “*Given the potential variability of genetic background in available patient material, we introduced the Y1758C mutation (referred to as knock-in (KI)) into BJ-5ta immortalised fibroblasts (referred to as BJ-hTERT), and also generated ATRX knock-out (KO) clones using CRISPR/Cas9 technology*.”; secondly, the available evidence suggests that variability in inflammatory signalling in patient material reflects the stochastic nature of perturbed chromatin accessibility and gene expression. This phenomenon is already well-established in the context of ATRX; best illustrated by the finding that only a proportion of patients with germline mutations in *ATRX* manifest indices of alpha thalassemia (PMID: 18409179). Following up on this observation, in demonstrating a function for ATRX on both heterochromatin and euchromatin, Richard Gibbons (a co-author on our paper) (PMID: 35710802) went on to show that the role of ATRX at the well-characterized α-globin locus in erythroid cells leads to stochastic changes in gene expression and cell fate in only a subset of cells (mediated through interdependent effects on DNA replication, chromatin modification and chromatin accessibility).

3. The mechanism of the non-canonical nuclear function of cGAS remains unresolved

While we agree that uncertainty remains as to the precise mechanisms involved, our findings suggest a model where the loss of ATRX function affects chromatin landscape, leading to the altered distributions of DAXX and H3.3, and associated changes in gene transcription. In this context, cGAS exerts a non-canonical role in regulating chromatin remodelling mediated by ATRX, a process that occurs independently of the activation state of cGAS.

4. Alternative mechanisms have not been sufficiently excluded …..the observed IFN upregulation may not be due to ATRX-Y1758C itself, but could instead be explained by other interferonopathy causes, ERV/repeats, ALT/telomere DNA, genome instability, or cell culture/CRISPR selection artifacts.

While ATRX is well known to repress ERVs, this function was mostly observed in stem cells, where the role of ATRX in maintaining DNA methylation at early developmental stages is critical to silence repetitive elements (PMIDs: 25938714, 26340527). Given that the functions of ATRX are likely to be cell-type and context specific, we have no evidence that ATRX loss activates ERVs in terminally differentiated cells, such as the fibroblasts that we use. More relevant still, in most situations currently described to date, ERVs trigger IFN signalling through

a cGAMP-dependent mechanism (e.g. PMIDs: 36610399; 38991060; 39446896), a scenario that we show does not apply in the situation that we describe.

Extrachromosomal telomere repeat (ECTR) DNA inducing type I IFN signalling described in PMID 29106411 is cGAS-STING and phospho-IRF3 mediated (as shown in their Figure 3) – which is not the case in our data. In agreement, we did not detect cytoplasmic telomere foci by Telomere-FISH (Fluorescence *In Situ* Hybridization). This contrasts with the ALT positive cells shown in Figure 1a in PMID 29106411. These data, included in our manuscript as Fig. S9b, argue against the leakage of telomere fragments into the cytosol activating canonical DNA sensing.

We acknowledge that ATRX loss can compromise genomic stability (PMIDs: 18227278, 23563309), and we have explored this in our own work (Fig. S9a). However, the lack of cGAMP production (which would be expected to be produced if genomic DNA fragments were activating the canonical cGAS enzymatic pathway), and the demonstration that the interferon response is independent of cGAS catalytic activity, suggests that DNA damage is not the primary driver of the ISG upregulation observed here.

To mitigate the risk of clonal selection bias or stochastic secondary mutations, we utilized 3 to 6 independent CRISPR-engineered clones for each genotype and experiment. By prioritizing only the phenotypes and transcriptomic changes that were consistently present across all independent clones, we have minimized the likelihood that our observations are the result of random mutational burden or prolonged selection pressure.

5. The link between the clinical phenotype and IFN signature remains insufficiently established

We politely disagree with the reviewer, and consider the relationship between the IFN signature in our three patients with ATRX mutations, to be quite consistent with what we and others have described in multiple other type I interferonopathies.

6. Exclusion of other AGS/interferonopathy causes for the IFN phenotype in P1/P2

Given the extensive genetic and functional data that we present (summarised in our previous rebuttal), we are unsure why the reviewer does not accept that the variants that we have observed in ATRX are pathogenic mutations. The evidence in favour of the variants in *ATRX* being causative are overwhelming, noting here that all three patients have had confirmatory genetic testing in accredited diagnostic laboratories in the UK, France and the Netherlands, and that the two patients tested have also been shown to exhibit a genomic DNA methylation signature diagnostic of ATRX syndrome (performed and signed off by an accredited diagnostic laboratory in the UK i.e. the NHS, and in the Netherlands).

Summarising, there are multiple lines of evidence supporting the pathogenicity of Y1758C: Genetic: The variant is very rare i.e. it is not present on more than 1,100,000 alleles sequenced on gnomAD. In contrast, we identified two unrelated individuals (one from the UK, one from France) with this variant, both of whom manifest a similar neuroinflammatory phenotype associated with enhanced type I IFN signalling in blood. Phenotypic: Patient 2 has classical facial features of ATRX syndrome. Further, this patient demonstrates a genomic DNA methylation signature diagnostic of ATRX syndrome (see PMIDs: 29304373; 28293299). Experimental: Our biochemical studies show that the Y1758C substitution in ATRX is hypomorphic for ATPase activity, and is associated with a marked reduction of translocase activity (as described in Mitson et al. (PMID: 21505078) and Tang et al. (PMID: 14990586) for other mutations of ATRX associated with retained protein expression). Further, our functional studies show that the Y1758C induces enhanced type I IFN signalling both ex vivo (using patient-derived cells) and in vitro (using a variety of cell models).

7. Functional distinction between Y1758C and ATRX-null/previously reported missense mutations … a disease mechanism specific to Y1758C cannot be claimed

Please see our response to the reviewer’s Point 1 above. To reiterate, we do not claim, and have never claimed, that the Y1758 mutation is mechanistically distinct.

Reviewer #5:

No comments.

Reviewer #6:

No comments.
