# Supplementary material for: ATRX deficiency drives aberrant type I interferon signalling through cGAS-dependent transcriptional dysregulation
Source: Nat Commun. Author manuscript; Available in PMC 2026 Oct 25. (PMC7619637; doi:10.1038/s41467-026-78094-8)
Supplement: Supplementary file 1 [file EMS219617-supplement-Supplementary.docx]

**SUPPLEMENTARY FIGURES**

**Figure S1. Clinical features and interferon scores in P1 and P2**

**a** Facial appearance of P2. **b** Cerebral imaging in P2: MRI imaging at seven years of age shows a large area of abnormal white matter signal in the right fronto-parietal region, with a cystic area within it (a) (axial T2), and extensive, bilateral but asymmetric, mineralisation in the basal ganglia (b, c) (axial SWI), which was confirmed to be calcification by CT (d: taken at age of five years). **c** Cerebral imaging in P1 at 14 years of age: MRI imaging demonstrates a large cystic lesion in the left parietal region with heterogeneous signal intensity (a: axial T2, b: axial FLAIR) with surrounding contrast enhancement (c: coronal post-contrast T1). There is dense mineralisation of the basal ganglia bilaterally (d: axial GRE) and in the left cerebellar hemisphere (e: axial GRE). **d-e** Relative quantification (RQ) values of 24 interferon stimulated genes (ISGs) measured on a NanoString platform in whole blood of P1 (d) and P2 (e). The first number in brackets is the decimalised age in years at sampling, the second number is the interferon (IFN) score, with scores above 2.75 designated as positive. Dark blue bars represent the composite data of 27 controls. Note that the IFN score in whole blood of the mother of P1 was normal on the single occasion tested. **f** Left: dot plot showing the IFN score calculated from the median fold change of six ISGs (*IFI27, IFI44L, IFIT1, ISG15, RSAD2, SIGLEC1*) measured by qPCR in whole blood of controls, P1 and P2, and related to Figure 1d. Right: dot plot representation of the data shown in d-e. Red = P1, green = P2. Blask bar: median.

Source data are provided as a Source Data file.

**Figure S2. Gene set enrichment analysis (GSEA) of publicly available transcriptomic datasets relating to three ATRX deficient models, and clinical features of P3**

**a** Normalised enrichment scores (NES) of GSEA values with a p value < 0.05 of RNA-Seq data in *ATRX*-null induced pluripotent stem cells (iPSCs) (top), microarray data in *Atrx*-null mouse forebrain (middle) and RNA-Seq data in *Atrx*-null mouse neuronal progenitor cells (NPC) (bottom). Interferon-related pathways are highlighted in orange. Note: genes from the interferon gamma dataset overlap with the interferon alpha dataset. Transcriptional data were obtained from the Gene Expression Omnibus (GEO) repository, with the accession numbers GSE107878^1^, GSE12454^2^ and GSE100464^3^ as indicated above each data set. **b** Image showing the inflammatory skin lesions observed in P3. Note the appearance of the mouth, typical of ATR-X syndrome. **c** CLUSTAL Omega alignment of ATRX homologs. The position of the substituted A2137T in P3 is indicated in green.

**Figure S3. ATRX protein levels in patient primary fibroblasts and purified from *Saccharomyces Cerevisiae***

**a** Schematic of the ATRX protein (top) illustrating the mutations in P1 – P7; ADD = ATRX-DNMT3-DNMTL-like domain (amino acid (aa) residues 161-292; SNF2 (Sucrose Non-Fermentable) domain encompassing ATPase/Helicase domains, residues 1550-2226. Representative western blot (middle) and quantification (bottom) of ATRX protein in primary fibroblasts from controls (C) and indicated patients (P), [each circle represents an independent replicate, Kruskal-Wallis test, two-stage linear step-up procedure of Benjamin, Krieger and Yekutieli, q values are indicated, mean +/- SEM]. **b** Purification of recombinant human ATRX protein in budding yeast. SDS-PAGE of 1 μg of pooled fractions from WT, K1600R, and Y1758C ATRX, stained with InstantBlue Coomassie protein stain.

Source data are provided as a Source Data file**.**

**Figure S4. Characterisation of ATRX deficient cell lines**

**a** Quantification of secreted type I interferon in supernatant of BJ-hTERT fibroblasts KI or KO for *ATRX*, measured using a secreted embryonic alkaline phosphatase assay in HEK-Blue^TM^ ISG reporter cells. As a positive control, HEK-Blue cells were treated with 200 U/mL IFNa2 for 24 hours, [four independent experiments, Ordinary one-way ANOVA, two-stage linear step-up procedure of Benjamin, Krieger and Yekutieli, q values are indicated, mean +/- SEM]. **b** ATRX protein expression assessed by western blot in THP-1 cells following knock-down of *ATRX* using three short-hairpin (sh) RNAs. **c** Interferon (IFN) score (median expression of *IFI27, RSAD2, ISG15, IFI44L, IFIT1*) in THP-1 cells five days post-transduction with control short-hairpin (sh-controls) or three different sh-RNAs targeting *ATRX* (sh-ATRX), [each circle represents an independent repeat of an sh-RNA, n=4, ordinary one-way ANOVA, Dunnett’s multiple comparisons test, p values are indicated, black bars represent the median]. **d** Quantification of secreted type I interferon activity in supernatant from THP-1 cells upon knock-down of *ATRX* by sh-RNA (day 8 post-transduction), same as (a), [each circle represents a different sh-RNA, n=1, unpaired t-test]. **e** Schematic of differentiation of human iPSCs (induced Pluripotent Stem cells) to NPCs (Neural Stem cells) and then to forebrain neurons following neural induction and neuronal maturation using an established StemCell protocol. **f** Relative gene expression levels (expression in iPSC is set to 1) of stem cell and neural subtype markers measured by 3’ end mRNA sequencing in iPSCs, NPCs and neurons (Neu) WT or *ATRX* KI for the Y1758C substitution.

Source data are provided as a Source Data file.

**Figure S5. Interferon signalling pathway analysis**

**a** Efficiency of *MAVS* RNA knock-down in primary fibroblasts [three independent experiment]. **b** Effect on the interferon (IFN) score (median expression of *TNFAIP3, RSAD2, Mx1, ISG15, IFI44L, IFIT1, IFI6*) of si-RNA knock-down of *MAVS* in primary fibroblasts from controls, Y1758C ATRX mutant (P1 and P2) and ‘classical’ ATRX (P6) mutant fibroblasts, [circles represent individual repeats, Ratio paired t tests, q values are indicated]. **c** Protein expression assessed by western blot of MAVS (top) and ATRX (bottom) in THP-1 cells KO for *MAVS*, following knock-down of *ATRX* using three short-hairpin (sh) RNAs. **d** Interferon (IFN) score (median expression of *IFI27*, *IFIT1*, *RSAD2*, *ISG15*) in THP-1 cells five days post-transduction with control sh-RNAs (sh-controls) or three different sh-RNAs targeting *ATRX* (sh-ATRX) [each circle represents the mean of an independent experiment, n=4; Unpaired t test, Two-stage linear step-up procedure of Benjamini, Krieger and Yekutieli, q values are indicated, mean +/- SEM]. **e** Protein levels assessed by western blot in nuclear fractions of BJ-hTERT clones WT or KO for *CGAS*. **f-g** Principal component (PC) analysis of RNA-Seq data derived from four BJ-hTERT fibroblasts clones KO (f) or WT (g) for *CGAS*, and WT, KI or KO for *ATRX*. The number of differentially expressed genes (DEG, p adjusted < 0.1) identified between WT and KI or WT and KO *ATRX* cells are noted on the graph.

Source data are provided as a Source Data file.

**Figure S6. Canonical DNA sensing pathway analysis**

**a-b** Graphs illustrating total cGAMP levels measured by ELISA in wells containing total cellular lysate obtained from primary fibroblasts (a) or BJ-hTERT clones (b): dotted lines indicate the level of IC20 and IC80, representing the upper and lower limits of detection of the standard curve, respectively. Dots represent individual measurements and relate to Figure 4a and 4b. **c-d** Western blot (c) and qPCR (d) showing protein and RNA levels, respectively, of transfected WT or catalytic inactive (D319A mutant) cGAS constructs. **e** Protein levels assessed by western blot of BJ-hTERT fibroblasts clones WT or KO for *STING1*. **f** cGAMP levels measured in BJ-hTERT fibroblasts KO for *STING1* unstimulated (-) or stimulated (+) with HT-DNA for four hours, [one experiment, three replicates]. **g-h** *IFNB1* gene expression (g) and interferon (IFN) score (h) (median expression of *IFI27, IFI6, IFIT1, OAS1, ALDH1A1, IFIH1*) in BJ-hTERT WT or KO for *STING1*, unstimulated (NT), and stimulated with STING agonist diABZI or the MAVS agonist p (I:C) (synthetic analogue of double-stranded RNA polyinosinic-polycytidylic acid). **i** IFN score (median expression of *IFI27, IFI6, IFIT1, OAS1, ALDH1A1, IFIH1*) (left) and relative expression of *IFNB1* (right) measured by qPCR in BJ-hTERT fibroblasts KO for *STING1*, and WT, KI or KO for *ATRX* [each circle represents the mean of an individual clone, n=3, Kruskal-Wallis test, two-stage linear step-up procedure of Benjamin, Krieger and Yekutieli, mean +/- SEM]. **j** Left: Immunoblot of phosphorylated STAT1 (Y701), STING (S366), phosphorylated IRF3 (S396), total IRF3 and STING in THP-1 cells WT or KO for *IRF3*, transduced with control sh-RNAs or sh-RNAs targeting *ATRX*. The last lane relates to WT THP-1 cells stimulated with HT-DNA for four hours; Right: Quantification of STAT1 phosphorylation level over vinculin, [two independent experiments, circles are individual repeats, black bars indicate the means, Kruskal-Wallis test, two-stage linear step-up procedure of Benjamin, Krieger and Yekutieli, q values are indicated, black bars are median]. **k** IFN score (median expression of *IFI27*, *IFIT1*, *RSAD2*, *ISG15*) in THP-1 cells five days post-transduction with controls sh-RNA (sh-control) or three different sh-RNAs targeting *ATRX* (sh-ATRX) [each circle represents an sh-RNA, one independent experiment, Mann-Whitney tests, q values are indicated, mean +/- SEM]. Source data are provided as a Source Data file.

**Figure S7. Characterization of ATRX Y1758C chromatin binding**

**a** Subcellular fractionation of BJ-hTERT fibroblasts WT or KI for *ATRX*. Cells were separated into cytosolic and nuclear fractions, followed by sequential elution of nuclear pellets with the indicated concentrations of NaCl. ATRX, vinculin and H3 were assessed by western blot. **b** Principal component (PC) analysis comparing the ATRX genomic binding profiles between indicated genotypes.

**Figure S8. Loss of ATRX function disrupts DAXX and H3.3 occupancy at genes**

**a-b** Volcano plots displaying the results of a genome-wide differential binding analysis of DAXX between KI (a) or KO (b) and WT *ATRX* BJ-hTERT fibroblasts; grey, purple and green dots represent biding sites (peaks) that are, respectively, unchanged, depleted or enriched (p value < 0.05) in ATRX KI or KO cells. **c** DAXX occupancy assessed by CUT&Tag at +/- 2 kb of transcription start sites (TSS) of EMT-related genes dysregulated in BJ-hTERT fibroblasts KI and KO for *ATRX*, as compared to WT. **d-e** H3.3 occupancy assessed by CUT&Tag at +/- 2 kb of transcription start sites (TSS) of genes upregulated (d) or downregulated (e) in BJ-hTERT fibroblasts KI and KO for *ATRX*, as compared to WT. **f-g** Principal component (PC) analysis comparing DAXX (f) and H3.3 (g) genomic binding profiles between indicated genotypes. Source data are provided as a Source Data file.

**Figure S9. Features of genomic instability in ATRX patient fibroblasts**

**a** Staining of gamma-H2AX (gH2AX) (left) and foci quantification (right) detected using the FindFoci (GDSC) plugin and measured in three controls and patient primary fibroblasts, [two independent experiments, total number of cells analysed are 941 in controls, 201 in P1, 505 in P2, 258 in P5, 293 in P6, 178 in P7, 110 in P4, Ordinary one-way ANOVA, two-stage linear step-up procedure of Benjamin, Krieger and Yekutieli, p values are indicated, violin plot, min to max, show all points]. **b** Analysis of telomeric DNA by fluorescence in situ hybridization (Tel-FISH) (cyan) in three controls and patients P1 and P2 primary fibroblasts, and nuclei were visualized by DAPI staining (magenta), [two independent experiments, total cells numbers are 340 in controls, 147 cells in P1 and 279 cells in P2, Ordinary one-way ANOVA, two-stage linear step-up procedure of Benjamin, Krieger and Yekutieli, q values are indicated, mean+/-SEM]. White bars represent a scale of 10 μm. Source data are provided as a Source Data file.

**Uncropped western blots for supplementary figures.**

For all blots, this ladder was used: Chameleon duo pre-stained protein ladder (Licor P/N: 928-60000).

**Figure S3a.**

Red channel ATRX (280 kDa)	Green channel (vinculin: 120 kDa)

**Figure S4b.**

Green channel (vinculin: 120 kDa)	Green channel ATRX (280 kDa)

**Figure S5c.**

Green channel (MAVS: 70 kDa)	Green channel (vinculin: 120 kDa)

Low exposure	High exposure

Green channel (vinculin: 120 kDa)	Red channel (ATRX: 280 kDa)

**Figure S5e.**

Green channel (cGAS: 62 kDa, H3: 17 kDa)	Red channel (ATRX: 280 kDa)

**Figure S6c.**

Green channel (cGAS: 62 kDa, high exposure)	Green channel (vinculin: 120 kDa, low exposure)

**Figure S6e.**

Green channel

vinculin: 120 kDa, STING 38 kDa, low exposure	cGAS: 62 kDa, high exposure

**Figure S6j.**

IRF3 WT	IRF3 KO

Green channel, vinculin: 120 kDa, IRF3 50 kDa

Green channel, P-STING: 40 kDa

Red channel, STING: 40 kDa

Green channel, P-STAT1: 90 kDa

Green channel, P-IRF3: 50 kDa

**Fig. S7a**

cGAS KO ATRX WT	cGAS KO ATRX KI

Red channel (ATRX: 280 kDa)

Red channel (H3: 17 kDa)

Greev channel (vinculin: 120 kDa)

**REFERENCES**

1.	Deneault, E. *et al.* Complete Disruption of Autism-Susceptibility Genes by Gene Editing Predominantly Reduces Functional Connectivity of Isogenic Human Neurons. *Stem Cell Reports* **11**, 1211-1225 (2018).

2.	Levy, M.A., Fernandes, A.D., Tremblay, D.C., Seah, C. & Berube, N.G. The SWI/SNF protein ATRX co-regulates pseudoautosomal genes that have translocated to autosomes in the mouse genome. *BMC Genomics* **9**, 468 (2008).

3.	Danussi, C. *et al.* Atrx inactivation drives disease-defining phenotypes in glioma cells of origin through global epigenomic remodeling. *Nat Commun* **9**, 1057 (2018).

**SUPPLEMENTARY NOTES**

- Full DNA sequence of the ATRX-Yeast plasmid generated in this study:

ATRX_Y_Thermo (13889 bp), 1/23 to 23/23

- DNA sequence of the TFO-containing plasmid generated in this study:

25AEECQD_4160830_TFObindingsite_annotatedbyAF
